# Synthesis and Antiviral Evaluation of 5-(4-Aryl-1,3-butadiyn-1-yl)-uridines and Their Phosphoramidate Pronucleotides

**DOI:** 10.3390/molecules30010096

**Published:** 2024-12-29

**Authors:** Evan Saillard, Otmane Bourzikat, Koffi Assa, Vincent Roy, Luigi A. Agrofoglio

**Affiliations:** Institute of Organic and Analytical Chemistry (ICOA UMR 7311), CNRS, University of Orleans, F-45067 Orléans, France

**Keywords:** ribonucleosides, phosphoramidate pronucleotide, 5-substituted-uridines, Cadiot–Chodkiewicz cross-coupling, antiviral activity

## Abstract

The emergence of RNA viruses driven by global population growth and international trade highlights the urgent need for effective antiviral agents that can inhibit viral replication. Nucleoside analogs, which mimic natural nucleotides, have shown promise in targeting RNA-dependent RNA polymerases (RdRps). Starting from protected 5-iodouridine, we report the synthesis of *hitherto unknown C5*-substituted-(1,3-diyne)-uridines nucleosides and their phosphoramidate prodrugs. The modifications at *C5* include 4-(trifluoromethyl)benzene (**a**), 4-pentyl-benzene (**b**), 3,5-dimethoxy-benzene (**c**), 4-(trifluoromethoxy)benzene (**d**), 3-aniline (**e**), 4-pyridine (**f**), 3-thiophene (**g**), C_6_H_13_ (**h**), 2-pyrimidine (**i**), cyclopropyl (**j**), and phenyl (**k**) groups. These compounds were synthesized using Sonogashira palladium-catalyzed reactions and nickel–copper-catalyzed C-H activation between various alkynes, yielding between 25% and 67%. The antiviral activities of obtained compounds were measured through HTS against RNA viruses including influenza H1N1 and H3N2, human respiratory syncytial virus (RSV), SARS-CoV-2, Zika, hepatitis C virus (HCV), Hepatitis E virus (HEV), as well as against coronavirus (HCoV-229E). Unfortunately, none of them showed promising antiviral activity, with less than 85% inhibition observed in the cell viability screening of infected cells.

## 1. Introduction

**Figure 1:**
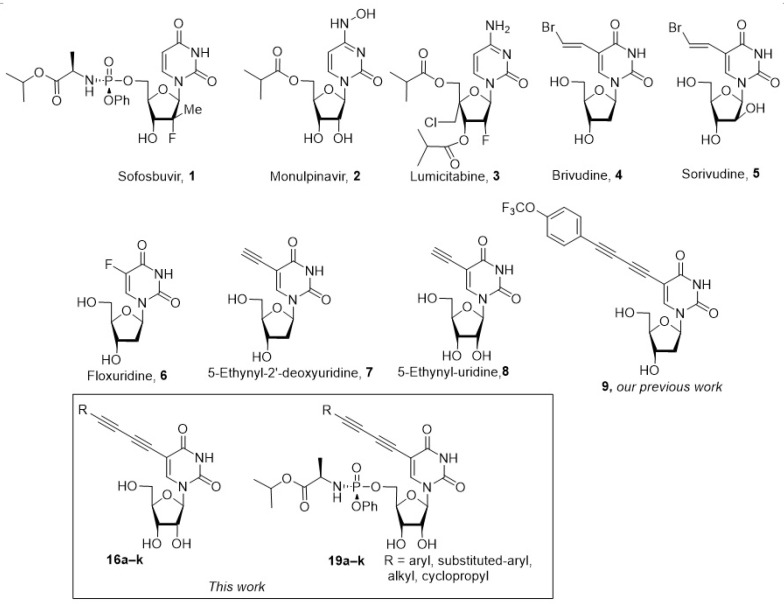
Some modified pyrimidine nucleoside antivirals and target compounds (**16a**–**k** and **19a**–**k**) [21].

Emerging and re-emerging RNA viruses have driven efforts to discover new small molecules that can block their replication. As the global population grows and international trade increases, new viruses continue to emerge, particularly RNA viruses associated with severe diseases and epidemics in the 21st century. SARS-CoV, DENV, Chikungunya, and H1N1 influenza, among others, have resulted in significant global outbreaks [1,2]. The urgency to discover effective antiviral agents has never been greater. In this context, nucleoside analogs have demonstrated significant potential as a key class of antivirals against DNA and, more recently, RNA viruses, as they disrupt viral replication by mimicking natural nucleotides [3,4,5]. If nucleoside analogs can target several cellular enzymes, their primary and major antiviral target is the viral polymerases, specifically the RNA-dependent RNA polymerases (RdRps) in RNA viruses, which share a degree of structural similarity [6,7] that facilitates the discovery of broad-spectrum antivirals [8]. Sofosbuvir **1**, a uridine analog prodrug bearing a bulky 2′-methyl group, is the standard of care for HCV treatment approved in 2013 [9]; molnupiravir **2** (EIDD-2108) [10] and lumicitabine **3** (ALS-8176) [11] are two nucleoside analog prodrugs bearing, respectively, a modification on the base moiety and on the sugar moiety at 2′ and 4′ positions (Figure 1). Those compounds are delivered as pronucleosides (for molnupiravir and lumicitabine, respectively); meanwhile, sofosbuvir is a nucleotide analog prodrug that belongs to the phosphoramidate class [12,13]. Among the various families of these analogs, *C5*-modified pyrimidine nucleosides [14] are particularly notable for their therapeutic potential mainly against DNA viruses [15] and as fluorescent probes [16]. *C5* modifications often involve adding functional groups, such as halogens, nitro and amino groups, alkyl, aryl, or hydrophobic groups, which enhance the molecule’s antiviral and anticancer properties. For instance, brivudine **4** (5-(*E*)-bromovinyl-2′-deoxyuridine) [17] and its *arabinose* derivative (sorivudine, **5**) [18] are potent drugs used to treat herpes simplex virus (HSV-1), varicella-zooster virus (VZV) and Epstein Barr virus (EBV) infections; meanwhile, floxuridine **6** (5-fluoro-2′-deoxyuridine) [19] is widely used in chemotherapy of colorectal, kidney, and stomach cancers. The 5-ethynyl-2′-deoxyuridine **7** [20] is also active against HSV-1 strain LYONS and KOS, whereas its ribosyl analogue **8** lacks antiviral activity but can be used for labeling of nascent RNA inside cells. Finally, our group has reported that 5-[4-(4-trifluoromethoxyphenyl)buta-1,3-diynyl]-2′-deoxyuridine **9** [21] exhibits activity against VZV, with an EC_50_ of 1 µM and a CC_50_ of 55 µM. These specific *C5* modifications play a crucial role in enhancing the stability, specificity, and efficacy of the molecules, making *C5*-modified nucleoside analogs promising candidates in the development of new therapeutic agents.

Based on the state of the art, *C5* modifications of pyrimidines generally carry nonpolar groups. Thus, as part of our drug discovery program, we report here the synthesis and antiviral evaluation of novel *C5*-substituted-(1,3-diyne)-uridine derivatives **16a**–**k** and their phosphoramidate analogs **19a**–**k**.

## 2. Results and Discussion

### 2.1. Chemistry

**Scheme 1:**
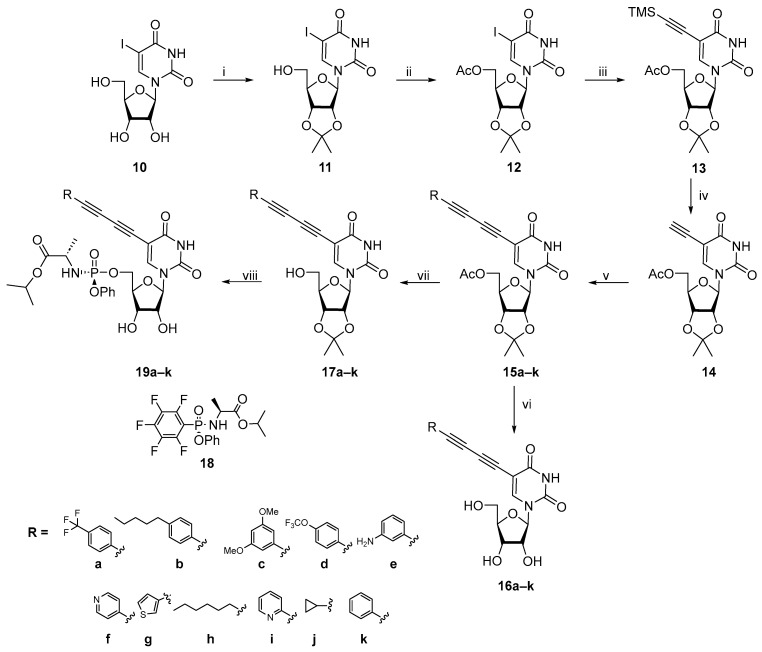
Reagents and conditions: (i) dry acetone, H_2_SO_4_, 4 Å molecular sieves, r.t., 48 h, 99%; (ii) Ac_2_O, pyridine, r.t., 15 h, 90%; (iii) ethynyltrimethylsilane, CuI/PdCl_2_(PPh_3_)_2_, Et_3_N, dry DMF, MW, 60 °C, 25 min, 90%; (iv) TBAF, THF, r.t., 3 h, quant; (v) alkyne, NiCl_2_·6H_2_O, CuI, TMEDA, O_2_, THF, r.t., 24 h, 31–67%; (vi) HCl 37%, MeOH, r.t., 24 h, 51–88%; (vii) NH_3_/MeOH (7N), MeOH, r.t., 24 h, 71–100%; (viii) (a) **18**, t-BuMgCl (1.7 M in THF), THF, 0 °C to r.t., 20 h; (b) HCl 37%, THF, r.t., 6 h, 28–84%.

Based on our previous article [21], we elaborated on the synthesis of a small library of *C5*-substituted-1,3-diynyl-uridine by metal-catalyzed alkyne C-H activation on the *C5*-ethynyl intermediate **14** with various alkynes (Scheme 1). Starting from the commercially available 5-iodouridine (**10**), the 2′ and 3′ position were protected with an *iso*propylidene group using dry acetone and sulphuric acid in the presence of 4 Å molecular sieves to afford **11** in quantitative yield, (Scheme 1). After acylation of the 5′-OH group using acetic anhydride in pyridine, the fully-protected compound **12** is isolated in 90% yield. The first key reaction consists of a Sonogashira cross-coupling reaction [22,23], with trimethylsilylacetylene (TMS) in the presence of Et_3_N, CuI and PdCl_2_(PPh_3_)_2_ in anhydrous DMF, activated by microwave irradiation at 60 °C for 25 min, yielding the desired product **13** in 90%. Compound **13** was desilylated using TBAF for 2 h to yield pure **14** quantitatively.

Compound **14** was then subjected to Cadiot–Chodkiewicz cross-couplings [24,25,26] with various commercially available EDG- and EWG-substituted aromatic and non-aromatic alkynes to generate unsymmetrical buta-1,3-diynes **15a**–**k**. Compared to other catalytic systems, the Cadiot–Chodkiewicz reaction favors the heterocoupling of different alkynes. The chosen terminal alkynes included 4-(trifluoromethyl)phenyl (**a**), 4-pentylphenyl (**b**), 3,5-dimethoxyphenyl (**c**), 4-(trifluoromethoxy)phenyl (**d**), 3-aminophenyl (**e**), 4-pyridyl (**f**), 3-thienyl (**g**), *n-*hexyl (**h**), 2-pyridyl (**i**), cyclopropyl (**j**), and phenyl (**k**), all explored using the same procedure. The Cadiot–Chodkiewicz cross-coupling reaction was performed under our optimized conditions [20], involving NiCl_2_·6H_2_O (5 mol%), CuI (5 mol%), and TMEDA (20 mol%) in THF under an oxygen atmosphere for 20 h at room temperature, yielding moderate to good yields (31–67%) of eleven analogues (**15a**–**k**). As observed in Table 1, the yield of the cross-coupling reaction depends on the acidity of the alkyne protons. Indeed, with deactivated aromatic alkynes or with aliphatic-substituted alkynes, the Cadiot–Chodkiewicz cross-coupling is less effective (entries 3, 6, 8, 10), resulting in decreased yields due to homodimerization of the aliphatic alkynes for the entries 8 and 10 and due to a low conversion for the entries 3 and 6.

Removal of the *iso*propylidene moiety from **15a**–**k** using a large excess of 37% HCl in methanol for a minimum of 16 h afforded the desired final nucleosides (**16a**–**k**) in good to excellent yields (51–88%). To reach the phosphoramidate analogs, **15a**–**k** were deacetylated in a methanolic ammonia solution (7*N*), yielding the expected nucleoside analogs **17a**–**k** in good yields; extraction was sometimes necessary to remove the formed acetamide. The 5′-OH group of **17a**–**k** was phosphorylated using a reported procedure employing *t*-BuMgCl (1.7 M in THF) and the commercially available chiral phosphoramide **18** in dry THF for 20 h. Afterward, after completion, 37% HCl was added to remove the *iso*propylidene, resulting in the desired final compounds **19a**–**k** with yields ranging from low to good (28–84%), (see SI for NMR spectra), which were evaluated for their antiviral activities.

### 2.2. Antiviral Activity

The antiviral activity of the obtained compounds was evaluated through high-throughput screening (HTS) on various French technology platforms [27]. Phenotypic screenings were conducted using robotic approaches to identify potential antiviral drug candidates, with biological assays miniaturized into 384-well plates and analyzed via high-content fluorescence confocal microscopy. Compounds demonstrating ≥85% antiviral activity and ≤85% cytotoxicity at 10 μM in the first screening were identified as hits, which will then be validated through dose-response analyses and tests on cell lines to determine their IC_50_ and CC_50_.

For the H1N1 SOV NLuc virus, produced in MDCK cells, activity and toxicity of nucleoside analogs at 10 μM were assessed via immunostaining, with favipiravir as a reference. Similarly, RSV mCherry virus, produced in Vero E6 cells, was evaluated using RSV604 as the reference. SARS-CoV-2 (Delta strain) was produced in A549-hACE2 cells, and cells were treated with compounds at 10 μM before infection, with remdesivir used as a reference, while cytopathic effect (CPE) measured inhibition and viability. HCoV-229E (GFP-expressing) was produced in Huh-7 cells, with infected cells quantified using GFP and total cells measured with Hoechst staining; after a 30 min incubation with compounds at 10 μM, fluorescence was analyzed following a 24 h period. Hepatitis E virus (GFP replicon) in PLC/PRF/5 cells and hepatitis C virus (JFH1, gt2a strain) in Huh-7 cells were both analyzed for activity and toxicity at 10 μM using fluorescence and immunostaining, respectively. H3N2 virus in A549-TMPRSS2 cells underwent immunostaining-based evaluation at the same concentration. Zika virus, marked with NanoLuciferase and produced in Vero cells, was assessed by exposing cells to compounds at 10 μM for 2 h before infection, with reductions in NanoLuciferase levels indicating antiviral activity and toxicity measured after 48 h.

No hit was found to significantly reduce the viral replication with ≥85% activity and ≤85% cytotoxicity. The lack of antiviral effects may be due to insufficient cellular activation, weak binding to structurally cellular or viral enzymes involved in the nucleoside metabolism, caused by *C5*-modifications on the uracil moiety.

## 3. Experimental Section

### 3.1. Chemistry

Commercially available chemicals were of reagent grade and used as received. All reactions requiring anhydrous conditions were carried out using oven-dried glassware and under an atmosphere of dry Argon. The reactions were monitored by thin layer chromatography (TLC) analysis using silica gel plates (Kieselgel 60F_254_, E. Merck, Rahway, NJ, USA). Column chromatography was performed on Silica Gel 60 M (0.040–0.063 mm, E. Merck) or by flash chromatography Buchi with FlashPure EcoFlex silica cartridge. The ^1^H and ^13^C NMR spectra were recorded on Bruker Avance DPX 250 or Bruker Avance 400 Spectrometers (Bruker, Champs sur Marne, France) using deuterated solvents as internal standard. Chemical shifts are given in ppm, and multiplicities are reported as s (singlet), d (doublet), t (triplet), q (quartet), bs (broad signal), m (multiplet), and dd (doublet of doublet). Multiplicities were determined by the DEPT 135 sequence. Attributions of protons and carbons were made with the help of HSQC and HMBC 2D NMRs. High-resolution mass spectra were performed on a Bruker Q-TOF MaXis mass spectrometer (Bruker Daltonics, Bremen, Germany) by the “Fédération de Recherche” ICOA/CBM (FR2708) platform. Microwaves-assisted reactions were carried out in a Biotage Initiator microwave synthesis instrument (Biotage Sweden AB, Uppsala, Sweden) and temperatures were measured by IR-sensor. Melting points (mp [°C]) were determined on a Kofler bench apparatus (Servilab, Le Mans, France) and are uncorrected.

#### 3.1.1. 5-Iodo-2′,3′-*O*-isopropylidene-uridine (**11**)

A round-bottom flask was charged with 5-iodouridine (**10**) (5 g, 13.52 mmol, 1 equiv.) and acetone (250 mL), and molecular sieves of 4 Å were added. The reaction mixture was cooled down to 0 °C, and H_2_SO_4_ (1.88 mL, 27.03 mmol, 2 equiv.) was added dropwise. The mixture was stirred for 48 h at room temperature. After the reaction completion, dry NaHCO_3_ was added until pH = 7. Salts and base were removed from reaction mixture by filtration under reduced pressure. The filtrate was concentrated under reduced pressure to obtain the desired compound (**11**) (5.50 g, 99% yield) as a yellow oil. CAS *#:* 19556-58-2. ^1^H NMR (400 MHz, MeOD) δ 8.36 (s, 1H, H_6_), 5.87 (d, *J* = 2.6 Hz, 1H, H_1_′), 4.90 (dd, *J* = 6.4, 2.4 Hz, 1H, H_2_′), 4.83 (dd, *J* = 6.5, 3.1 Hz, 1H, H_3_′), 4.24 (q, *J* = 3.7 Hz, 1H, H_4_′), 3.83–3.67 (m, 2H, H_5_′), 1.54 (s, 3H, CH_3_), 1.35 (s, 3H, CH_3_). ^13^C NMR (101 MHz, MeOD) δ 162.97, 152.32, 148.30, 115.06, 94.20, 88.70, 86.11, 82.18, 68.44, 62.94, 27.51, 25.51.

#### 3.1.2. 5′-*O*-Acetyl-5-iodo-2′,3′-*O*-isopropylidene-uridine (**12**)

To a solution of (**11**) (6.26 g, 15.26 mmol, 1 equiv.) in pyridine (135 mL) was added Ac_2_O (7.08 mL, 76.32 mmol, 5 equiv.). The reaction was stirred at room temperature for 15 h. After the reaction completion, the mixture was cooled down to 0 °C then quenched with methanol (3.06 mL, 76.32 mmol, 5 equiv.) and stirred at room temperature for 3 h. The solvents were removed under reduced pressure using toluene for co-evaporation if necessary. The residue was purified by silica gel column chromatography (CH_2_Cl_2_/MeOH, 95:5), and the desired product (**12**) (6.10 g, 90% yield) was isolated as a yellow oil. CAS #: 161612-73-3 ^1^H NMR (400 MHz, CDCl_3_) δ 8.51 (s, 1H, NH), 7.76 (s, 1H, H_6_), 5.74 (s, 1H, H_1_′), 4.90 (dd, *J* = 6.5, 2.1 Hz, 1H, H_2_′), 4.78 (dd, *J* = 6.9, 3.7 Hz, 1H, H_3_′), 4.40 (q, *J* = 4.4 Hz, 1H, H_4_′), 4.37–4.24 (m, 2H, H_5_′), 2.14 (s, 3H, OAc), 1.58 (s, 3H, CH_3_), 1.36 (s, 3H, CH_3_). ^13^C NMR (101 MHz, CDCl_3_) δ 170.47, 159.67, 149.50, 146.08, 115.00, 94.30, 85.22, 84.91, 80.92, 68.57, 64.10, 27.28, 25.45, 21.11, 0.14. HRMS-ESI (*m*/*z*) [M + H]^+^ calcd for C_14_H_18_IN_2_O_7_ 453.0154, found 453.0153. Melting point: 182–184 °C (deg.).

#### 3.1.3. 5′-*O*-Acetyl-2′,3′-*O*-isopropylidene-5-trimethylsilylethynyl-uridine (**13**)

In a dried microwave tube containing a solution of compound (**12**) (1.11 g, 2.45 mmol, 1 equiv.) in dry DMF (20 mL) under Argon was added ethynyltrimitylsilane (1.04 mL, 7.36 mmol, 3 equiv.) and Et_3_N (1.02 mL, 7.36 mmol, 3 equiv.). The reaction was stirred at room temperature for 15 min, then CuI (93 mg, 0.49 mmol, 0.2 equiv.) and Pd(PPh_3_)_2_Cl_2_ (171 mg, 0.24 mmol, 0.1 equiv.) were added, respectively. The reaction was heated at 60 °C under MW irradiations for 25 min. The solvent was evaporated, and the residue was extracted with EtOAc (3 × 25 mL). The organic layers were washed with brine, dried over MgSO_4_, filtrated, and concentrated under reduced pressure. The resulting crude product was then purified using silica gel flash chromatography (Petroleum Ether (PE)/EtOAc 7/3) to afford the desired compound (**13**) as a brown solid (4.56 g, 90% yield). mp 88–90 °C. ^1^H NMR (400 MHz, CDCl_3_) δ 9.52 (s, 1H, NH), 7.61 (d, *J* = 1.3 Hz, 1H, H_6_), 5.78 (d, *J* = 2.3 Hz, 1H, H_1_′), 4.87 (dd, *J* = 6.4, 2.1 Hz, 1H, H_2_′), 4.78 (dd, *J* = 6.5, 3.8 Hz, 1H, H_3_′), 4.39–4.34 (m, 1H, H_4_′), 4.33–4.23 (m, 2H, H_5_′), 2.11 (s, 3H, OAc), 1.55 (s, 3H, CH_3_), 1.33 (s, 3H, CH_3_), 0.19 (s, 9H, TMS). ^13^C NMR (101 MHz, CDCl_3_) δ 170.46, 161.32, 149.18, 144.47, 114.91, 100.74, 100.11, 94.93, 93.74, 85.00, 84.87, 80.72, 64.04, 27.20, 25.37, 20.97, −0.12. HRMS-ESI (*m*/*z*) [M + H]^+^ calcd for C_19_H_26_N_2_O_7_Si 423.1578, found 423.1582.

#### 3.1.4. 5′-*O*-Acetyl-5-ethynyl-2′,3′-*O*-isopropylidene-uridine (**14**)

To a solution of (**13**) (4.51 g, 10.67 mmol, 1 equiv.) in THF (80 mL) was added TBAF (2.79 mL, 11.21 mmol, 1.05 equiv.). The mixture was stirred at room temperature for 2 h. After reaction completion, the solvent was removed under reduced pressure. The dried resulting solid was retaken in EtOAc and then washed with water (2 × 40 mL) and brine. After that, it was dried over MgSO_4_, filtrated, and concentrated under reduced pressure. The residue was purified by silica gel flash chromatography (PE/EtOAc 6/4). The resulting product (**14**) was obtained as a yellow solid (2.84 g, 76% yield). mp 182–184 °C (dec.) ^1^H NMR (400 MHz, CDCl_3_) δ 7.69 (s, 1H, H_6_), 5.78 (d, *J* = 2.2 Hz, 1H, H_1_′), 4.89 (dd, *J* = 6.4, 2.2 Hz, 1H, H_2_′), 4.79 (dd, *J* = 6.4, 4.0 Hz, 1H, H_3_′), 4.41 (q, *J* = 4.3 Hz, 1H, H_4_′), 4.35–4.25 (m, 2H, H_5_′), 3.19 (s, 1H, C≡C-H), 2.12 (s, 3H, OAc), 1.57 (s, 3H, CH_3_), 1.35 (s, 3H, CH_3_). ^13^C NMR (101 MHz, CDCl_3_) δ 170.48, 161.12, 148.90, 144.99, 115.00, 99.53, 94.13, 85.27, 85.02, 82.60, 80.80, 74.28, 64.08, 27.26, 25.42, 20.95. HRMS-ESI (*m*/*z*) [M + H]^+^ calcd for C_16_H_19_N_2_O_7_ 351.1186, found 351.1187.

#### 3.1.5. General Procedure for Cadiot–Chodkiewicz Cross-Coupling

A solution of NiCl_2_·6H_2_O (55.0 mg, 0.26 mmol, 0.5 equiv.), CuI (82.5 mg, 0.26 mmol, 0.5 equiv.), and TMEDA (0.06 mL, 0.26 mmol, 0.5 equiv.) in THF (10 mL) was stirred under oxygen atmosphere at room temperature for 5 min. Then, alkyne (2.60 mmol, 5 equiv.) was added to the reaction mixture. The starting material (**14**) (300 mg, 0.52 mmol, 1 equiv.) was dissolved in THF (5 mL) and slowly added. The mixture was allowed for 24 h at room temperature then evaporated in vacuo and purified by silica gel column chromatography with a mixture of Petroleum Ether (PE) and EtOAc.

*5′-O-Acetyl-5-[4-[4-(trifluoromethyl)phenyl]buta-1,3-diynyl]-2′,3′-O-isopropylidene-uridine* (**15a**). Yield 45% as a yellow solid. ^1^H NMR (400 MHz, CDCl_3_) δ 9.68 (s, 1H, NH), 7.80 (s, 1H, H_6_), 7.57 (s, 4H, Ar), 5.79 (s, 1H, H_1_′), 4.93 (d, *J* = 6.1 Hz, 1H, H_2_′), 4.80 (dd, *J* = 6.6, 3.7 Hz, 1H, H_3_′), 4.41 (dd, *J* = 8.6, 4.4 Hz, 1H, H_4_′), 4.33 (d, *J* = 5.8 Hz, 2H, H_5_′), 2.13 (s, 3H, OAc), 1.56 (s, 3H, CH_3_), 1.34 (s, 3H, CH_3_), ^13^C NMR (101 MHz, CDCl_3_) δ 170.54, 161.35, 148.94, 146.18, 132.88, 131.58, 131.26, 130.93, 130.61, 125.55, 125.51, 125.48, 125.44, 125.32, 125.15, 122.44, 114.96, 99.26, 94.36, 85.46, 85.04, 81.06, 80.84, 78.31, 75.78, 73.56, 64.09, 27.19, 25.35, 20.86, ^9^F NMR (376 MHz, CDCl_3_) δ −63.02.

*5′-O-Acetyl-5-[4-(4-pentylphenyl)buta-1,3-diynyl]-2′,3′-O-*iso*propylidene-uridine* (**15b**). Yield 47% as a pale yellow powder. ^1^H NMR (400 MHz, CDCl_3_) δ 8.77 (s, 1H, NH), 7.75 (s, 1H, H_6_), 7.40 (d, *J* = 7.3 Hz, 2H, Ar H_2,6_), 7.14 (d, *J* = 7.8 Hz, 2H, Ar H_3,5_), 5.80 (s, 1H, H_1_′), 4.89 (d, *J* = 6.4 Hz, 1H, H_2_′), 4.79 (dd, *J* = 6.5, 4.0 Hz, 1H, H_3_′), 4.41 (t, *J* = 4.2 Hz, 1H, H_4_′), 4.37–4.29 (m, 2H, H_5_′), 2.60 (t, *J* = 7.7 Hz, 2H, CH_2_ 1), 2.16 (s, 3H, OAc), 1.64–1.53 (m, 5H, CH_2_ and CH_3_), 1.36 (s, 3H, CH_3_), 1.34–1.28 (m, 4H, 2CH_2_), 0.88 (t, *J* = 6.6 Hz, 3H, CH_3 aliphatic_). ^13^C NMR (101 MHz, CDCl_3_) δ 170.50, 160.87, 148.73, 145.26, 145.16, 132.66, 128.76, 118.52, 115.05, 99.98, 94.04, 85.33, 85.11, 83.38, 80.74, 79.26, 73.00, 71.60, 64.04, 36.12, 31.55, 30.94, 27.28, 25.43, 22.62, 20.92, 14.11.

*5′-O-Acetyl-5-[4-(3,5-dimethoxyphenyl)buta-1,3-diynyl]-2′,3′-O-*iso*propylidene-uridine* (**15c**). Yield 35% as a pale yellow powder. ^1^H NMR (400 MHz, CDCl_3_) δ 9.02 (s, 1H, NH), 7.76 (s, 1H, H_6_), 6.64 (s, 2H, Ar H_2,6_), 6.49 (h, *J* = 4.1, 2.1 Hz, 1H, Ar H_4_), 5.79 (d, *J* = 2.0 Hz, 1H, H_1_′), 4.91 (d, *J* = 6.2 Hz, 1H, H_2_′), 4.80 (dd, *J* = 6.1, 4.5 Hz, 1H, H_3_′), 4.41 (dd, *J* = 9.3, 4.5 Hz, 1H, H_4_′), 4.36–4.27 (m, 2H, H_5_′), 3.78 (s, 6H, OMe), 2.15 (s, 3H, OAc), 1.58 (s, 3H, CH_3_), 1.36 (s, 3H, CH_3_). ^13^C NMR (101 MHz, CDCl_3_) δ 170.51, 160.97, 160.70, 148.80, 145.55, 122.66, 115.01, 110.39, 103.35, 99.74, 94.19, 85.39, 85.09, 82.94, 80.79, 78.91, 73.09, 72.07, 64.06, 55.61, 27.25, 25.41, 20.91.

*5′-O-Acetyl-5-[4-[4-(trifluoromethoxy)phenyl]buta-1,3-diynyl]-2′,3′-O-*iso*propylidene-uridine* (**15d**). Yield 50% as a yellow solid. ^1^H NMR (400 MHz, CDCl_3_) δ 9.41 (s, 1H, NH), 7.78 (s, 1H, H_6_), 7.52 (d, *J* = 8.6 Hz, 2H, Ar H_3,5_), 7.16 (d, *J* = 8.3 Hz, 2H, Ar H_2,6_), 5.79 (s, 1H, H_1_′), 4.92 (d, *J* = 6.4 Hz, 1H, H_2_′), 4.80 (dd, *J* = 6.5, 3.9 Hz, 1H, H_3_′), 4.41 (dd, *J* = 9.0, 4.6 Hz, 1H, H_4_′), 4.38–4.28 (m, 2H, H_5_′), 2.14 (s, 3H, OAc), 1.57 (s, 3H, CH_3_), 1.35 (s, 3H, CH_3_). ^13^C NMR (101 MHz, CDCl_3_) δ 170.54, 161.24, 149.86, 149.84, 148.89, 145.87, 134.29, 121.71, 121.01, 120.18, 119.14, 114.98, 99.48, 94.30, 85.43, 85.06, 81.25, 80.81, 78.57, 74.38, 72.67, 64.09, 27.22, 25.37, 20.89, 0.10. ^19^F NMR (376 MHz, CDCl_3_) δ −57.75.

*5′-O-Acetyl-5-[4-(3-aminophenyl)buta-1,3-diynyl]-2′,3′-O-*iso*propylidene-uridine* (**15e**). Yield 55% as a dark green solid. ^1^H NMR (400 MHz, MeOD) δ 8.09 (s, 1H, H_6_), 7.07 (t, *J* = 7.8 Hz, 1H, Ar H_6_), 6.85–6.77 (m, 2H, Ar H_2,5_), 6.78–6.70 (m, 1H, Ar H_4_), 5.83 (d, *J* = 2.0 Hz, 1H, H_1_′), 5.05 (dd, *J* = 6.3, 2.0 Hz, 1H, H_2_′), 4.86 (d, *J* = 3.8 Hz, 1H, H_3_′), 4.38 (dd, *J* = 8.8, 4.4 Hz, 1H, H_4_′), 4.31 (d, *J* = 4.6 Hz, 2H, H_5_′), 2.11 (s, 3H, OAc), 1.54 (s, 3H, CH_3_), 1.35 (s, 3H, CH_3_). ^13^C NMR (101 MHz, MeOD) δ 172.25, 164.00, 150.78, 148.38, 130.32, 122.87, 119.31, 117.80, 115.36, 99.59, 95.50, 86.71, 86.11, 83.78, 82.33, 78.63, 73.55, 73.11, 65.17, 27.41, 25.48, 20.72.

*5′-O-Acetyl-5-[4-(4-pyridyl)buta-1,3-diynyl]-2′,3′-O-*iso*propylidene-uridine* (**15f**). Yield 31% as a brown foam. ^1^H NMR (400 MHz, MeOD) δ 8.56 (d, *J* = 5.1 Hz, 2H, Ar H_3,5_), 8.18 (s, 1H, H_6_), 7.50 (d, *J* = 4.5 Hz, 2H, Ar H_2,6_), 5.83 (s, 1H, H_1_′), 5.06 (dd, *J* = 6.3, 2.0 Hz, 1H, H_2_′), 4.88–4.84 (m, 1H, H_3_′), 4.39 (q, *J* = 4.5 Hz, 1H, H_4_′), 4.32 (d, *J* = 4.7 Hz, 2H, H_5_′), 2.10 (s, 3H, OAc), 1.54 (s, 3H, CH_3_), 1.35 (s, 3H, CH_3_). ^13^C NMR (101 MHz, MeOD) δ 172.18, 163.72, 150.70, 150.49, 149.51, 131.96, 127.67, 115.36, 98.68, 95.75, 86.83, 86.13, 82.38, 79.36, 79.05, 77.40, 77.23, 65.18, 27.41, 25.47, 20.71.

*5′-O-Acetyl-5-[4-(3-thienyl)buta-1,3-diynyl]-2′,3′-O-*iso*propylidene-uridine* (**15g**). Yield 67% as a brown oil. ^1^H NMR (400 MHz, CDCl_3_) δ 9.44 (s, 1H, NH), 7.75 (s, 1H, H_6_), 7.60–7.55 (m, 1H, H_a_), 7.29–7.23 (m, 1H, H_c_), 7.13 (d, *J* = 4.9 Hz, 1H, H_d_), 5.79 (s, 1H, H_1_′), 4.91 (dt, *J* = 6.4, 1.5 Hz, 1H, H_2_′), 4.79 (dd, *J* = 6.5, 4.0 Hz, 1H, H_3_′), 4.40 (q, *J* = 4.3 Hz, 1H, H_4_′), 4.32 (t, *J* = 4.5 Hz, 2H, H_5_′), 2.13 (s, 3H, OAc), 1.56 (s, 3H, CH_3_), 1.34 (s, 3H, CH_3_). ^13^C NMR (101 MHz, CDCl_3_) δ 170.57, 161.24, 148.93, 145.57, 131.91, 130.25, 125.82, 120.56, 114.94, 99.70, 94.15, 85.36, 85.04, 80.76, 78.85, 78.06, 73.25, 72.00, 64.07, 50.85, 27.21, 25.37, 20.90.

*5′-O-Acetyl-5-deca-1,3-diynyl-2′,3′-O-*iso*propylidene-uridine* (**15h**). Yield 25% as an orange oil. ^1^H NMR (400 MHz, CDCl_3_) δ 9.14 (s, 1H, NH), 7.69 (s, 1H, H_6_), 5.78 (s, 1H, H_1_′), 4.91–4.86 (m, 1H, H_2_′), 4.78 (dd, *J* = 4.8, 3.2 Hz, 1H, H_3_′), 4.39 (q, *J* = 4.2 Hz, 1H, H_4_′), 4.36–4.26 (m, 2H, H_5_′), 2.31 (t, *J* = 7.0 Hz, 2H, CH_2a_), 2.13 (s, 3H, OAc), 1.57 (s, 3H, CH_3_), 1.52 (q, *J* = 7.2 Hz, 2H, CH_2_), 1.39 (d, *J* = 7.2 Hz, 2H, CH_2_), 1.35 (s, 3H, CH_3_), 1.32–1.24 (m, 4H, 2CH_2_), 0.88 (t, *J* = 6.5 Hz, 3H, CH_3 aliphatic_). ^13^C NMR (101 MHz, CDCl_3_) δ 170.53, 161.29, 148.91, 145.27, 114.97, 100.04, 93.93, 86.44, 85.26, 85.03, 80.71, 79.54, 65.08, 64.84, 64.05, 31.37, 28.63, 28.18, 27.24, 25.40, 22.61, 20.89, 19.66, 14.15, 0.11.

*5′-O-Acetyl-5-[4-(2-pyridyl)buta-1,3-diynyl]-2′,3′-O-*iso*propylidene-uridine* (**15i**). Yield 37% as a black foam. ^1^H NMR (400 MHz, CDCl_3_) δ 9.92 (s, 1H, NH), 8.60 (d, *J* = 4.9 Hz, 1H, Ar H_2_), 7.86 (s, 1H, H_6_), 7.68 (t, *J* = 7.7 Hz, 1H, Ar H_5_), 7.52 (d, *J* = 7.9 Hz, 1H, Ar H_6_), 7.32–7.26 (m, 1H, Ar H_4_), 5.83 (d, *J* = 1.8 Hz, 1H, H_1_′), 4.97 (dd, *J* = 7.2, 2.2 Hz, 1H, H_2_′), 4.88–4.79 (m, 1H, H_3_′), 4.48–4.39 (m, 1H, H_4_′), 4.34 (d, *J* = 5.1 Hz, 2H, H_5_′), 2.15 (s, 3H, OAc), 1.57 (s, 3H, CH_3_), 1.35 (s, 3H, CH_3_). ^13^C NMR (101 MHz, CDCl_3_) δ 170.54, 161.26, 150.32, 148.98, 146.48, 141.89, 136.33, 128.46, 123.79, 114.82, 99.10, 94.17, 85.38, 84.98, 81.22, 80.75, 78.19, 73.43, 73.35, 64.01, 27.15, 25.32, 20.87.

*5′-O-Acetyl-5-(4-cyclopropylbuta-1,3-diynyl)-2′,3′-O-*iso*propylidene-uridine* (**15j**). Yield 40% as a yellow solid. ^1^H NMR (400 MHz, CDCl_3_) δ 8.47 (s, 1H, NH), 7.67 (s, 1H, H_6_), 5.77 (d, *J* = 2.3 Hz, 1H, H_1_′), 4.86 (dd, *J* = 7.0, 2.5 Hz, 1H, H_2_′), 4.77 (dd, *J* = 7.0, 3.5 Hz, 1H, H_3_′), 4.40 (p, *J* = 4.5, 3.9 Hz, 1H, H_4_′), 4.37–4.26 (m, 2H, H_5_′), 2.13 (s, 3H, OAc), 1.57 (s, 3H, CH_3_), 1.35 (s, 3H, CH_3_), 0.91–0.79 (m, 5H, cyclopropyl). ^13^C NMR (101 MHz, CDCl_3_) δ 170.49, 160.87, 148.68, 145.09, 115.03, 100.10, 93.93, 89.50, 85.25, 85.08, 80.72, 79.88, 64.41, 64.03, 27.28, 25.43, 20.91, 9.28.

*5′-O-Acetyl-5-(4-phenylbuta-1,3-diynyl)-2′,3′-O-*iso*propylidene-uridine* (**15k**). Yield 66% as an orange oil. ^1^H NMR (400 MHz, CDCl_3_) δ 9.09 (s, 1H, NH), 7.76 (s, 1H, H_6_), 7.49 (d, *J* = 7.2 Hz, 2H, Ar H_2,6_), 7.41–7.28 (m, 3H, Ar H_3,4,5_), 5.80 (s, 1H, H_1_′), 4.91 (d, *J* = 6.0 Hz, 1H, H_2_′), 4.80 (dd, *J* = 6.8, 3.5 Hz, 1H, H_3_′), 4.41 (d, *J* = 4.4 Hz, 1H, H_4_′), 4.38–4.28 (m, 2H, H_5_′), 2.15 (s, 3H, OAc), 1.58 (s, 3H, CH_3_), 1.36 (s, 3H, CH_3_). ^13^C NMR (101 MHz, CDCl_3_) δ 170.52, 161.03, 148.83, 145.53, 132.69, 129.62, 128.60, 121.46, 115.01, 99.80, 94.13, 85.37, 85.08, 82.91, 80.76, 78.98, 73.58, 72.04, 64.06, 27.25, 25.41, 20.92.

#### 3.1.6. General Procedure for Ribonucleosides **16a**–**k**

A solution of compounds **15a**–**k** (0.11 mmol, 1 equiv.), respectively, dissolved in MeOH (4 mL) with HCl (37%) (0.20 mL, 2.31 mmol, 21 equiv.) was stirred at room temperature for 24 h. After reaction completion, the solvent was removed under reduced pressure and the residue was diluted in EtOAc (25 mL) and neutralized using saturated NaHCO_3_. The mixture was extracted with EtOAc (3 × 15 mL). The organic layers were washed with brine, dried over MgSO_4_, filtrated, and concentrated under reduced pressure. The resulting crude product was then purified using silica gel flash chromatography (CH_2_Cl_2_/MeOH 95/5) to afford the desired compound.

*5-[4-[4-(Trifluoromethyl)phenyl]buta-1,3-diynyl]-uridine* (**16a**). Yield 71% as yellow powder. mp 214–216 °C (dec.). ^1^H NMR (400 MHz, MeOD) δ 8.65 (s, 1H, H_6_), 7.68 (d, *J* = 1.5 Hz, 4H, Ar), 5.89 (t, *J* = 1.3 Hz, 1H, H_1_′), 4.20 (d, *J* = 3.4 Hz, 2H, H_2_′_,3_′), 4.04 (s, 1H, H_4_′), 3.92 (dd, *J* = 12.4, 2.5 Hz, 1H, H_5_′), 3.77 (dd, *J* = 12.3, 2.5 Hz, 1H, H_5_′). ^13^C NMR (101 MHz, MeOD) δ 163.97, 151.12, 147.98, 133.97, 132.03, 131.70, 126.96, 126.64, 126.61, 126.57, 126.53, 123.93, 98.82, 91.39, 86.30, 80.83, 77.51, 76.79, 76.25, 76.04, 70.67, 61.57. ^19^F NMR (376 MHz, MeOD) δ −64.50. HRMS-ESI (*m*/*z*) [M + H]^+^ calcd for C_20_H_16_F_3_N_2_O_6_ 437.0953, found 437.0955.

*5-[4-(4-Pentylphenyl)buta-1,3-diynyl]-uridine* (**16b**). Yield 69% as dark yellow powder. mp 116–118 °C. ^1^H NMR (400 MHz, MeOD) δ 8.58 (s, 1H, H_6_), 7.38 (d, *J* = 7.5 Hz, 2H, Ar H_2,6_), 7.17 (d, *J* = 7.4 Hz, 2H, Ar H_3,5_), 5.88 (s, 1H, H_1_′), 4.18 (s, 2H, H_2_′_,3_′), 4.03 (s, 1H, H_4_′), 3.90 (d, *J* = 11.9 Hz, 1H, H_5_′), 3.75 (d, *J* = 11.8 Hz, 1H, H_5_′), 2.60 (t, *J* = 7.6 Hz, 2H, CH_2_), 1.59 (p, *J* = 7.4 Hz, 2H, CH_2_), 1.37–1.22 (m, 4H, 2CH_2_), 0.88 (t, *J* = 6.7 Hz, 3H, CH_3 aliphatic_). ^13^C NMR (101 MHz, MeOD) δ 164.10, 151.14, 147.28, 146.15, 133.34, 129.76, 119.82, 99.38, 91.23, 86.30, 82.87, 78.21, 76.16, 73.96, 73.83, 70.71, 61.61, 36.79, 32.47, 32.00, 23.47, 14.28. HRMS-ESI (*m*/*z*) [M + H]^+^ calcd for C_24_H_27_N_2_O_6_ 439.1880, found 439.1864.

*5-[4-(3,5-Dimethoxyphenyl)buta-1,3-diynyl]-uridine* (**16c**). Yield 52% as clear powder. mp 254–256 °C (dec.). ^1^H NMR (400 MHz, MeOD) δ 8.62 (s, 1H, H_6_), 6.64 (d, *J* = 1.9 Hz, 2H, Ar H_2,6_), 6.55 (t, *J* = 2.5 Hz, 1H, Ar H_4_), 5.89 (s, 1H, H_1_′), 4.19 (d, *J* = 3.3 Hz, 2H, H_2_′_,3_′), 4.04 (s, 1H, H_4_′), 3.91 (d, *J* = 12.7 Hz, 1H, H_5_′), 3.81 (d, *J* = 5.1 Hz, 1H, H_5_′), 3.78 (s, 6H, OMe). ^13^C NMR (101 MHz, MeOD) δ 162.30, 147.46, 143.09, 132.78, 123.97, 111.47, 104.92, 95.81, 86.02, 78.42, 70.59, 67.43, 62.53, 55.96. HRMS-ESI (*m*/*z*) [M + H]^+^ calcd for C_21_H_21_N_2_O_8_ 429.1294, found 429.1292.

*5-[4-[4-(Trifluoromethoxy)phenyl]buta-1,3-diynyl]-uridine* (**16d**). Yield 87% as clear powder. mp 198–200 °C (dec.). ^1^H NMR (400 MHz, MeOD) δ 8.63 (s, 1H, H_6_), 7.60 (d, *J* = 8.8 Hz, 2H, Ar H_3,5_), 7.29 (d, *J* = 8.3 Hz, 2H, Ar H_2,6_), 5.89 (d, *J* = 2.7 Hz, 1H, H_1_′), 4.19 (d, *J* = 3.4 Hz, 2H, H_2_′_,3_′), 4.04 (p, *J* = 2.7 Hz, 1H, H_4_′), 3.92 (dd, *J* = 12.3, 2.5 Hz, 1H, H_5_′), 3.77 (dd, *J* = 12.2, 2.6 Hz, 1H, H_5_′). ^13^C NMR (101 MHz, MeOD) δ 164.02, 151.14, 150.92, 147.75, 135.34, 122.27, 121.93, 98.99, 91.34, 86.32, 80.93, 77.71, 76.24, 75.37, 75.15, 70.71, 61.60. ^19^F NMR (376 MHz, MeOD) δ −59.42. HRMS-ESI (*m*/*z*) [M + H]^+^ calcd for C_20_H_16_F_3_N_2_O_7_ 453.0903, found 453.0904.

*5-[4-(3-Aminophenyl)buta-1,3-diynyl]-uridine* (**16e**). Yield 76% as yellow powder. mp 178–180 °C (dec.). ^1^H NMR (400 MHz, MeOD) δ 8.59 (s, 1H, H_6_), 7.07 (t, *J* = 7.8 Hz, 1H, Ar H_3_), 6.84–6.70 (m, 3H, Ar H_4,5,6_), 5.90 (d, *J* = 2.8 Hz, 1H, H_1_′), 4.19 (d, *J* = 3.7 Hz, 2H, H_2_′_,3_′), 4.04 (p, *J* = 2.5 Hz, 1H, H_4_′), 3.91 (dd, *J* = 12.2, 2.5 Hz, 1H, H_5_′), 3.77 (dd, *J* = 12.2, 2.6 Hz, 1H, H_5_′). ^13^C NMR (101 MHz, MeOD) δ 164.13, 151.21, 149.33, 147.31, 130.30, 123.01, 122.82, 119.27, 117.74, 99.42, 91.24, 86.36, 83.49, 78.31, 76.21, 73.77, 73.31, 70.80, 61.66. HRMS-ESI (*m*/*z*) [M + H]^+^ calcd for C_19_H_18_N_3_O_6_ 384.1190, found 384.1190.

*5-[4-(4-Pyridyl)buta-1,3-diynyl]-uridine* (**16f**). Yield 97% as black powder. mp 248–250 °C (dec.). ^1^H NMR (400 MHz, DMSO) δ 8.66 (s, 1H, H_6_), 8.63 (s, 2H, Ar H_3,5_), 7.55 (s, 2H, Ar H_2.6_), 5.72 (d, *J* = 3.8 Hz, 1H, H_1_′), 5.53 (d, *J* = 5.0 Hz, 1H, OH_2_′), 5.38 (t, *J* = 4.9 Hz, 1H, OH_5_′), 5.18 (d, *J* = 5.4 Hz, 1H, OH_3_′), 4.10–3.94 (m, 2H, H_2_′_,3_′), 3.91–3.86 (m, 1H, H_4_’), 3.70 (dd, *J* = 8.6, 3.8 Hz, 1H, H_5_′), 3.60 (dd, *J* = 7.8, 3.6 Hz, 1H, H_5_’). ^13^C NMR (101 MHz, DMSO) δ 161.44, 157.06, 150.17, 149.38, 147.23, 126.00, 102.76, 96.14, 89.05, 84.74, 79.80, 79.31, 77.58, 77.31, 74.19, 68.95, 59.89. HRMS-ESI (*m*/*z*) [M + H]^+^ calcd for C_18_H_16_N_3_O_6_ 370.1030, found 370.1034.

*5-[4-(3-Thienyl)buta-1,3-diynyl]-uridine* (**16g**). Yield 61% as yellow powder. mp 270–272 °C (dec.). ^1^H NMR (400 MHz, MeOD) δ 8.60 (s, 1H, H_6_), 7.73 (t, *J* = 1.7 Hz, 1H, H_a_), 7.45 (dd, *J* = 5.2, 3.0 Hz, 1H, H_c_), 7.16 (d, *J* = 5.0 Hz, 1H, H_d_), 5.89 (d, *J* = 2.7 Hz, 1H, H_1_′), 4.19 (d, *J* = 3.4 Hz, 2H, H_2_′_,3_′), 4.04 (d, *J* = 2.8 Hz, 1H, H_4_′), 3.91 (dd, *J* = 12.1, 2.5 Hz, 1H, H_5_′), 3.76 (dd, *J* = 12.2, 2.5 Hz, 1H, H_5_′). ^13^C NMR (101 MHz, MeOD) δ 164.28, 151.37, 149.71, 147.42, 139.97, 132.81, 130.98, 127.26, 121.78, 99.29, 91.28, 86.34, 78.11, 78.05, 76.22, 74.40, 73.90, 70.77, 61.64. HRMS-ESI (*m*/*z*) [M + H]^+^ calcd for C_17_H_15_N_2_O_6_S 375.0649, found 375.0645.

*5-Deca-1,3-diynyl-uridine* (**16h**). Yield 46% as orange powder. mp 116–118 °C. ^1^H NMR (400 MHz, MeOD) δ 8.48 (s, 1H, H_6_), 5.87 (d, *J* = 2.8 Hz, 1H, H_1_′), 4.17 (d, *J* = 3.4 Hz, 2H, H_2_′_,3_′), 4.03 (d, *J* = 3.7 Hz, 1H, H_4_′), 3.89 (dd, *J* = 12.3, 2.5 Hz, 1H, H_5_′), 3.75 (dd, *J* = 12.1, 2.6 Hz, 1H, H_5_′), 2.34 (t, *J* = 6.9 Hz, 2H, CH_2_), 1.53 (p, *J* = 7.1 Hz, 2H, CH_2_), 1.41 (p, *J* = 7.0, 6.4 Hz, 2H, CH_2_), 1.34–1.29 (m, 4H, 2 CH_2_), 0.91 (t, *J* = 6.8 Hz, 3H, CH_3_). ^13^C NMR (101 MHz, MeOD) δ 164.22, 151.32, 146.85, 91.07, 86.20, 85.84, 79.41, 79.09, 78.76, 76.04, 70.70, 65.85, 61.62, 32.33, 29.49, 29.23, 23.49, 19.99, 14.36. HRMS-ESI (*m*/*z*) [M + H]^+^ calcd for C_19_H_15_N_2_O_6_ 377.1709, found 377.1707.

*5-[4-(2-Pyridyl)buta-1,3-diynyl]-uridine* (**16i**). Yield 51% as black powder. mp 250–252 °C (dec.). ^1^H NMR (400 MHz, MeOD) δ 8.70 (s, 1H, H_6_), 8.54 (d, *J* = 4.2 Hz, 1H, Ar H_3_), 7.87 (dd, *J* = 15.5, 7.7 Hz, 1H, Ar H_6_), 7.63 (d, *J* = 7.9 Hz, 1H, Ar H_5_), 7.45 (dd, *J* = 7.6, 5.0 Hz, 1H, Ar H_4_), 5.89 (d, *J* = 2.7 Hz, 1H, H_1_′), 4.19 (d, *J* = 3.4 Hz, 2H, H_2_′_,3_′), 4.07–4.02 (m, 1H, H_4_′), 3.92 (dd, *J* = 12.3, 2.5 Hz, 1H, H_5_′), 3.77 (dd, *J* = 12.3, 2.5 Hz, 1H, H_5_′). ^13^C NMR (101 MHz, MeOD) δ 163.89, 151.04, 148.33, 138.65, 129.74, 125.53, 98.58, 91.38, 86.33, 80.75, 77.27, 76.25, 76.21, 74.74, 73.51, 70.66, 61.56, 59.60. HRMS-ESI (*m*/*z*) [M + H]^+^ calcd for C_18_H_16_N_3_O_6_ 370.1032, found 370.1034.

*5-(4-Cyclopropylbuta-1,3-diynyl)-uridine* (**16j**). Yield 88% as white powder. mp 108–110 °C (dec.). ^1^H NMR (400 MHz, MeOD) δ 8.48 (s, 1H, H_6_), 5.87 (d, *J* = 2.9 Hz, 1H, H_1_′), 4.17 (d, *J* = 3.3 Hz, 2H, H_2_′_,3_′), 4.02 (d, *J* = 3.6 Hz, 1H, H_4_′), 3.89 (dd, *J* = 12.1, 2.6 Hz, 1H, H_5_′), 3.75 (dd, *J* = 12.3, 2.7 Hz, 1H, H_5_′), 1.47–1.37 (m, 1H, CH_cyclopropyl_), 0.93–0.86 (m, 2H, CH_2_), 0.77–0.70 (m, 2H, CH_2_). ^13^C NMR (101 MHz, MeOD) δ 164.30, 151.22, 147.02, 99.66, 91.17, 89.07, 86.33, 79.14, 76.13, 70.84, 66.55, 61.71, 61.05, 9.42, 0.74. HRMS-ESI (*m*/*z*) [M + H]^+^ calcd for C_16_H_17_N_2_O_6_ 331.1079, found 331.1081.

*5-(4-Phenylbuta-1,3-diynyl)-uridine* (**16k**). Yield 62% as yellow powder. mp 184–186 °C (dec.). ^1^H NMR (400 MHz, MeOD) δ 8.62 (s, 1H, H_6_), 7.49 (d, *J* = 8.1 Hz, 2H, Ar H_2,6_), 7.44–7.38 (m, 2H, Ar H_3,5_), 7.36 (d, *J* = 6.5 Hz, 1H, Ar H_4_), 5.89 (d, *J* = 2.7 Hz, 1H, H_1_′), 4.19 (d, *J* = 2.5 Hz, 2H, H_2_′_,3_′), 4.06–4.02 (m, 1H, H_4_′), 3.91 (dd, *J* = 12.2, 2.5 Hz, 1H, H_5_′), 3.77 (dd, *J* = 12.3, 2.7 Hz, 1H, H_5_′). ^13^C NMR (101 MHz, MeOD) δ 164.08, 151.18, 147.50, 133.40, 130.58, 129.72, 122.79, 99.24, 91.30, 86.33, 82.56, 78.04, 76.23, 74.42, 70.76, 61.63. HRMS-ESI (*m*/*z*) [M + H]^+^ calcd for C_19_H_17_N_2_O_6_ 369.1085, found 369.1081.

#### 3.1.7. General Procedure for Acetyl Deprotection

Compounds **15a**–**k** (0.24 mmol, 1 equiv.) were respectively dissolved in an excess of NH_3_/MeOH (7 N) (3.5 mL). The reaction mixture was stirred at room temperature for 24 h, and then volatiles were removed under reduced pressure. The residue was purified by silica gel column chromatography (CH_2_Cl_2_/MeOH 95/5) to give compounds **17a**–**k**, respectively.

*5-[4-[4-(Trifluoromethyl)phenyl]buta-1,3-diynyl]-2′,3′-O-*iso*propylidene-uridine* (**17a**). Yield 80% as clear powder. ^1^H NMR (400 MHz, MeOD) δ 8.41 (s, 1H, H_6_), 7.68 (s, 4H, Ar), 5.90 (d, *J* = 2.6 Hz, 1H, H_1_′), 4.92 (dd, *J* = 6.4, 2.4 Hz, 1H, H_2_′), 4.84 (m, 1H, H_3_′), 4.28 (q, *J* = 3.7 Hz, 1H, H_4_′), 3.83 (dd, *J* = 12.0, 3.2 Hz, 1H, H_5_′), 3.74 (dd, *J* = 12.0, 4.2 Hz, 1H, H_5_′), 1.54 (s, 3H, CH_3_), 1.35 (s, 3H, CH_3_). ^13^C NMR (101 MHz, MeOD) δ 164.00, 150.90, 148.87, 134.01, 132.05, 131.73, 126.94, 126.61, 126.57, 126.53, 123.93, 115.02, 98.78, 94.59, 88.97, 86.40, 82.17, 80.90, 77.61, 76.74, 75.97, 62.90, 27.49, 25.49. ^19^F NMR (376 MHz, MeOD) δ −64.50.

*5-[4-(4-Pentylphenyl)buta-1,3-diynyl]-2′,3′-O-*iso*propylidene-uridine* (**17b**). Yield 71% as dark powder. ^1^H NMR (400 MHz, MeOD) δ 8.35 (s, 1H, H_6_), 7.40 (d, *J* = 7.8 Hz, 2H, Ar H_2,6_), 7.19 (d, *J* = 7.8 Hz, 2H, Ar H_3,5_), 5.90 (d, *J* = 2.6 Hz, 1H, H_1_′), 4.91 (dd, *J* = 6.4, 2.6 Hz, 1H, H_2_′), 4.87–4.85 (m, 1H, H_3_′), 4.27 (q, *J* = 3.6 Hz, 1H, H_4_′), 3.82 (dd, *J* = 11.9, 3.3 Hz, 1H, H_5_′), 3.74 (dd, *J* = 12.0, 4.2 Hz, 1H, H_5_′), 2.62 (t, *J* = 7.7 Hz, 2H, CH_2_), 1.62 (p, *J* = 7.4 Hz, 2H, CH_2_), 1.55 (s, 3H, CH_3_), 1.35 (s, 3H, CH_3_), 1.34–1.25 (m, 4H, 2CH_2_), 0.90 (t, *J* = 6.9 Hz, 3H, CH_3 aliphatic_). ^13^C NMR (101 MHz, MeOD) δ 164.15, 150.98, 148.20, 146.24, 133.43, 129.81, 119.90, 115.05, 99.40, 94.50, 88.89, 86.33, 83.01, 82.18, 78.39, 73.98, 73.87, 62.93, 36.84, 32.53, 32.04, 27.51, 25.52, 23.51, 14.32.

*5-[4-(3,5-Dimethoxyphenyl)buta-1,3-diynyl]-2′,3′-O-*iso*propylidene-uridine* (**17c**). Yield 75% as orange powder. ^1^H NMR (400 MHz, MeOD) δ 8.37 (s, 1H, H_6_), 6.65 (d, *J* = 2.3 Hz, 2H, Ar H_2,6_), 6.54 (t, *J* = 2.3 Hz, 1H, Ar H_4_), 5.90 (d, *J* = 2.6 Hz, 1H, H_1_′), 4.91 (dd, *J* = 6.3, 2.6 Hz, 1H, H_2_′), 4.28 (q, *J* = 3.6 Hz, 1H, H_3_′), 3.82 (dd, *J* = 11.9, 3.3 Hz, 1H, H_4_′), 3.78 (s, 6H, OMe), 3.77–3.73 (m, 2H, H_5_′), 1.54 (s, 3H, CH_3_), 1.35 (s, 3H, CH_3_). ^13^C NMR (101 MHz, MeOD) δ 162.28, 150.94, 148.42, 123.97, 115.03, 111.12, 103.74, 99.17, 94.55, 88.93, 86.36, 82.72, 82.18, 78.12, 74.33, 73.84, 62.92, 55.97, 27.50, 25.50.

*5-[4-[4-(Trifluoromethoxy)phenyl]buta-1,3-diynyl]-2′,3′-O-*iso*propylidene-uridine* (**17d**). Yield > 99% as dark orange powder. ^1^H NMR (400 MHz, DMSO) δ 11.82 (s, 1H, NH), 8.43 (s, 1H, H_6_), 7.74 (d, *J* = 7.4 Hz, 2H, Ar H_3,5_), 7.44 (d, *J* = 8.3 Hz, 2H, Ar H_2,6_), 5.84 (d, *J* = 2.3 Hz, 1H, H_1_′), 5.22 (d, *J* = 5.0 Hz, 1H, OH), 4.94 (dd, *J* = 6.2, 2.3 Hz, 1H, H_2_′), 4.76 (dd, *J* = 6.4, 3.4 Hz, 1H, H_3_′), 4.14 (q, *J* = 4.2 Hz, 1H, H_4_′), 3.70–3.54 (m, 2H, H_5_′), 1.49 (s, 3H, CH_3_), 1.29 (s, 3H, CH_3_). ^13^C NMR (101 MHz, DMSO) δ 161.59, 149.21, 147.53, 141.03, 134.50, 121.46, 121.17, 119.84, 118.76, 112.80, 96.46, 91.75, 87.23, 84.18, 80.15, 76.07, 76.06, 74.52, 61.02, 26.93, 25.11. ^19^F NMR (376 MHz, DMSO) δ −56.72.

*5-[4-(3-Aminophenyl)buta-1,3-diynyl]-2′,3′-O-*iso*propylidene-uridine* (**17e**). Yield 71% as dark powder. ^1^H NMR (400 MHz, MeOD) δ 8.35 (s, 1H, H_6_), 7.07 (t, *J* = 7.8 Hz, 1H, Ar H_6_), 6.84–6.77 (m, 2H, Ar H_2,5_), 6.74 (dd, *J* = 8.1, 2.3 Hz, 1H, Ar H_4_), 5.90 (d, *J* = 2.5 Hz, 1H, H_1_′), 4.91 (dd, *J* = 6.4, 2.5 Hz, 1H, H_2_′), 4.87–4.84 (m, 1H, H_3_′), 4.27 (q, *J* = 3.5 Hz, 1H, H_4_′), 3.82 (dd, *J* = 11.9, 3.2 Hz, 1H, H_5_′), 3.74 (dd, *J* = 12.0, 4.2 Hz, 1H, H_5_′), 1.55 (s, 3H, CH_3_), 1.35 (s, 3H, CH_3_). ^13^C NMR (101 MHz, MeOD) δ 164.15, 150.96, 149.35, 148.19, 130.30, 123.00, 122.83, 119.28, 117.73, 115.06, 99.41, 94.50, 88.89, 86.32, 83.55, 82.17, 78.42, 73.71, 73.29, 62.92, 27.50, 25.51.

*5-[4-(4-Pyridyl)buta-1,3-diynyl]-2′,3′-O-*iso*propylidene-uridine* (**17f**). Yield 75% as dark orange powder. ^1^H NMR (400 MHz, DMSO) δ 8.64 (d, *J* = 5.1 Hz, 2H, Ar H_3,5_), 8.47 (s, 1H, H_6_), 7.55 (d, *J* = 5.1 Hz, 2H, Ar H_2,6_), 5.83 (d, *J* = 2.3 Hz, 1H, H_1_′), 5.23 (t, *J* = 5.1 Hz, 1H, OH), 4.94 (dd, *J* = 6.2, 2.3 Hz, 1H, H_2_′), 4.76 (dd, *J* = 6.3, 3.4 Hz, 1H, H_3_′), 4.16 (q, *J* = 4.0 Hz, 1H, H_4_′), 3.69–3.53 (m, 2H, H_5_′), 1.48 (s, 3H, CH_3_), 1.29 (s, 3H, CH_3_). ^13^C NMR (101 MHz, DMSO) δ 161.42, 150.01, 149.11, 148.05, 128.56, 125.87, 112.81, 96.10, 91.86, 87.32, 84.24, 80.15, 78.83, 77.73, 77.52, 75.68, 61.01, 26.94, 25.12.

*5-[4-(3-Thienyl)buta-1,3-diynyl]-2′,3′-O-*iso*propylidene-uridine* (**17g**). Yield 89% as dark orange powder. ^1^H NMR (400 MHz, MeOD) δ 8.35 (s, 1H, H_6_), 7.74 (d, *J* = 3.0 Hz, 1H, Ar H_a_), 7.47–7.43 (m, 1H, Ar H_c_), 7.17 (d, *J* = 5.1 Hz, 1H, H_d_), 5.90 (d, *J* = 2.5 Hz, 1H, H_1_′), 4.91 (dd, *J* = 6.3, 2.5 Hz, 1H, H_2_′), 4.87–4.84 (m, 1H, H_3_′), 4.27 (q, *J* = 3.7 Hz, 1H, H_4_′), 3.82 (dd, *J* = 12.0, 3.2 Hz, 1H, H_5_′), 3.74 (dd, *J* = 11.9, 4.2 Hz, 1H, H_5_′), 1.55 (s, 3H, CH_3_), 1.35 (s, 3H, CH_3_). ^13^C NMR (101 MHz, MeOD) δ 148.29, 132.85, 130.99, 127.23, 121.75, 115.05, 99.30, 94.51, 88.90, 86.33, 82.17, 78.24, 77.99, 74.04, 73.92, 62.92, 27.50, 25.51.

*5-Deca-1,3-diynyl-2′,3′-O-*iso*propylidene-uridine* (**17h**). Yield 73% as clear powder. ^1^H NMR (400 MHz, MeOD) δ 8.27 (s, 1H, H_6_), 5.88 (d, *J* = 2.6 Hz, 1H, H_1_′), 4.89 (dd, *J* = 6.5, 2.4 Hz, 2H, H_2_′_,3_′), 4.25 (q, *J* = 3.6 Hz, 1H, H_4_′), 3.80 (dd, *J* = 12.0, 3.3 Hz, 1H, H_5_′), 3.72 (dd, *J* = 12.0, 4.3 Hz, 1H, H_5_′), 2.36 (t, *J* = 6.9 Hz, 2H, CH_2_), 1.59–1.40 (m, 7H, CH_3_, 2CH_2_), 1.33 (d, *J* = 9.2 Hz, 7H, CH_3_, 2CH_2_), 0.92 (t, *J* = 6.7 Hz, 3H, CH_3 aliphatic_). ^13^C NMR (101 MHz, MeOD) δ 164.34, 150.98, 147.96, 115.08, 99.62, 94.38, 88.80, 86.24, 85.94, 82.16, 78.97, 67.13, 65.89, 62.91, 32.42, 29.58, 29.33, 27.50, 25.51, 23.59, 20.01, 14.35.

*5-[4-(2-Pyridyl)buta-1,3-diynyl]-2′,3′-O-*iso*propylidene-uridine* (**17i**). Yield 70% as dark orange powder. ^1^H NMR (400 MHz, MeOD) δ 8.54 (d, *J* = 5.0 Hz, 1H, Ar H_3_), 8.44 (s, 1H, H_6_), 7.86 (t, *J* = 7.8 Hz, 1H, Ar H_5_), 7.63 (d, *J* = 7.7 Hz, 1H, Ar H_6_), 7.44 (dd, *J* = 7.7, 5.0 Hz, 1H, Ar H_4_), 5.90 (d, *J* = 2.6 Hz, 1H, H_1_′), 4.92 (dd, *J* = 6.4, 2.5 Hz, 1H, H_2_′), 4.86 (d, *J* = 3.2 Hz, 1H, H_3_′), 4.29 (q, *J* = 3.5 Hz, 1H, H_4_′), 3.83 (dd, *J* = 12.0, 3.2 Hz, 1H, H_5_′), 3.74 (dd, *J* = 12.0, 4.1 Hz, 1H, H_5_′), 1.55 (s, 3H, CH_3_), 1.35 (s, 3H, CH_3_). ^13^C NMR (101 MHz, MeOD) δ 163.89, 151.05, 149.19, 142.69, 138.60, 133.03, 129.76, 125.52, 115.03, 98.55, 94.62, 89.00, 86.41, 82.18, 80.80, 77.38, 76.16, 74.70, 62.90, 27.49, 25.51.

*5-(4-Cyclopropylbuta-1,3-diynyl)-2′,3′-O-*iso*propylidene-uridine* (**17j**). Yield 81% as clear powder. ^1^H NMR (400 MHz, MeOD) δ 8.26 (s, 1H, H_6_), 5.88 (d, *J* = 2.6 Hz, 1H, H_1_′), 4.89 (dd, *J* = 6.5, 2.3 Hz, 1H, H_2_′), 4.83–4.80 (m, 1H, H_3_′), 4.25 (q, *J* = 3.7 Hz, 1H, H_4_′), 3.80 (dd, *J* = 11.9, 3.3 Hz, 1H, H_5_′), 3.72 (dd, *J* = 11.9, 4.2 Hz, 1H, H_5_′), 1.54 (s, 3H, CH_3_), 1.42 (tt, *J* = 8.7, 5.1 Hz, 1H, CH), 1.34 (s, 3H, CH_3_), 0.89 (dd, *J* = 8.0, 3.0 Hz, 2H, CH_2_), 0.77–0.71 (m, 2H, CH_2_). ^13^C NMR (101 MHz, MeOD) δ 164.32, 150.95, 147.97, 115.05, 99.62, 94.37, 89.12, 88.79, 86.24, 82.15, 79.23, 66.48, 62.91, 61.02, 27.50, 25.50, 9.44.

*5-(4-Phenylbuta-1,3-diynyl)-2′,3′-O-*iso*propylidene-uridine* (**17k**). Yield 82% as clear yellow powder. ^1^H NMR (400 MHz, MeOD) δ 8.37 (s, 1H, H_6_), 7.53–7.47 (m, 2H, Ar H_2,6_), 7.44–7.33 (m, 3H, Ar H_3,4,5_), 5.90 (d, *J* = 2.1 Hz, 1H, H_1_′), 4.91 (dd, *J* = 6.4, 2.5 Hz, 1H, H_2_′), 4.85 (d, *J* = 3.3 Hz, 1H, H_3_′), 4.27 (q, *J* = 3.5 Hz, 1H, H_4_′), 3.82 (dd, *J* = 11.9, 3.2 Hz, 1H, H_5_′), 3.74 (dd, *J* = 12.0, 4.2 Hz, 1H, H_5_′), 1.55 (s, 3H, CH_3_), 1.35 (s, 3H, CH_3_). ^13^C NMR (101 MHz, MeOD) δ 164.10, 150.95, 148.37, 133.42, 130.59, 129.71, 122.77, 115.05, 99.24, 94.53, 88.92, 86.34, 82.63, 82.18, 78.15, 74.39, 74.35, 62.92, 27.50, 25.51.

#### 3.1.8. General Procedure for Ribonucleoside Phosphoramidate Pronucleotides **9a**–**k**

In a round-bottom flask (dry and flashed), compounds **17a**–**k** (0.14 mmol, 1 equiv.) were respectively dissolved in THF (6 mL) under inert atmosphere. The reaction mixture was cooled down to 0 °C, then *t*-BuMgCl (0.25 mL, 0.42 mmol, 3 equiv.) was added dropwise. After 1 h of stirring, commercially available phosphoramidate reagent **18** (193 mg, 0.42 mmol, 3 equiv.) was dissolved in 2 mL of THF then added dropwise to the mixture. The reaction was stirred at room temperature for 19 h. HCl (37%) (0.1 mL, 2.92 mmol, 21 equiv.) was then added, and the mixture was stirred for 6 h at room temperature. After completion, the solvent was evaporated, and the residue was diluted in EtOAc (30 mL) and neutralized using saturated NaHCO_3_. The mixture was extracted with EtOAc (3 × 15 mL). The organic layers were washed with brine, dried over MgSO_4_, filtrated, and concentrated under reduced pressure. The resulting crude product was then purified using silica gel flash chromatography (CH_2_Cl_2_/Acetone/MeOH 95/2.5/2.5) to give compounds **19a**–**k**, respectively.

*5-[4-[4-(Trifluoromethyl)phenyl]buta-1,3-diynyl]-uridine-5′-O-[phenyl-(isopropoxy-*l*-alaninyl)]-phosphate* (**19a**). Yield 62% as yellow powder. mp 88–90 °C (dec.). ^1^H NMR (400 MHz, MeOD) δ 8.17 (s, 1H, H_6_), 7.69 (d, *J* = 8.2 Hz, 2H, Ar H_3,5_), 7.63 (d, *J* = 8.2 Hz, 2H, Ar H_2,6_), 7.38–7.27 (m, 4H, OPh), 7.15 (t, *J* = 7.1 Hz, 1H, OPh H_4_), 5.89 (d, *J* = 3.9 Hz, 1H, H_1_′), 4.95 (p, *J* = 6.3 Hz, 1H, O*i*-Pr CH), 4.41 (ddd, *J* = 11.6, 5.5, 2.0 Hz, 1H, H_5_′), 4.34 (ddd, *J* = 11.7, 5.7, 3.1 Hz, 1H, H_5_′), 4.21–4.13 (m, 3H, H_2_′_,3_′_,4_′), 4.02–3.91 (m, 1H, CHNH), 1.34 (d, *J* = 7.1 Hz, 3H, CH_3_ Methyl), 1.21 (s, 3H, CH_3_ O*i*-Pr), 1.19 (s, 3H, CH_3_ O*i*-Pr). ^13^C NMR (101 MHz, MeOD) δ 174.36, 174.31, 163.77, 152.12, 151.03, 147.09, 134.02, 132.11, 131.24, 130.85, 126.91, 126.61, 126.57, 126.22, 121.52, 121.47, 99.37, 91.48, 84.19, 84.10, 81.06, 78.15, 76.84, 75.90, 75.53, 70.80, 70.26, 67.17, 67.12, 51.81, 21.96, 21.90, 20.76, 20.70. ^31^P NMR (162 MHz, MeOD) δ 3.86. ^19^F NMR (376 MHz, MeOD) δ −64.51. HRMS-ESI (*m*/*z*) [M + H]^+^ calcd for C_32_H_32_F_3_N_3_O_10_P 706.1782, found 706.1772.

*5-[4-(4-Pentylphenyl)buta-1,3-diynyl]-uridine-5′-O-[phenyl-(isopropoxy-*l*-alaninyl)]-phosphate* (**19b**). Yield 84% as yellow powder. mp 78–80 °C (dec.). ^1^H NMR (400 MHz, MeOD) δ 8.10 (s, 1H, H_6_), 7.37 (s, 1H, Ar H_3_), 7.35 (s, 1H, Ar H_5_), 7.34–7.27 (m, 4H, OPh), 7.19 (d, *J* = 8.2 Hz, 2H, Ar H_2,6_), 7.14 (t, *J* = 7.1 Hz, 1H, OPh H_4_), 5.89 (d, *J* = 4.1 Hz, 1H, H_1_′), 4.95 (p, *J* = 6.3 Hz, 1H, O*i*-Pr CH), 4.41 (ddd, *J* = 11.8, 5.3, 1.8 Hz, 1H, H_5_′), 4.34 (ddd, *J* = 11.7, 5.8, 2.8 Hz, 1H, H_5_′), 4.21–4.13 (m, 3H, H_2_′_,3_′_,4_′), 4.02–3.93 (m, 1H, CHNH), 2.63 (t, *J* = 7.7 Hz, 2H, CH_2_), 1.62 (p, *J* = 7.5 Hz, 2H, CH_2_), 1.36–1.28 (m, 7H, CH_3_ Methyl, 2CH_2_), 1.20 (s, 3H, CH_3_ *i*-Pr), 1.19 (s, 3H, CH_3_ *i*-Pr), 0.91 (t, *J* = 6.9 Hz, 3H, CH_3 aliphatic_). ^13^C NMR (101 MHz, MeOD) δ 174.37, 174.32, 163.90, 152.10, 152.03, 151.09, 146.45, 146.30, 133.47, 130.84, 129.81, 126.21, 121.52, 121.48, 119.85, 99.91, 91.35, 84.20, 84.12, 83.27, 78.93, 75.49, 73.98, 73.96, 70.82, 70.26, 67.25, 67.20, 51.82, 36.85, 32.54, 32.08, 23.54, 21.96, 21.92, 14.35. ^31^P NMR (162 MHz, MeOD) δ 3.86. HRMS-ESI (*m*/*z*) [M + H]^+^ calcd for C_36_H_43_N_3_O_10_P 708.2686, found 708.2680.

*5-[4-(3,5-Dimethoxyphenyl)buta-1,3-diynyl]-uridine-5′-O-[phenyl-(isopropoxy-*l*-alaninyl)]-phosphate* (**19c**). Yield 60% as orange powder. mp 70–72 °C (dec.). ^1^H NMR (400 MHz, MeOD) δ 8.12 (s, 1H, H_6_), 7.38–7.28 (m, 4H, OPh), 7.15 (t, *J* = 7.3 Hz, 1H, OPh H_4_), 6.60 (d, *J* = 2.3 Hz, 2H, Ar H_2,6_), 6.55 (t, *J* = 2.3 Hz, 1H, Ar H_4_), 5.89 (d, *J* = 4.0 Hz, 1H, H_1_′), 4.96 (p, *J* = 6.2 Hz, 1H, O*i*-Pr CH), 4.45–4.38 (m, 1H, H_5_′), 4.34 (td, *J* = 8.0, 6.7, 4.1 Hz, 1H, H_5_′), 4.17 (dd, *J* = 8.9, 4.3 Hz, 3H, H_2_′_,3_′_,4_′), 4.02–3.93 (m, 1H, CHNH), 3.77 (s, 6H, OMe), 1.35 (d, *J* = 7.1 Hz, 3H, CH_3_ Methyl), 1.20 (d, *J* = 6.2 Hz, 6H, 2CH_3_ *i*-Pr). ^13^C NMR (101 MHz, MeOD) δ 174.36, 174.31, 163.83, 162.25, 152.10, 152.03, 151.06, 146.65, 130.85, 126.22, 123.92, 121.53, 121.48, 111.16, 103.73, 99.73, 91.39, 84.19, 84.10, 82.99, 78.69, 75.52, 74.32, 73.95, 70.80, 70.27, 67.22, 67.17, 55.98, 51.82, 21.96, 21.91, 20.78, 20.72. ^31^P NMR (162 MHz, MeOD) δ 3.87. HRMS-ESI (*m*/*z*) [M + H]^+^ calcd for C_33_H_36_N_3_O_12_P 698.2108, found 698.2109.

*5-[4-[4-(Trifluoromethoxy)phenyl]buta-1,3-diynyl]-uridine-5′-O-[phenyl-(isopropoxy-*l*-alaninyl)]-phosphate* (**19d**). Yield 69% as clear yellow powder. mp 250–252 °C (dec.). ^1^H NMR (400 MHz, MeOD) δ 8.14 (s, 1H, H_6_), 7.55 (d, *J* = 9.0 Hz, 2H, Ar H_3,5_), 7.34 (t, *J* = 7.7 Hz, 2H, Ar H_2,6_), 7.29 (d, *J* = 8.3 Hz, 4H, OPh), 7.15 (t, *J* = 7.1 Hz, 1H, OPh H_4_), 5.88 (d, *J* = 3.8 Hz, 1H, H_1_′), 4.95 (p, *J* = 6.3 Hz, 1H, O*i*-Pr CH), 4.41 (dd, *J* = 11.6, 5.4 Hz, 1H, H_5_′), 4.37–4.30 (m, 1H, H_5_′), 4.22–4.13 (m, 3H, H_2_′_,3_′_,4_′), 4.02–3.91 (m, 1H, CHNH), 1.35 (d, *J* = 7.1 Hz, 3H, CH_3_ Methyl), 1.21 (s, 3H, CH_3_ *i*-Pr), 1.19 (s, 3H, CH_3_ *i*-Pr). ^13^C NMR (101 MHz, MeOD) δ 174.37, 174.32, 163.79, 152.13, 152.06, 151.06, 150.97, 146.87, 135.39, 130.83, 126.20, 123.07, 122.26, 121.87, 121.51, 121.47, 120.52, 99.57, 91.49, 84.20, 84.11, 81.22, 78.37, 75.50, 75.43, 75.03, 70.82, 70.27, 67.21, 67.16, 51.83, 21.95, 21.90, 20.76, 20.70. ^31^P NMR (162 MHz, MeOD) δ 3.85. ^19^F NMR (376 MHz, MeOD) δ −59.42. HRMS-ESI (*m*/*z*) [M + H]^+^ calcd for C_32_H_32_F_3_N_3_O_11_P 722.1706, found 722.1702.

*5-[4-(3-Aminophenyl)buta-1,3-diynyl]-uridine-5′-O-[phenyl-(isopropoxy-*l*-alaninyl)]-phosphate* (**19e**). Yield 45% as yellow powder. mp 110–112 °C (dec.). ^1^H NMR (400 MHz, MeOD) δ 8.09 (s, 1H, H_6_), 7.38–7.27 (m, 4H, OPh), 7.16 (t, *J* = 7.1 Hz, 1H, OPh H_4_), 7.07 (t, *J* = 7.8 Hz, 1H, Ar H_6_), 6.78 (s, 1H, Ar H_2_), 6.75 (d, *J* = 8.0 Hz, 2H, Ar H_3,4_), 5.89 (d, *J* = 4.2 Hz, 1H, H_1_′), 4.96 (p, *J* = 6.3 Hz, 1H, O*i*-Pr CH), 4.40 (ddd, *J* = 11.7, 5.4, 1.8 Hz, 1H, H_5_′), 4.34 (ddd, *J* = 11.8, 5.8, 2.7 Hz, 1H, H_5_′), 4.17 (dd, *J* = 9.5, 4.5 Hz, 3H, H_2_′_,3_′_,4_′), 4.03–3.93 (m, 1H, CHNH), 1.35 (d, *J* = 7.2 Hz, 3H, CH_3_ Methyl), 1.21 (s, 3H, CH_3_ *i*-Pr), 1.19 (s, 3H, CH_3_ *i*-Pr). ^13^C NMR (101 MHz, MeOD) δ 174.40, 174.35, 163.95, 152.07, 152.01, 151.13, 149.36, 146.40, 130.86, 130.29, 126.25, 122.94, 122.88, 121.52, 121.47, 119.30, 117.77, 99.96, 91.33, 84.22, 84.14, 83.86, 79.00, 75.47, 73.69, 73.40, 70.83, 70.29, 67.26, 67.21, 51.83, 21.96, 21.91, 20.76, 20.70. ^31^P NMR (162 MHz, MeOD) δ 3.87. HRMS-ESI (*m*/*z*) [M + H]^+^ calcd for C_31_H_34_N_4_O_10_P 653.2011, found 653.2007.

*5-[4-(4-Pyridyl)buta-1,3-diynyl]-uridine-5′-O-[phenyl-(isopropoxy-*l*-alaninyl)]-phosphate* (**19f**). Yield 35% as dark orange powder. mp 106–108 °C (dec.). ^1^H NMR (400 MHz, MeOD) δ 8.56 (d, *J* = 1.6 Hz, 1H, Ar H_5_), 8.55 (d, *J* = 1.7 Hz, 1H, Ar H_3_), 8.20 (s, 1H, H_6_), 7.43 (d, *J* = 1.7 Hz, 1H, Ar H_6_), 7.42 (d, *J* = 1.7 Hz, 1H, Ar H_2_), 7.35–7.27 (m, 4H, OPh), 7.15 (d, *J* = 7.2 Hz, 1H, OPh H_4_), 5.88 (d, *J* = 3.7 Hz, 1H, H_1_′), 4.95 (p, *J* = 6.3 Hz, 1H, O*i*-Pr CH), 4.42 (ddd, *J* = 11.7, 5.5, 2.1 Hz, 1H, H_5_′), 4.37–4.31 (m, 1H, H_5_′), 4.21–4.14 (m, 3H, H_2_′_,3_′_,4_′), 3.95 (dd, *J* = 10.1, 7.1 Hz, 1H, CHNH), 1.34 (d, *J* = 7.1 Hz, 3H, CH_3_ Methyl), 1.21 (s, 3H, CH_3_ *i*-Pr), 1.19 (s, 3H, CH_3_ *i*-Pr). ^13^C NMR (101 MHz, MeOD) δ 174.35, 174.30, 163.67, 152.11, 152.05, 150.99, 150.45, 147.50, 132.04, 130.85, 127.65, 126.23, 121.52, 121.47, 99.04, 91.56, 84.17, 84.09, 79.36, 79.29, 77.72, 77.38, 75.54, 70.78, 70.26, 67.13, 67.08, 51.80, 21.96, 21.91, 20.75, 20.69. ^31^P NMR (162 MHz, MeOD) δ 3.86. HRMS-ESI (*m*/*z*) [M + H]^+^ calcd for C_30_H_32_N_4_O_10_P 639.1855, found 639.1851.

*5-[4-(3-Thienyl)buta-1,3-diynyl]-uridine-5′-O-[phenyl-(isopropoxy-*l*-alaninyl)]-phosphate* (**19g**). Yield 57% as orange powder. mp 94–96 °C (dec.). ^1^H NMR (400 MHz, MeOD) δ 8.10 (s, 1H, H_6_), 7.70 (d, *J* = 3.0 Hz, 1H, H_a_), 7.45 (dd, *J* = 5.1, 2.9 Hz, 1H, H_c_), 7.37–7.27 (m, 4H, OPh), 7.18–7.10 (m, 2H, OPh H_4_, H_d_), 5.88 (d, *J* = 4.0 Hz, 1H, H_1_′), 4.96 (p, *J* = 6.3 Hz, 1H, O*i*-Pr CH), 4.45–4.30 (m, 2H, H_5_′), 4.21–4.12 (m, 3H, H_2_′_,3_′_,4_′), 4.01–3.92 (m, 1H, CHNH), 1.35 (d, *J* = 7.1 Hz, 3H, CH_3_ Methyl), 1.21 (s, 3H, CH_3_ *i*-Pr), 1.19 (s, 3H, CH_3_ *i*-Pr). ^13^C NMR (101 MHz, MeOD) δ 174.38, 174.33, 163.87, 152.10, 152.03, 151.09, 146.53, 132.93, 131.00, 130.83, 127.24, 126.20, 121.72, 121.52, 121.47, 99.86, 91.40, 84.20, 84.12, 78.81, 78.27, 75.47, 74.02, 70.83, 70.27, 67.25, 67.20, 51.82, 21.96, 21.91, 20.76, 20.69. ^31^P NMR (162 MHz, MeOD) δ 3.85. HRMS-ESI (*m*/*z*) [M + H]^+^ calcd for C_29_H_31_N_3_O_10_PS 644.1456, found 644.1462.

*5-Deca-1,3-diynyl-uridine-5′-O-[phenyl-(isopropoxy-*l*-alaninyl)]-phosphate* (**19h**). Yield 28% as clear yellow powder. mp 92–94 °C (dec.). ^1^H NMR (400 MHz, MeOD) δ 8.02 (s, 1H, H_6_), 7.37 (t, *J* = 7.9 Hz, 2H, OPh), 7.29 (d, *J* = 7.7 Hz, 2H, OPh), 7.20 (t, *J* = 7.5 Hz, 1H, OPh H_4_), 5.86 (d, *J* = 4.4 Hz, 1H, H_1_′), 4.97 (p, *J* = 6.2 Hz, 1H, O*i*-Pr CH), 4.41–4.29 (m, 2H, H_5_′), 4.19–4.10 (m, 3H, H_2_′_,3_′_,4_′), 4.01–3.92 (m, 1H, CHNH), 2.32 (t, *J* = 6.9 Hz, 2H, CH_2_), 1.51 (p, *J* = 6.8 Hz, 2H, CH_2_), 1.41 (q, *J* = 7.4 Hz, 2H, CH_2_), 1.34 (d, *J* = 6.7 Hz, 3H, CH_3_ Methyl), 1.31–1.25 (m, 4H, 2CH_2_), 1.23 (d, *J* = 2.3 Hz, 3H, CH_3_ *i*-Pr), 1.21 (d, *J* = 2.3 Hz, 3H, CH_3_ *i*-Pr), 0.91 (t, *J* = 6.8 Hz, 3H, CH_3 aliphatic_). ^13^C NMR (101 MHz, MeOD) δ 174.37, 174.32, 152.13, 152.06, 146.20, 130.83, 126.19, 121.54, 121.49, 100.11, 91.27, 86.29, 84.18, 84.09, 79.56, 75.37, 70.81, 70.25, 67.31, 67.26, 67.15, 65.95, 51.80, 32.44, 29.60, 29.29, 23.59, 21.98, 21.93, 20.77, 20.70, 20.06, 14.38. ^31^P NMR (162 MHz, MeOD) δ 3.80. HRMS-ESI (*m*/*z*) [M + H]^+^ calcd for C_31_H_41_N_3_O_10_P 646.2524, found 646.2524.

*5-[4-(2-Pyridyl)buta-1,3-diynyl]-uridine-5′-O-[phenyl-(isopropoxy-*l*-alaninyl)]-phosphate* (**19i**). Yield 49% as dark orange powder. mp 92–94 °C (dec.). ^1^H NMR (400 MHz, MeOD) δ 8.54 (d, *J* = 5.0 Hz, 1H, Ar H_3_), 8.17 (d, *J* = 1.5 Hz, 1H, H_6_), 7.85 (t, *J* = 7.7 Hz, 1H, Ar H_5_), 7.57 (d, *J* = 7.9 Hz, 1H, Ar H_6_), 7.44 (dd, *J* = 7.7, 5.1 Hz, 1H, Ar H_4_), 7.38–7.26 (m, 4H, OPh), 7.13 (t, *J* = 7.1 Hz, 1H, OPh H_4_), 5.88 (d, *J* = 2.7 Hz, 1H, H_1_′), 4.96 (p, *J* = 6.3 Hz, 1H, O*i*-Pr CH), 4.41 (dd, *J* = 11.6, 5.7 Hz, 1H, H_5_′), 4.36–4.30 (m, 1H, H_5_′), 4.18 (t, *J* = 4.6 Hz, 3H, H_2_′_,3_′_,4_′), 4.01–3.90 (m, 1H, CHNH), 1.35 (d, *J* = 7.2 Hz, 3H, CH_3_ Methyl), 1.21 (s, 3H, CH_3_ *i*-Pr), 1.19 (s, 3H, CH_3_ *i*-Pr). ^13^C NMR (101 MHz, MeOD) δ 163.89, 151.05, 149.19, 142.69, 138.60, 129.76, 125.52, 115.03, 98.55, 94.62, 89.00, 86.41, 82.18, 80.80, 77.38, 76.16, 74.70, 62.90, 27.49, 25.51. ^31^P NMR (162 MHz, MeOD) δ 3.89. HRMS-ESI (*m*/*z*) [M + H]^+^ calcd for C_30_H_32_N_4_O_10_P 639.1814, found 639.1811.

*5-(4-Cyclopropylbuta-1,3-diynyl)-uridine-5′-O-[phenyl-(isopropoxy-*l*-alaninyl)]-phosphate* (**19j**). Yield 48% as clear powder. mp 142–144 °C. ^1^H NMR (400 MHz, MeOD) δ 8.00 (s, 1H, H_6_), 7.38 (t, *J* = 7.8 Hz, 2H, OPh), 7.28 (d, *J* = 8.0 Hz, 2H, OPh), 7.20 (t, *J* = 7.3 Hz, 1H, OPh H_4_), 5.86 (d, *J* = 4.3 Hz, 1H, H_1_′), 4.97 (p, *J* = 6.2 Hz, 1H, O*i*-Pr CH), 4.40–4.29 (m, 2H, H_5_′), 4.19–4.10 (m, 3H, H_2_′_,3_′_,4_′), 4.00–3.91 (m, 1H, CHNH), 1.40 (td, *J* = 8.6, 4.5 Hz, 1H, CH_cyclopropyl_), 1.34 (d, *J* = 7.1 Hz, 3H, CH_3_ Methyl), 1.22 (dd, *J* = 6.8, 2.7 Hz, 6H, 2CH_3_ *i*-Pr), 0.88 (dt, *J* = 7.8, 3.4 Hz, 2H, CH2_cyclopropyl_), 0.73–0.66 (m, 2H, CH2_cyclopropyl_). ^13^C NMR (101 MHz, MeOD) δ 174.38, 174.33, 164.08, 152.11, 152.05, 151.10, 146.19, 130.83, 126.21, 121.52, 121.48, 100.17, 91.30, 89.46, 84.18, 84.10, 79.83, 75.38, 70.82, 70.27, 67.30, 67.25, 66.49, 61.11, 51.81, 21.97, 21.93, 20.74, 20.68, 9.46, 0.78. ^31^P NMR (162 MHz, MeOD) δ 3.81. HRMS-ESI (*m*/*z*) [M + H]^+^ calcd for C_31_H_33_N_3_O_10_P 638.1898, found 638.1898.

*5-(4-Phenylbuta-1,3-diynyl)-uridine-5′-O-[phenyl-(isopropoxy-*l*-alaninyl)]-phosphate* (**19k**). Yield 49% as orange powder. mp 88–90 °C. ^1^H NMR (400 MHz, MeOD) δ 8.12 (s, 1H, H_6_), 7.45 (dd, *J* = 8.2, 1.7 Hz, 2H, Ar), 7.42–7.34 (m, 3H, Ar), 7.34–7.26 (m, 4H, OPh), 7.14 (t, *J* = 7.0 Hz, 1H, OPh H_4_), 5.89 (d, *J* = 4.0 Hz, 1H, H_1_′), 4.96 (p, *J* = 6.3 Hz, 1H, O*i*-Pr CH), 4.41 (ddd, *J* = 11.8, 5.3, 1.9 Hz, 1H, H_5_’), 4.34 (ddd, *J* = 11.8, 5.7, 2.7 Hz, 1H, H_5_′), 4.21–4.13 (m, 3H, H_2_′_,3_′_,4_′), 4.03–3.93 (m, 1H, CHNH), 1.35 (d, *J* = 7.1 Hz, 3H, CH_3_ Methyl), 1.20 (s, 3H, CH_3_ *i*-Pr), 1.19 (s, 3H, CH_3_ *i*-Pr). ^13^C NMR (101 MHz, MeOD) δ 174.37, 174.32, 163.92, 152.10, 152.03, 151.11, 146.60, 133.46, 130.83, 130.63, 129.70, 126.20, 122.71, 121.51, 121.47, 99.78, 91.38, 84.19, 84.11, 82.88, 78.71, 75.49, 74.51, 74.35, 70.81, 70.26, 67.24, 67.19, 51.82, 21.96, 21.91, 20.76, 20.70. ^31^P NMR (162 MHz, MeOD) δ 3.86. HRMS-ESI (*m*/*z*) [M + H]^+^ calcd for C_28_H_33_N_3_O_10_P 602.1896, found 602.1898.

### 3.2. Antiviral Activity Assays

#### 3.2.1. Against SARS-CoV-2 Virus

##### Cell Lines, Cell Culture, and Reagents

The human pulmonary alveolar A549-hACE2 cells were engineered using a lentiviral vector from Flash Therapeutics (Toulouse, France) to express the human receptor ACE2. A549 cells obtained from ECACC (#86012804) were transduced and maintained in RPMI supplemented with 10% heat inactivated fetal bovine serum (FBS), 50 U/mL of penicillin, 50 µg/mL of streptomycin, 25 mM of HEPES, and 1 mM of sodium pyruvate at 37 °C with 5% CO_2_. Cells were then sorted by FACS in order to obtain an A549-hACE2 population stably expressing hACE2.

##### SARS-CoV-2 Virus Stock and Titration for Antiviral Screening

The strain Delta (B.1.617.2) hCoV-19/France/HDF-IPP11602i/2021 was supplied by the National Reference Centre for Respiratory Viruses hosted by Institut Pasteur (Paris, France) and headed by Pr. Sylvie van der Werf. The human sample from which strain hCoV-19/France/HDF-IPP11602i/2021 was isolated has been provided by Dr. Guiheneuf Raphaël, CH Simone Veil, Beauvais, France. Moreover, the strain was supplied through the European Virus Archive goes Global (EVAg) platform, a project that has received funding from the European Union’s Horizon 2020 research and innovation program under the grant agreement No 653316. The viruses were propagated in VeroE6 cells with DMEM containing 25 mM HEPES at 37 °C with 5% CO_2_ and were harvested 72 h post inoculation. Virus stocks were stored at –80 °C. Virus titration from stocks and infected cell culture supernatants were monitored using plaque assays on a monolayer of VeroE6 cells, using 100 µL of virus solution. Samples were serially diluted, and the plaque forming unit (PFU) values were determined using crystal violet coloration on cells and subsequent scoring of the amounts of wells displaying cytopathic effects. Calculations allow us to determine the titer as the number of PFU/mL.

##### Assessment of Antiviral Activity

Antiviral activity was assessed by a cytopathic effect (CPE) reduction assay on A549-hACE2 cells. Briefly, 25,000 cells per well were cultured in 96-well culture plates for 24 h. Drugs were diluted from stock (5 mM or 10 mM in DMSO) to different concentrations (from 20 µM on 8 dilutions) for testing antiviral activity on A549-hACE2. Cells were incubated with 100 µL of the compounds diluted in RPMI, 0.1% DMSO at the indicated concentrations, and the plate were incubated for 2 h. Subsequently, cells either were mock infected (for analysis of cytotoxicity of the compound) or infected with 400 PFU of virus per well (MOI of 0.01) in a total volume of 110 μL of medium with compound. Cell viability was assessed 4 days post infection by MTS assay using a Cell Titer 96 aqueous cell proliferation kit (Promega, Madison, WI, USA) for cell cytotoxicity or Viral Tox GloTM (Tox Glo assay) for CPE measurement. Absorption at 495 nm (MTS) or luminescence (Tox Glo) were measured with an EnVision multilabel plate reader (PerkinElmer, Waltham, MA, USA). The 50% effective concentration (EC50 (the concentration required to inhibit virus-induced cell death by 50%)) and the 50% cytotoxic concentration (CC50 (the concentration that reduces the viability of uninfected cells to 50% of that of untreated control cells)) were determined using 4-parameter nonlinear regression with GraphPad Prism v8.0, based on the following calculations:

$$\mathrm{Cell}\;\mathrm{cytotoxicity},\;\%\mathrm{Viability}=\left(\frac{drug}{cell\;DMSO}\right)\times 100$$

$$\mathrm{Percentage}\;\mathrm{of}\;\mathrm{viral}\;\mathrm{replication}\;\mathrm{inhibition},\;\%\mathrm{Inhibit}=\left(\frac{drug-infected\;cells}{cell\;DMSO-infected\;cells}\right)\times 100$$

Data are mean ± sd of three biological replicates.

Screening against other viruses was carried out by HTS on the Virocrib platform (https://www.virocrib.fr/, accessed on 23 December 2024).

## 4. Conclusions

In conclusion, this study highlights the synthesis and antiviral evaluation of novel *C5*-substituted-(1,3-diyne)-uridine derivatives and their phosphoramidate pronucleotides. Despite structural variations and successful synthesis using Sonogashira and Cadiot–Chodkiewicz reactions, these compounds showed no significant (≥85% inhibition of infected cell viability) antiviral activity against selected RNA viruses with ≤85% toxicity. The lack of effectiveness may be attributed to challenges in cellular activation, poor binding affinity to diverse viral RdRps, and potential interference in enzymatic recognition due to *C5*-modifications.

## Figures and Tables

**Table 1:** Yields obtained for the Cadiot–Chodkiewicz cross-coupling.

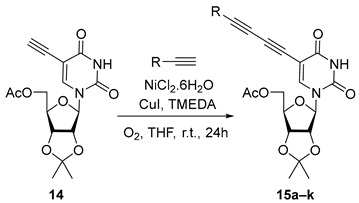			
Entry	R Substituent	Product Number	Yield [%]
1	4-(trifluoromethyl)phenyl-	**15a**	45
2	4-pentylphenyl-	**15b**	47
3	3,5-dimethoxyphenyl-	**15c**	35
4	4-(trifluoromethoxy)phenyl-	**15d**	50
5	3-aminophenyl-	**15e**	55
6	4-pyridyl-	**15f**	31
7	3-thienyl	**15g**	67
8	*n*-hexyl-	**15h**	25
9	2-pyridyl-	**15i**	37
10	cyclopropyl-	**15j**	40
11	phenyl-	**15k**	66

## Data Availability

Data are contained within the article and Supplementary Materials.

## Supplementary Materials

The following supporting information can be downloaded at: https://www.mdpi.com/article/10.3390/molecules30010096/s1, ^1^H-, ^13^C-, ^19^F-, and ^31^P-NMR spectra of all compounds.
