# Supplementary material for: Synthesis and Antiviral Evaluation of 5-(4-Aryl-1,3-butadiyn-1-yl)-uridines and Their Phosphoramidate Pronucleotides
Source: Molecules. 2024 Dec 29;30(1):96. doi: 10.3390/molecules30010096 (PMC11722124; doi:10.3390/molecules30010096)
Supplement: Supplementary file 1 [file molecules-30-00096-s001.zip › molecules-3354861-supplementary.pdf]

CD$_3$OD

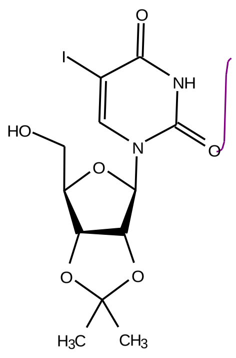

11

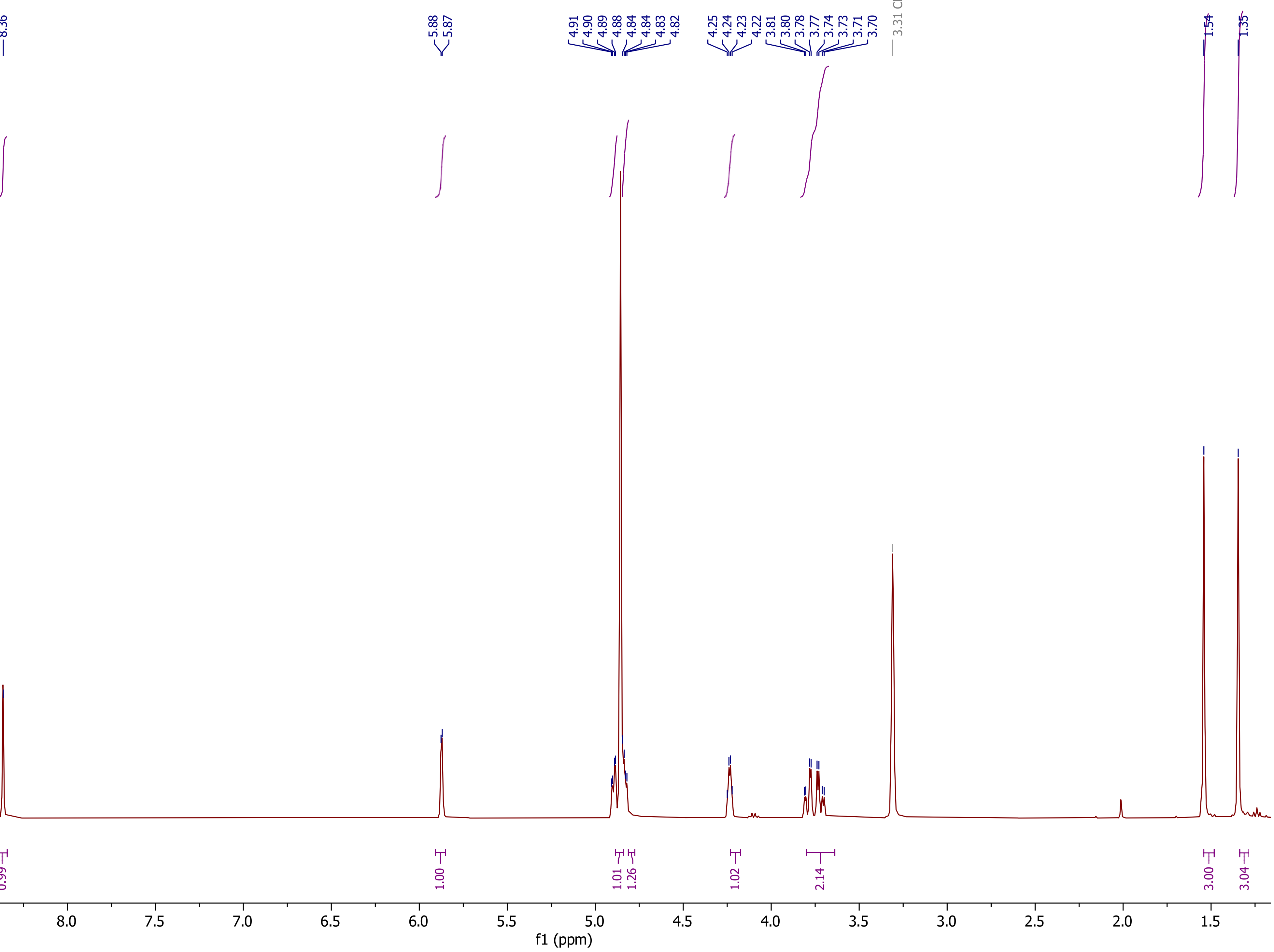
8.36
5.88
5.87
4.91
4.90
4.89
4.88
4.84
4.84
4.83
4.82
4.25
4.24
4.23
4.22
3.81
3.80
3.78
3.77
3.74
3.73
3.71
3.70
3.31 CD3OD
1.54
1.35
0.99
1.00
1.01
1.26
1.02
2.14
3.00
3.04
9.0
8.5
8.0
7.5
7.0
6.5
6.0
5.5
5.0
4.5
4.0
3.5
3.0
2.5
2.0
1.5
f1 (ppm)

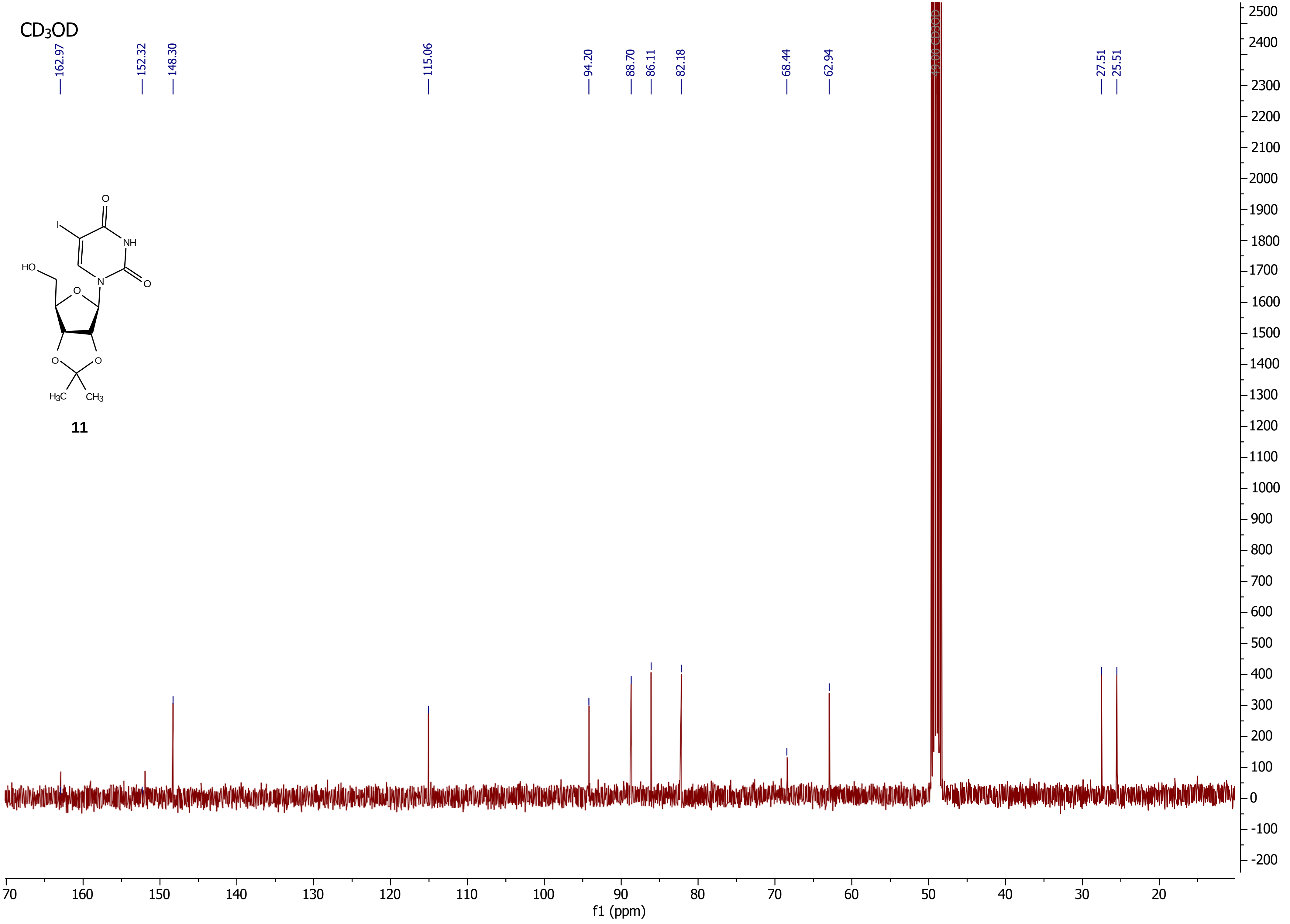
CD3OD
162.97
152.32
148.30
115.06
94.20
88.70
86.11
82.18
68.44
62.94
49.00 CD3OD
27.51
25.51
HO
O
I
NH
N
O
O
O
H3C
CH3
11
f1 (ppm)

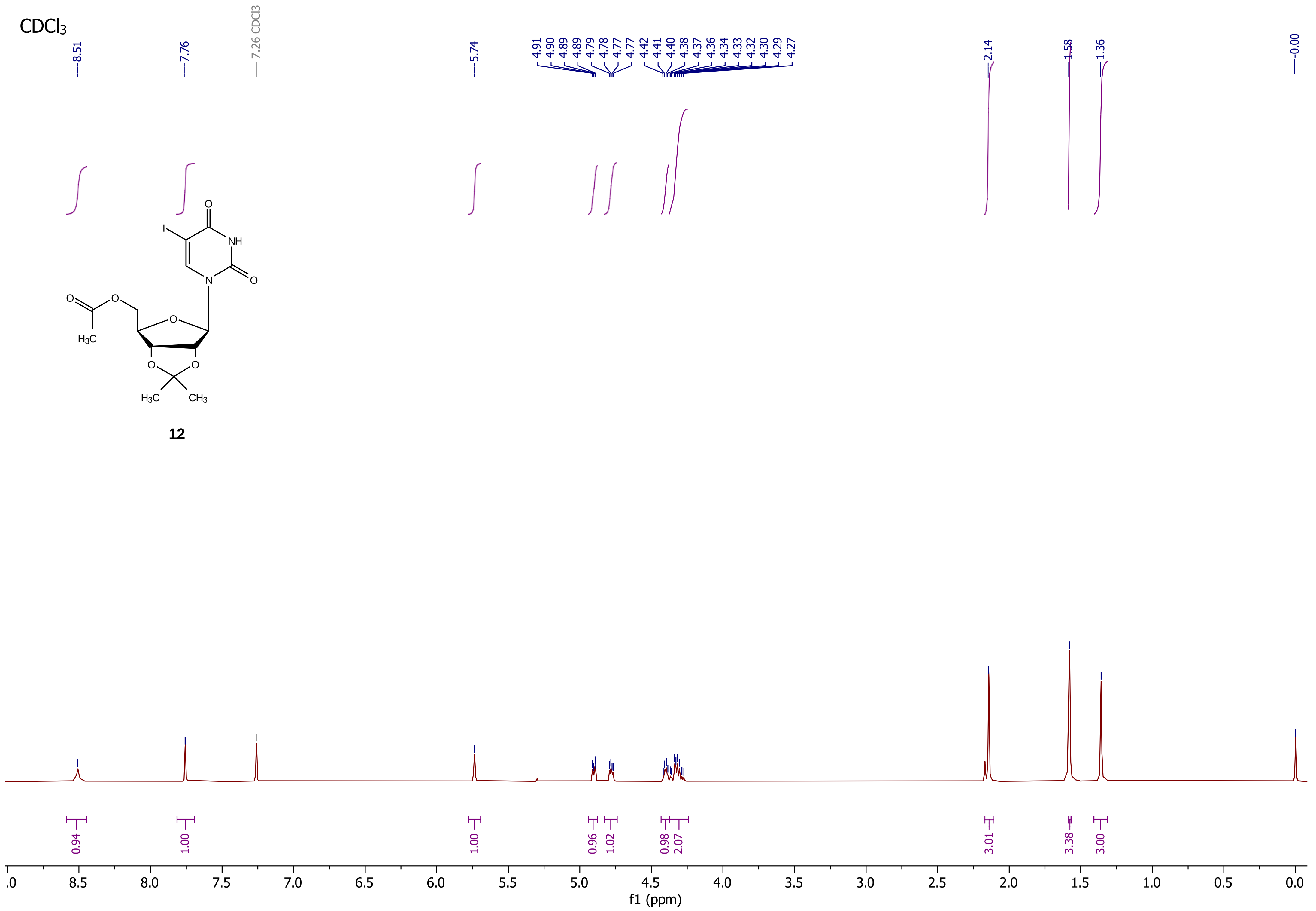
CDCl3
8.51
7.76
7.26 CDCl3
5.74
4.91 4.90 4.89 4.89 4.79 4.78 4.77 4.77 4.42 4.41 4.40 4.38 4.37 4.36 4.34 4.33 4.32 4.30 4.29 4.27
2.14
1.58
1.36
-0.00
12
0.94
1.00
1.00
0.96
1.02
0.98
2.07
3.01
3.38
3.00
f1 (ppm)

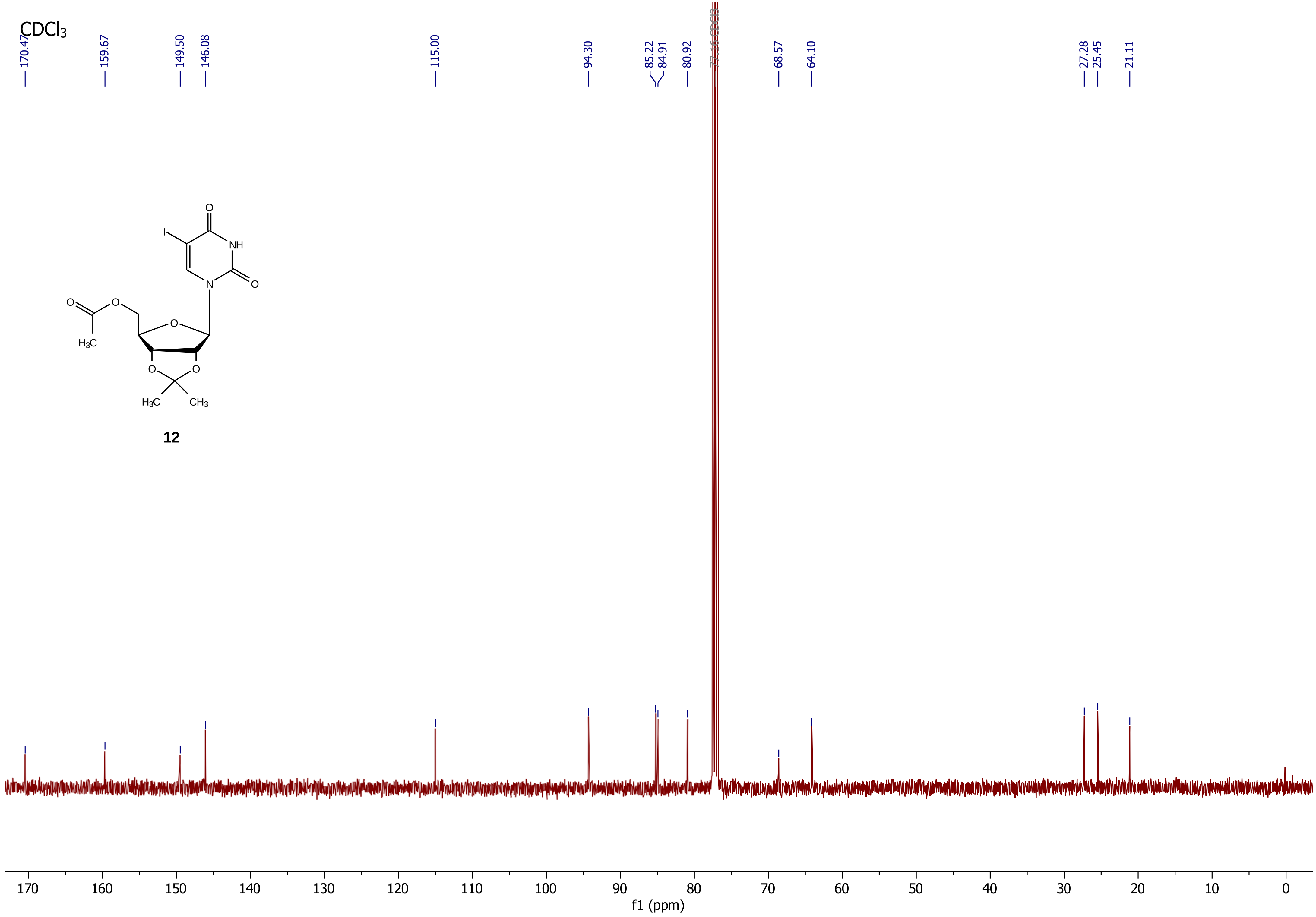
CDCl3
170.47
159.67
149.50
146.08
115.00
94.30
85.22
84.91
80.92
68.57
64.10
27.28
25.45
21.11
I
NH
O
N
O
O
O
H3C
O
O
H3C
CH3
12
170
160
150
140
130
120
110
100
90
80
70
60
50
40
30
20
10
0
f1 (ppm)

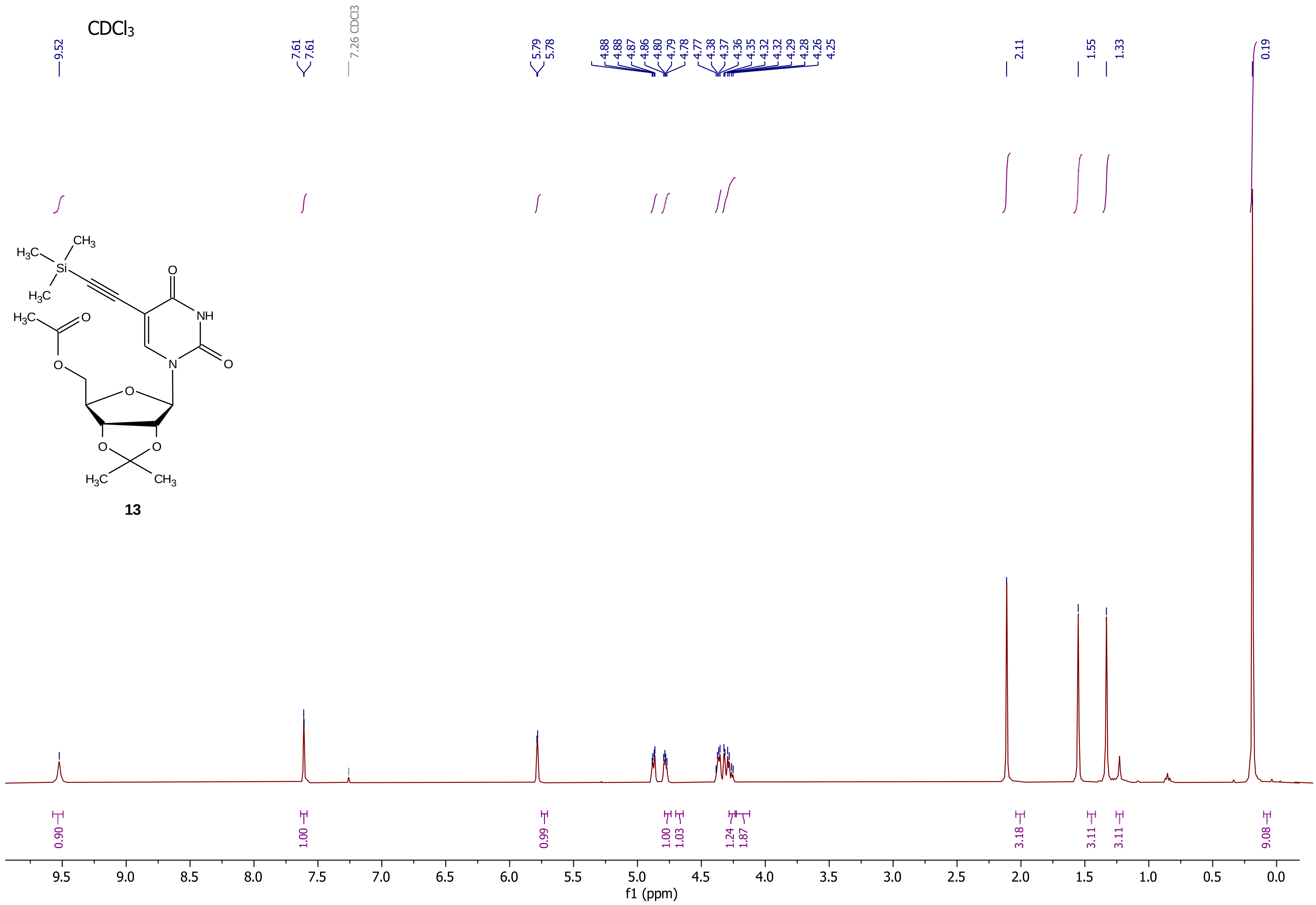

CDCl3
9.52
7.61
7.61
7.26 CDCl3
5.79
5.78
4.88
4.88
4.87
4.86
4.80
4.79
4.78
4.77
4.38
4.37
4.36
4.35
4.32
4.32
4.29
4.28
4.26
4.25
2.11
1.55
1.33
0.19
0.90
1.00
0.99
1.00
1.03
1.24
1.87
3.18
3.11
3.11
9.08
9.5
9.0
8.5
8.0
7.5
7.0
6.5
6.0
5.5
5.0
4.5
4.0
3.5
3.0
2.5
2.0
1.5
1.0
0.5
0.0
f1 (ppm)

13

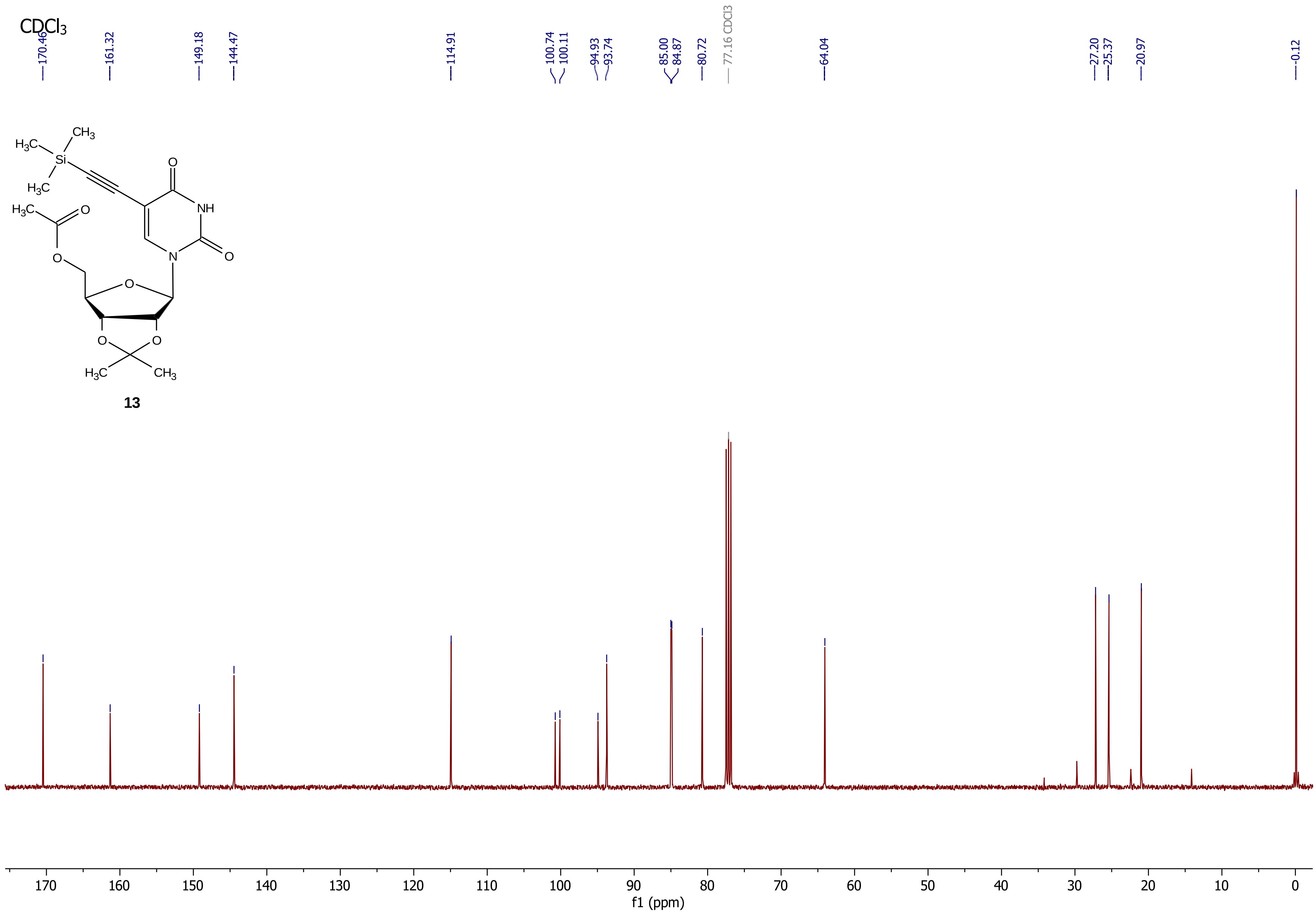
CDCl3
170.46
161.32
149.18
144.47
114.91
100.74
100.11
94.93
93.74
85.00
84.87
80.72
77.16 CDCl3
64.04
27.20
25.37
20.97
-0.12
H3C
CH3
Si
O
NH
O
N
O
H3C
O
O
O
O
H3C
CH3
170
160
150
140
130
120
110
100
90
80
70
60
50
40
30
20
10
0
f1 (ppm)

13

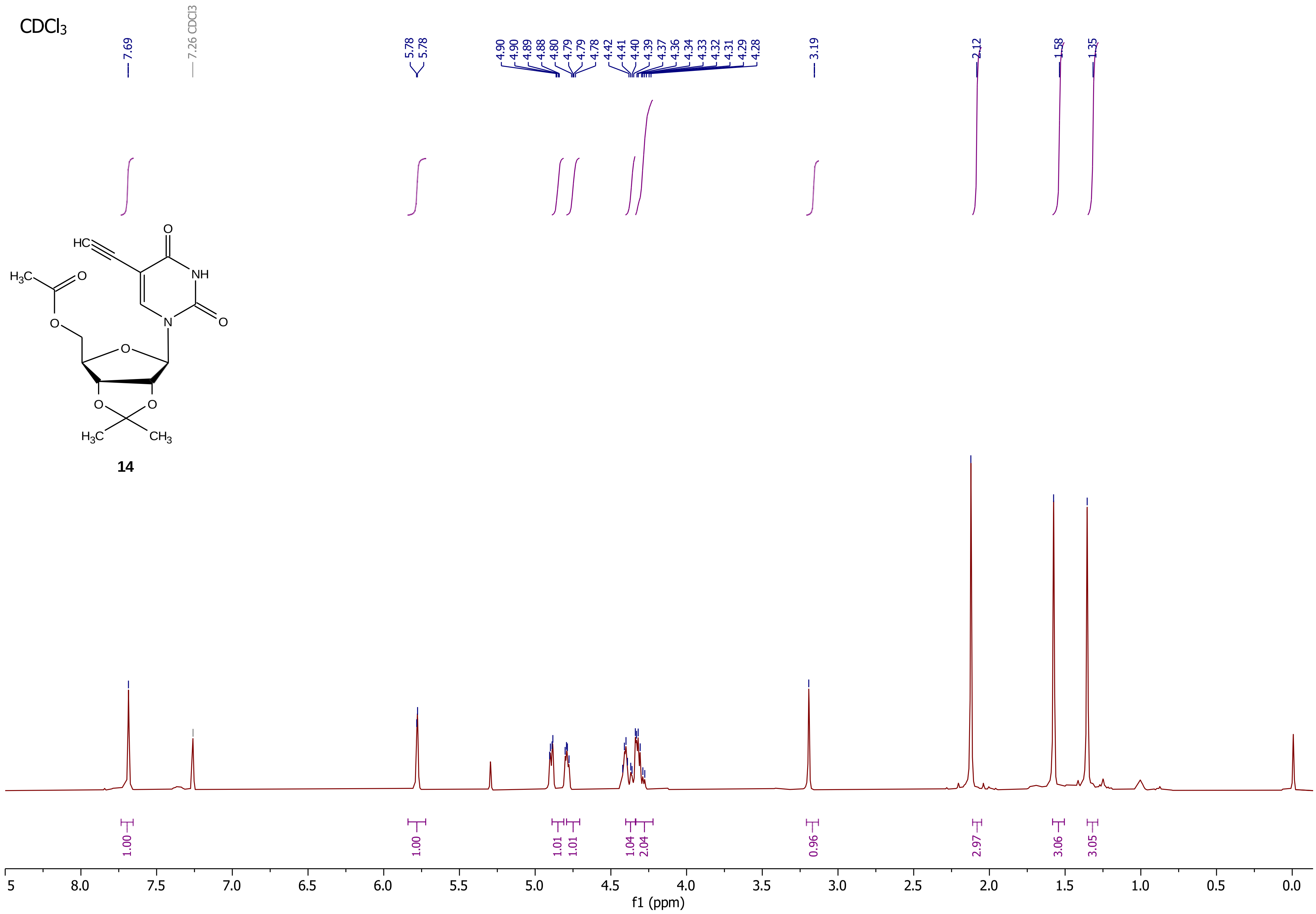
CDCl3
7.69
7.26 CDCl3
5.78
5.78
4.90
4.90
4.89
4.88
4.80
4.79
4.79
4.78
4.42
4.41
4.40
4.39
4.37
4.36
4.34
4.33
4.32
4.31
4.29
4.28
3.19
2.12
1.58
1.35
HC
O
NH
N
O
H3C
O
O
O
O
O
H3C
CH3
14
1.00
1.00
1.01
1.01
1.04
2.04
0.96
2.97
3.06
3.05
8.0
7.5
7.0
6.5
6.0
5.5
5.0
4.5
4.0
3.5
3.0
2.5
2.0
1.5
1.0
0.5
0.0
f1 (ppm)

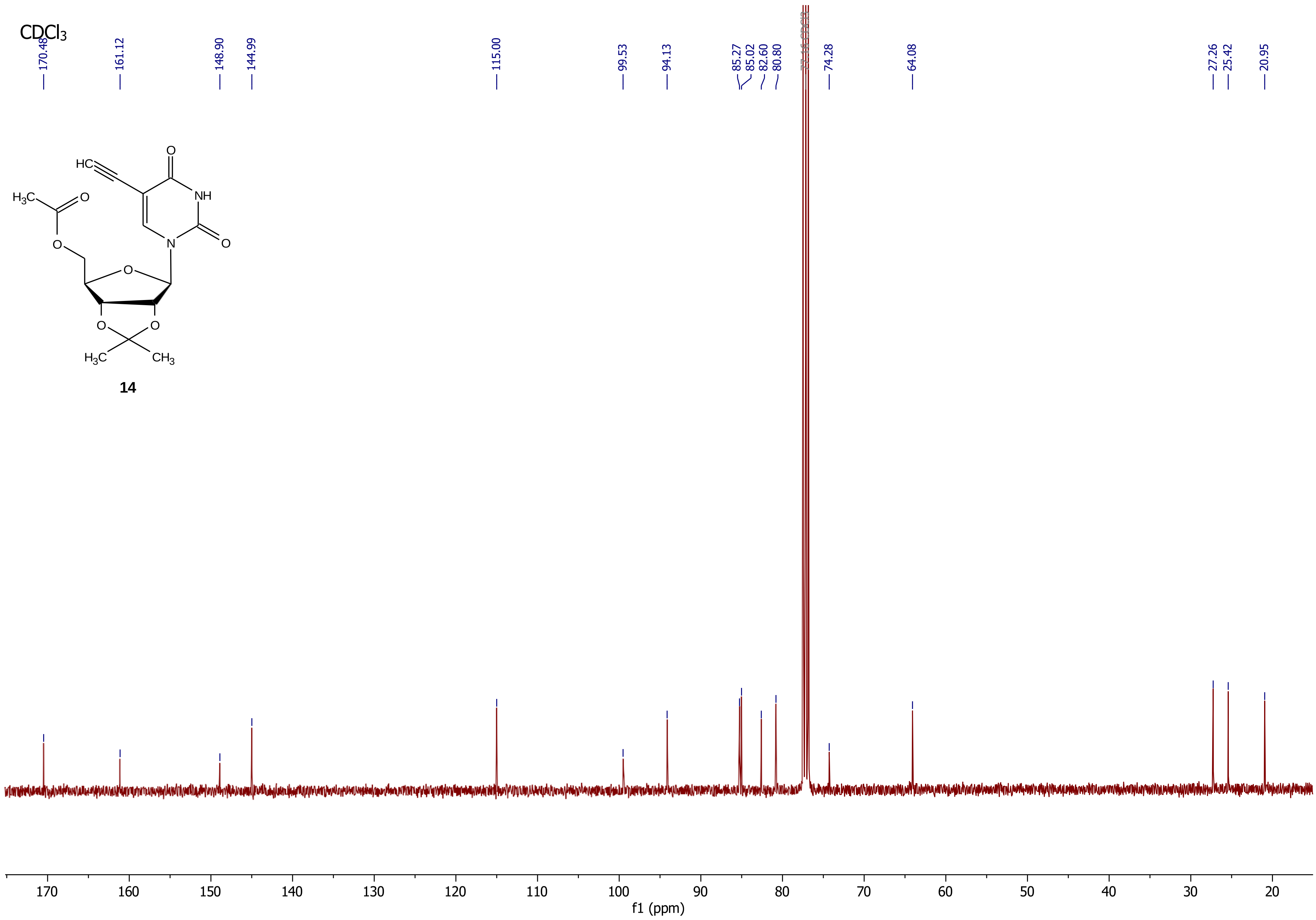
CDCl3
170.48
161.12
148.90
144.99
115.00
99.53
94.13
85.27
85.02
82.60
80.80
74.28
64.08
27.26
25.42
20.95
HC
O
NH
O
N
H3C
O
O
O
O
O
H3C
CH3
14
170
160
150
140
130
120
110
100
90
80
70
60
50
40
30
20
f1 (ppm)

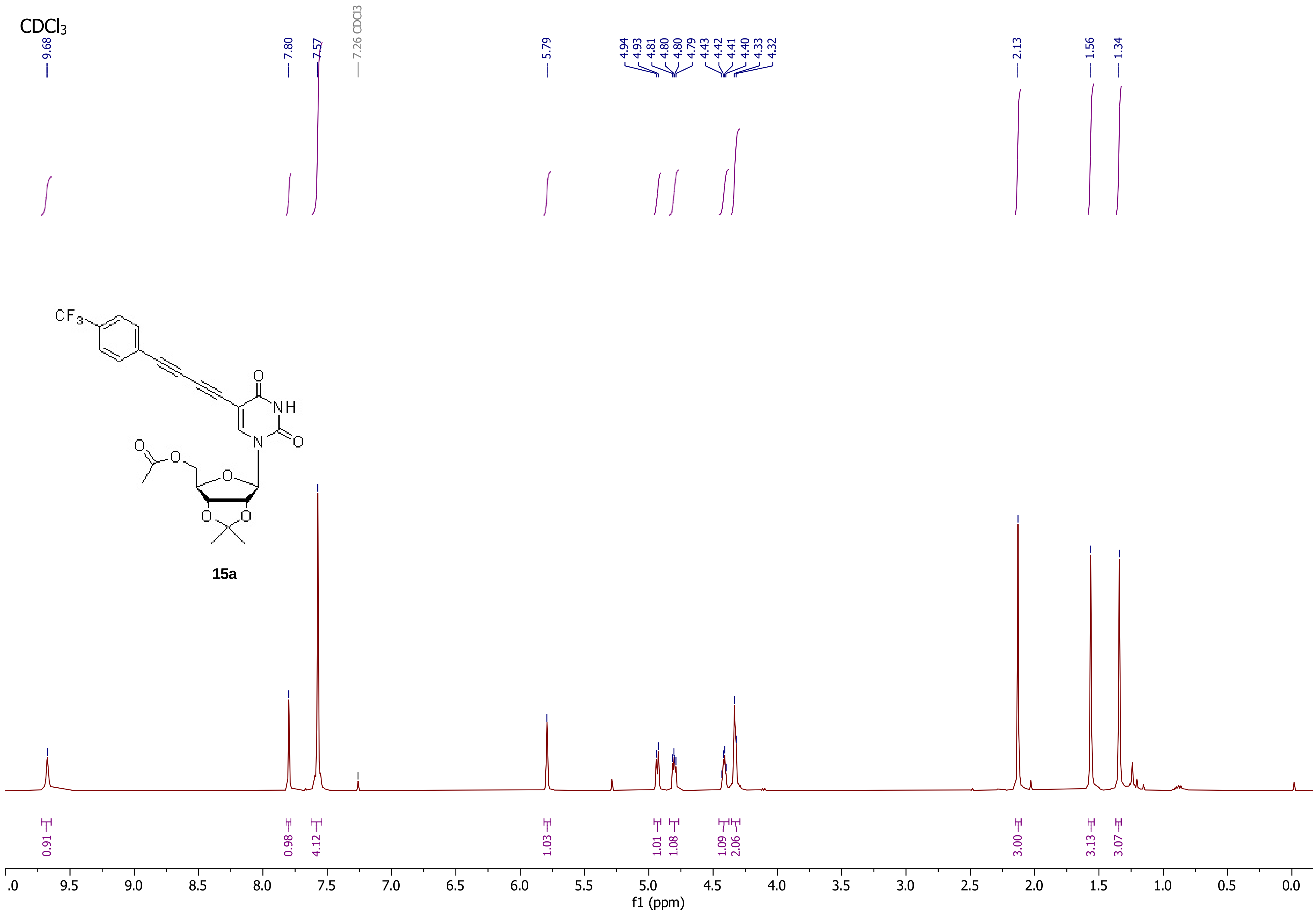

CDCl3
9.68
7.80
7.57
7.26 CDCl3
5.79
4.94
4.93
4.81
4.80
4.80
4.79
4.43
4.42
4.41
4.40
4.33
4.32
2.13
1.56
1.34
CF3
O
NH
N
O
O
O
O
O
O
15a
0.91
0.98
4.12
1.03
1.01
1.08
1.09
2.06
3.00
3.13
3.07
.0
9.5
9.0
8.5
8.0
7.5
7.0
6.5
6.0
5.5
5.0
4.5
4.0
3.5
3.0
2.5
2.0
1.5
1.0
0.5
0.0
f1 (ppm)

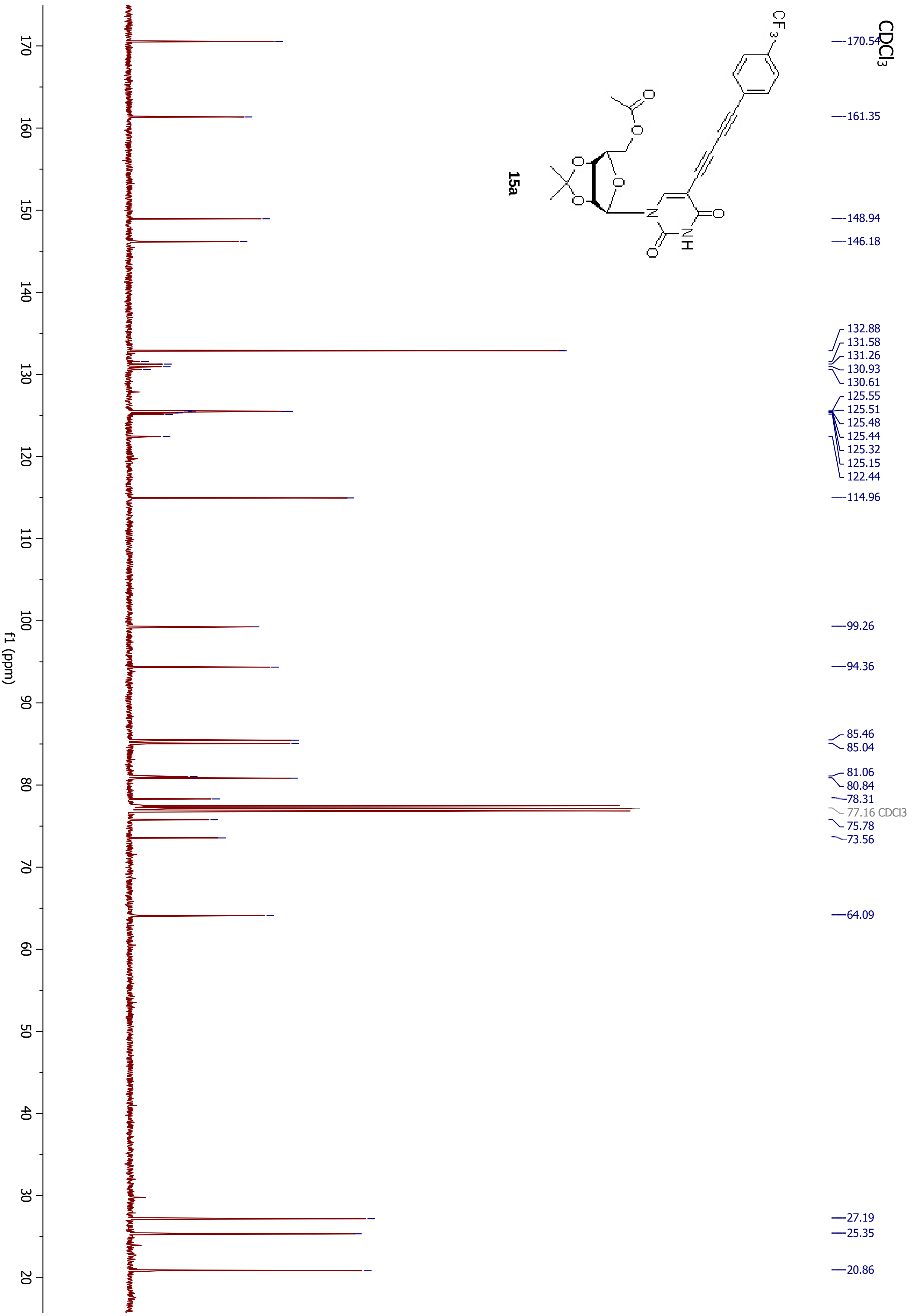

CDCl3
15a
CF3
O
NH
N
O
O
O
O
O
O
170.54
161.35
148.94
146.18
132.88
131.58
131.26
130.93
130.61
125.55
125.51
125.48
125.44
125.32
125.15
122.44
114.96
99.26
94.36
85.46
85.04
81.06
80.84
78.31
77.16 CDCl3
75.78
73.56
64.09
27.19
25.35
20.86
f1 (ppm)
170
160
150
140
130
120
110
100
90
80
70
60
50
40
30
20

CDCl3

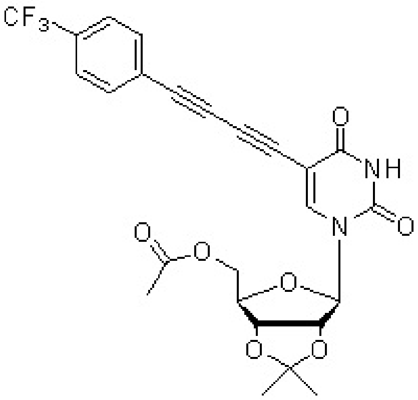
CF3
O
NH
N
O
O
O
O
O
O
O

15a

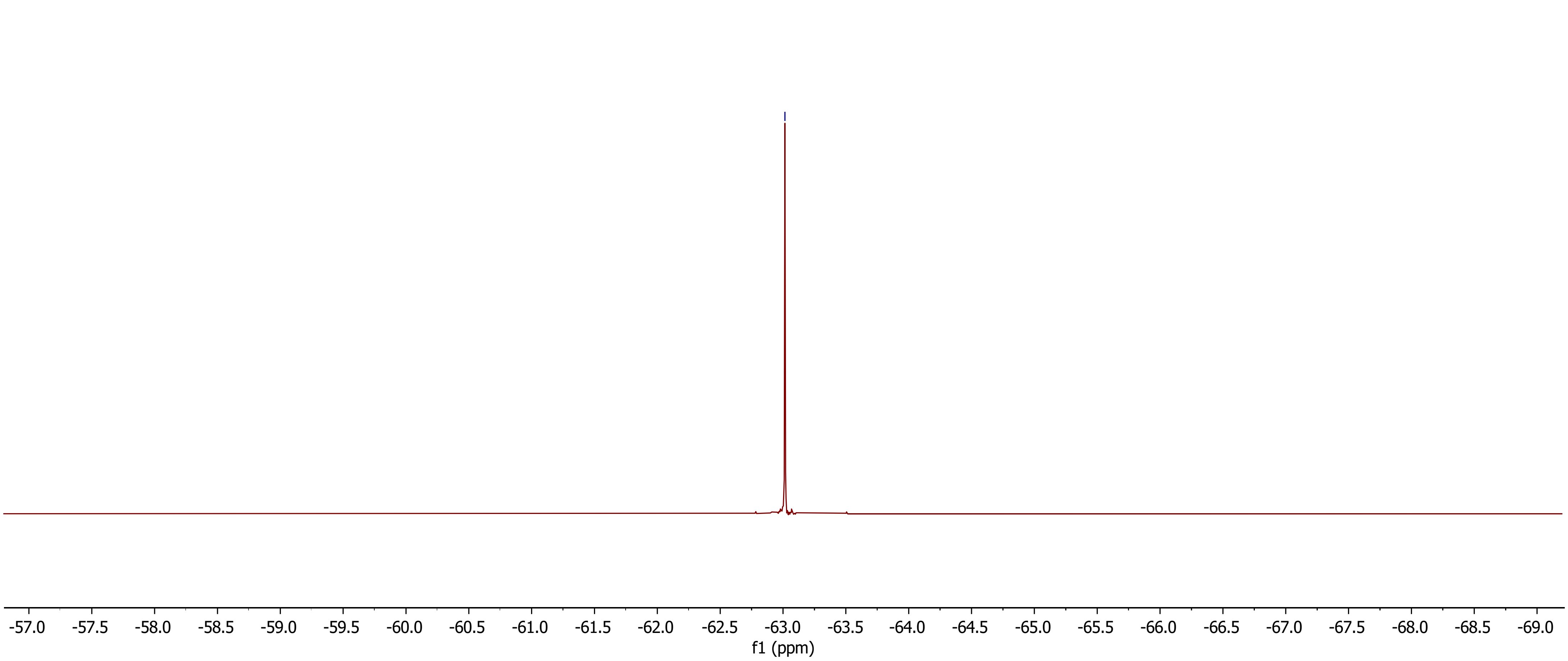
-63.02
-57.0
-57.5
-58.0
-58.5
-59.0
-59.5
-60.0
-60.5
-61.0
-61.5
-62.0
-62.5
-63.0
-63.5
-64.0
-64.5
-65.0
-65.5
-66.0
-66.5
-67.0
-67.5
-68.0
-68.5
-69.0
f1 (ppm)

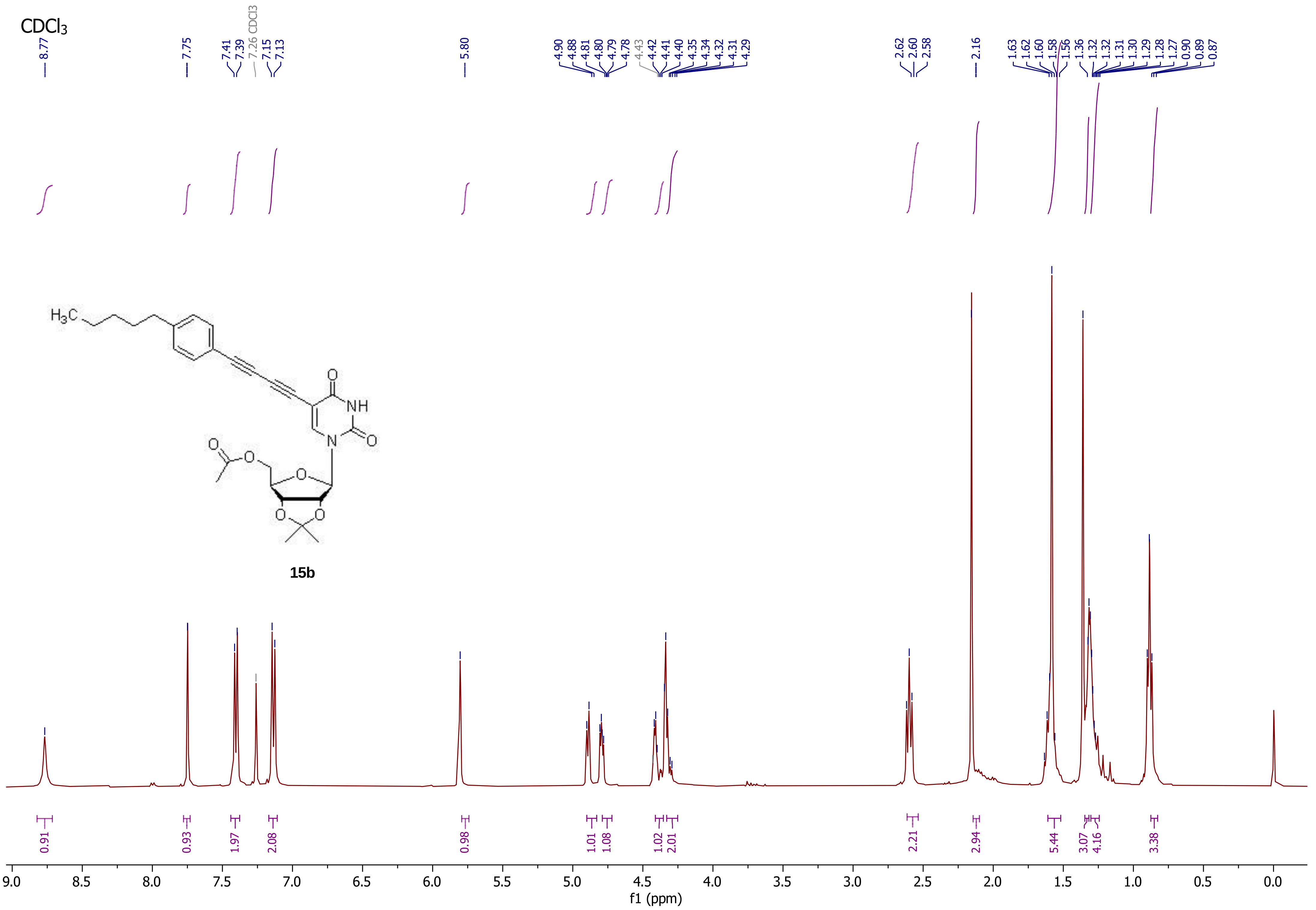
CDCl3
8.77
7.75
7.41
7.39
7.26 CDCl3
7.15
7.13
5.80
4.90
4.88
4.81
4.80
4.79
4.78
4.43
4.42
4.41
4.40
4.35
4.34
4.32
4.31
4.29
2.62
2.60
2.58
2.16
1.63
1.62
1.60
1.58
1.56
1.36
1.32
1.32
1.31
1.30
1.29
1.28
1.27
0.90
0.89
0.87
H3C
O
NH
N
O
15b
0.91
0.93
1.97
2.08
0.98
1.01
1.08
1.02
2.01
2.21
2.94
5.44
3.07
4.16
3.38
9.0
8.5
8.0
7.5
7.0
6.5
6.0
5.5
5.0
4.5
4.0
3.5
3.0
2.5
2.0
1.5
1.0
0.5
0.0
f1 (ppm)

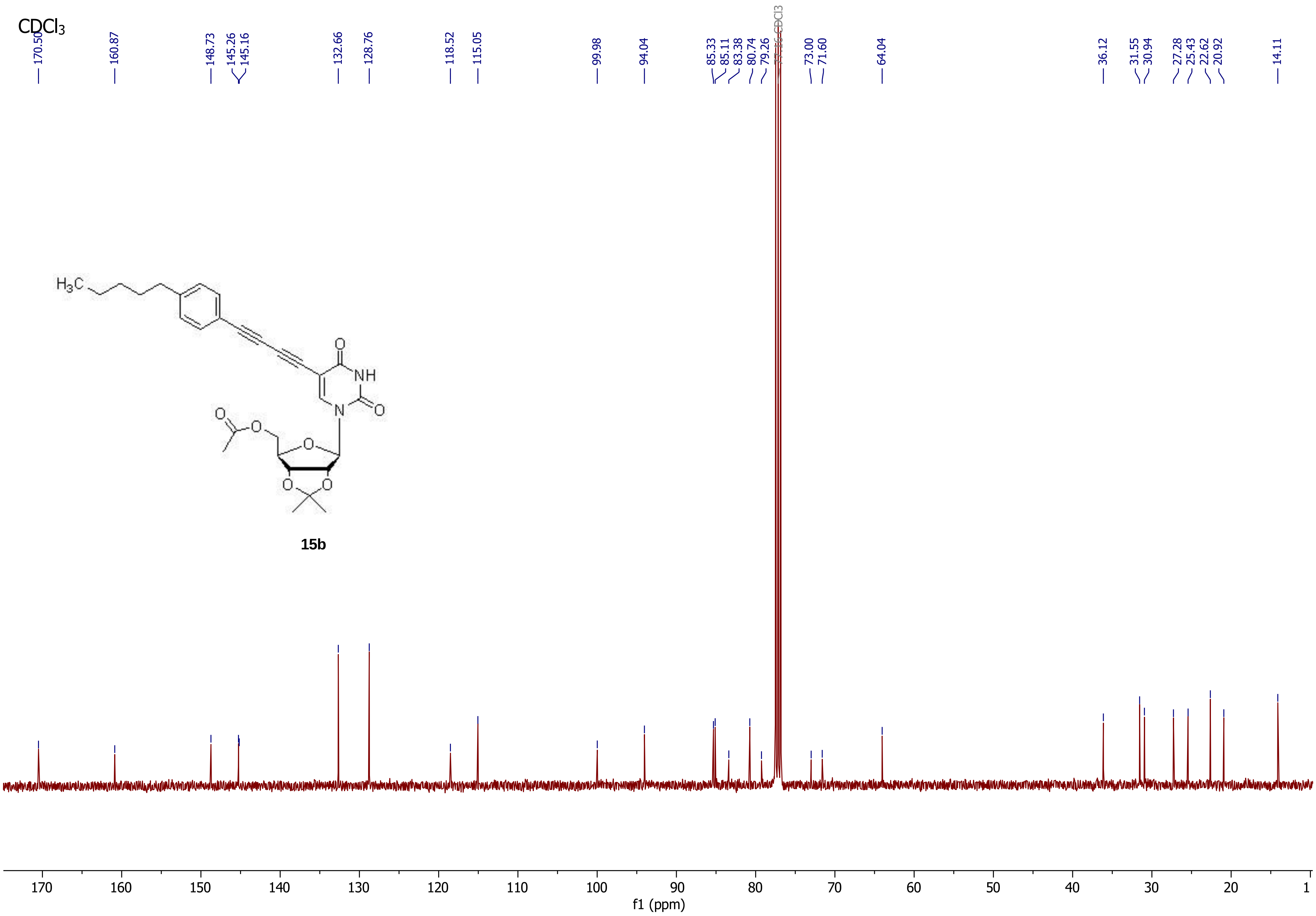
15b
CDCl3
170.50
160.87
148.73
145.26
145.16
132.66
128.76
118.52
115.05
99.98
94.04
85.33
85.11
83.38
80.74
79.26
77.16 CDCl3
73.00
71.60
64.04
36.12
31.55
30.94
27.28
25.43
22.62
20.92
14.11
170 160 150 140 130 120 110 100 90 80 70 60 50 40 30 20 1
f1 (ppm)

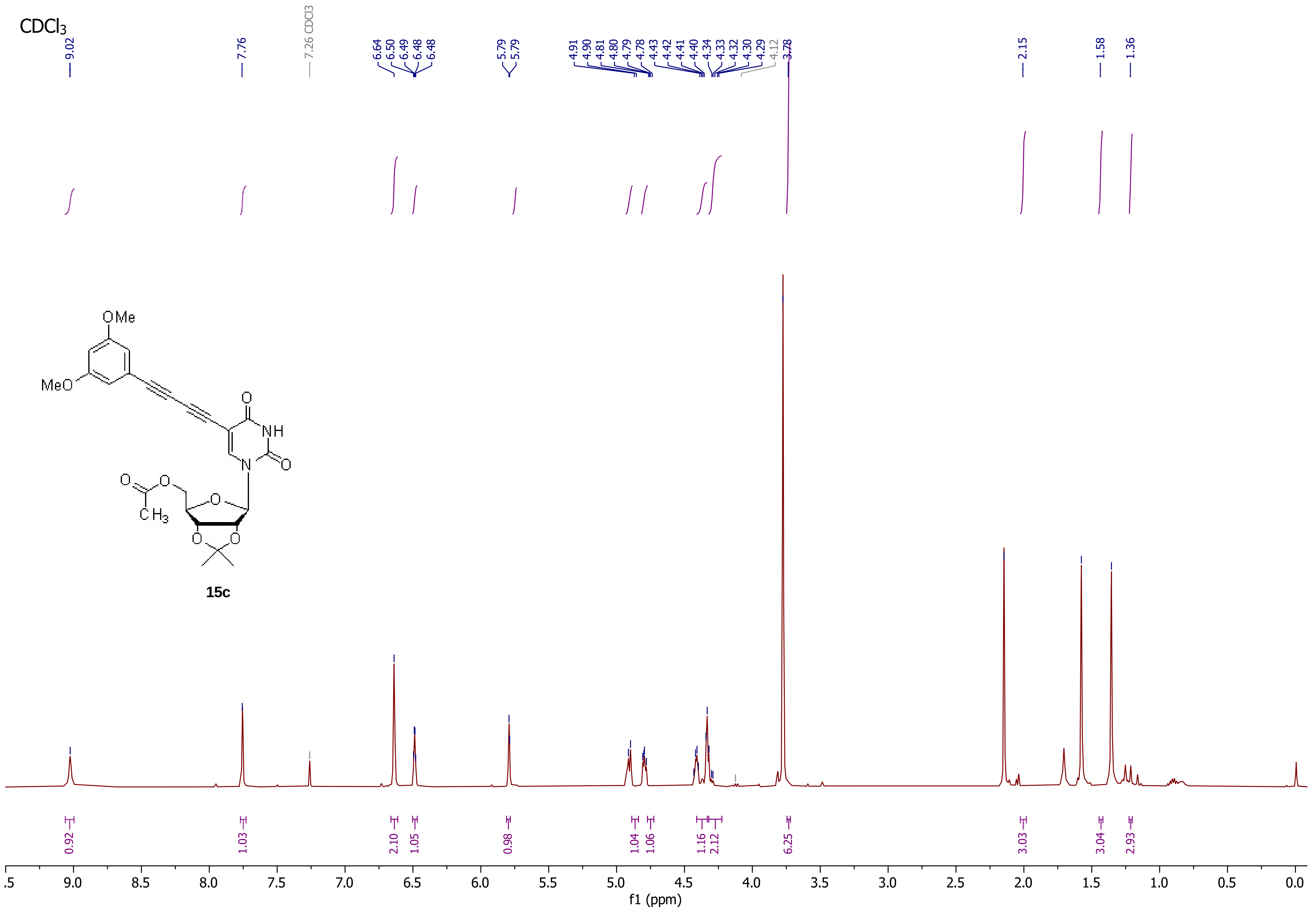
CDCl3
9.02
7.76
7.26 CDCl3
6.64
6.50
6.49
6.48
6.48
5.79
5.79
4.91
4.90
4.81
4.80
4.79
4.78
4.43
4.42
4.41
4.40
4.34
4.33
4.32
4.30
4.29
4.12
3.78
2.15
1.58
1.36
OMe
MeO
O
NH
N
O
O
O
CH3
O
O
O
15c
0.92
1.03
2.10
1.05
0.98
1.04
1.06
1.16
2.12
6.25
3.03
3.04
2.93
.5
9.0
8.5
8.0
7.5
7.0
6.5
6.0
5.5
5.0
4.5
4.0
3.5
3.0
2.5
2.0
1.5
1.0
0.5
0.0
f1 (ppm)

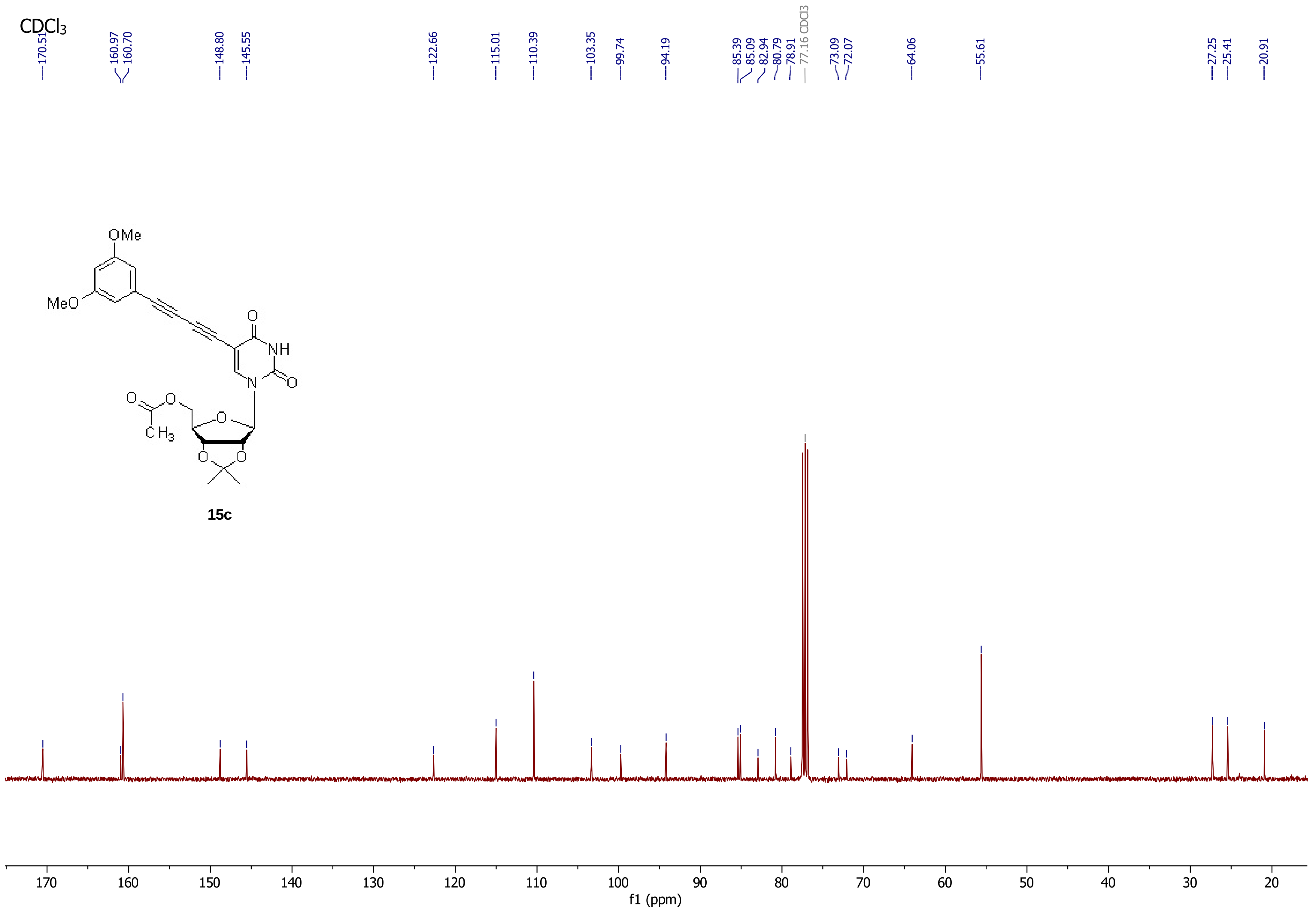
CDCl3
170.51
160.97
160.70
148.80
145.55
122.66
115.01
110.39
103.35
99.74
94.19
85.39
85.09
82.94
80.79
78.91
77.16 CDCl3
73.09
72.07
64.06
55.61
27.25
25.41
20.91
OMe
MeO
NH
N
CH3
15c
170
160
150
140
130
120
110
100
90
80
70
60
50
40
30
20
f1 (ppm)

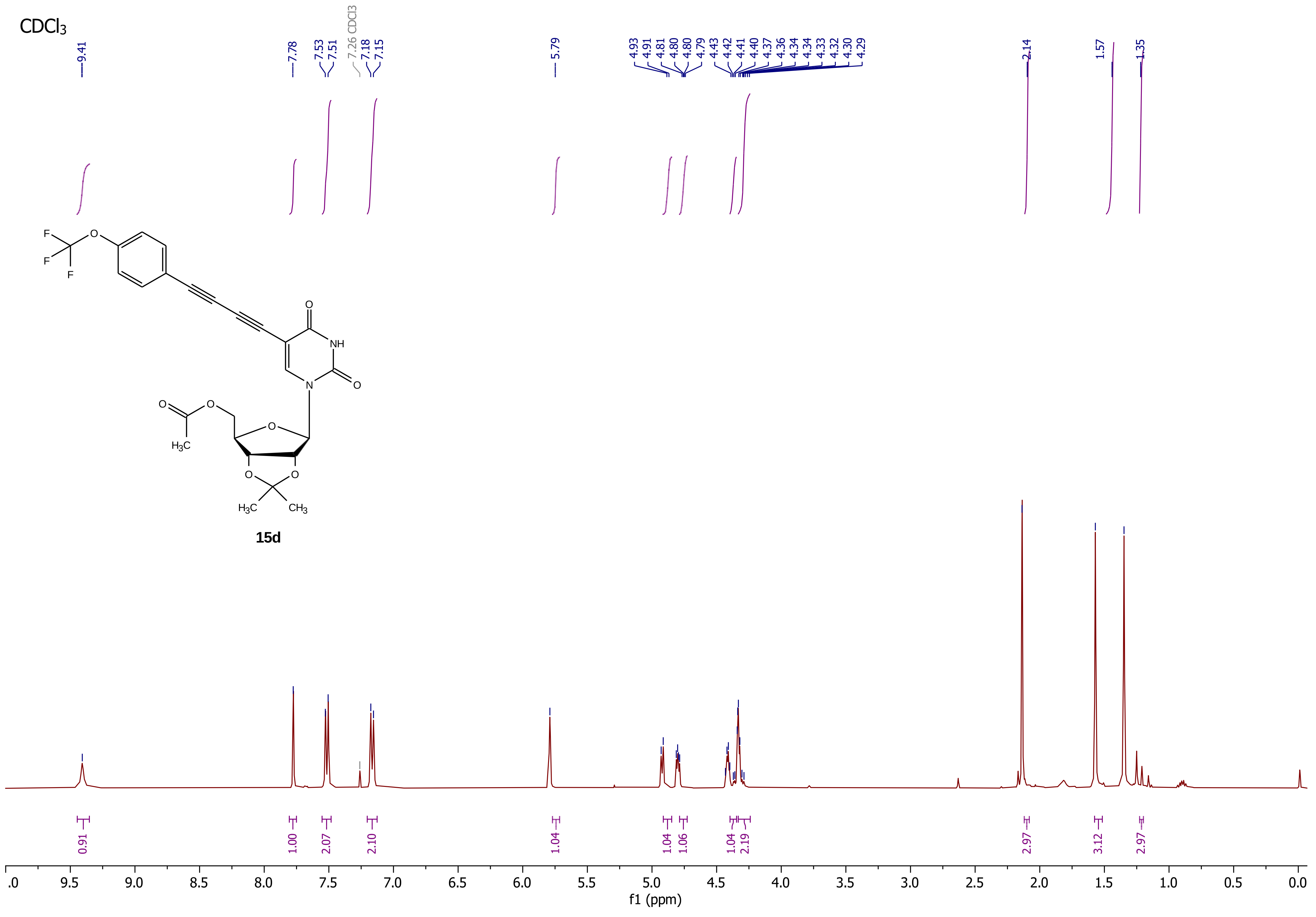
CDCl3
9.41
7.78
7.53
7.51
7.26 CDCl3
7.18
7.15
5.79
4.93
4.91
4.81
4.80
4.80
4.79
4.43
4.42
4.41
4.40
4.37
4.36
4.34
4.34
4.33
4.32
4.30
4.29
2.14
1.57
1.35
15d
0.91
1.00
2.07
2.10
1.04
1.04
1.06
1.04
2.19
2.97
3.12
2.97
f1 (ppm)

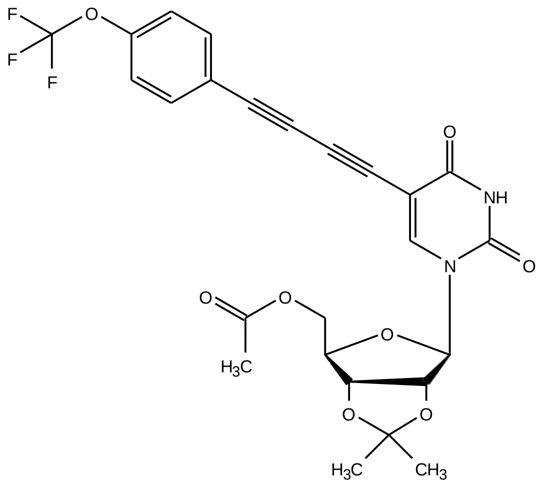

15d

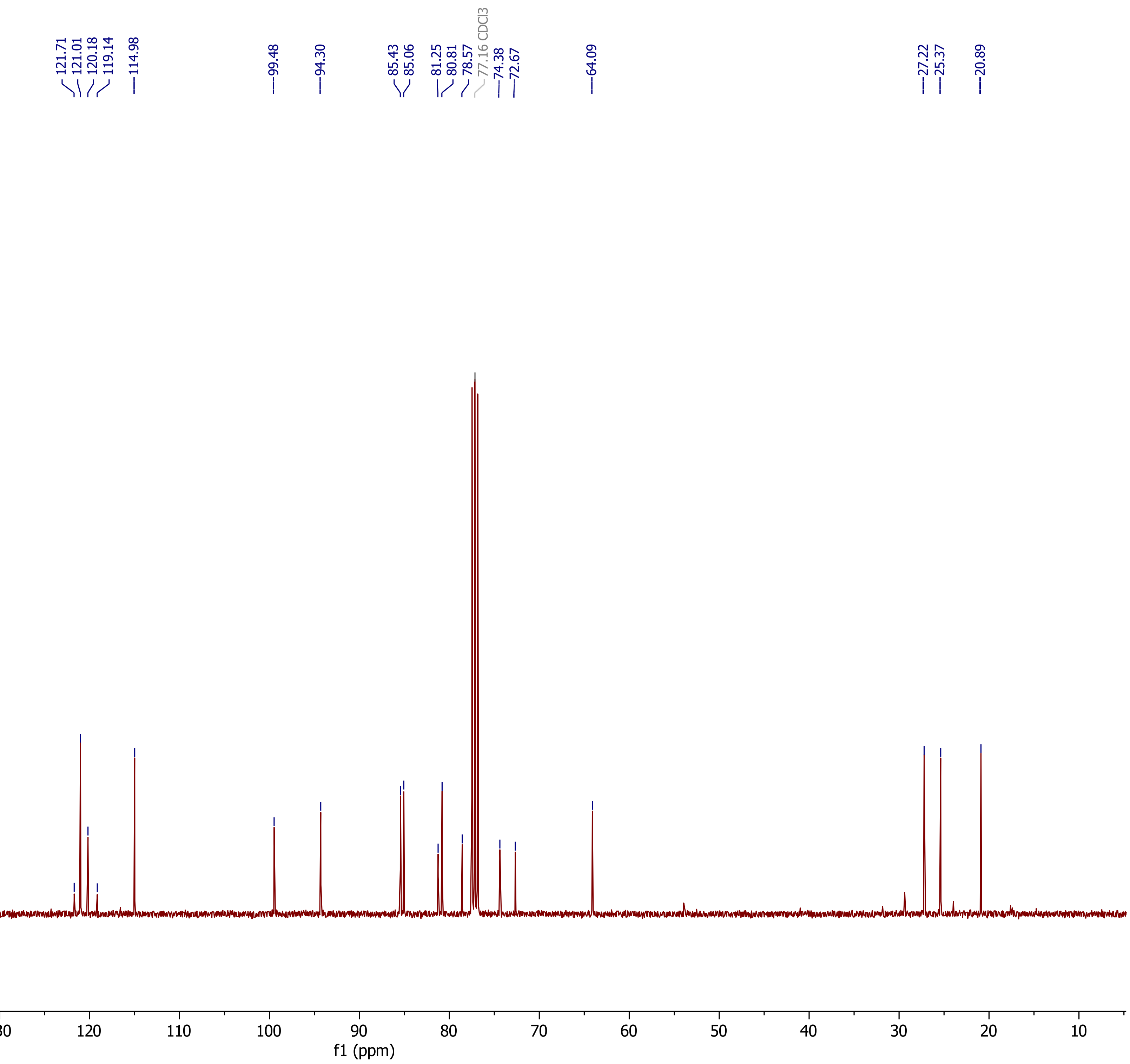
CDCl3
170.54
161.24
149.86
149.84
148.89
145.87
134.29
121.71
121.01
120.18
119.14
114.98
99.48
94.30
85.43
85.06
81.25
80.81
78.57
77.16 CDCl3
74.38
72.67
64.09
27.22
25.37
20.89
170
160
150
140
130
120
110
100
90
80
70
60
50
40
30
20
10
f1 (ppm)

$CDCl_3$

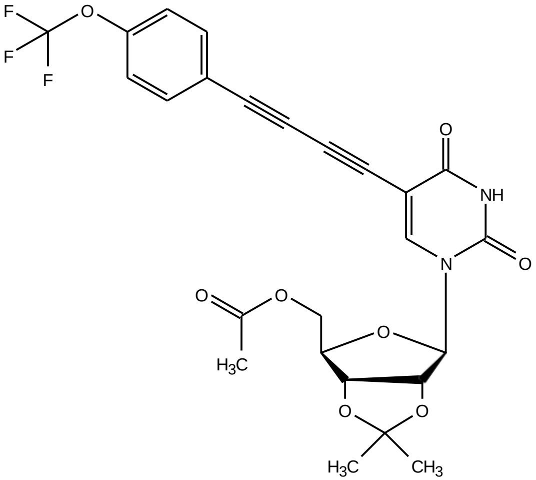

15d

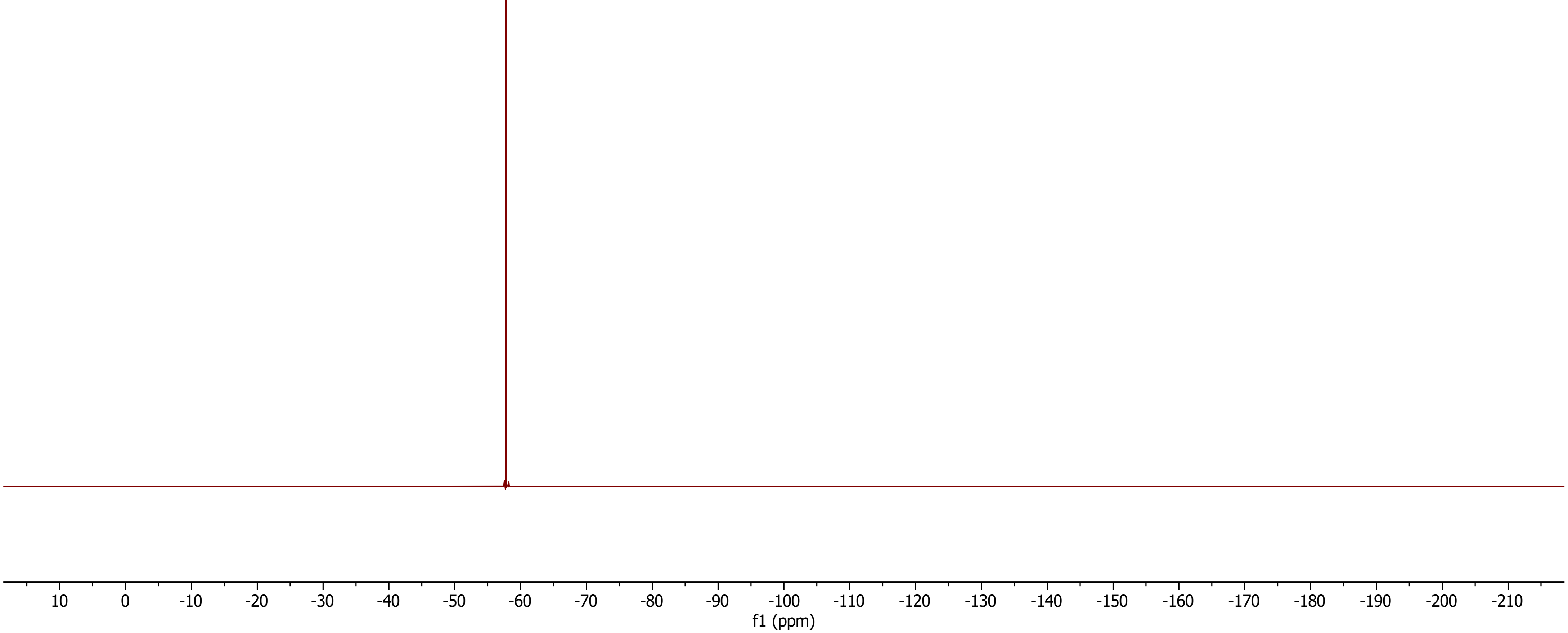

-57.75
10 0 -10 -20 -30 -40 -50 -60 -70 -80 -90 -100 -110 -120 -130 -140 -150 -160 -170 -180 -190 -200 -210
f1 (ppm)

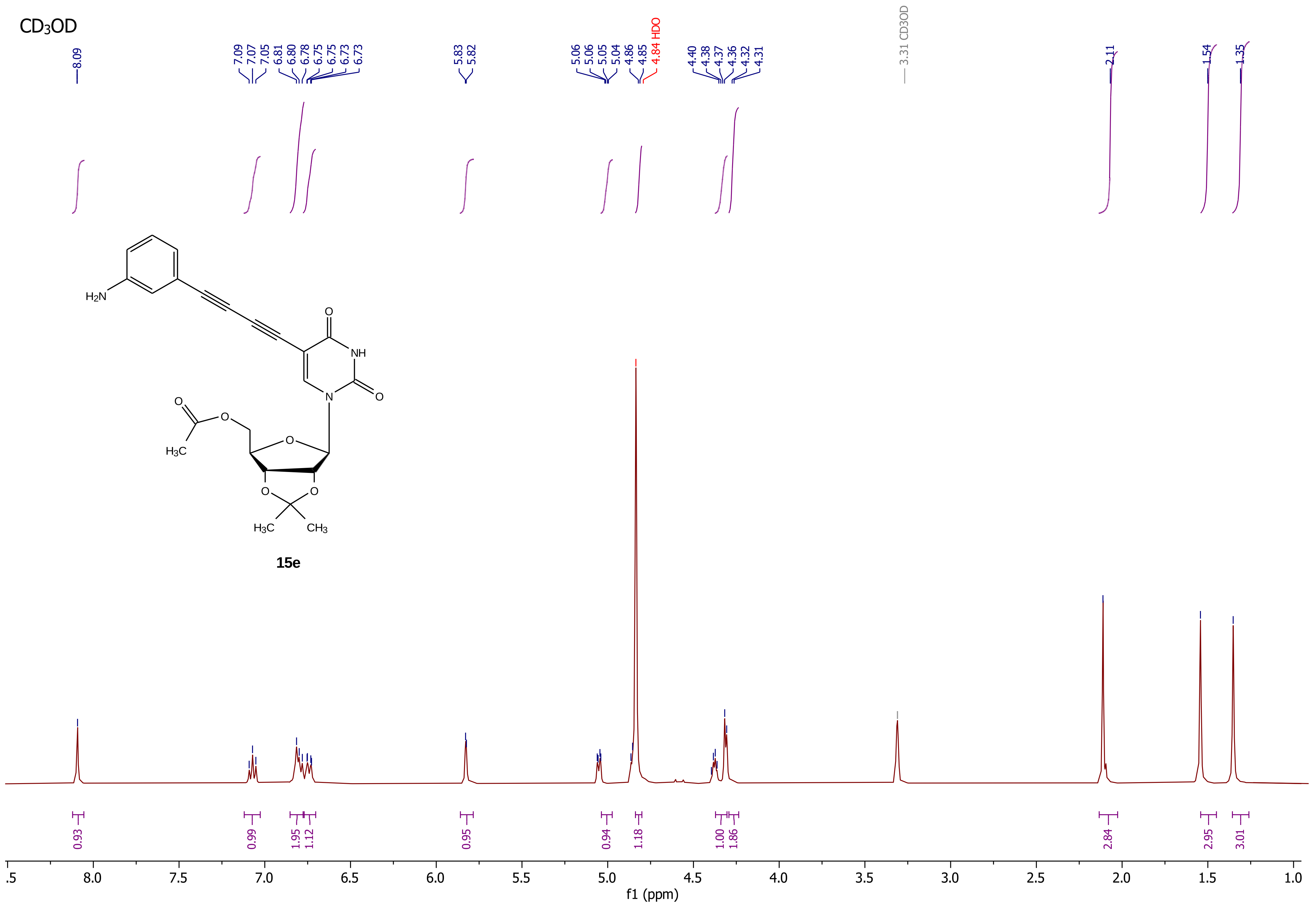
CD3OD
8.09
7.09
7.07
7.05
6.81
6.80
6.78
6.75
6.75
6.73
6.73
5.83
5.82
5.06
5.06
5.05
5.04
4.86
4.85
4.84 HDO
4.40
4.38
4.37
4.36
4.32
4.31
3.31 CD3OD
2.11
1.54
1.35
H2N
NH
O
N
O
O
O
H3C
O
O
O
H3C
CH3
0.93
0.99
1.95
1.12
0.95
0.94
1.18
1.00
1.86
2.84
2.95
3.01
.5
8.0
7.5
7.0
6.5
6.0
5.5
5.0
4.5
4.0
3.5
3.0
2.5
2.0
1.5
1.0
f1 (ppm)
15e

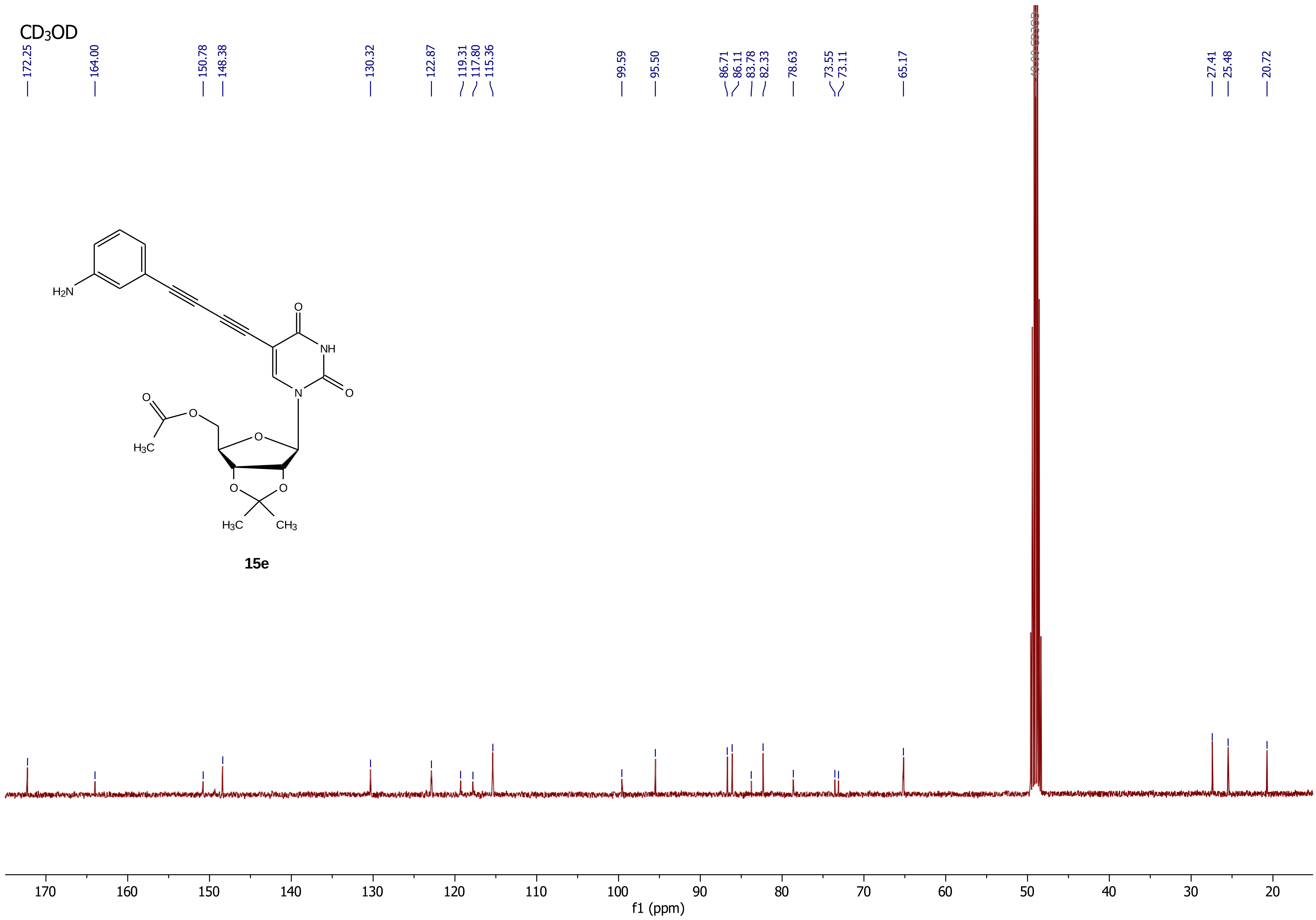
CD3OD
172.25
164.00
150.78
148.38
130.32
122.87
119.31
117.80
115.36
99.59
95.50
86.71
86.11
83.78
82.33
78.63
73.55
73.11
65.17
49.00 CD3OD
27.41
25.48
20.72
H2N
O
NH
N
O
O
O
H3C
O
O
O
H3C
CH3
15e
170
160
150
140
130
120
110
100
90
80
70
60
50
40
30
20
f1 (ppm)

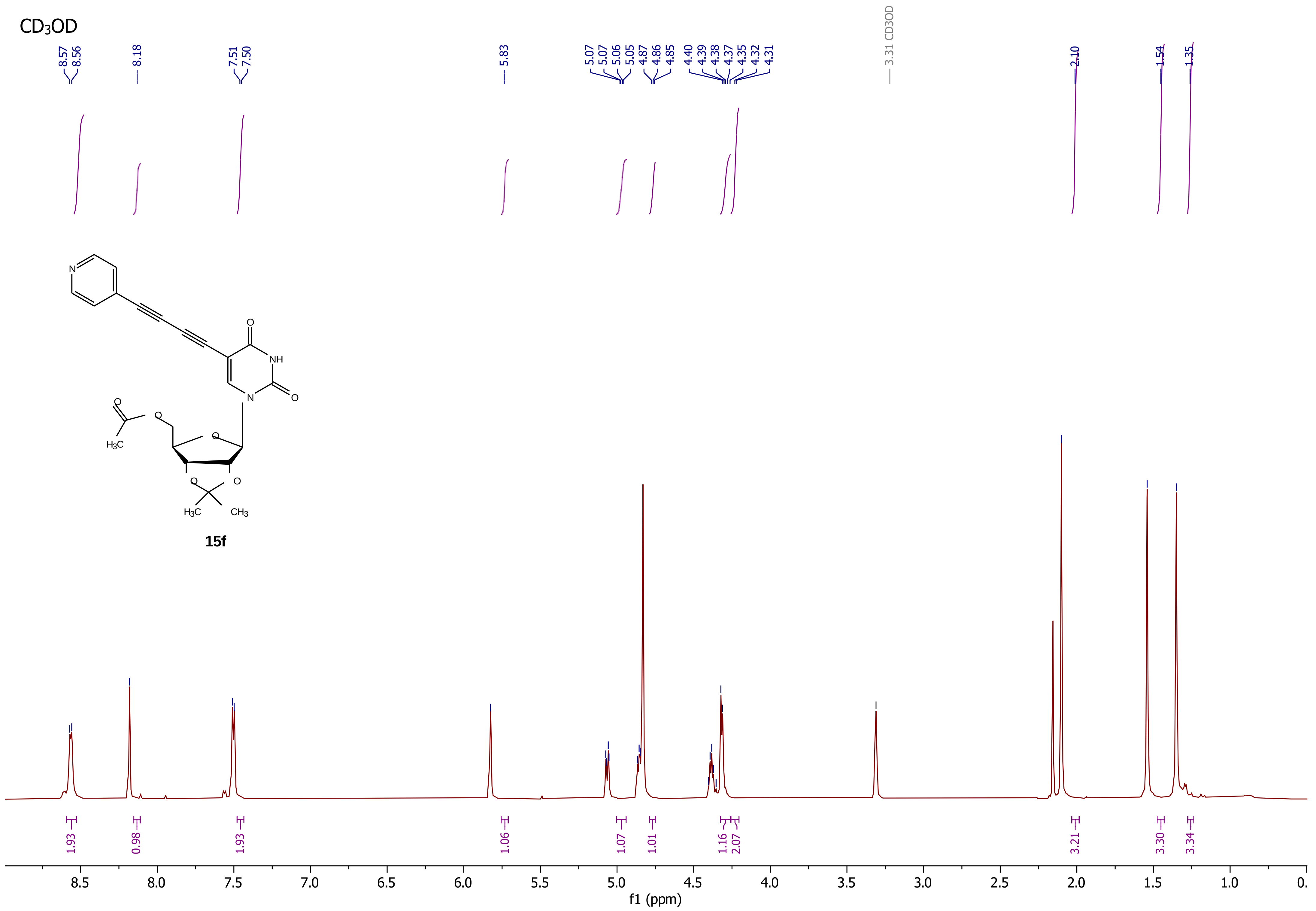

CD3OD
8.57
8.56
8.18
7.51
7.50
5.83
5.07
5.07
5.06
5.05
4.87
4.86
4.85
4.40
4.39
4.38
4.37
4.35
4.32
4.31
3.31 CD3OD
2.10
1.54
1.35
N
NH
O
O
N
O
O
H3C
O
O
H3C
CH3
15f
1.93
0.98
1.93
1.06
1.07
1.01
1.16
2.07
3.21
3.30
3.34
8.5
8.0
7.5
7.0
6.5
6.0
5.5
5.0
4.5
4.0
3.5
3.0
2.5
2.0
1.5
1.0
0.
f1 (ppm)

CD$_3$OD

172.18 163.72 150.70 150.49 149.51 131.96 127.67 115.36 98.68 95.75 86.83 86.13 82.38 79.36 79.05 77.40 77.23 65.18 49.00 CD3OD 27.41 25.47 20.71

22000 21000 20000 19000 18000 17000 16000 15000 14000 13000 12000 11000 10000 9000 8000 7000 6000 5000 4000 3000 2000 1000 0 -1000

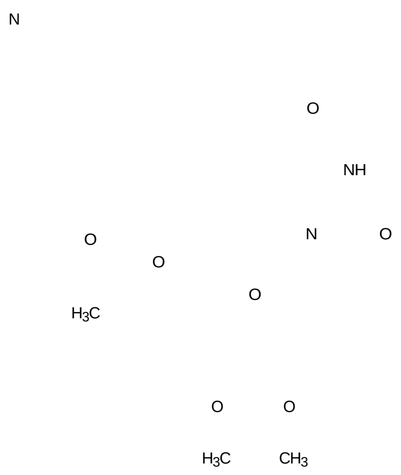

15f

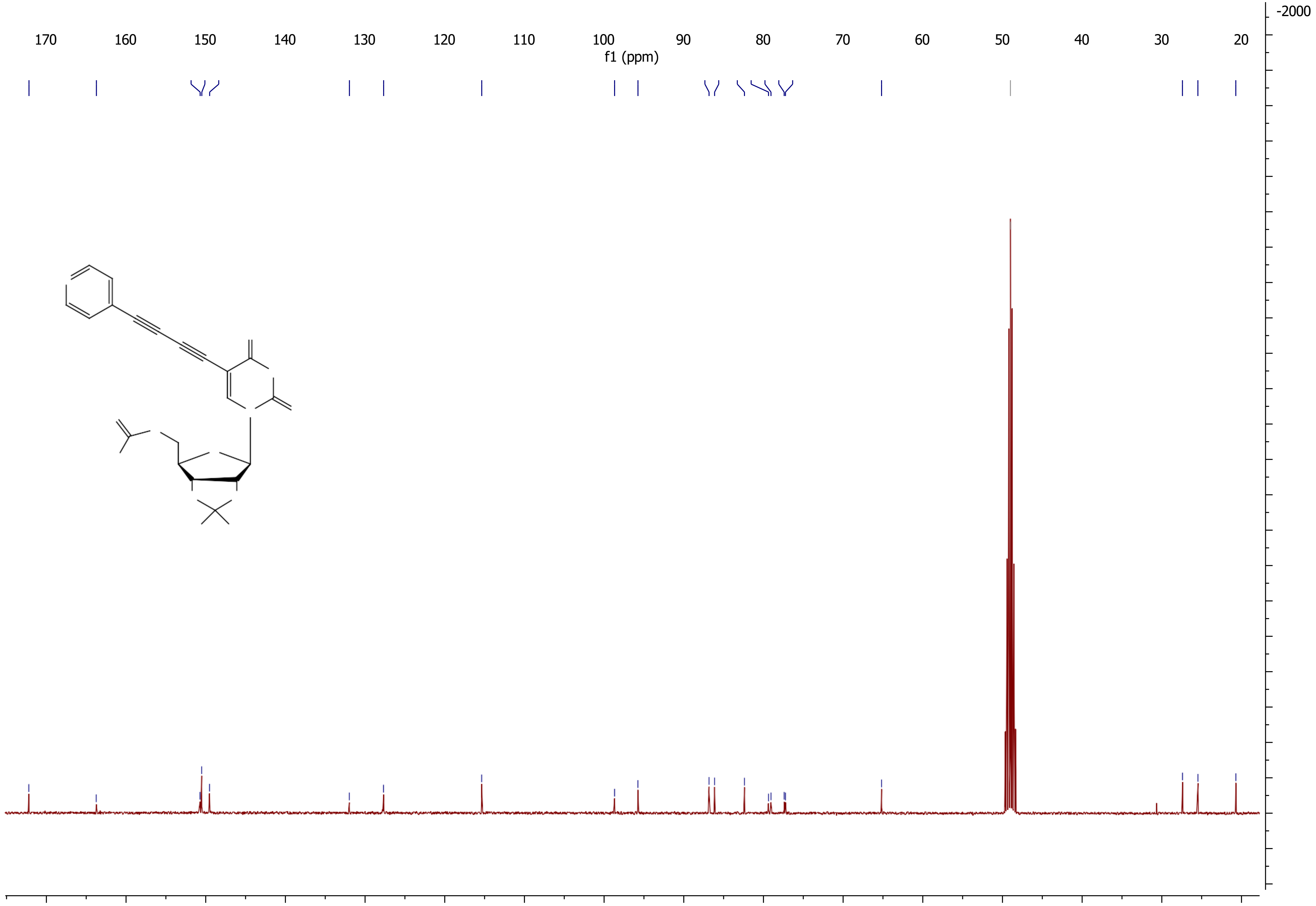
-2000
170 160 150 140 130 120 110 100 90 80 70 60 50 40 30 20
f1 (ppm)

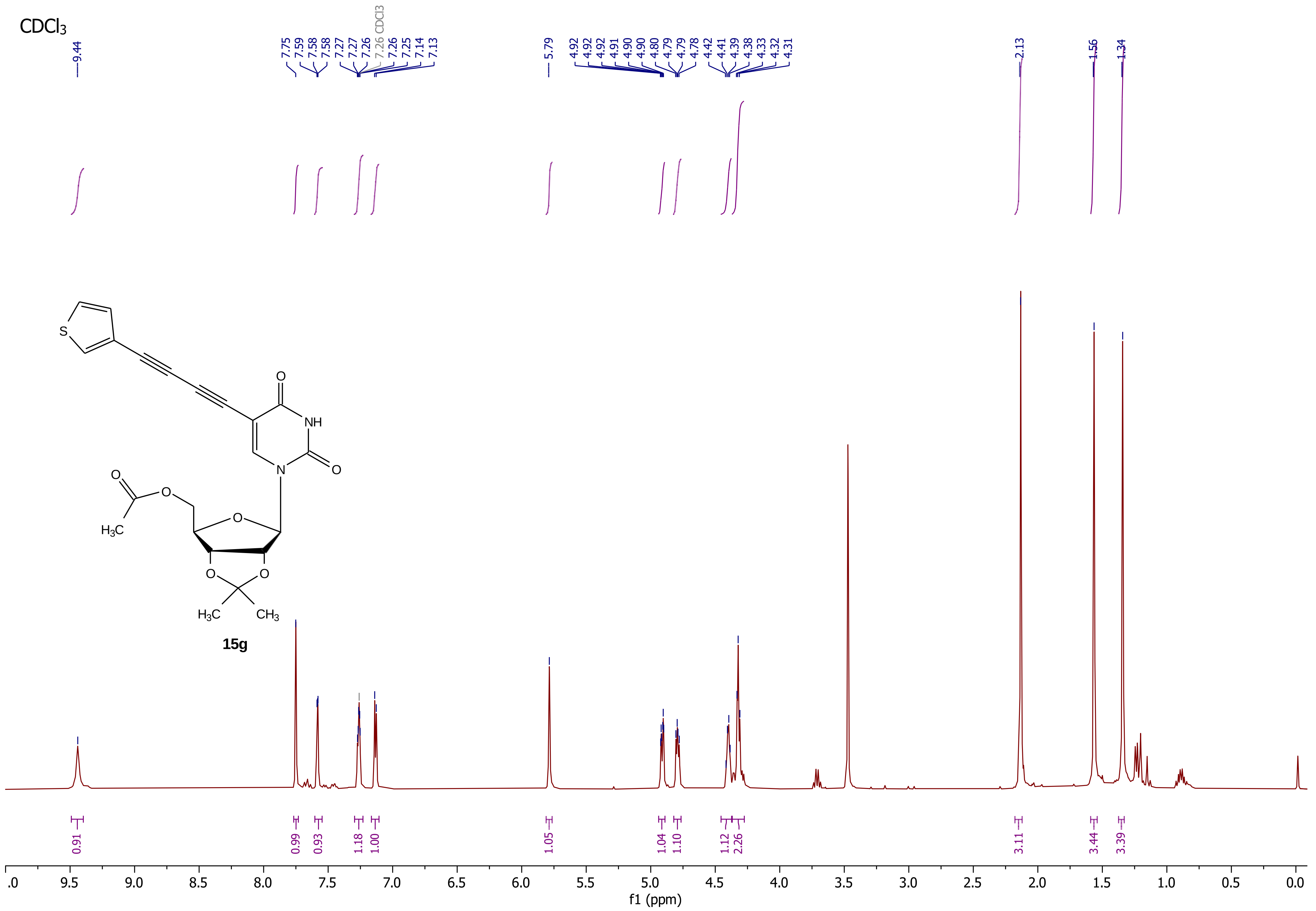
CDCl3
9.44
7.75
7.59
7.58
7.58
7.27
7.27
7.26
7.26 CDCl3
7.26
7.25
7.14
7.13
5.79
4.92
4.92
4.92
4.91
4.90
4.90
4.80
4.79
4.79
4.78
4.42
4.41
4.39
4.38
4.33
4.32
4.31
2.13
1.56
1.34
S
O
NH
O
N
O
O
H3C
O
O
O
H3C
CH3
15g
0.91
0.99
0.93
1.18
1.00
1.05
1.04
1.10
1.12
2.26
3.11
3.44
3.39
.0
9.5
9.0
8.5
8.0
7.5
7.0
6.5
6.0
5.5
5.0
4.5
4.0
3.5
3.0
2.5
2.0
1.5
1.0
0.5
0.0
f1 (ppm)

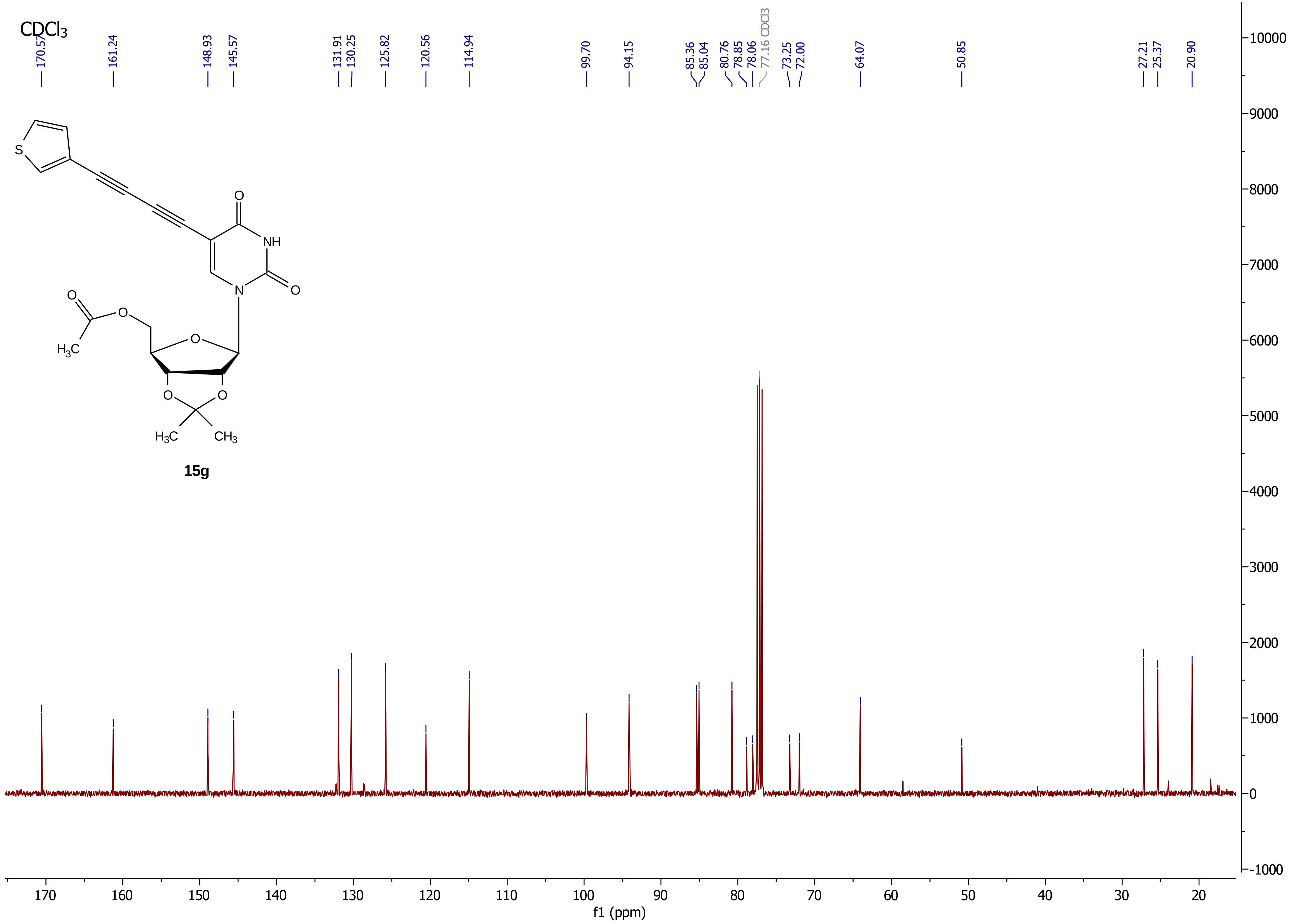
CDCl3
170.57
161.24
148.93
145.57
131.91
130.25
125.82
120.56
114.94
99.70
94.15
85.36
85.04
80.76
78.85
78.06
77.16 CDCl3
73.25
72.00
64.07
50.85
27.21
25.37
20.90
S
O
NH
N
O
H3C
CH3
f1 (ppm)
15g

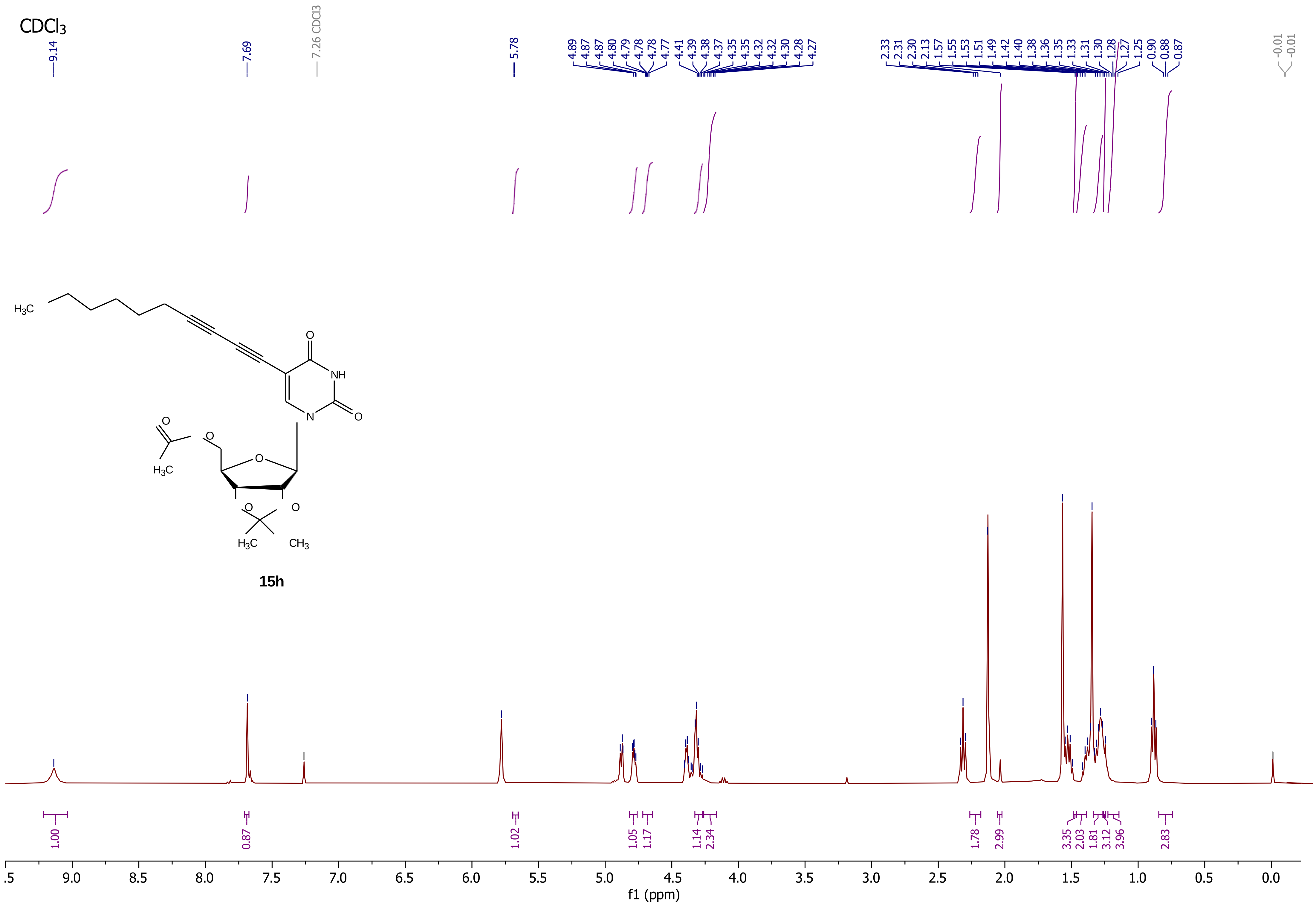
CDCl3
9.14
7.69
7.26 CDCl3
5.78
4.89 4.87 4.87 4.80 4.79 4.78 4.78 4.77 4.41 4.39 4.38 4.37 4.35 4.35 4.32 4.32 4.30 4.28 4.27
2.33 2.31 2.30 2.13 1.57 1.55 1.53 1.51 1.49 1.42 1.40 1.38 1.36 1.35 1.33 1.31 1.30 1.28 1.27 1.25 0.90 0.88 0.87
-0.01 -0.01
H3C
O
NH
N
O
O
O
H3C
O
O
O
H3C
CH3
15h
1.00
0.87
1.02
1.05
1.17
1.14
2.34
1.78
2.99
3.35
2.03
1.81
3.12
3.96
2.83
f1 (ppm)

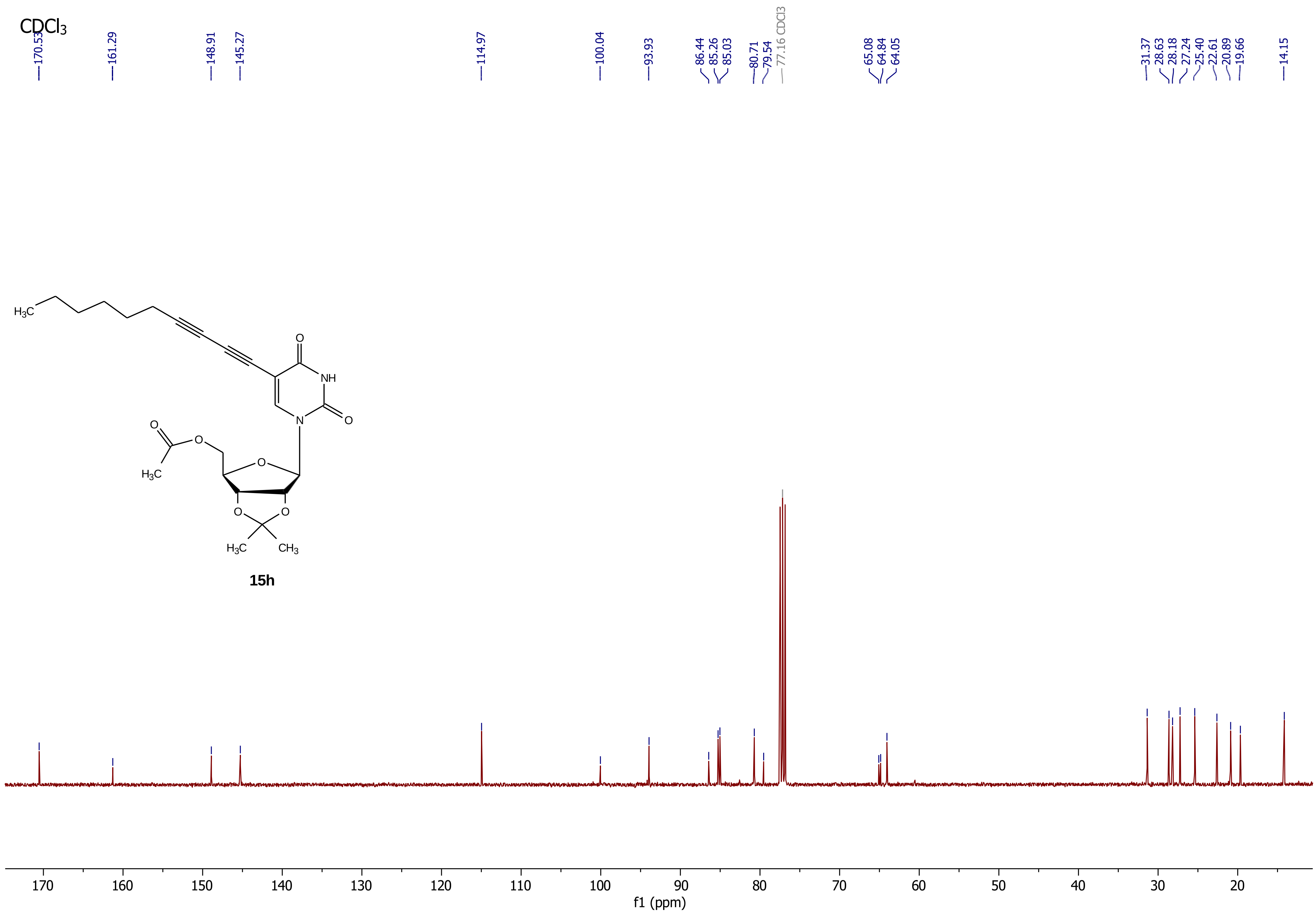

CDCl3
170.53
161.29
148.91
145.27
114.97
100.04
93.93
86.44
85.26
85.03
80.71
79.54
77.16 CDCl3
65.08
64.84
64.05
31.37
28.63
28.18
27.24
25.40
22.61
20.89
19.66
14.15
15h
f1 (ppm)

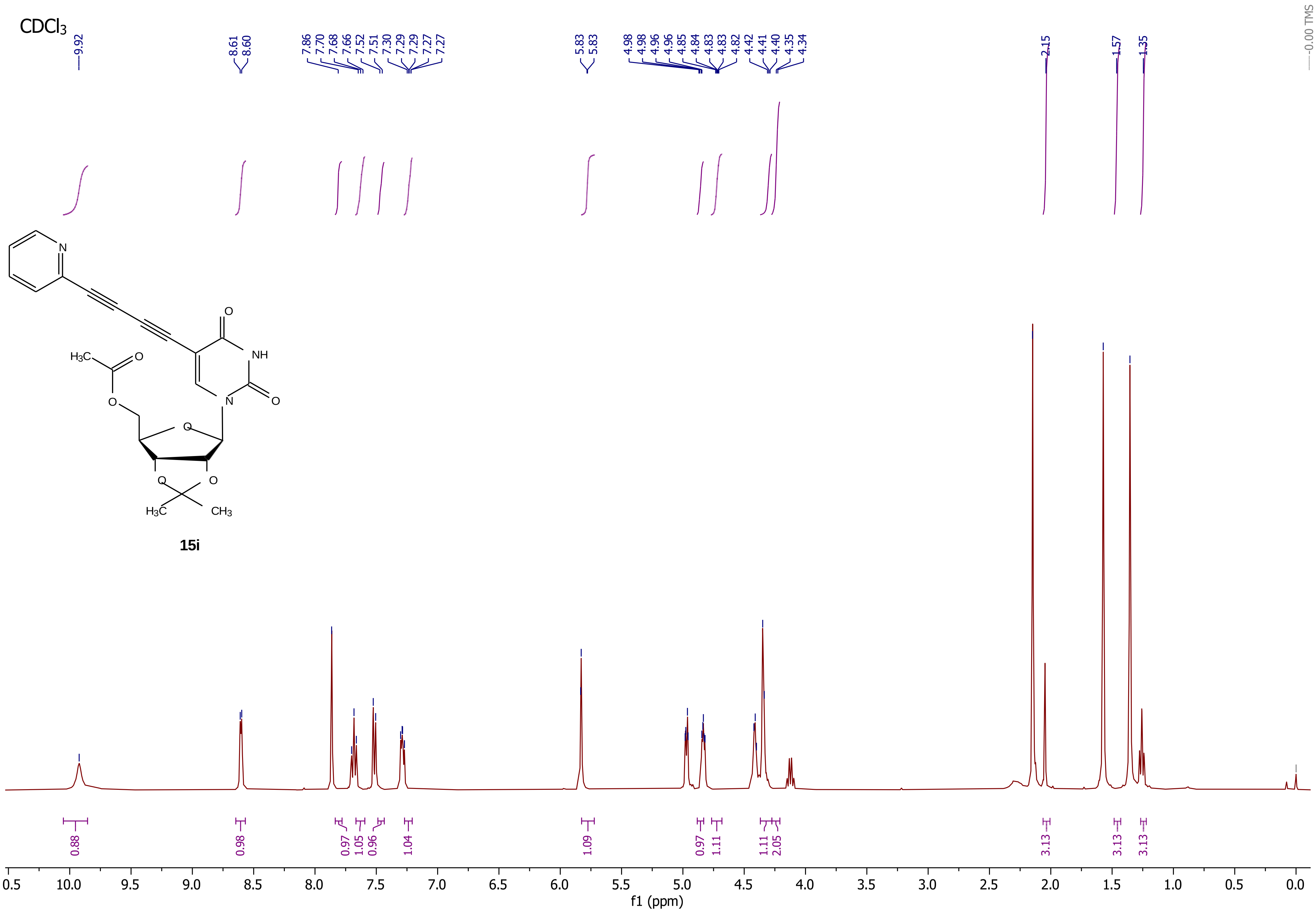

CDCl3
9.92
8.61
8.60
7.86
7.70
7.68
7.66
7.52
7.51
7.30
7.29
7.29
7.27
7.27
5.83
5.83
4.98
4.98
4.96
4.96
4.85
4.84
4.83
4.83
4.82
4.42
4.41
4.40
4.35
4.34
2.15
1.57
1.35
-0.00 TMS
N
O
NH
O
N
H3C
O
O
O
O
O
H3C
CH3
15i
0.88
0.98
0.97
1.05
0.96
1.04
1.09
0.97
1.11
1.11
2.05
3.13
3.13
3.13
f1 (ppm)

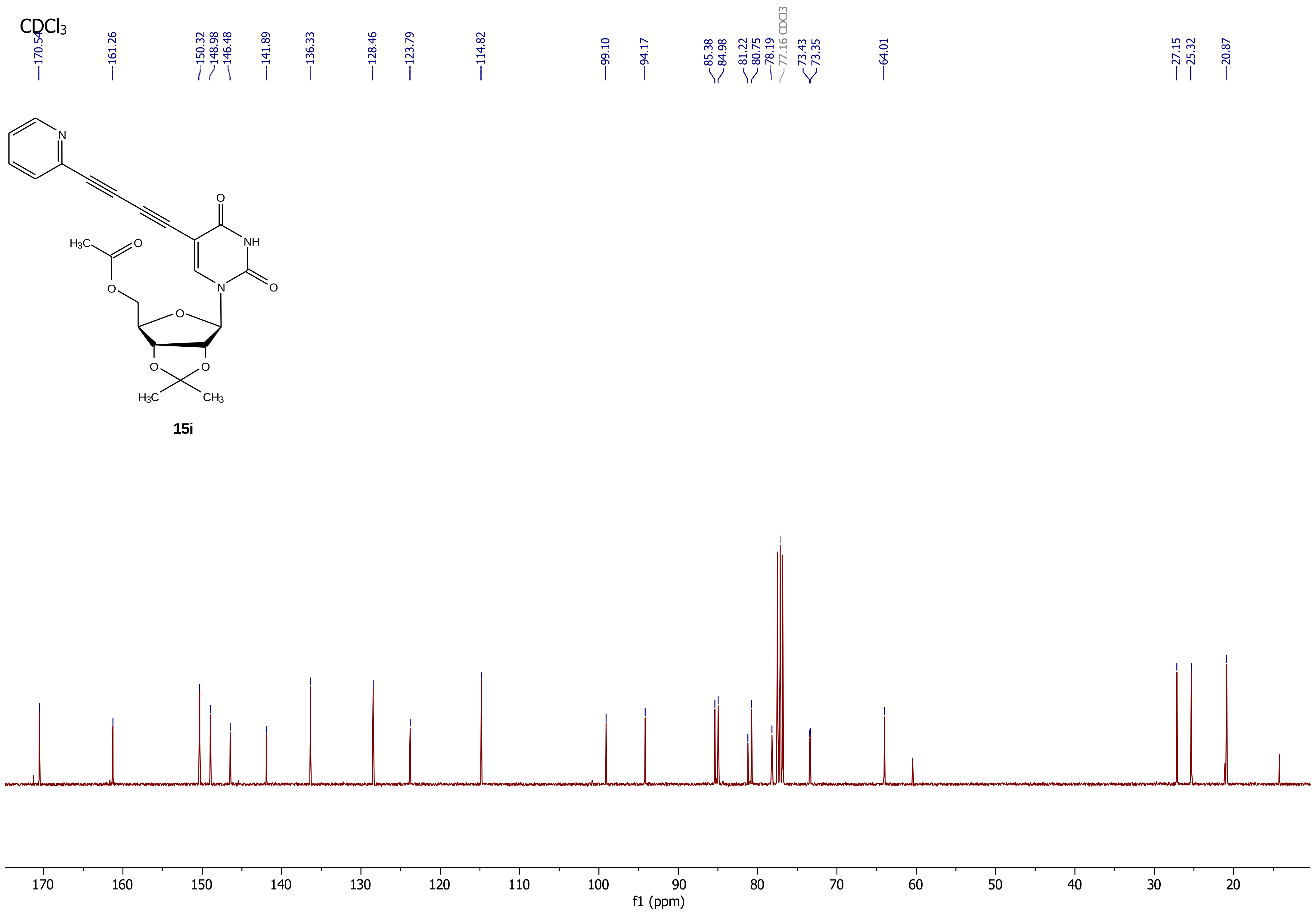
CDCl3
170.54
161.26
150.32
148.98
146.48
141.89
136.33
128.46
123.79
114.82
99.10
94.17
85.38
84.98
81.22
80.75
78.19
77.16 CDCl3
73.43
73.35
64.01
27.15
25.32
20.87
N
O
NH
H3C
O
O
N
O
O
O
H3C
CH3
15i
170
160
150
140
130
120
110
100
90
80
70
60
50
40
30
20
f1 (ppm)

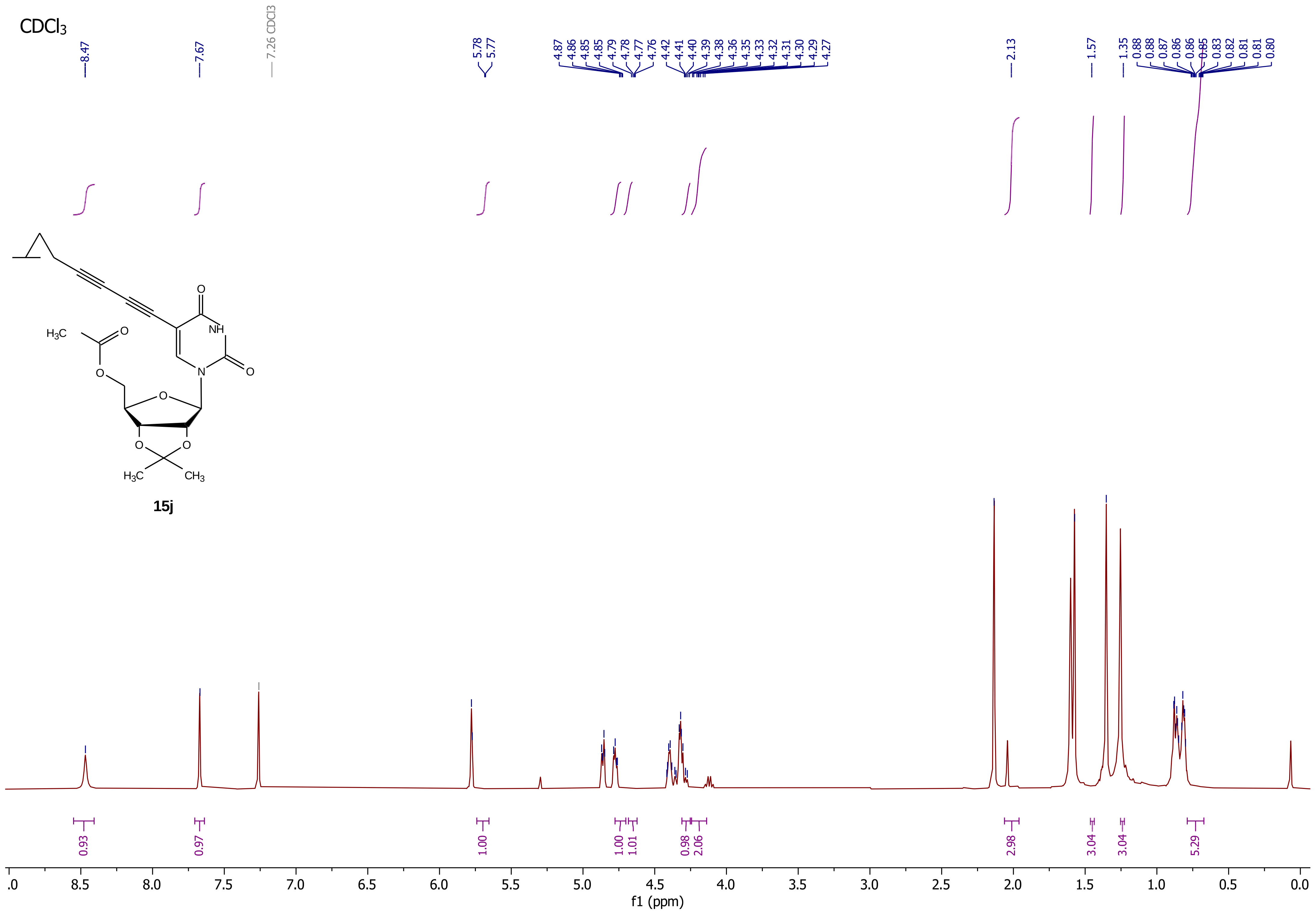

CDCl3
8.47
7.67
7.26 CDCl3
5.78
5.77
4.87
4.86
4.85
4.85
4.79
4.78
4.77
4.76
4.42
4.41
4.40
4.39
4.38
4.36
4.35
4.33
4.32
4.31
4.30
4.29
4.27
2.13
1.57
1.35
0.88
0.88
0.87
0.86
0.86
0.85
0.83
0.82
0.81
0.81
0.80
15j
0.93
0.97
1.00
1.00
1.01
0.98
2.06
2.98
3.04
3.04
5.29
f1 (ppm)

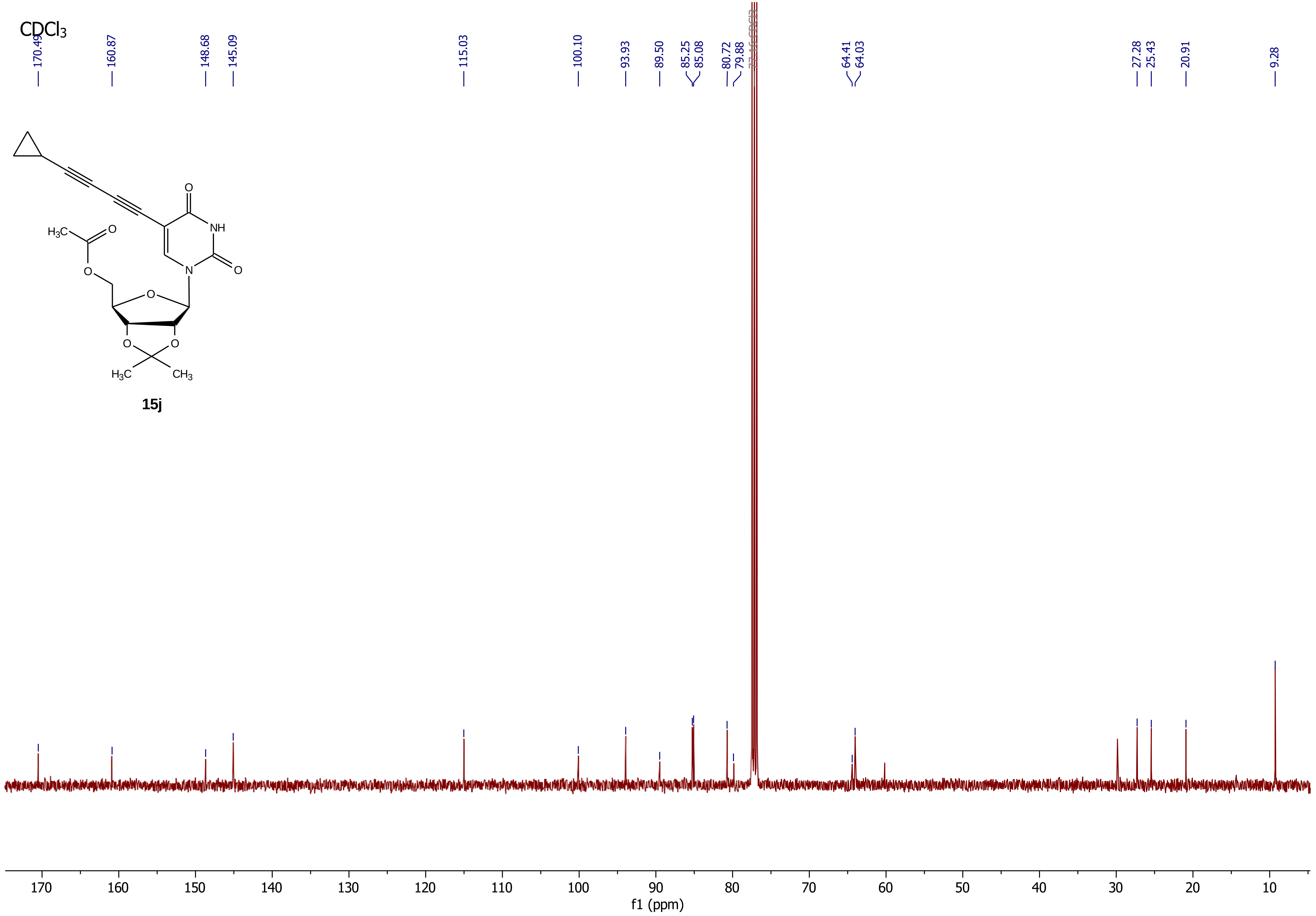
CDCl3
170.49
160.87
148.68
145.09
115.03
100.10
93.93
89.50
85.25
85.08
80.72
79.88
64.41
64.03
27.28
25.43
20.91
9.28
NH
O
N
H3C
CH3
15j
f1 (ppm)
170
160
150
140
130
120
110
100
90
80
70
60
50
40
30
20
10

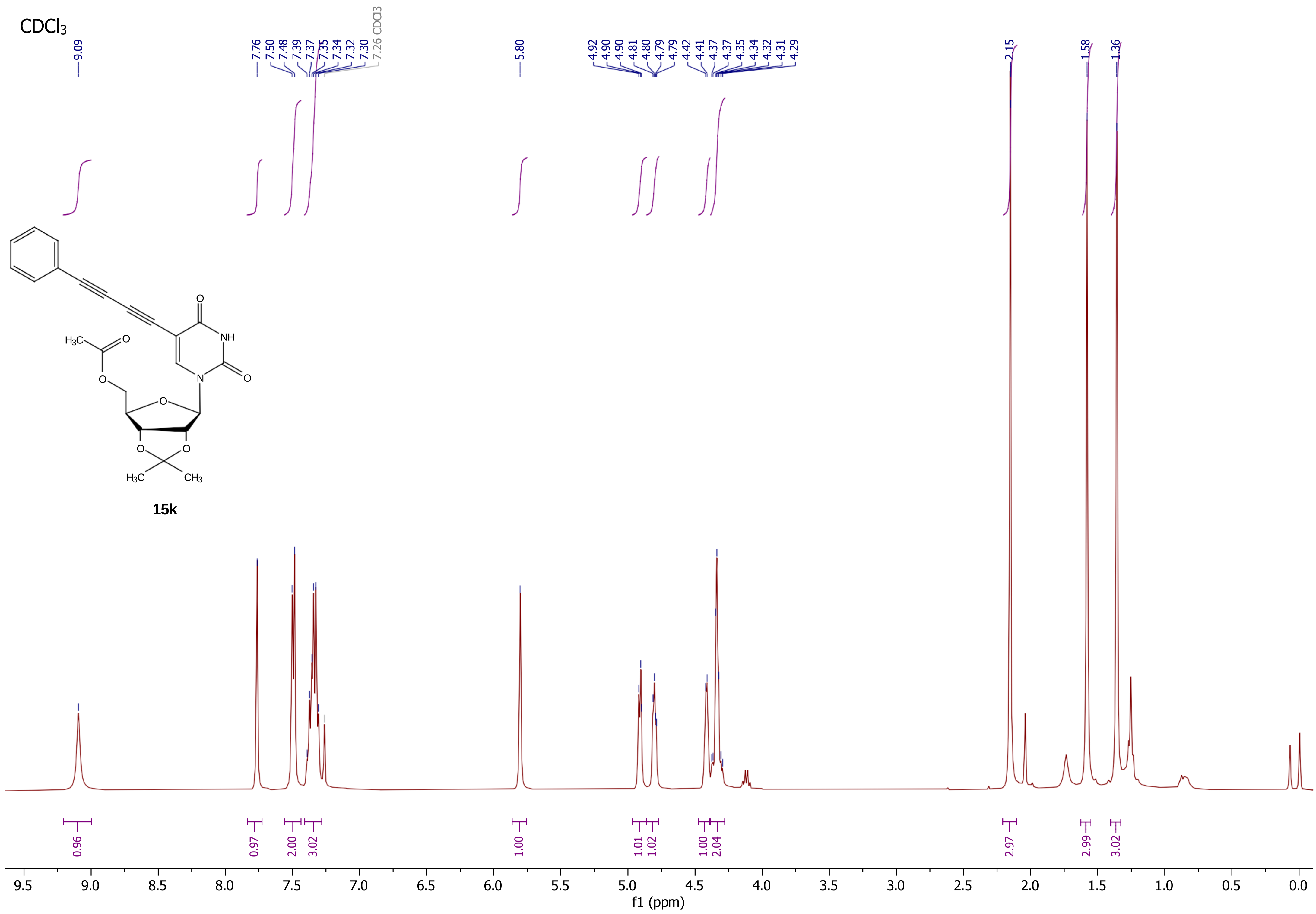
CDCl3
9.09
7.76
7.50
7.48
7.39
7.37
7.35
7.34
7.32
7.30
7.26 CDCl3
5.80
4.92
4.90
4.90
4.81
4.80
4.79
4.79
4.42
4.41
4.37
4.37
4.35
4.34
4.32
4.31
4.29
2.15
1.58
1.36
0.96
0.97
2.00
3.02
1.00
1.01
1.02
1.00
2.04
2.97
2.99
3.02
9.5 9.0 8.5 8.0 7.5 7.0 6.5 6.0 5.5 5.0 4.5 4.0 3.5 3.0 2.5 2.0 1.5 1.0 0.5 0.0
f1 (ppm)
O
NH
O
N
H3C
O
O
O
O
O
H3C
CH3

15k

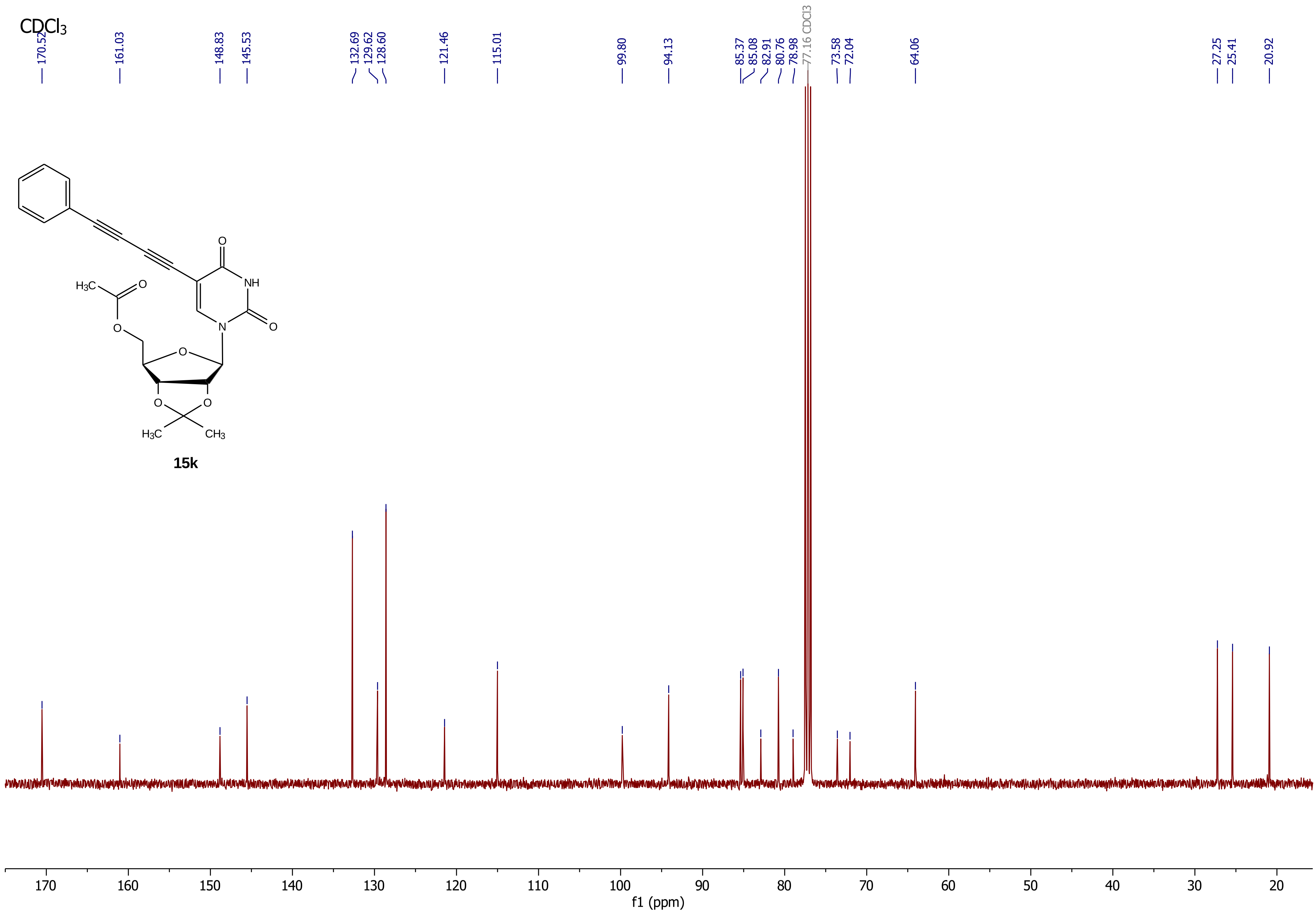
CDCl3
170.52
161.03
148.83
145.53
132.69
129.62
128.60
121.46
115.01
99.80
94.13
85.37
85.08
82.91
80.76
78.98
77.16 CDCl3
73.58
72.04
64.06
27.25
25.41
20.92
15k
170 160 150 140 130 120 110 100 90 80 70 60 50 40 30 20
f1 (ppm)

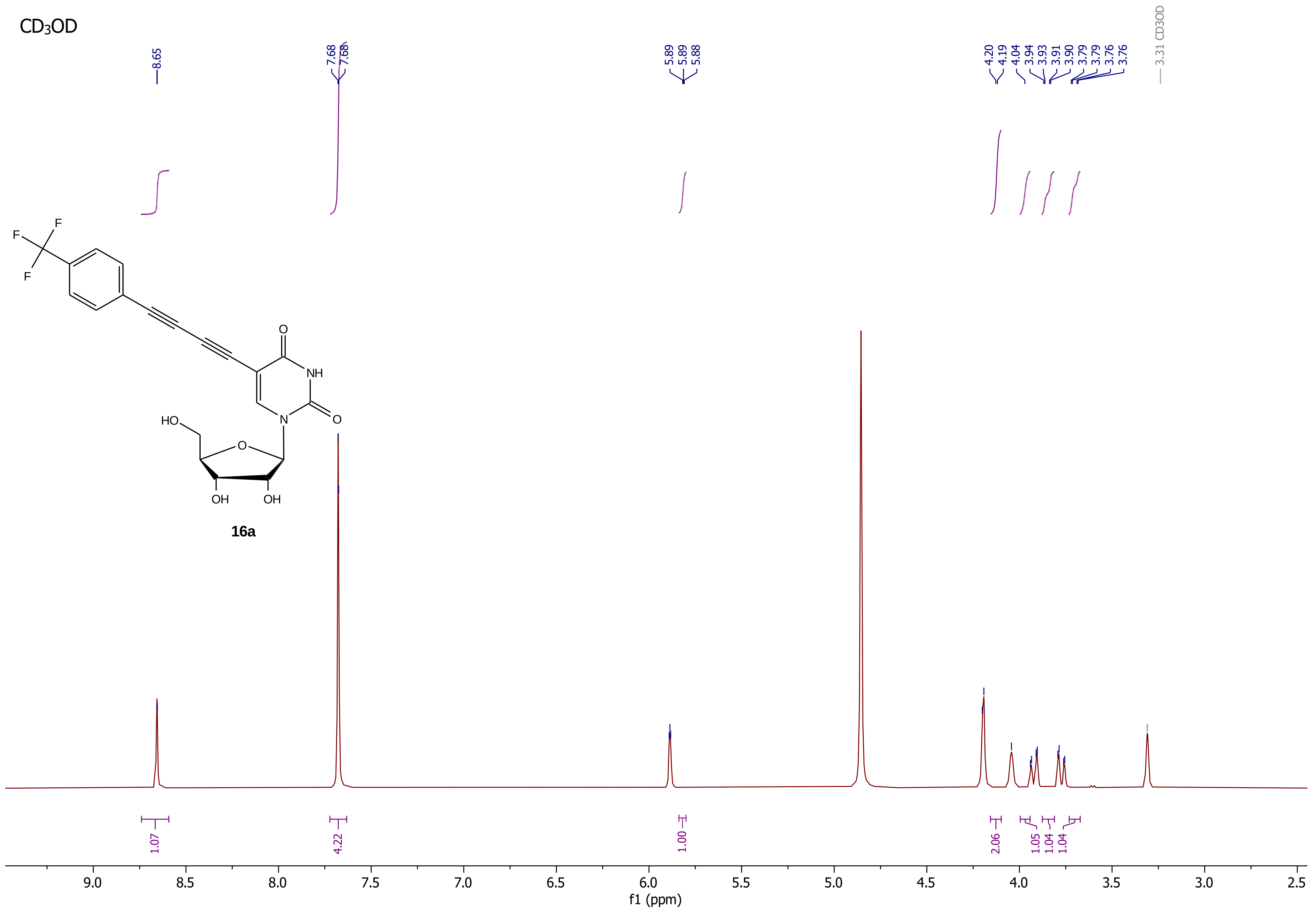
CD3OD
8.65
7.68
7.68
5.89
5.89
5.88
4.20
4.19
4.04
3.94
3.93
3.91
3.90
3.79
3.79
3.76
3.76
3.31 CD3OD
16a
1.07
4.22
1.00
2.06
1.05
1.04
1.04
9.0 8.5 8.0 7.5 7.0 6.5 6.0 5.5 5.0 4.5 4.0 3.5 3.0 2.5
f1 (ppm)

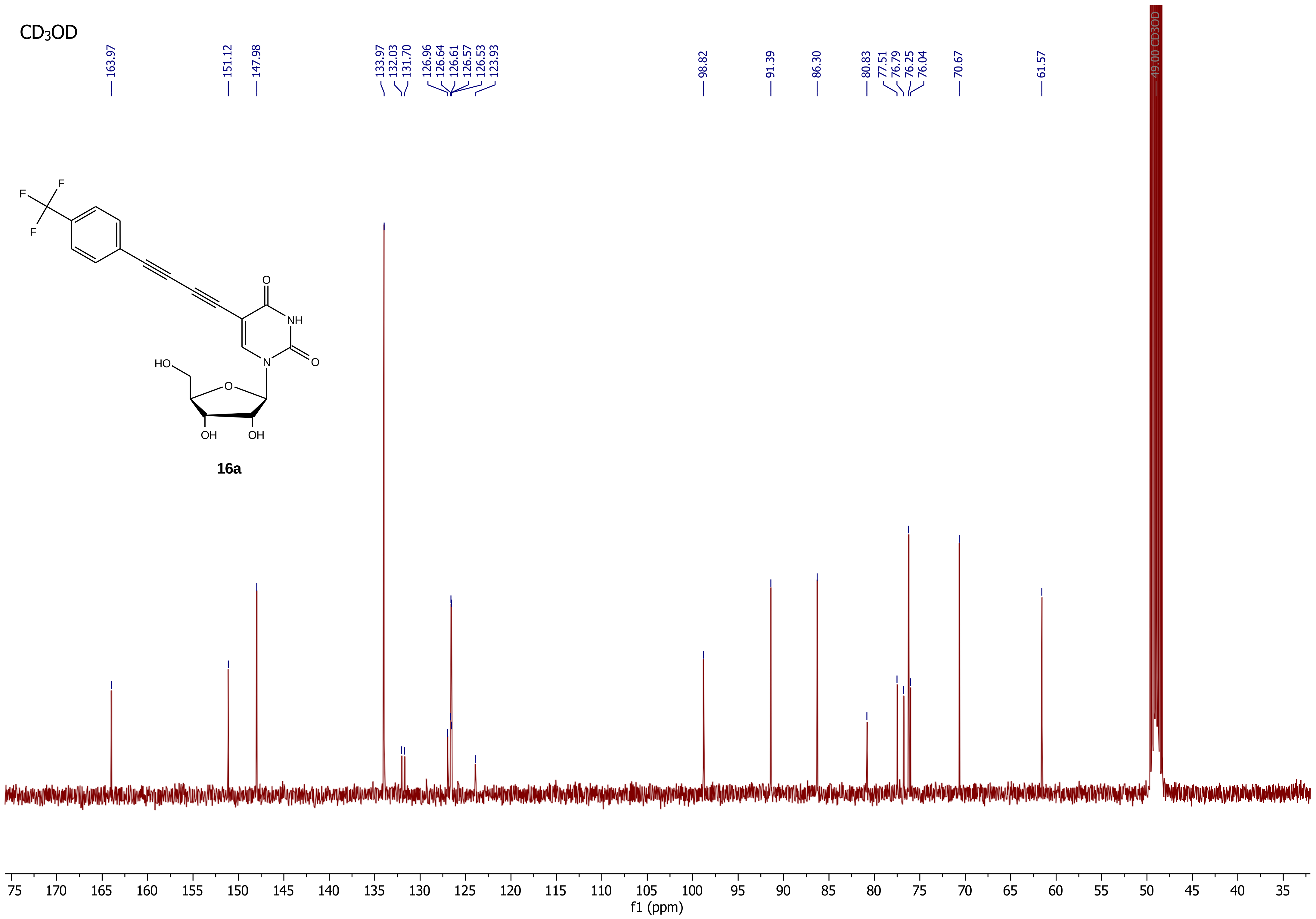
CD3OD
163.97
151.12
147.98
133.97
132.03
131.70
126.96
126.64
126.61
126.57
126.53
123.93
98.82
91.39
86.30
80.83
77.51
76.79
76.25
76.04
70.67
61.57
F
F
F
O
NH
O
N
HO
O
OH
OH
16a
75
170
165
160
155
150
145
140
135
130
125
120
115
110
105
100
95
90
85
80
75
70
65
60
55
50
45
40
35
f1 (ppm)

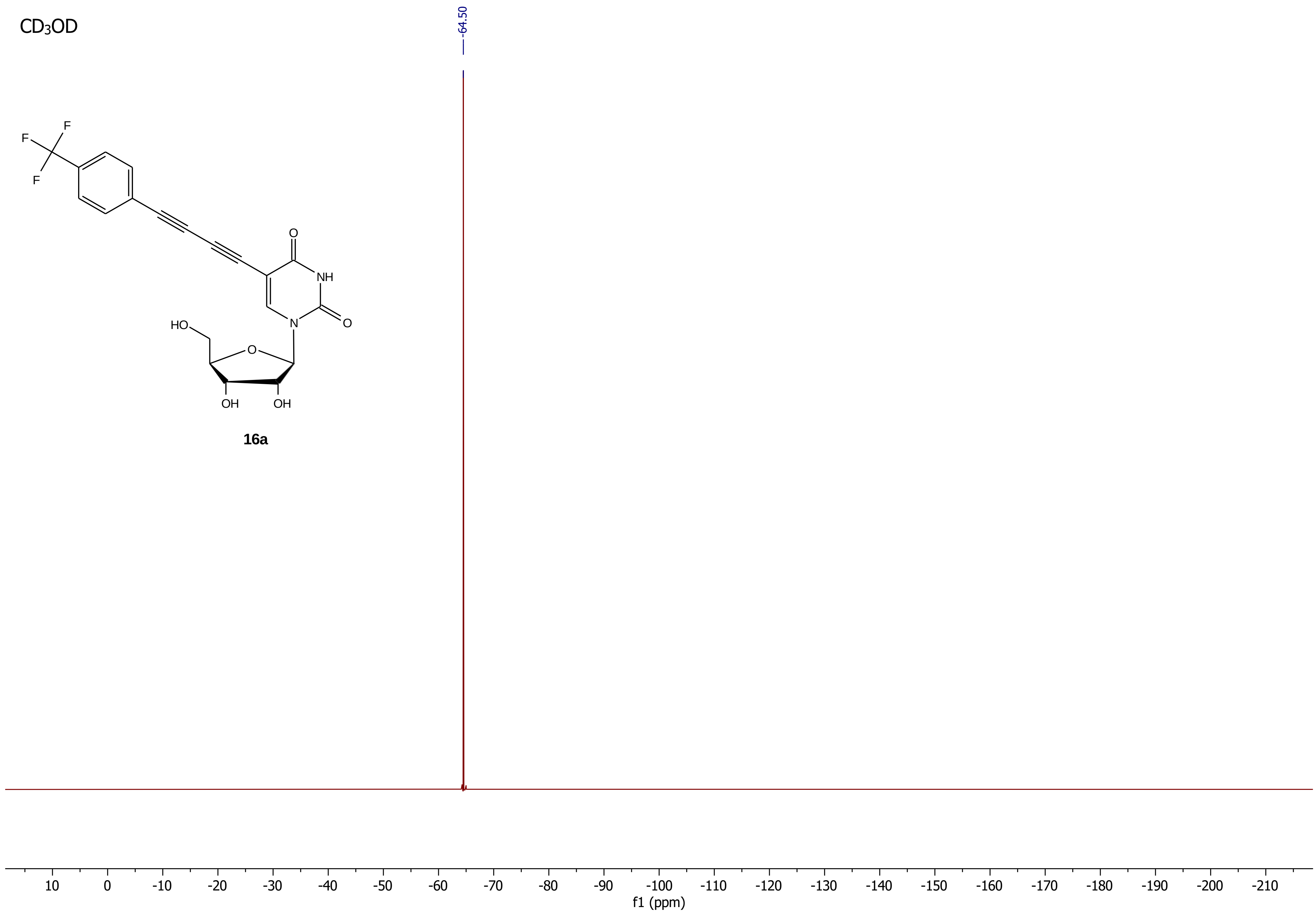
CD3OD
-64.50
F
F
F
O
NH
O
N
HO
O
OH
OH
16a
10 0 -10 -20 -30 -40 -50 -60 -70 -80 -90 -100 -110 -120 -130 -140 -150 -160 -170 -180 -190 -200 -210
f1 (ppm)

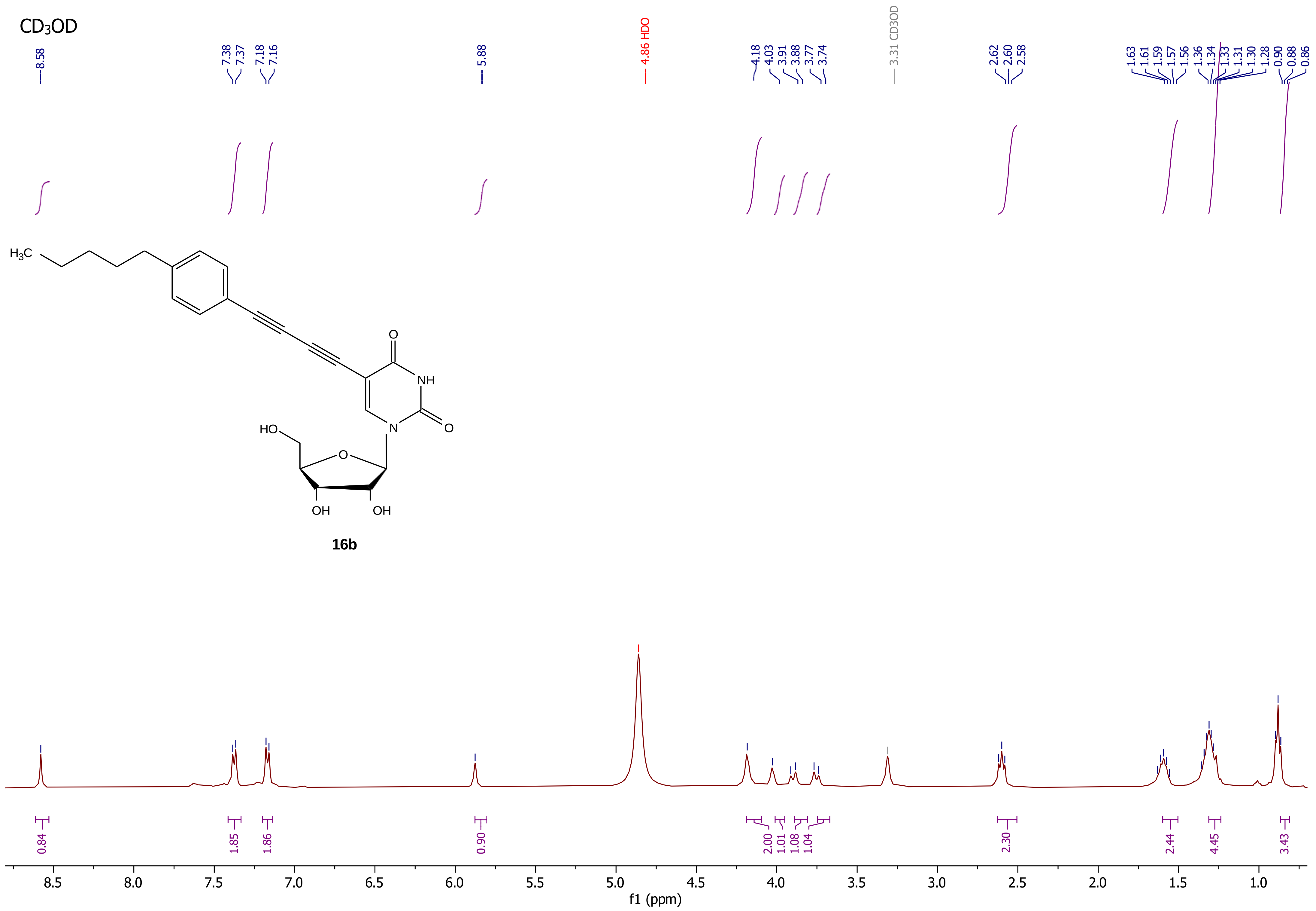
CD3OD
8.58
7.38
7.37
7.18
7.16
5.88
4.86 HDO
4.18
4.03
3.91
3.88
3.77
3.74
3.31 CD3OD
2.62
2.60
2.58
1.63
1.61
1.59
1.57
1.56
1.36
1.34
1.33
1.31
1.30
1.28
0.90
0.88
0.86
H3C
NH
O
O
N
HO
O
OH
OH
16b
0.84
1.85
1.86
0.90
2.00
1.01
1.08
1.04
2.30
2.44
4.45
3.43
8.5
8.0
7.5
7.0
6.5
6.0
5.5
5.0
4.5
4.0
3.5
3.0
2.5
2.0
1.5
1.0
f1 (ppm)

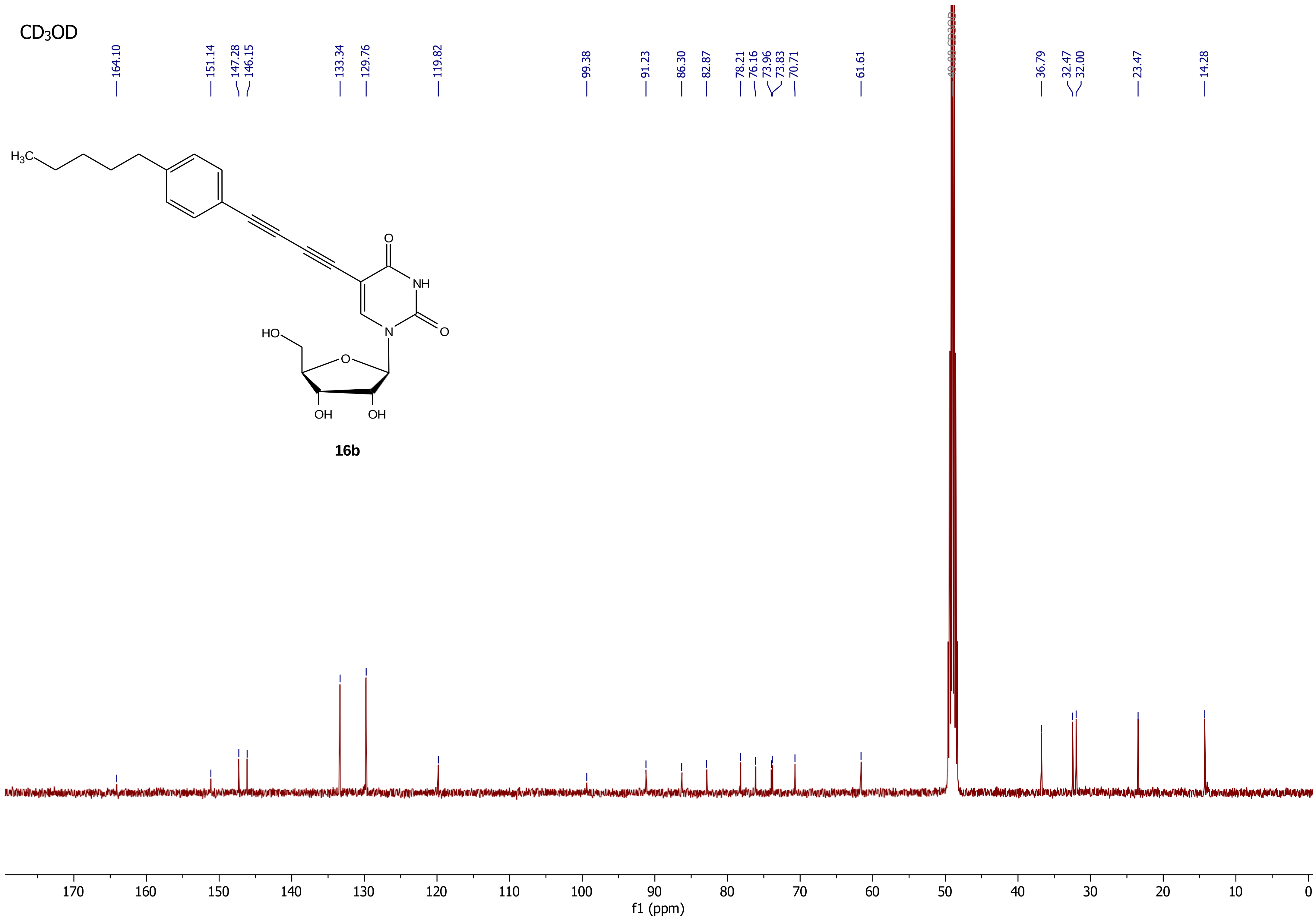
CD3OD
164.10
151.14
147.28
146.15
133.34
129.76
119.82
99.38
91.23
86.30
82.87
78.21
76.16
73.96
73.83
70.71
61.61
36.79
32.47
32.00
23.47
14.28
H3C
O
NH
O
N
HO
O
OH
OH
16b
170
160
150
140
130
120
110
100
90
80
70
60
50
40
30
20
10
0
f1 (ppm)

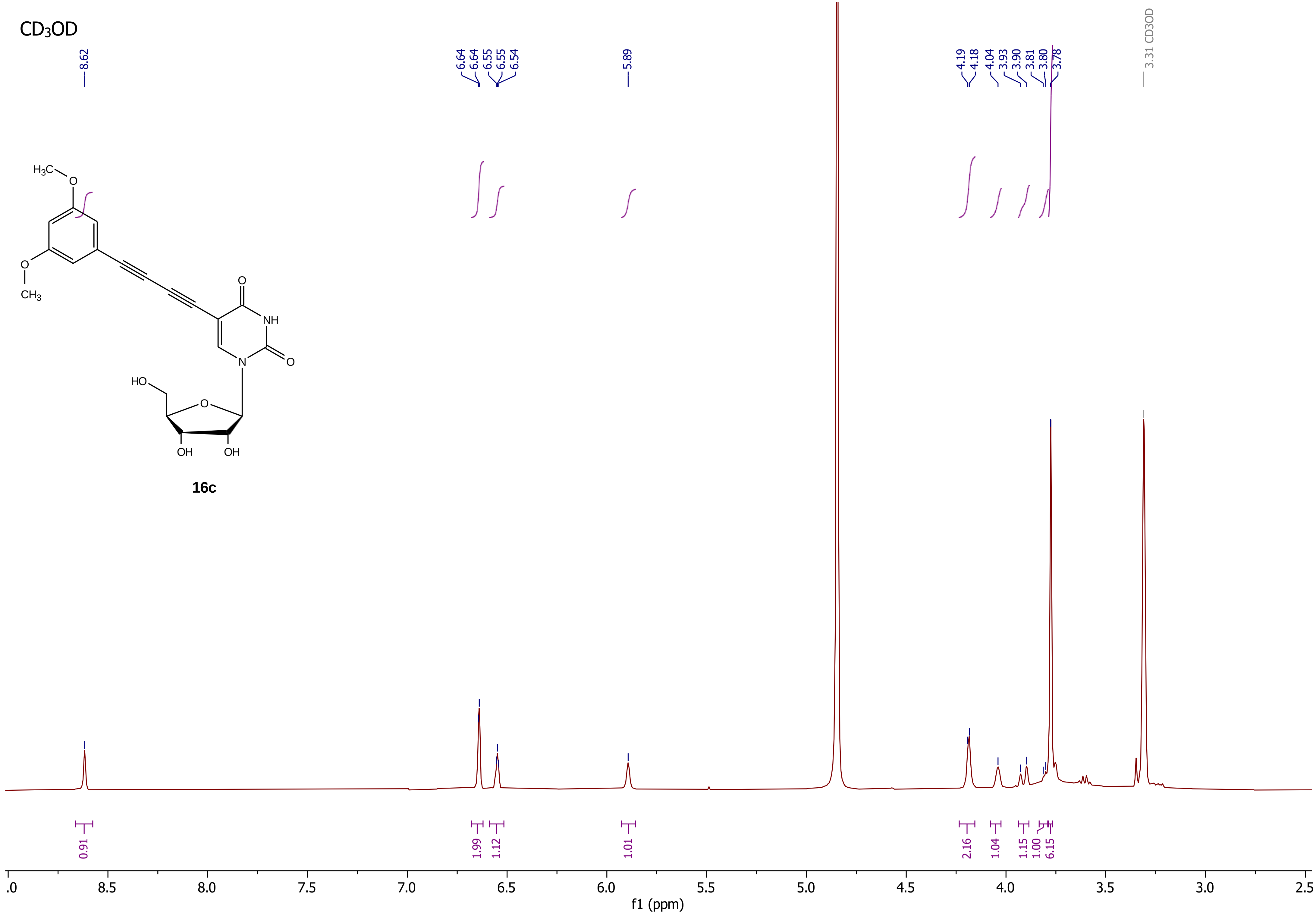

CD3OD
8.62
6.64
6.64
6.55
6.55
6.54
5.89
4.19
4.18
4.04
3.93
3.90
3.81
3.80
3.78
3.31 CD3OD
H3C
O
O
CH3
O
NH
O
N
HO
O
OH
OH
0.91
1.99
1.12
1.01
2.16
1.04
1.15
1.00
6.15
.0
8.5
8.0
7.5
7.0
6.5
6.0
5.5
5.0
4.5
4.0
3.5
3.0
2.5
f1 (ppm)

16c

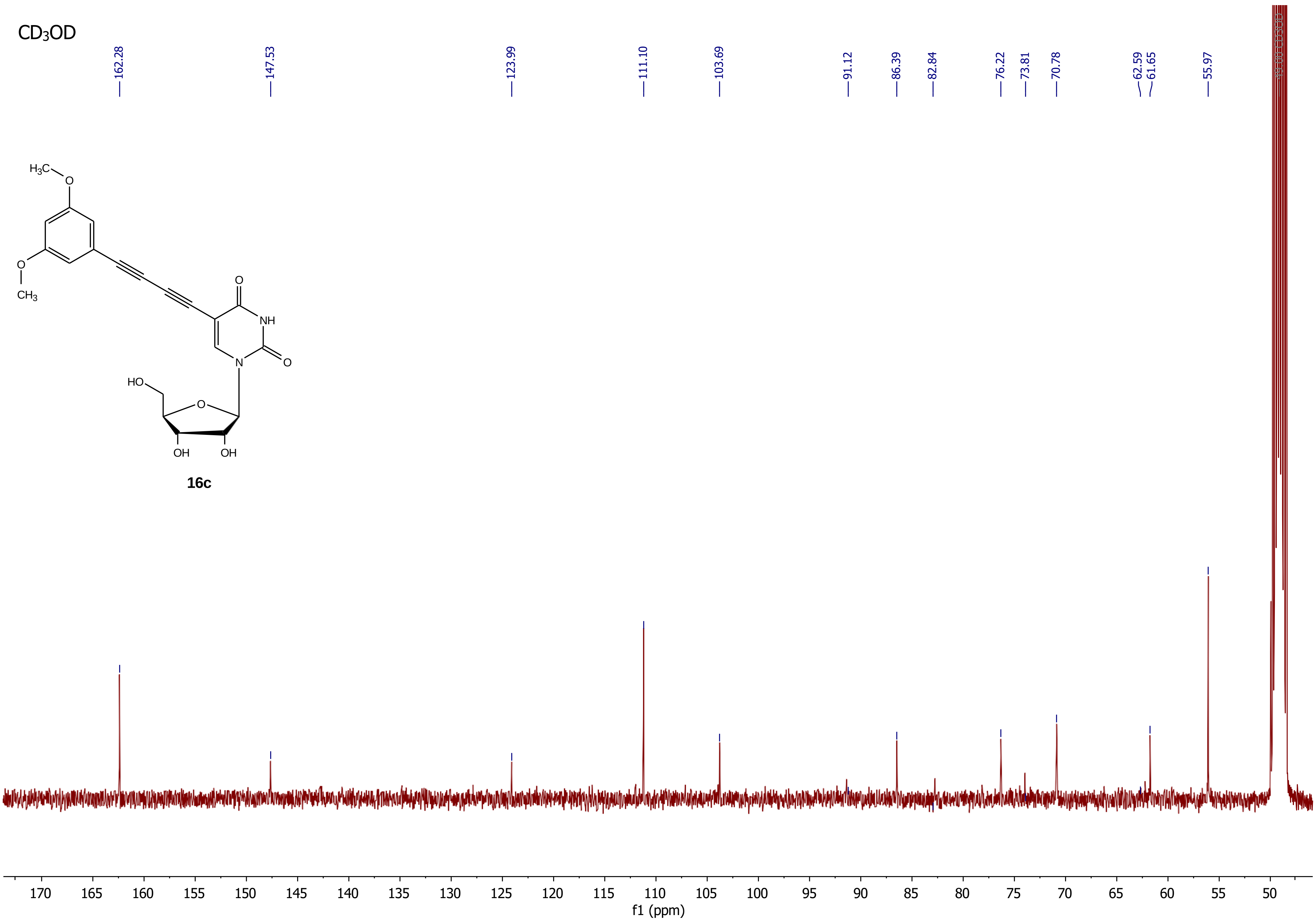
CD3OD
162.28
147.53
123.99
111.10
103.69
91.12
86.39
82.84
76.22
73.81
70.78
62.59
61.65
55.97
H3C
O
O
CH3
O
NH
O
N
HO
O
OH
OH
16c
170
165
160
155
150
145
140
135
130
125
120
115
110
105
100
95
90
85
80
75
70
65
60
55
50
f1 (ppm)

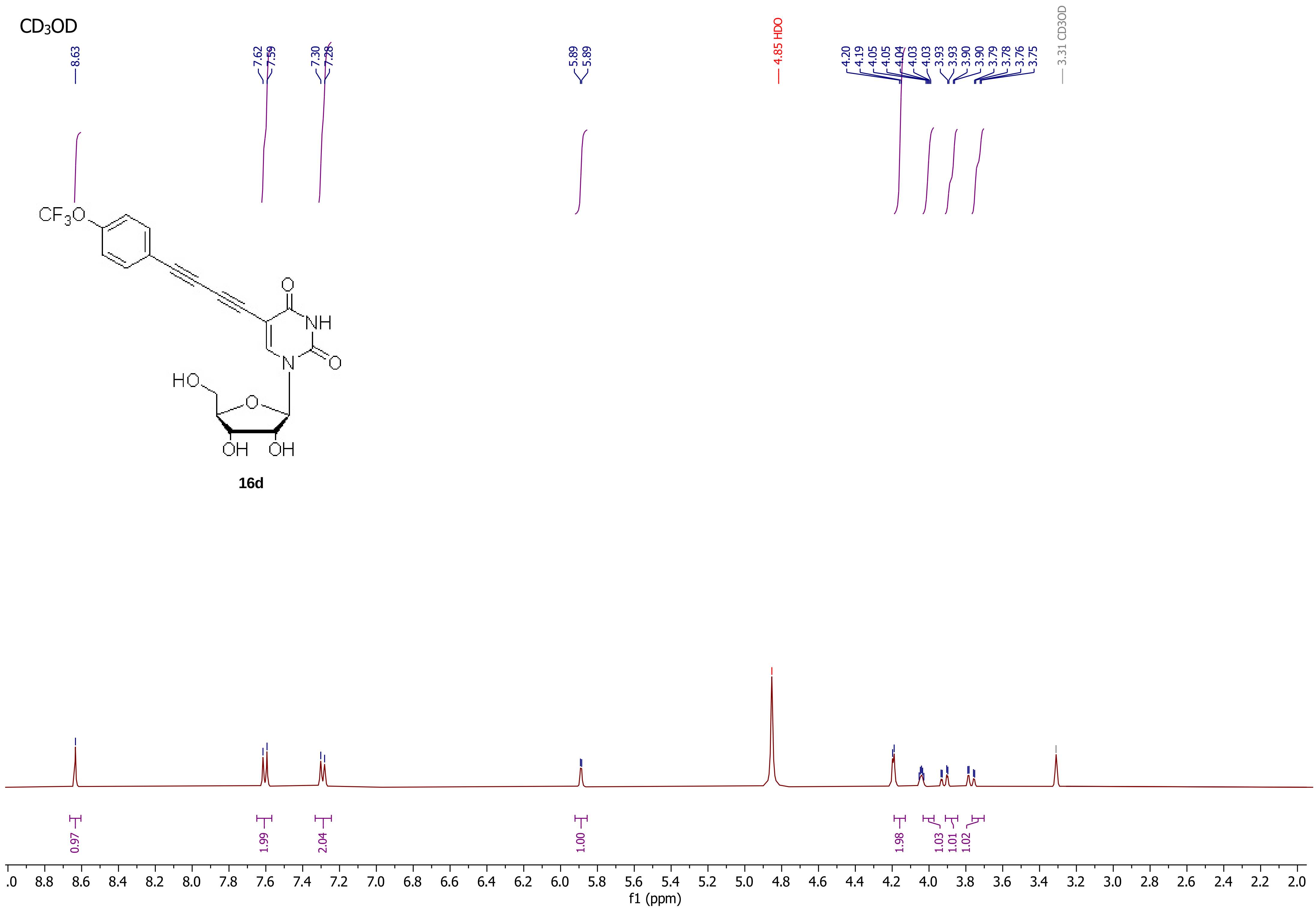
CD3OD
8.63
7.62
7.59
7.30
7.28
5.89
5.89
4.85 HDO
4.20
4.19
4.05
4.05
4.04
4.03
4.03
3.93
3.93
3.90
3.90
3.79
3.78
3.76
3.75
3.31 CD3OD
CF3O
NH
O
N
HO
OH
OH
16d
0.97
1.99
2.04
1.00
1.98
1.03
1.01
1.02
.0
8.8
8.6
8.4
8.2
8.0
7.8
7.6
7.4
7.2
7.0
6.8
6.6
6.4
6.2
6.0
5.8
5.6
5.4
5.2
5.0
4.8
4.6
4.4
4.2
4.0
3.8
3.6
3.4
3.2
3.0
2.8
2.6
2.4
2.2
2.0
f1 (ppm)

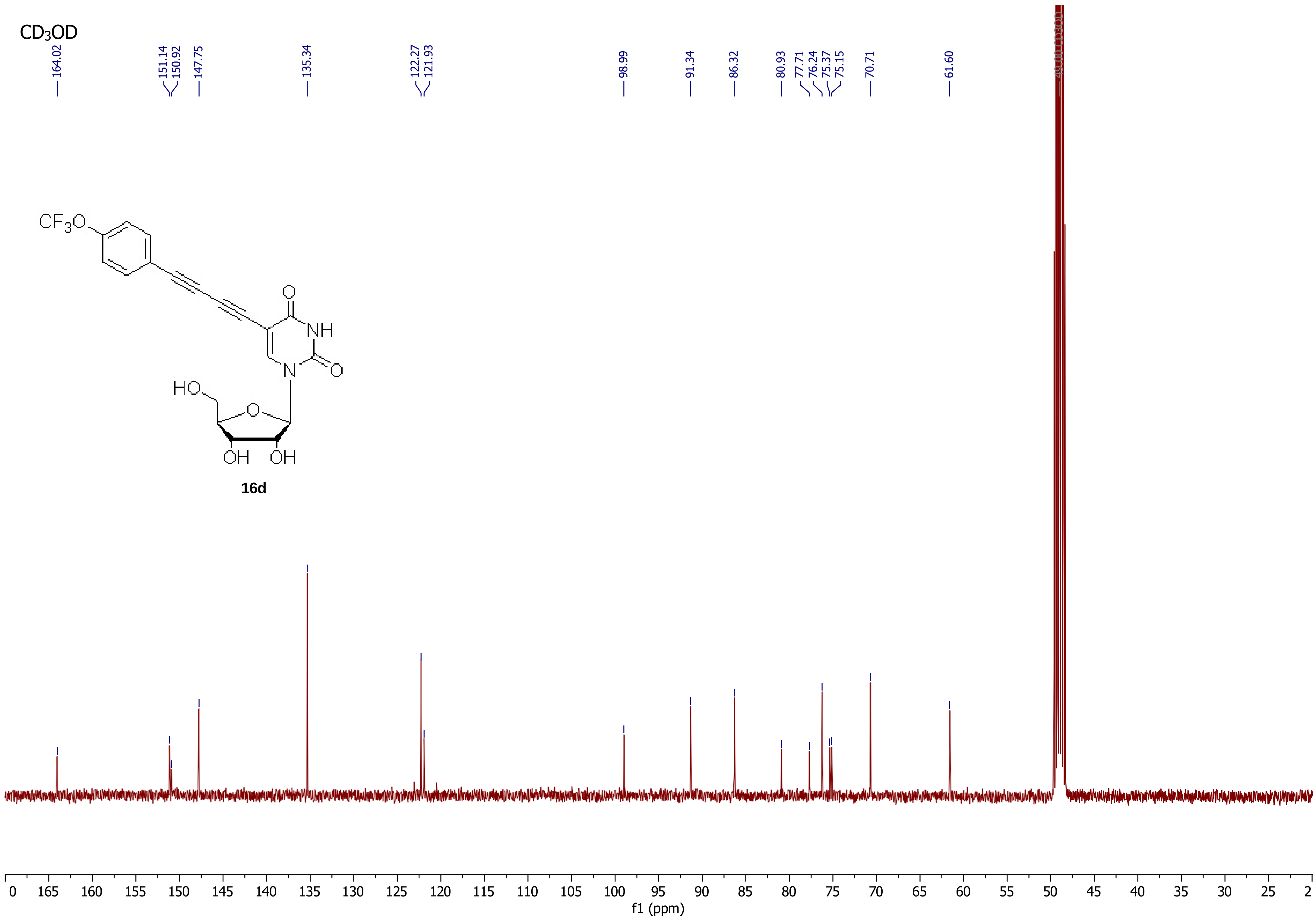
CD3OD
164.02
151.14
150.92
147.75
135.34
122.27
121.93
98.99
91.34
86.32
80.93
77.71
76.24
75.37
75.15
70.71
61.60
49.00 CD3OD
CF3O
NH
O
N
HO
OH
16d
f1 (ppm)

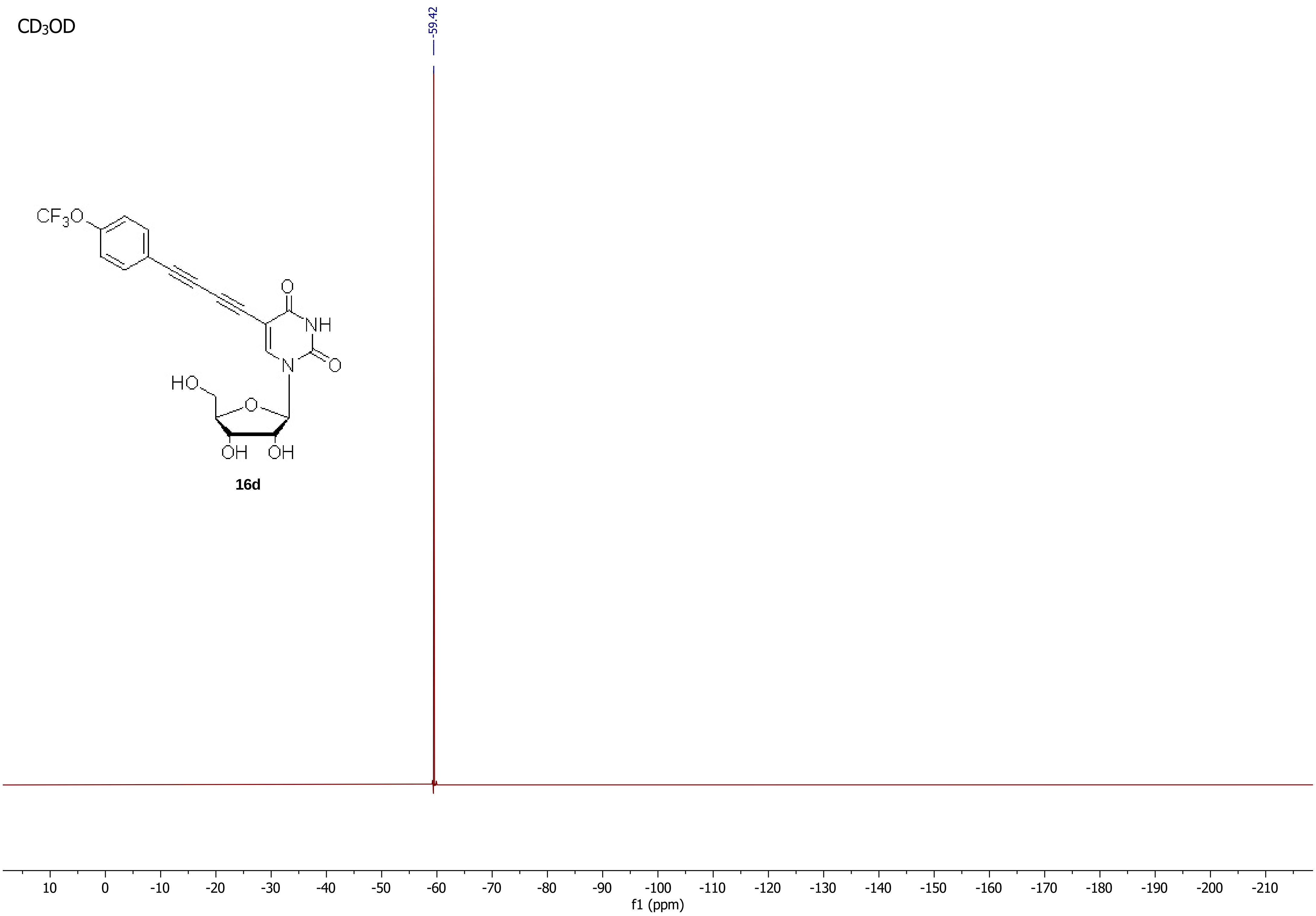
CD3OD
-59.42
CF3O
NH
O
N
HO
OH
OH
16d
10 0 -10 -20 -30 -40 -50 -60 -70 -80 -90 -100 -110 -120 -130 -140 -150 -160 -170 -180 -190 -200 -210
f1 (ppm)

CD$_3$OD

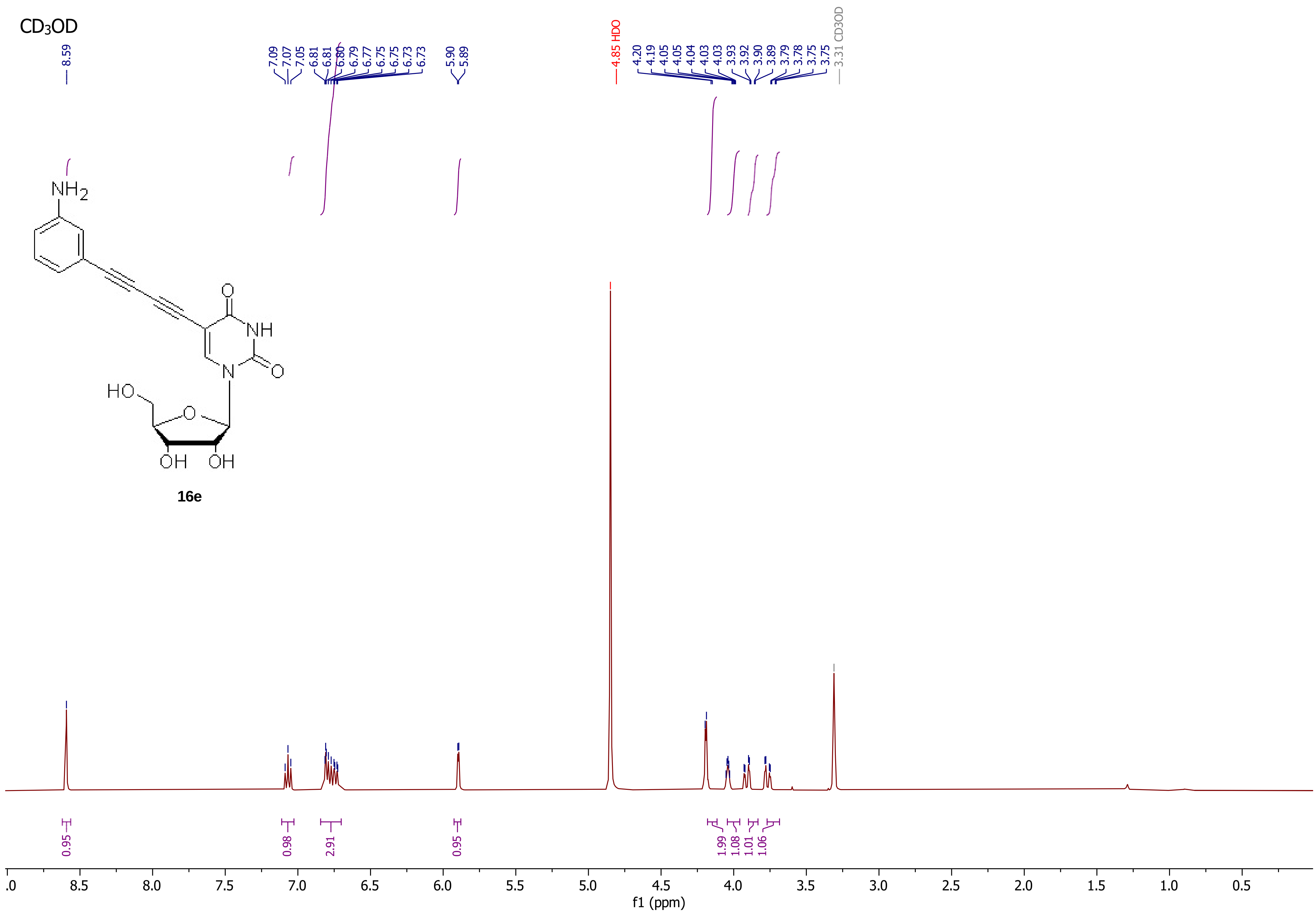
NH$_2$
NH
O
O
N
HO
O
OH
OH
8.59
7.09
7.07
7.05
6.81
6.81
6.80
6.79
6.77
6.75
6.75
6.73
6.73
5.90
5.89
4.85 HDO
4.20
4.19
4.05
4.05
4.04
4.03
4.03
3.93
3.92
3.90
3.89
3.79
3.78
3.75
3.75
3.31 CD3OD
0.95
0.98
2.91
0.95
1.99
1.08
1.01
1.06
f1 (ppm)

16e

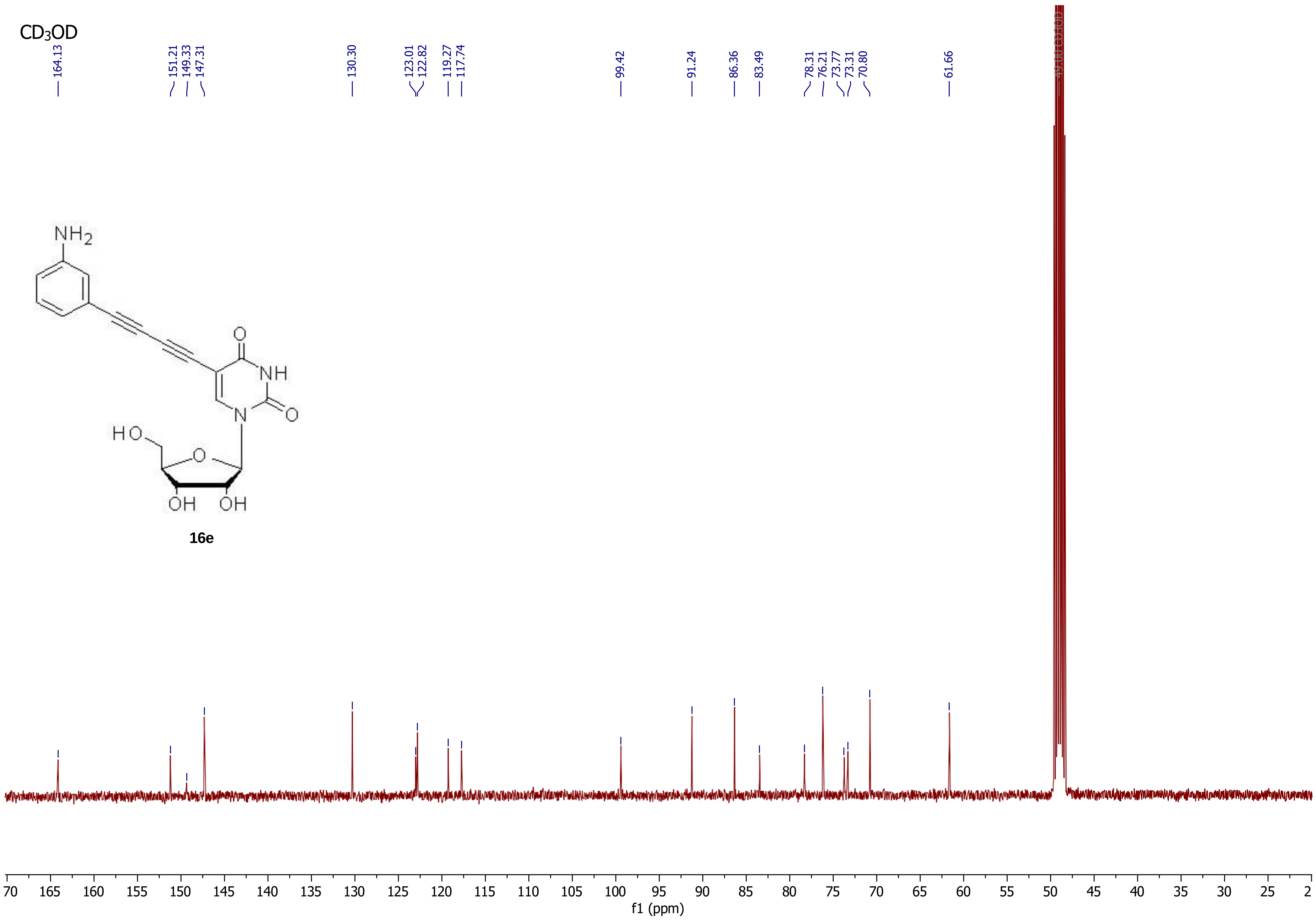
CD3OD
164.13
151.21
149.33
147.31
130.30
123.01
122.82
119.27
117.74
99.42
91.24
86.36
83.49
78.31
76.21
73.77
73.31
70.80
61.66
49.00 CD3OD
NH2
NH
O
N
HO
OH
OH
16e
70 165 160 155 150 145 140 135 130 125 120 115 110 105 100 95 90 85 80 75 70 65 60 55 50 45 40 35 30 25 2
f1 (ppm)

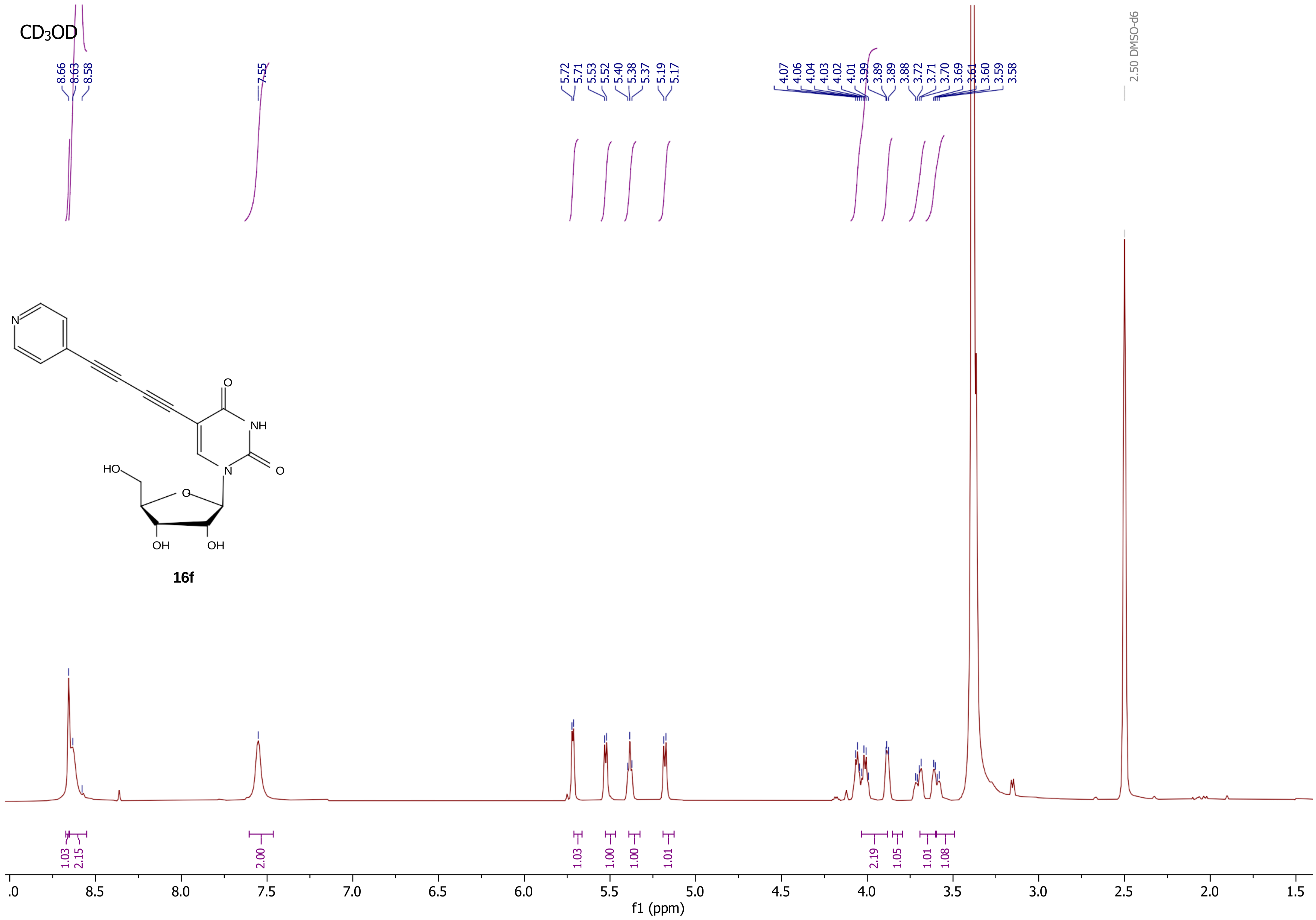
CD3OD
8.66
8.63
8.58
7.55
5.72
5.71
5.53
5.52
5.40
5.38
5.37
5.19
5.17
4.07
4.06
4.04
4.03
4.02
4.01
3.99
3.89
3.89
3.88
3.72
3.71
3.70
3.69
3.61
3.60
3.59
3.58
2.50 DMSO-d6
N
O
NH
HO
OH
16f
1.03
2.15
2.00
1.00
1.01
2.19
1.05
1.08
f1 (ppm)

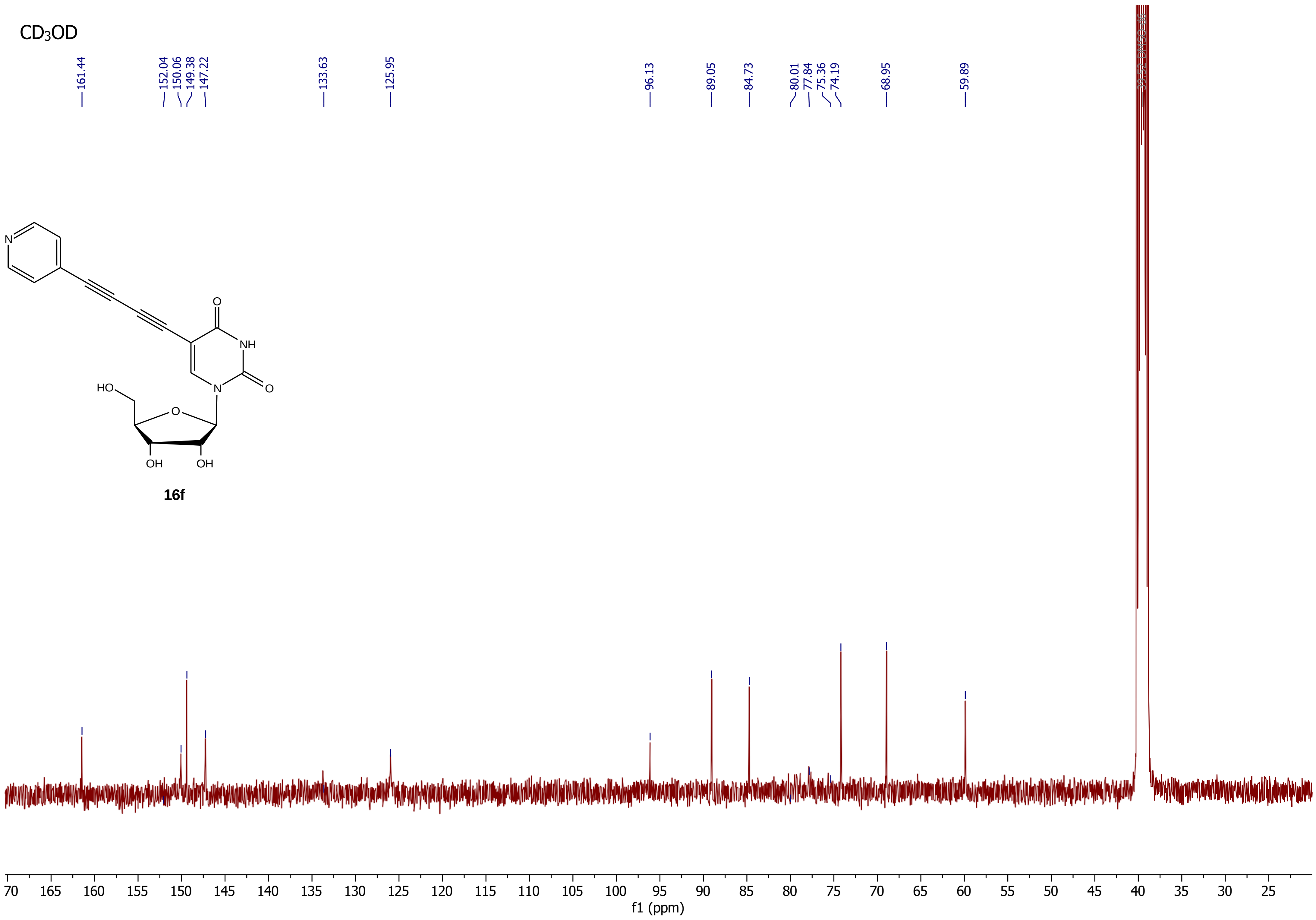
CD3OD
161.44
152.04
150.06
149.38
147.22
133.63
125.95
96.13
89.05
84.73
80.01
77.84
75.36
74.19
68.95
59.89
N
O
NH
O
N
HO
O
OH
OH
16f
70 165 160 155 150 145 140 135 130 125 120 115 110 105 100 95 90 85 80 75 70 65 60 55 50 45 40 35 30 25
f1 (ppm)

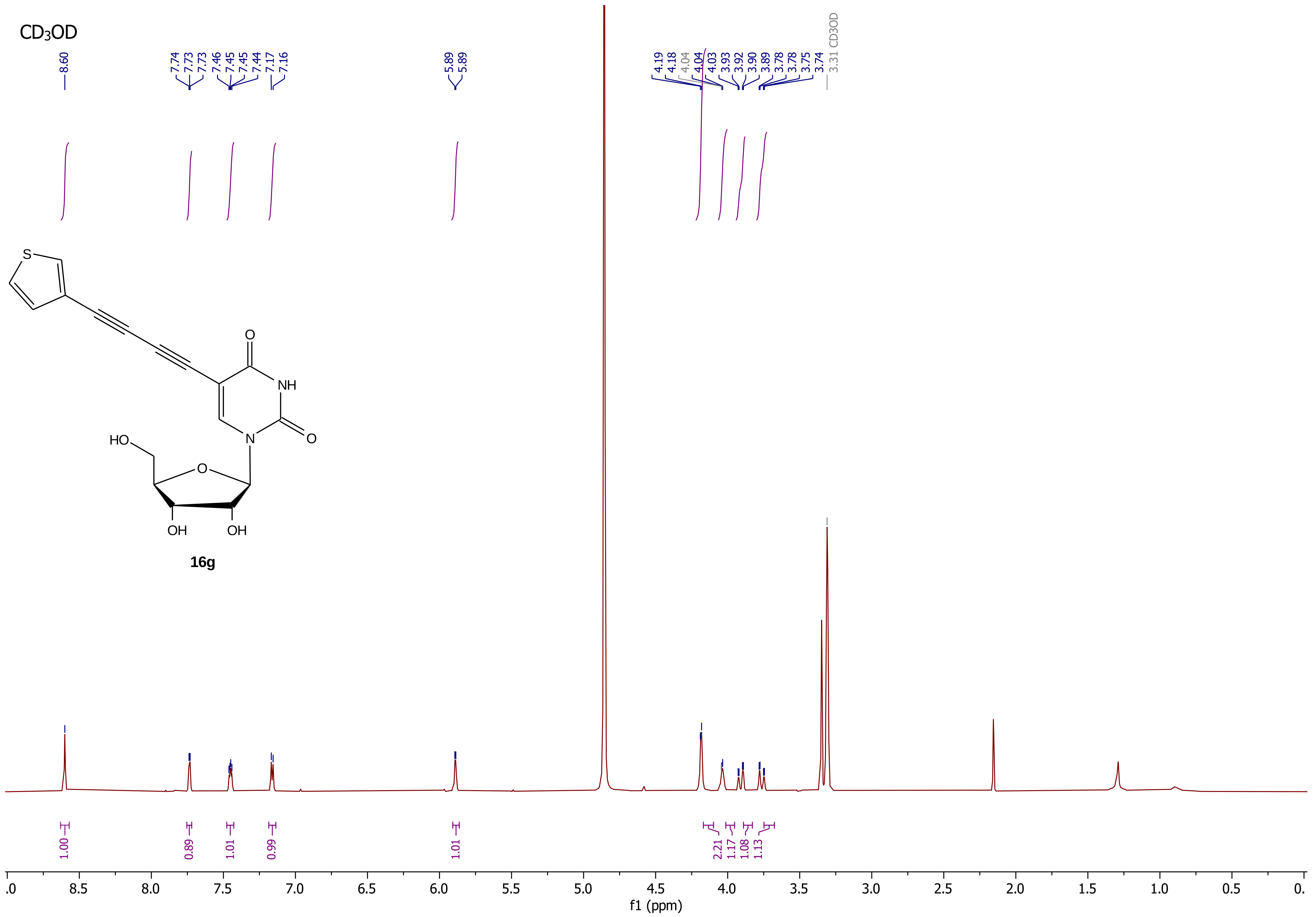
CD3OD
8.60
7.74
7.73
7.73
7.46
7.45
7.45
7.44
7.17
7.16
5.89
5.89
4.19
4.18
4.04
4.04
4.03
3.93
3.92
3.90
3.89
3.78
3.78
3.75
3.74
3.31 CD3OD
S
O
NH
O
N
HO
O
OH
OH
16g
1.00
0.89
1.01
0.99
1.01
2.21
1.17
1.08
1.13
.0
8.5
8.0
7.5
7.0
6.5
6.0
5.5
5.0
4.5
4.0
3.5
3.0
2.5
2.0
1.5
1.0
0.5
0.
f1 (ppm)

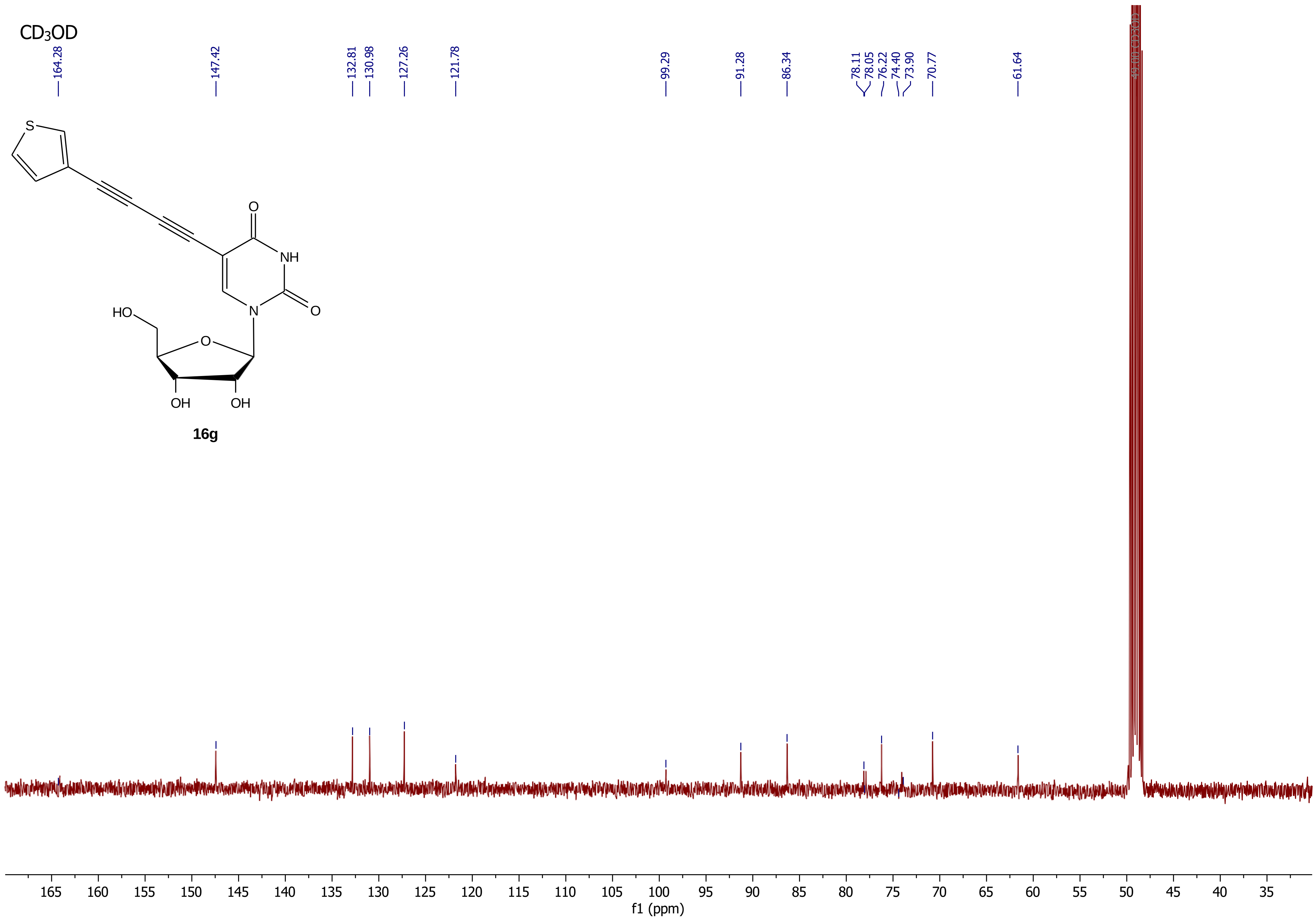
CD3OD
164.28
147.42
132.81
130.98
127.26
121.78
99.29
91.28
86.34
78.11
78.05
76.22
74.40
73.90
70.77
61.64
49.00 CD3OD
S
O
NH
N
O
HO
O
OH
OH
16g
165 160 155 150 145 140 135 130 125 120 115 110 105 100 95 90 85 80 75 70 65 60 55 50 45 40 35
f1 (ppm)

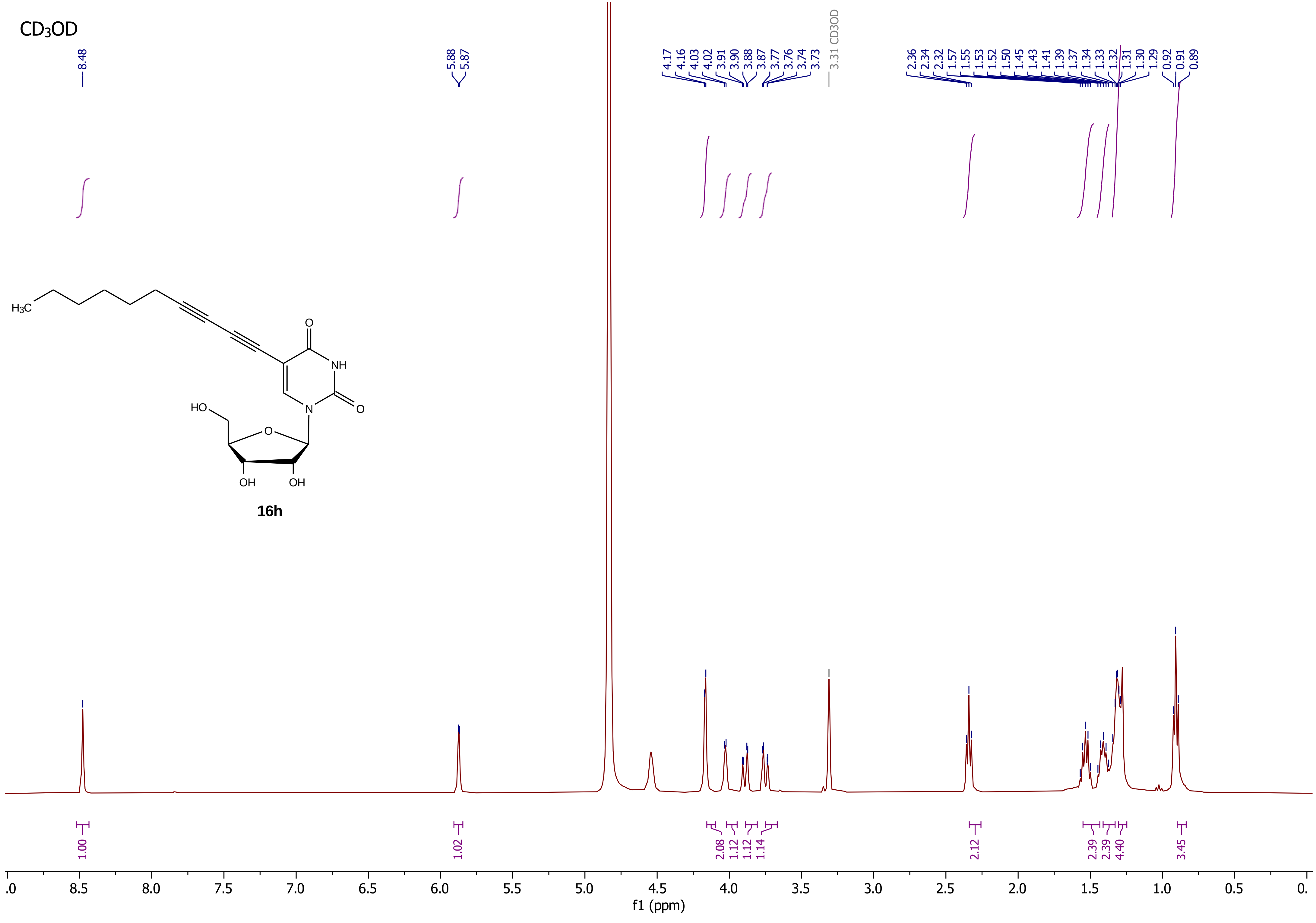
CD3OD
8.48
5.88
5.87
4.17
4.16
4.03
4.02
3.91
3.90
3.88
3.87
3.77
3.76
3.74
3.73
3.31 CD3OD
2.36
2.34
2.32
1.57
1.55
1.53
1.52
1.50
1.45
1.43
1.41
1.39
1.37
1.34
1.33
1.32
1.31
1.30
1.29
0.92
0.91
0.89
H3C
O
NH
O
N
HO
O
OH
OH
1.00
1.02
2.08
1.12
1.12
1.14
2.12
2.39
2.39
4.40
3.45
f1 (ppm)

16h

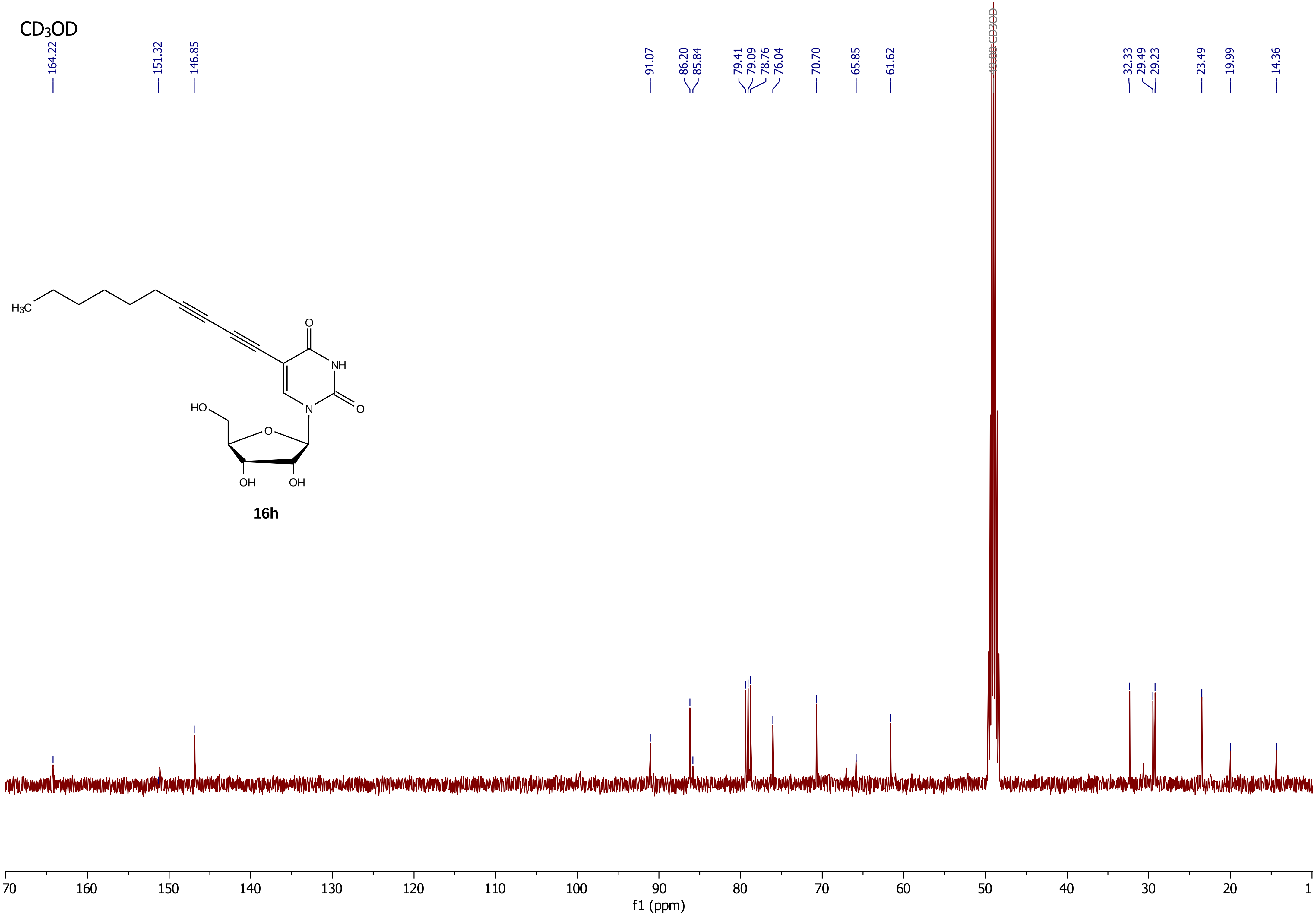
CD3OD
164.22
151.32
146.85
91.07
86.20
85.84
79.41
79.09
78.76
76.04
70.70
65.85
61.62
32.33
29.49
29.23
23.49
19.99
14.36
H3C
O
NH
O
N
HO
O
OH
OH
16h
f1 (ppm)

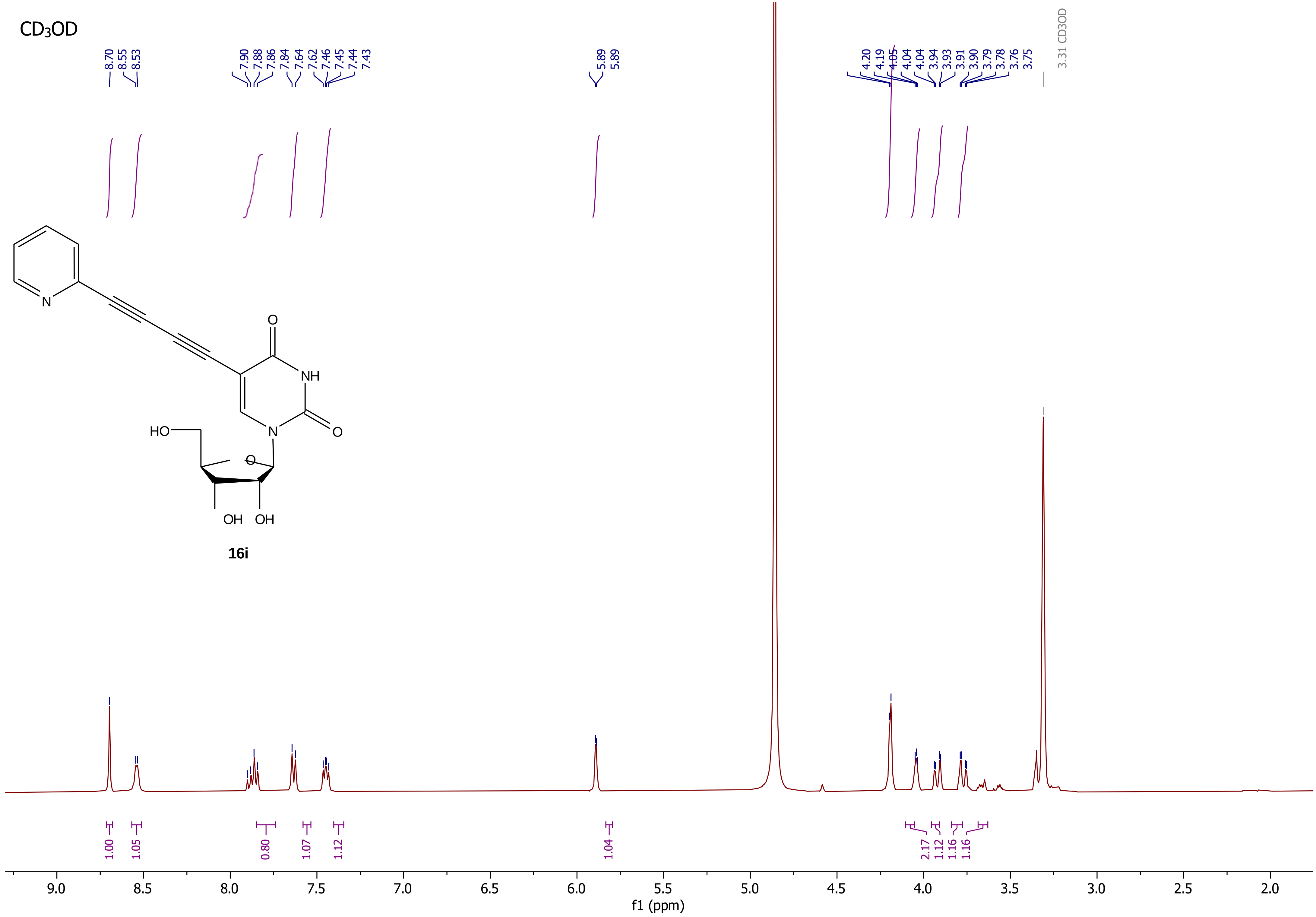
CD3OD
8.70
8.55
8.53
7.90
7.88
7.86
7.84
7.64
7.62
7.46
7.45
7.44
7.43
5.89
5.89
4.20
4.19
4.05
4.04
4.04
3.94
3.93
3.91
3.90
3.79
3.78
3.76
3.75
3.31 CD3OD
N
O
NH
O
N
HO
O
OH OH
16i
1.00
1.05
0.80
1.07
1.12
1.04
2.17
1.12
1.16
1.16
9.0
8.5
8.0
7.5
7.0
6.5
6.0
5.5
5.0
4.5
4.0
3.5
3.0
2.5
2.0
f1 (ppm)

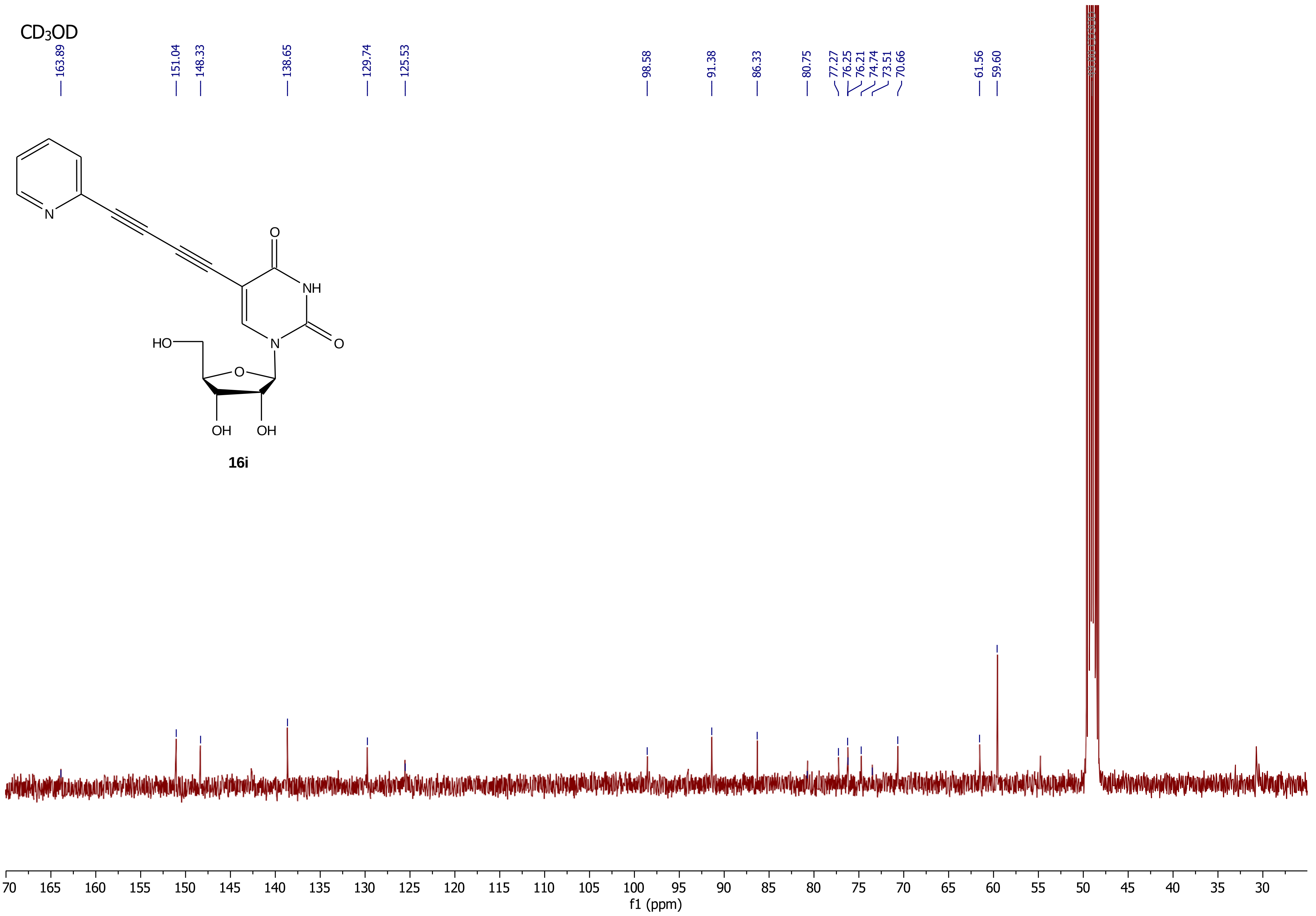
CD3OD
163.89
151.04
148.33
138.65
129.74
125.53
98.58
91.38
86.33
80.75
77.27
76.25
76.21
74.74
73.51
70.66
61.56
59.60
HO
O
OH
OH
N
NH
N
16i
70
165
160
155
150
145
140
135
130
125
120
115
110
105
100
95
90
85
80
75
70
65
60
55
50
45
40
35
30
f1 (ppm)

CD3OD

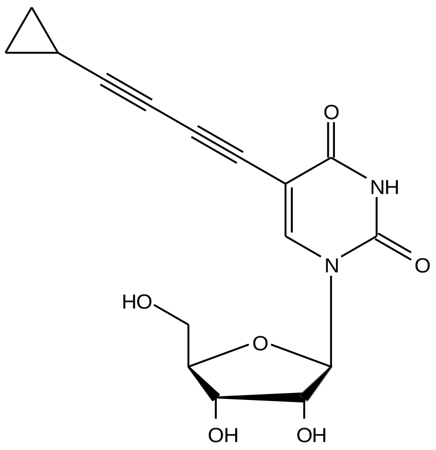

16j

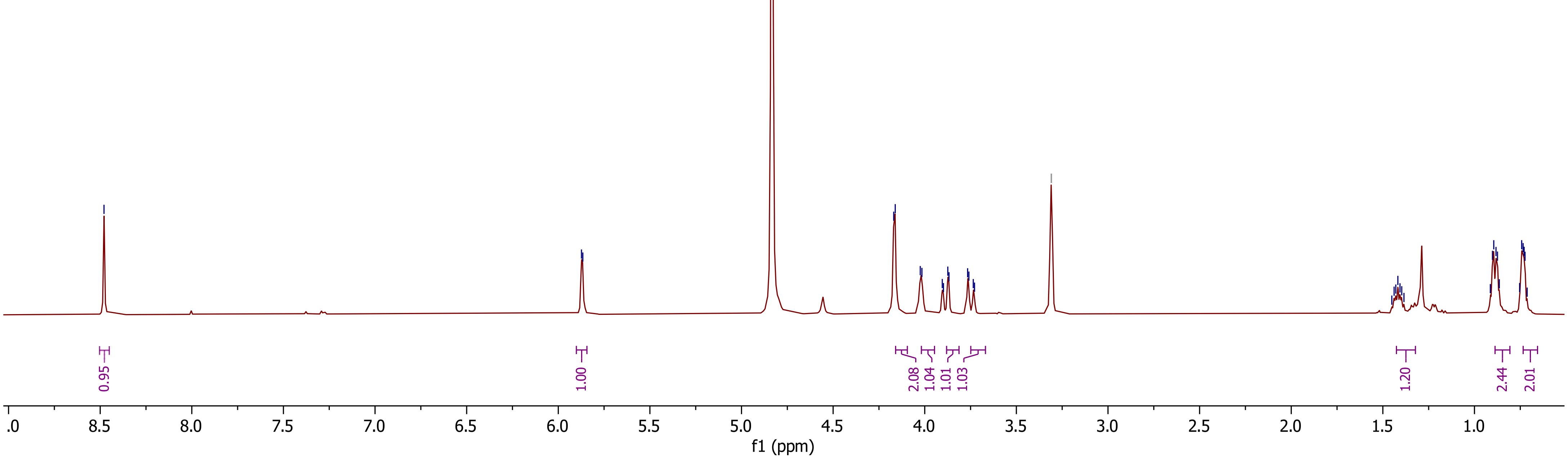
8.48
5.87
5.87
4.83 HDO
4.17
4.16
4.02
4.02
3.90
3.90
3.87
3.87
3.77
3.76
3.74
3.73
3.31 CD3OD
1.45
1.44
1.43
1.42
1.41
1.40
1.38
0.91
0.90
0.89
0.88
0.87
0.86
0.75
0.74
0.73
0.73
0.72
0.71
0.95
1.00
2.08
1.04
1.01
1.03
1.20
2.44
2.01
.0
8.5
8.0
7.5
7.0
6.5
6.0
5.5
5.0
4.5
4.0
3.5
3.0
2.5
2.0
1.5
1.0
f1 (ppm)

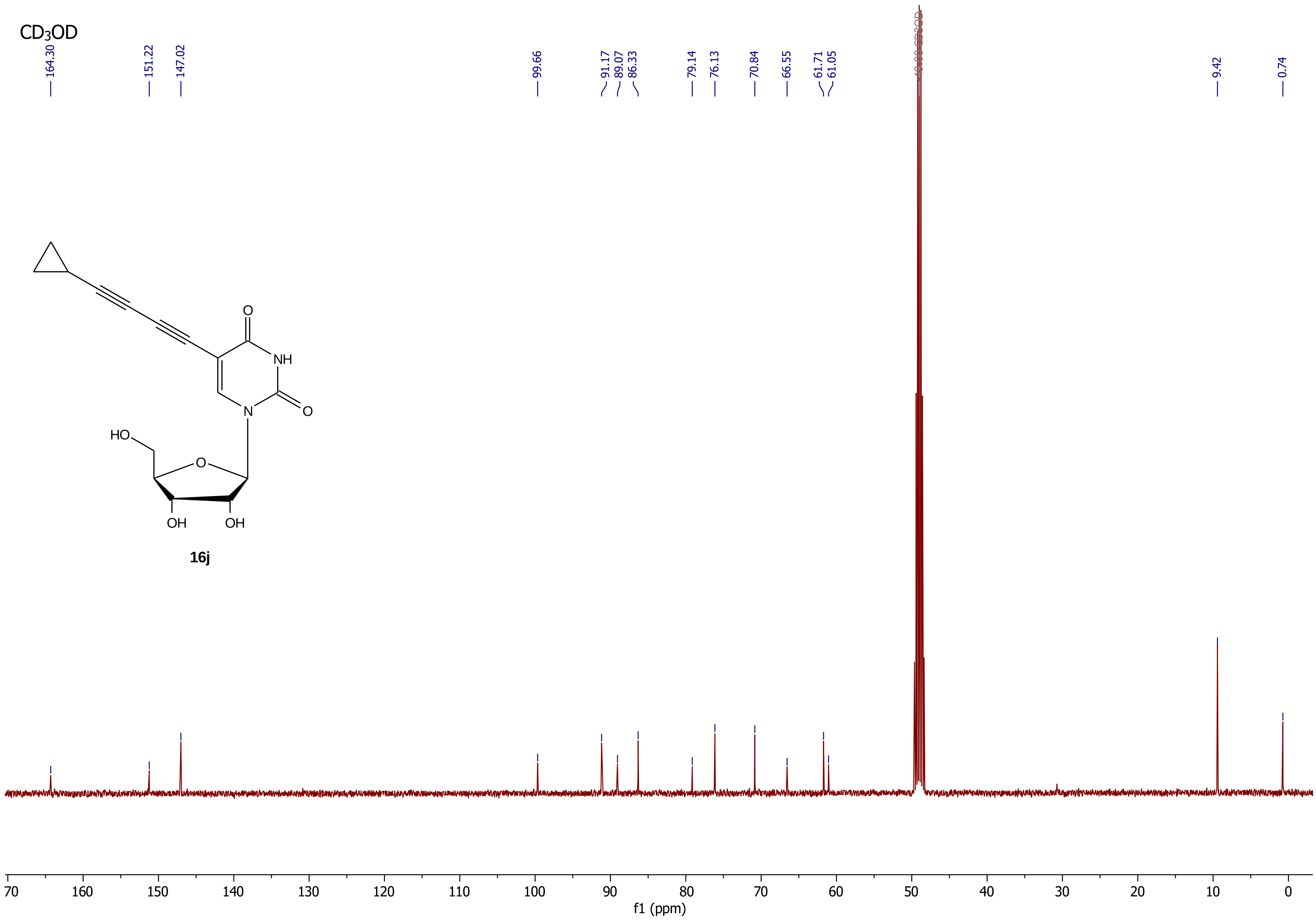

CD3OD
164.30
151.22
147.02
99.66
91.17
89.07
86.33
79.14
76.13
70.84
66.55
61.71
61.05
9.42
0.74
O
NH
O
N
HO
O
OH
OH
16j
70 160 150 140 130 120 110 100 90 80 70 60 50 40 30 20 10 0
f1 (ppm)

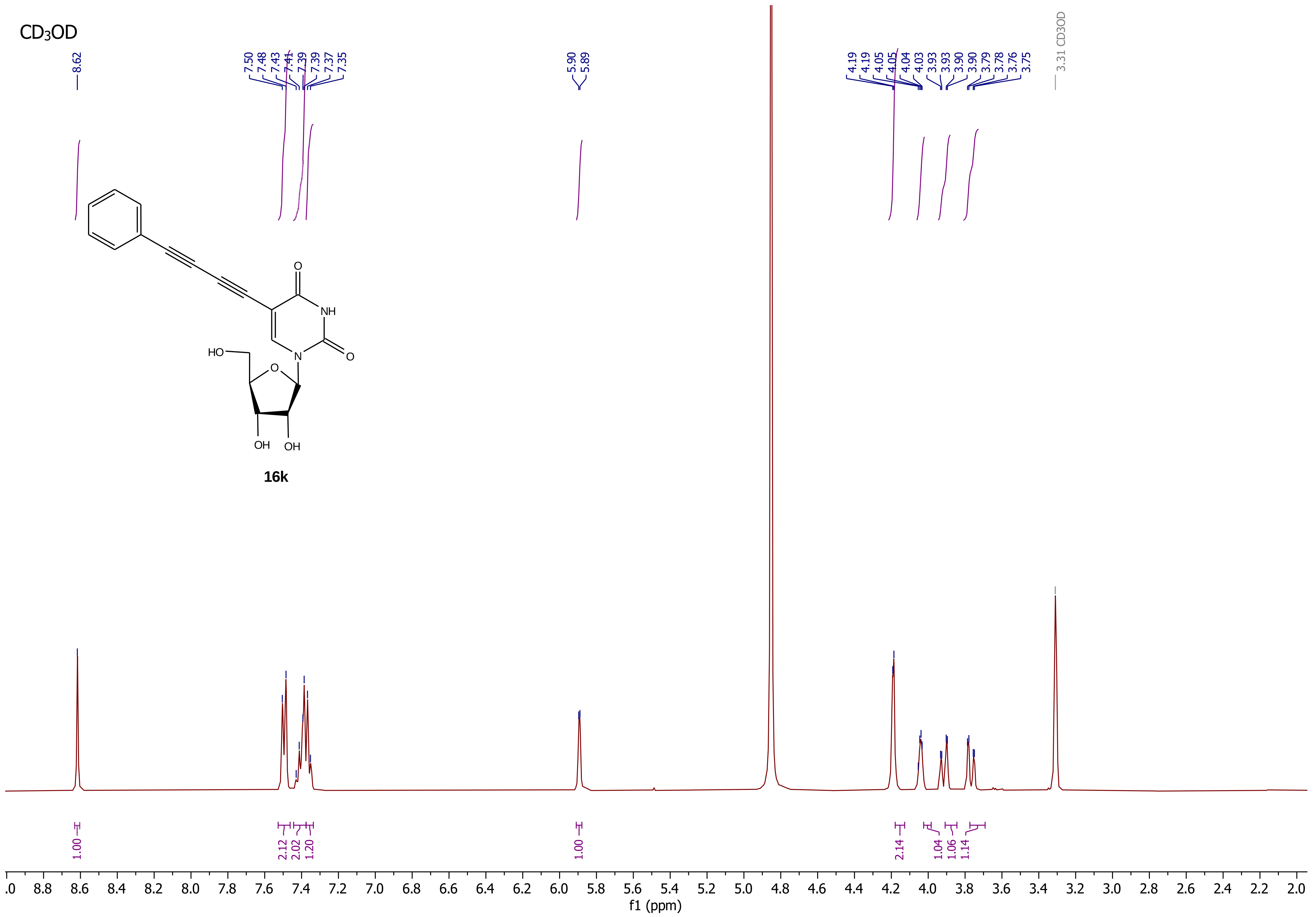

CD3OD
8.62
7.50
7.48
7.43
7.41
7.39
7.39
7.37
7.35
5.90
5.89
4.19
4.19
4.05
4.05
4.04
4.03
3.93
3.93
3.90
3.90
3.79
3.78
3.76
3.75
3.31 CD3OD
HO
O
OH
OH
N
NH
O
O
16k
1.00
2.12
2.02
1.20
1.00
2.14
1.04
1.06
1.14
f1 (ppm)

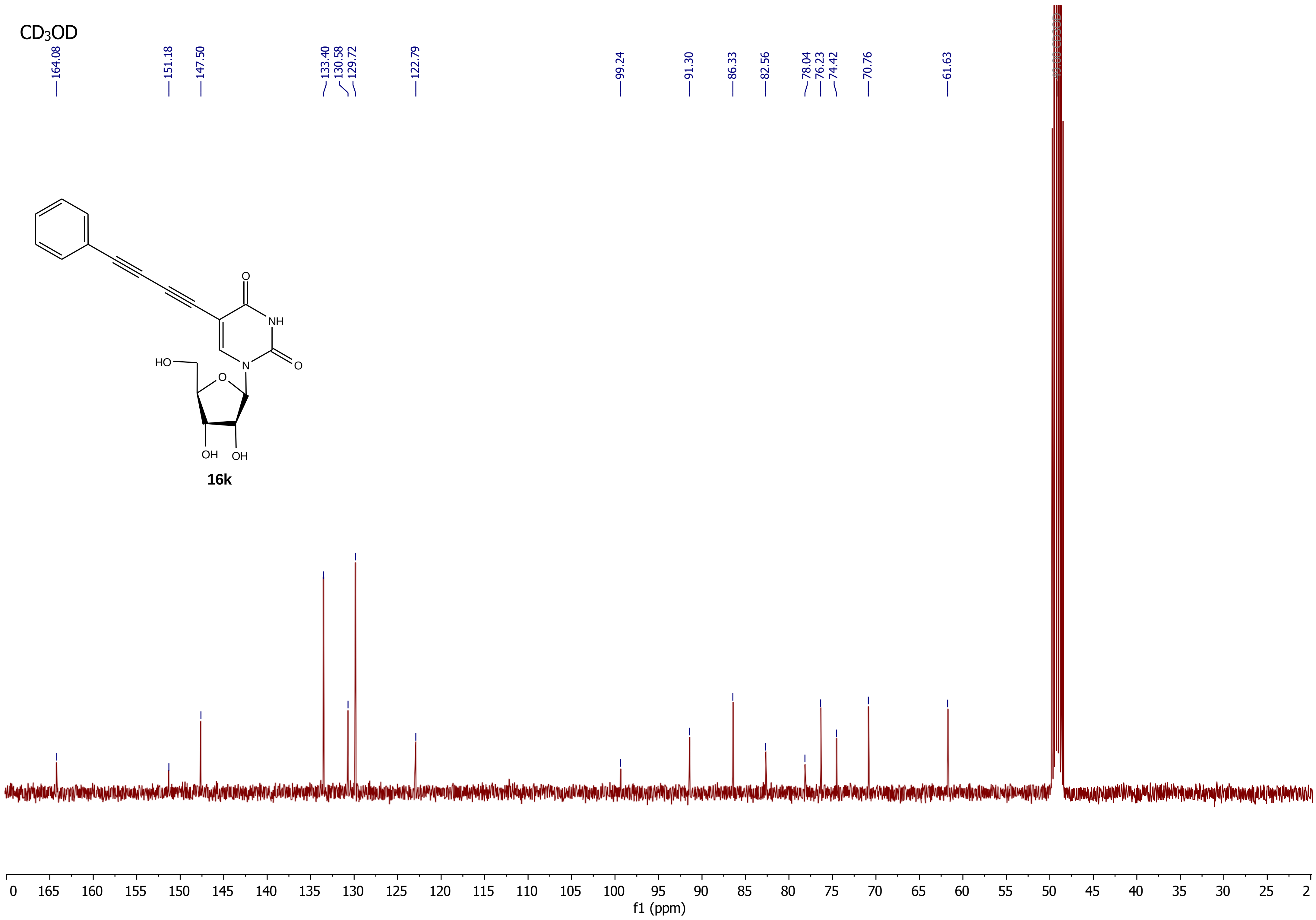
CD3OD
164.08
151.18
147.50
133.40
130.58
129.72
122.79
99.24
91.30
86.33
82.56
78.04
76.23
74.42
70.76
61.63
O
NH
O
N
HO
O
OH
OH
16k
0 165 160 155 150 145 140 135 130 125 120 115 110 105 100 95 90 85 80 75 70 65 60 55 50 45 40 35 30 25 2
f1 (ppm)

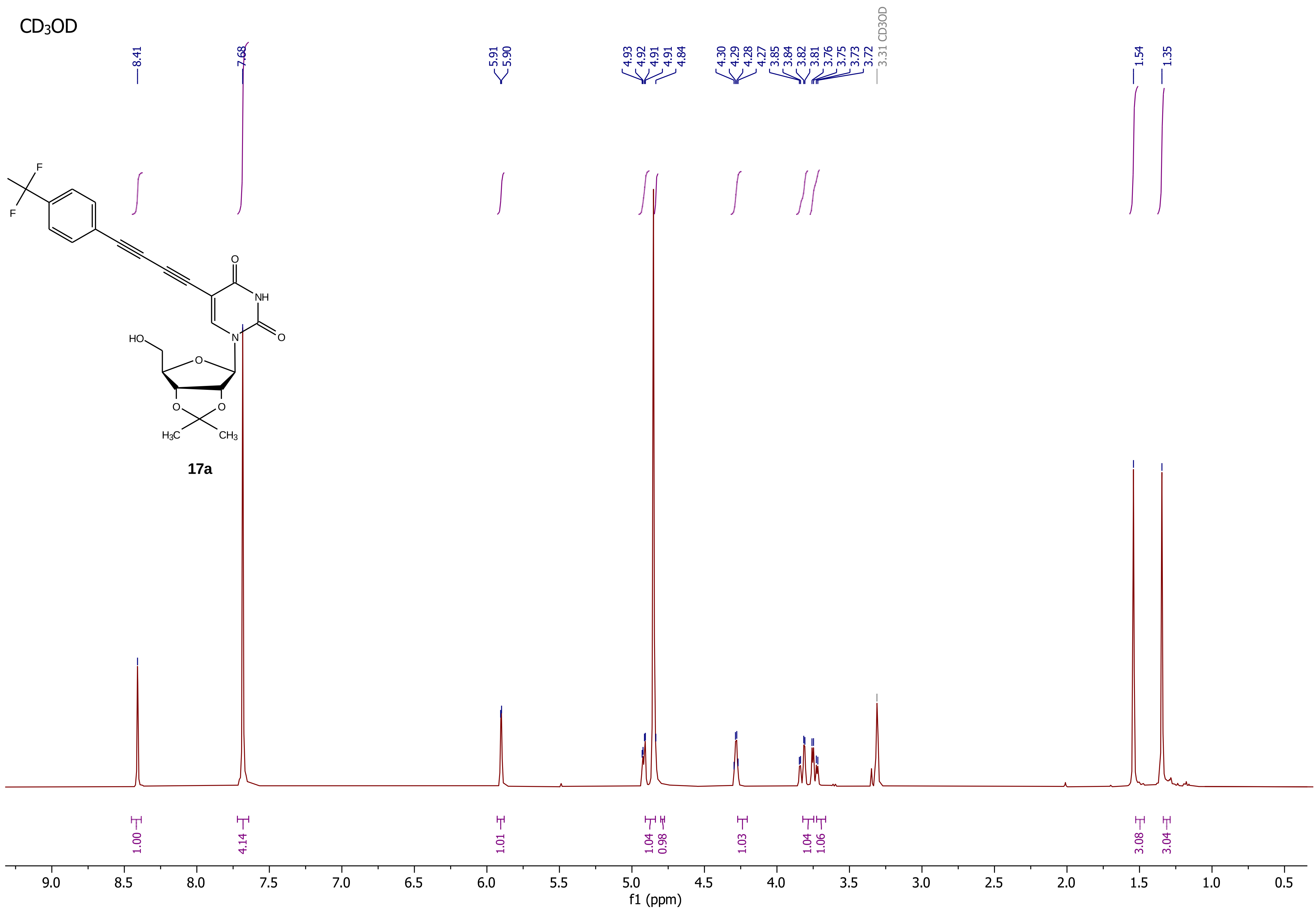
CD3OD
8.41
7.68
5.91
5.90
4.93
4.92
4.91
4.91
4.84
4.30
4.29
4.28
4.27
3.85
3.84
3.82
3.81
3.76
3.75
3.73
3.72
3.31 CD3OD
1.54
1.35
F
F
O
NH
O
N
HO
O
O
O
H3C
CH3
17a
1.00
4.14
1.01
1.04
0.98
1.03
1.04
1.06
3.08
3.04
9.0
8.5
8.0
7.5
7.0
6.5
6.0
5.5
5.0
4.5
4.0
3.5
3.0
2.5
2.0
1.5
1.0
0.5
f1 (ppm)

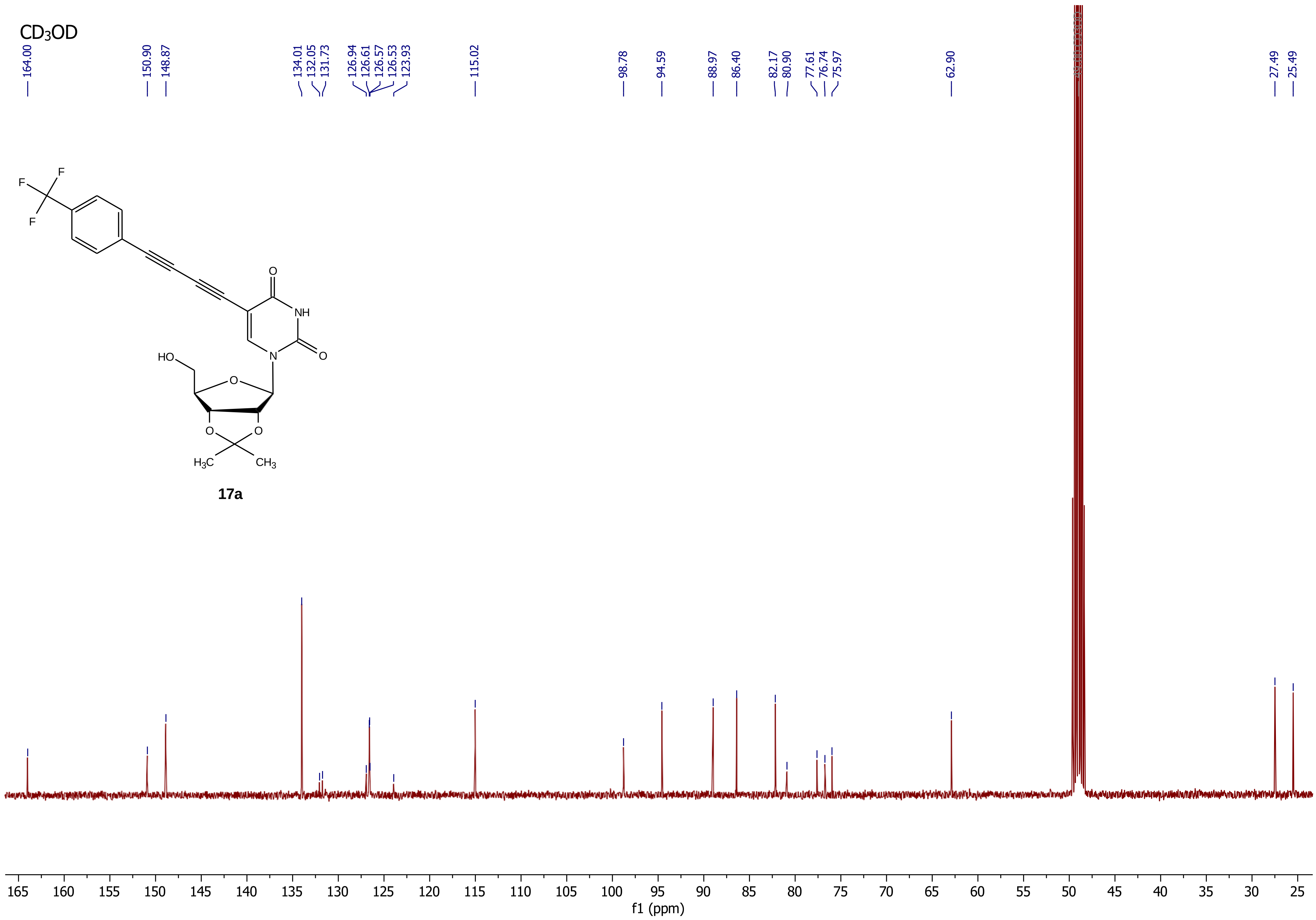
CD3OD
164.00
150.90
148.87
134.01
132.05
131.73
126.94
126.61
126.57
126.53
123.93
115.02
98.78
94.59
88.97
86.40
82.17
80.90
77.61
76.74
75.97
62.90
27.49
25.49
F
F
F
O
NH
N
O
HO
O
O
O
H3C
CH3
17a
165
160
155
150
145
140
135
130
125
120
115
110
105
100
95
90
85
80
75
70
65
60
55
50
45
40
35
30
25
f1 (ppm)

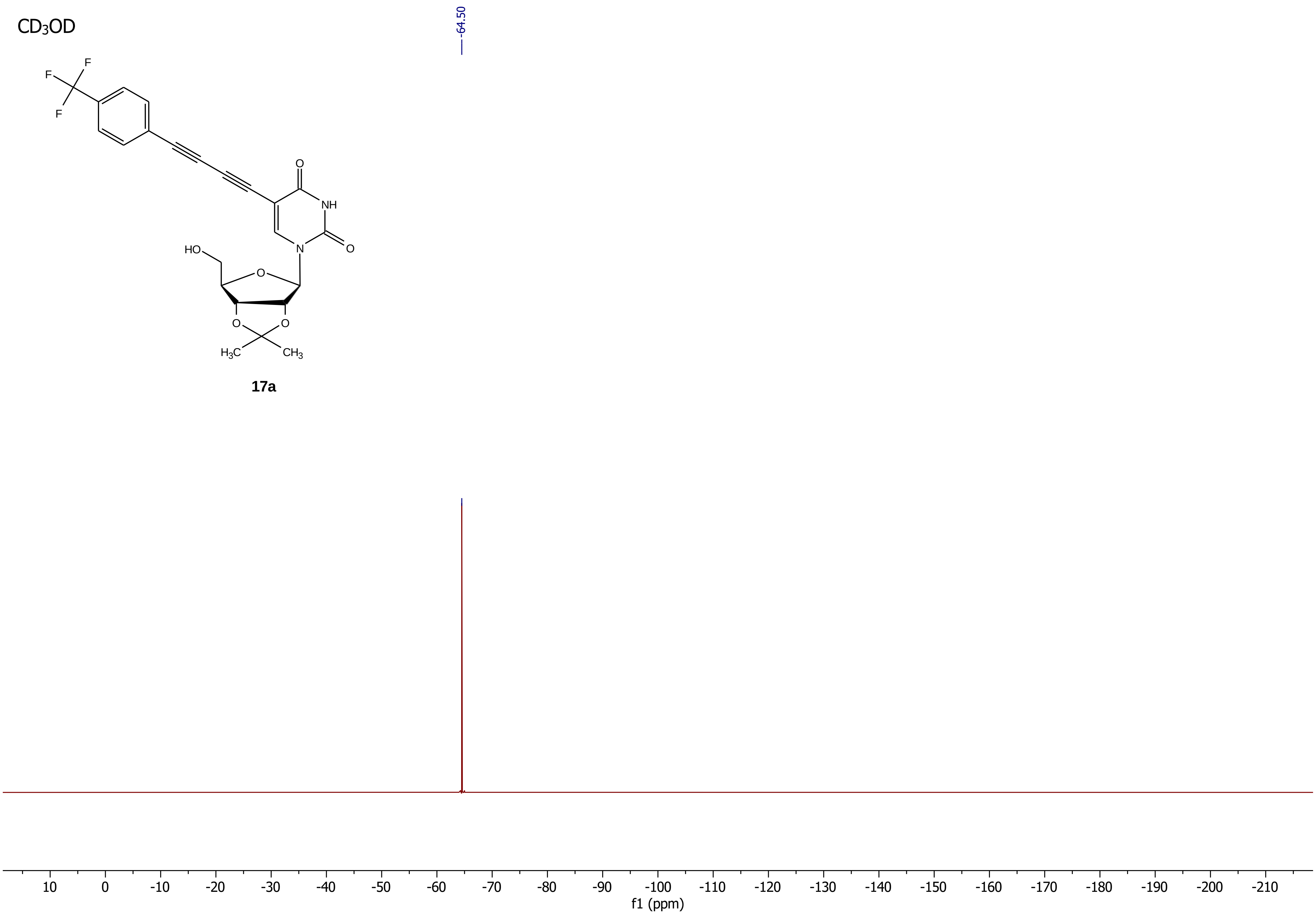
CD3OD
-64.50
F
F
F
O
NH
O
N
HO
O
O
O
H3C
CH3
17a
10 0 -10 -20 -30 -40 -50 -60 -70 -80 -90 -100 -110 -120 -130 -140 -150 -160 -170 -180 -190 -200 -210
f1 (ppm)

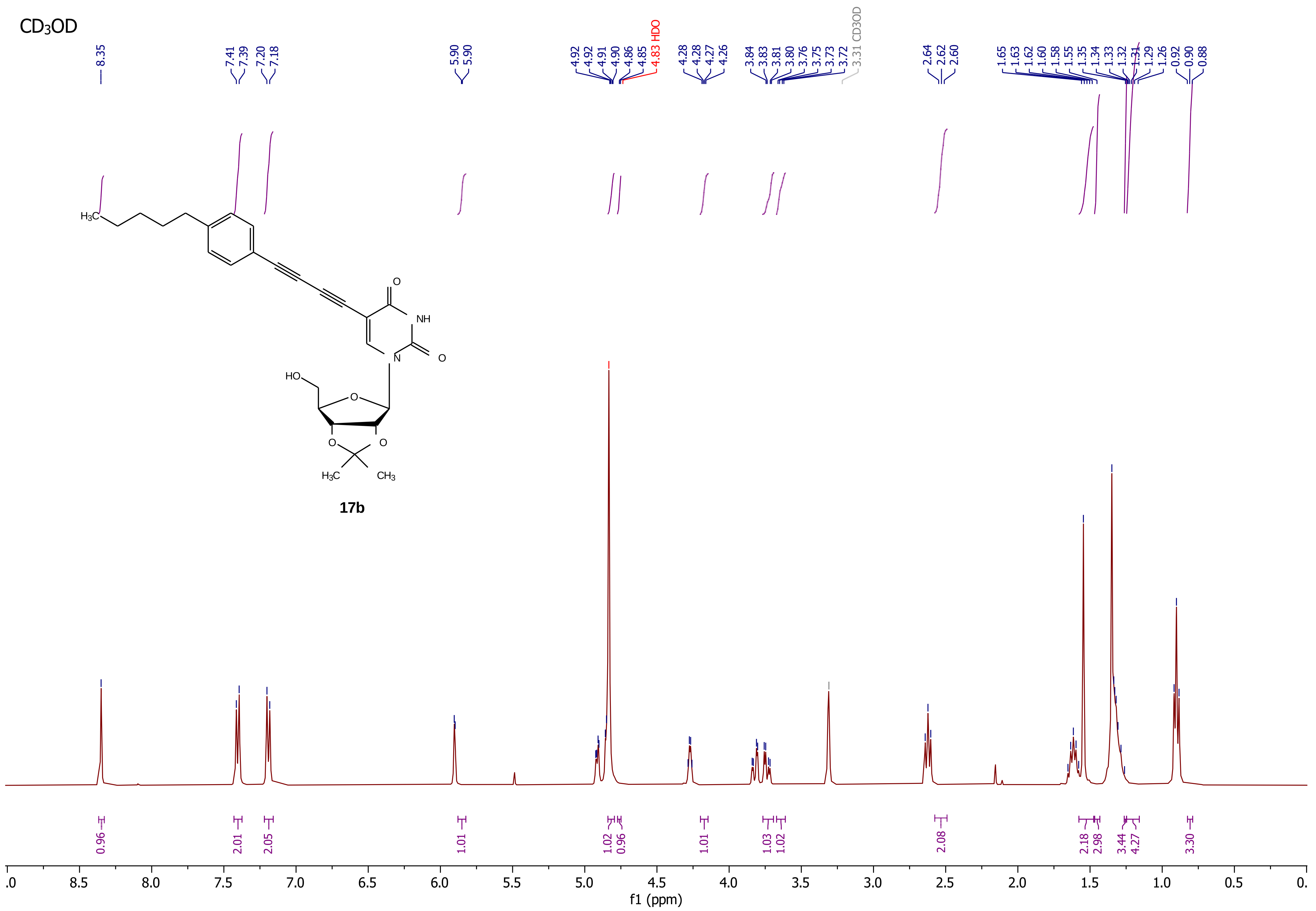
CD3OD
8.35
7.41
7.39
7.20
7.18
5.90
5.90
4.92
4.92
4.91
4.90
4.86
4.85
4.83 HDO
4.28
4.28
4.27
4.26
3.84
3.83
3.81
3.80
3.76
3.75
3.73
3.72
3.31 CD3OD
2.64
2.62
2.60
1.65
1.63
1.62
1.60
1.58
1.55
1.35
1.34
1.33
1.32
1.31
1.29
1.26
0.92
0.90
0.88
0.96
2.01
2.05
1.01
1.02
0.96
1.01
1.03
1.02
2.08
2.18
2.98
3.44
4.27
3.30
f1 (ppm)
17b

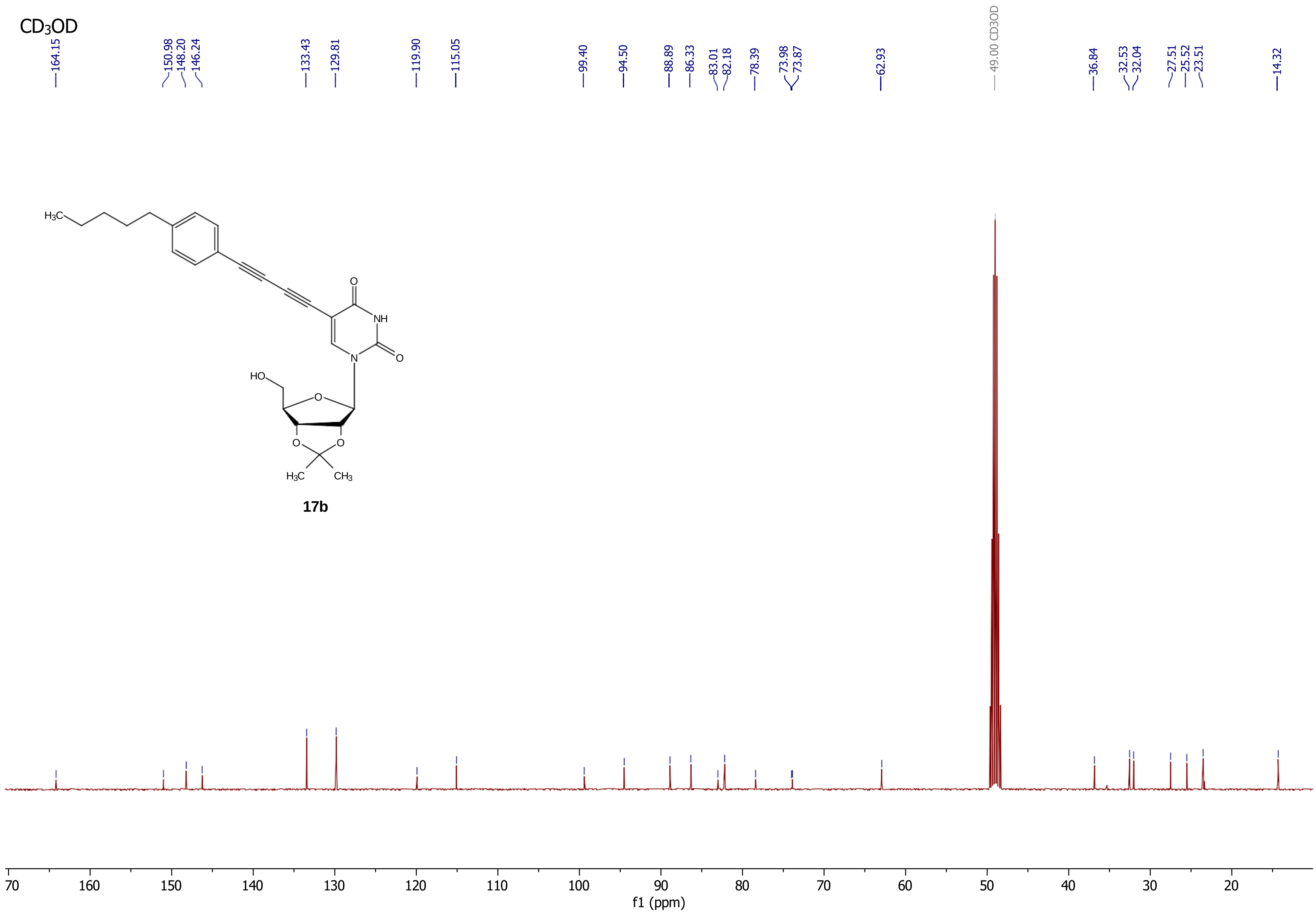
CD3OD
164.15
150.98
148.20
146.24
133.43
129.81
119.90
115.05
99.40
94.50
88.89
86.33
83.01
82.18
78.39
73.98
73.87
62.93
49.00 CD3OD
36.84
32.53
32.04
27.51
25.52
23.51
14.32
H3C
HO
O
NH
N
H3C
CH3
17b
70
160
150
140
130
120
110
100
90
80
70
60
50
40
30
20
f1 (ppm)

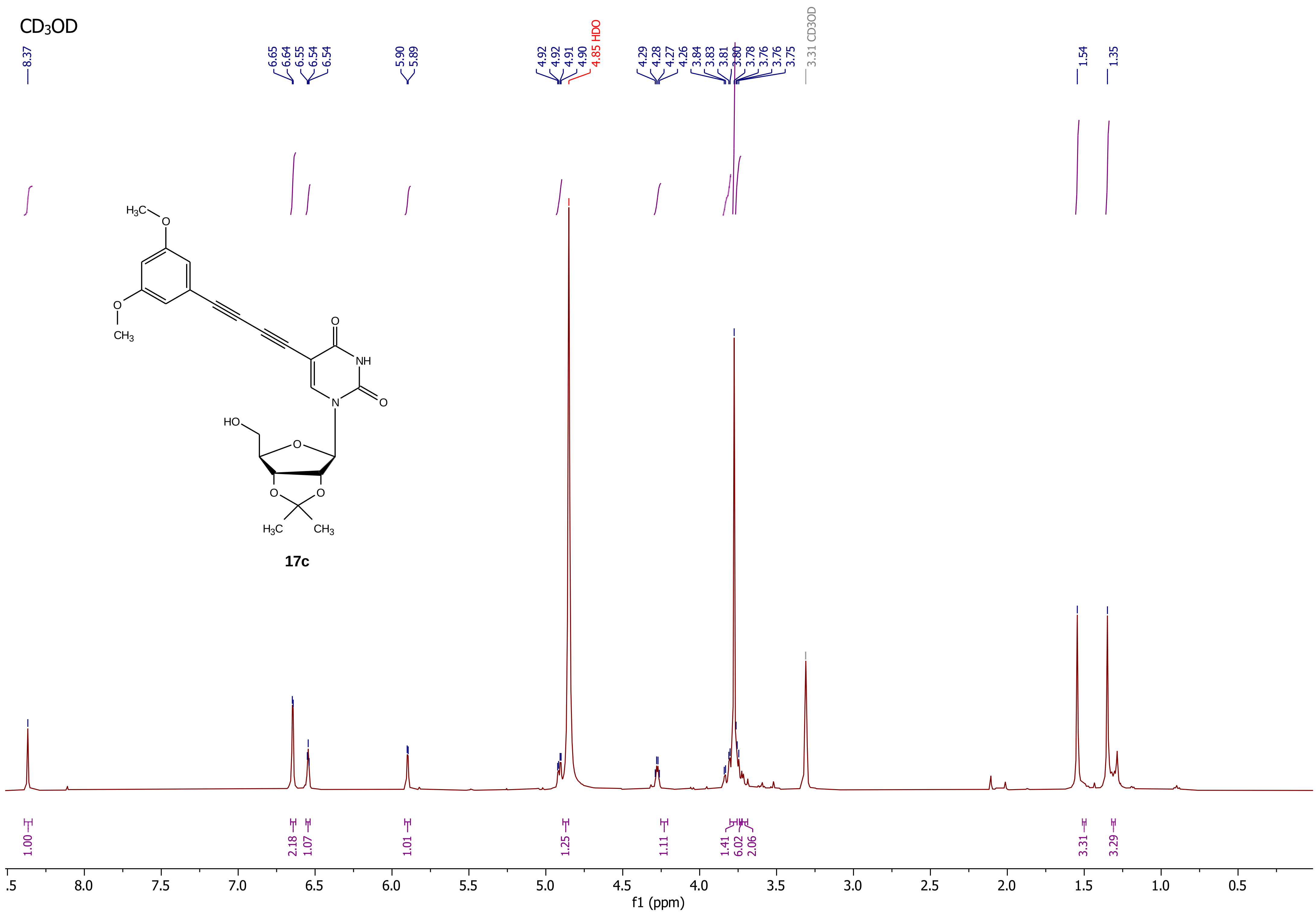

CD3OD
8.37
6.65
6.64
6.55
6.54
6.54
5.90
5.89
4.92
4.92
4.91
4.90
4.85 HDO
4.29
4.28
4.27
4.26
3.84
3.83
3.81
3.80
3.78
3.76
3.76
3.75
3.31 CD3OD
1.54
1.35
H3C
O
O
CH3
O
NH
N
O
HO
O
O
O
H3C
CH3
17c
1.00
2.18
1.07
1.01
1.25
1.11
1.41
6.02
2.06
3.31
3.29
.5
8.0
7.5
7.0
6.5
6.0
5.5
5.0
4.5
4.0
3.5
3.0
2.5
2.0
1.5
1.0
0.5
f1 (ppm)

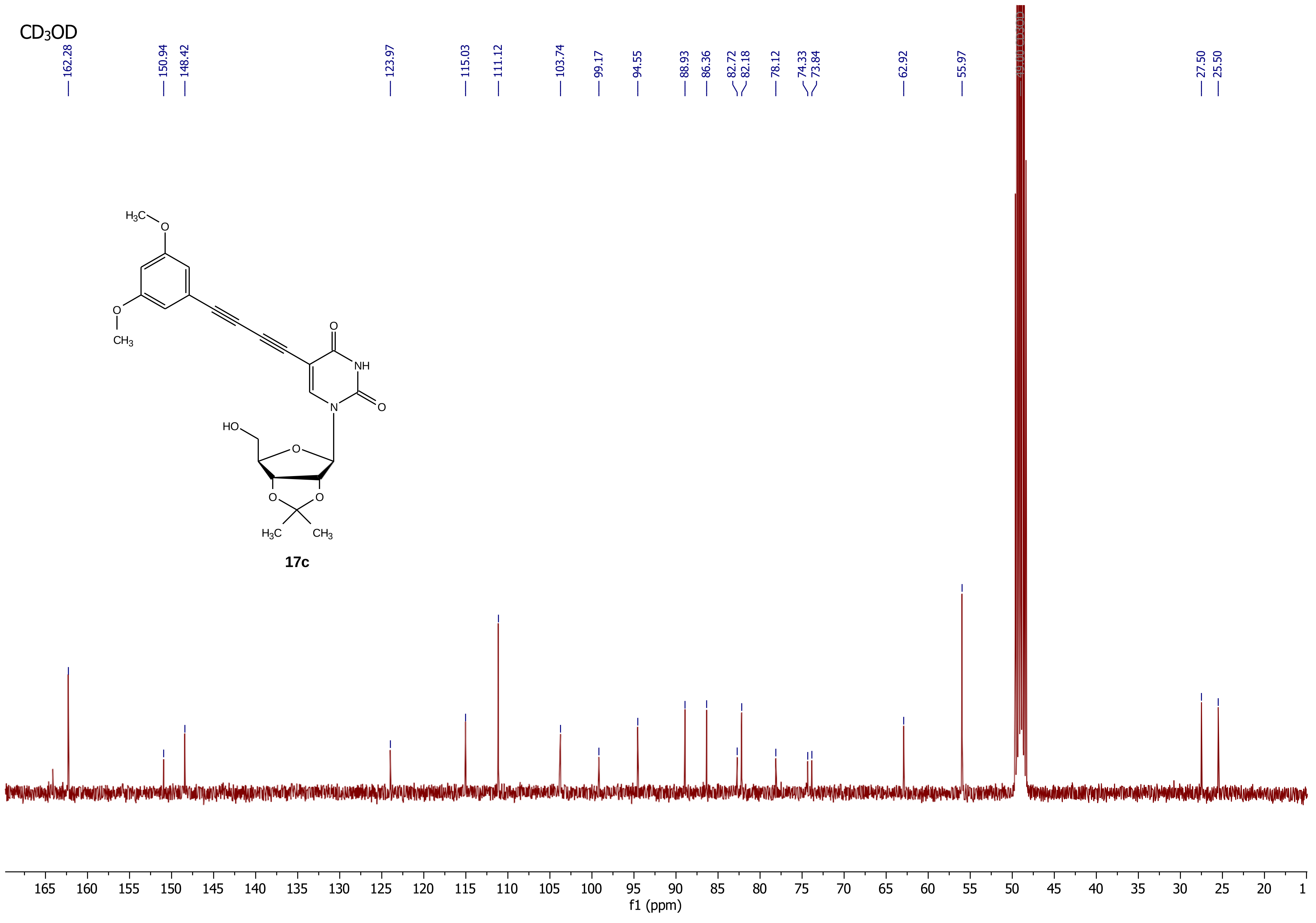
CD3OD
162.28
150.94
148.42
123.97
115.03
111.12
103.74
99.17
94.55
88.93
86.36
82.72
82.18
78.12
74.33
73.84
62.92
55.97
49.00 CD3OD
27.50
25.50
17c
f1 (ppm)

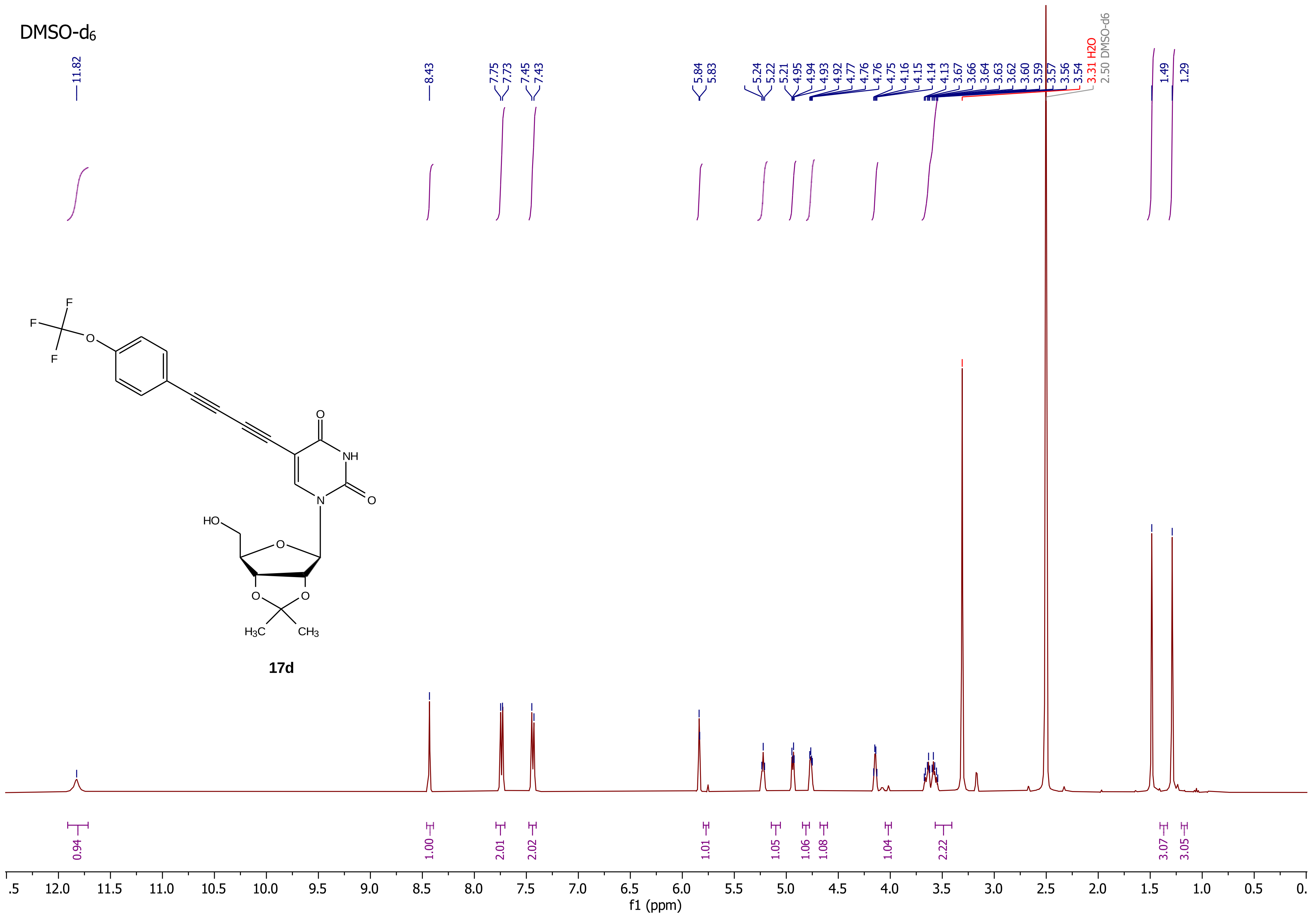
DMSO-d6
11.82
8.43
7.75
7.73
7.45
7.43
5.84
5.83
5.24
5.22
5.21
4.95
4.94
4.93
4.92
4.77
4.76
4.76
4.75
4.16
4.15
4.14
4.13
3.67
3.66
3.64
3.63
3.62
3.60
3.59
3.57
3.56
3.54
3.31 H2O
2.50 DMSO-d6
1.49
1.29
F
F
F
O
O
NH
O
N
HO
O
O
O
H3C
CH3
17d
0.94
1.00
2.01
2.02
1.01
1.05
1.06
1.08
1.04
2.22
3.07
3.05
.5
12.0
11.5
11.0
10.5
10.0
9.5
9.0
8.5
8.0
7.5
7.0
6.5
6.0
5.5
5.0
4.5
4.0
3.5
3.0
2.5
2.0
1.5
1.0
0.5
0.
f1 (ppm)

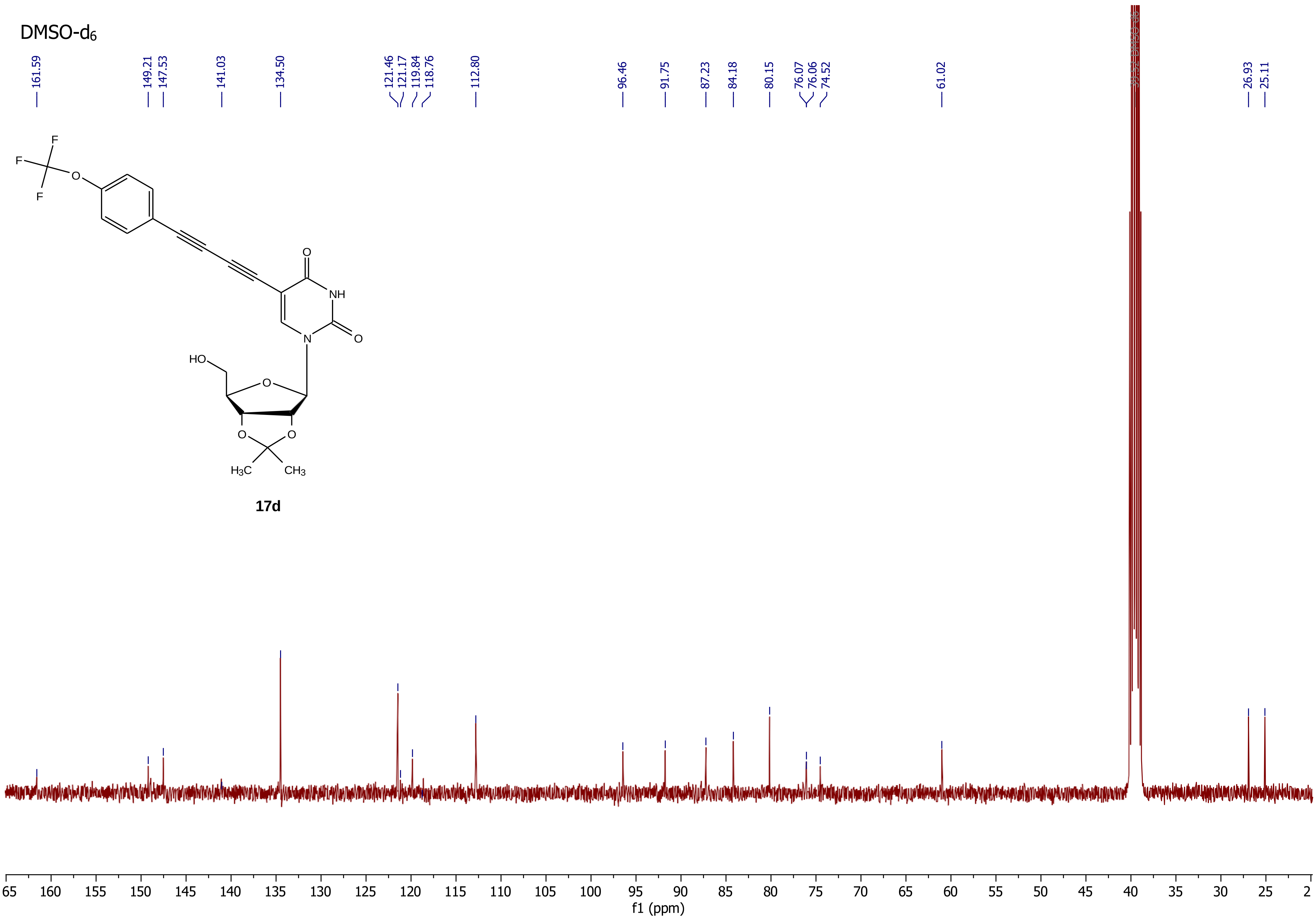
DMSO-d6
161.59
149.21
147.53
141.03
134.50
121.46
121.17
119.84
118.76
112.80
96.46
91.75
87.23
84.18
80.15
76.07
76.06
74.52
61.02
26.93
25.11
F
F
F
O
O
NH
N
O
HO
O
O
O
H3C
CH3
17d
f1 (ppm)

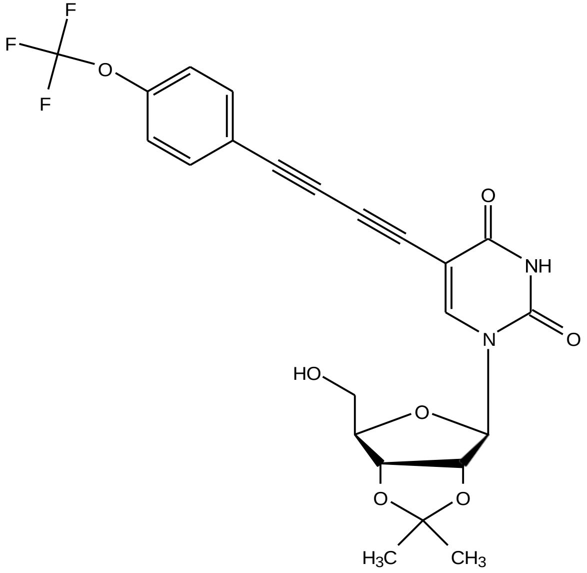
F
F
O
F
O
NH
O
N
HO
O
O
O
H3C
CH3

17d

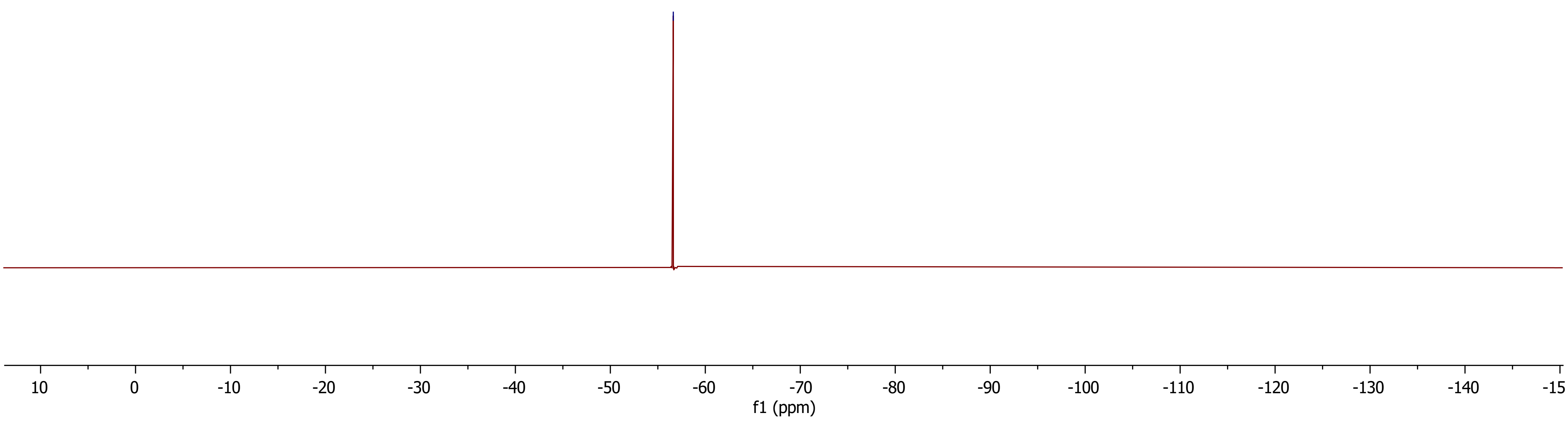
DMSO-d6
-56.72
10
0
-10
-20
-30
-40
-50
-60
-70
-80
-90
-100
-110
-120
-130
-140
-15
f1 (ppm)

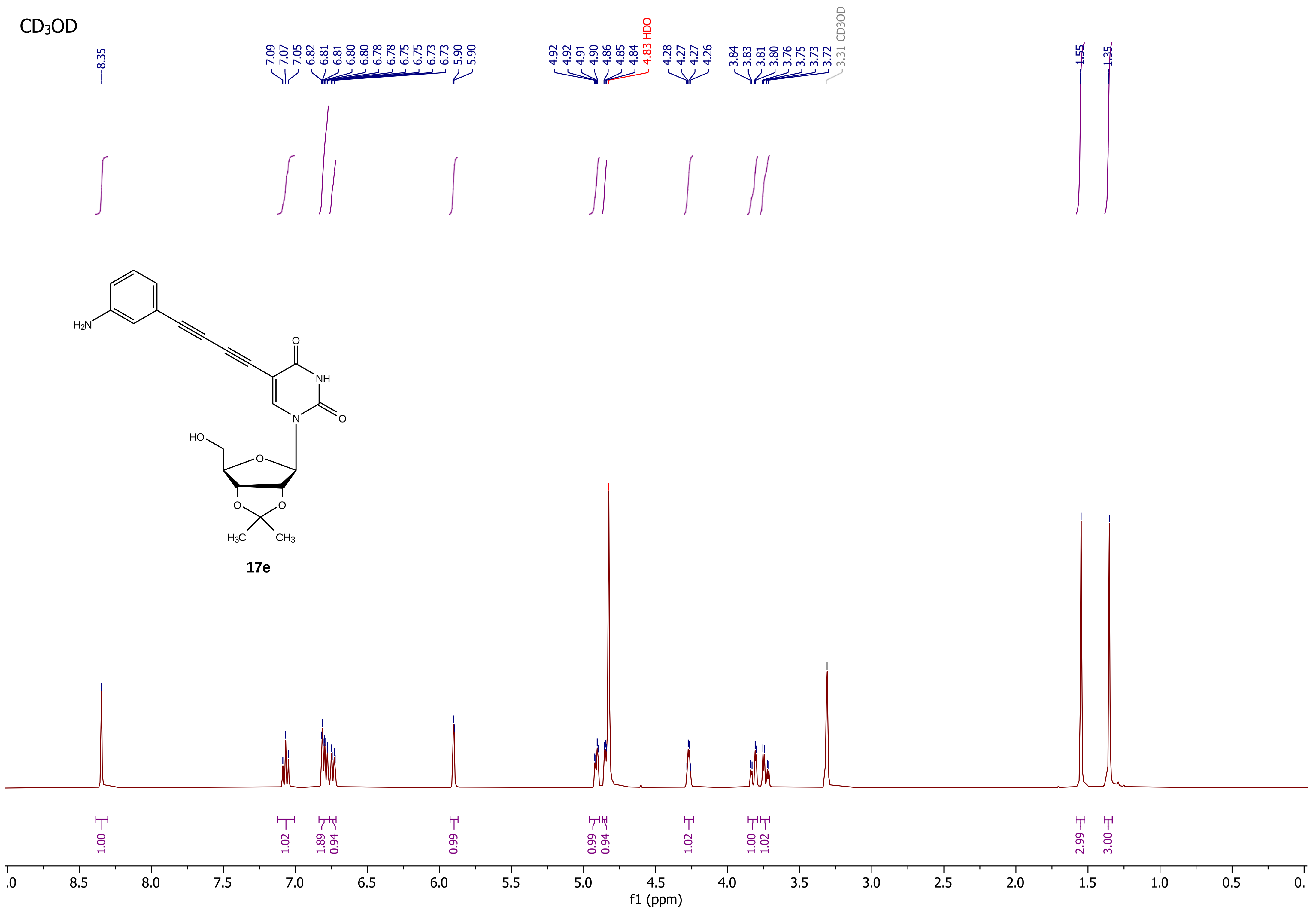
CD3OD
8.35
7.09 7.07 7.05 6.82 6.81 6.81 6.80 6.80 6.78 6.78 6.75 6.75 6.73 6.73 5.90 5.90
4.92 4.92 4.91 4.90 4.86 4.85 4.84 4.83 HDO
4.28 4.27 4.27 4.26
3.84 3.83 3.81 3.80 3.76 3.75 3.73 3.72 3.31 CD3OD
1.55 1.35
H2N
NH
O
N
HO
H3C CH3
17e
1.00 1.02 1.89 0.94 0.99 0.99 0.94 1.02 1.00 1.02 2.99 3.00
f1 (ppm)

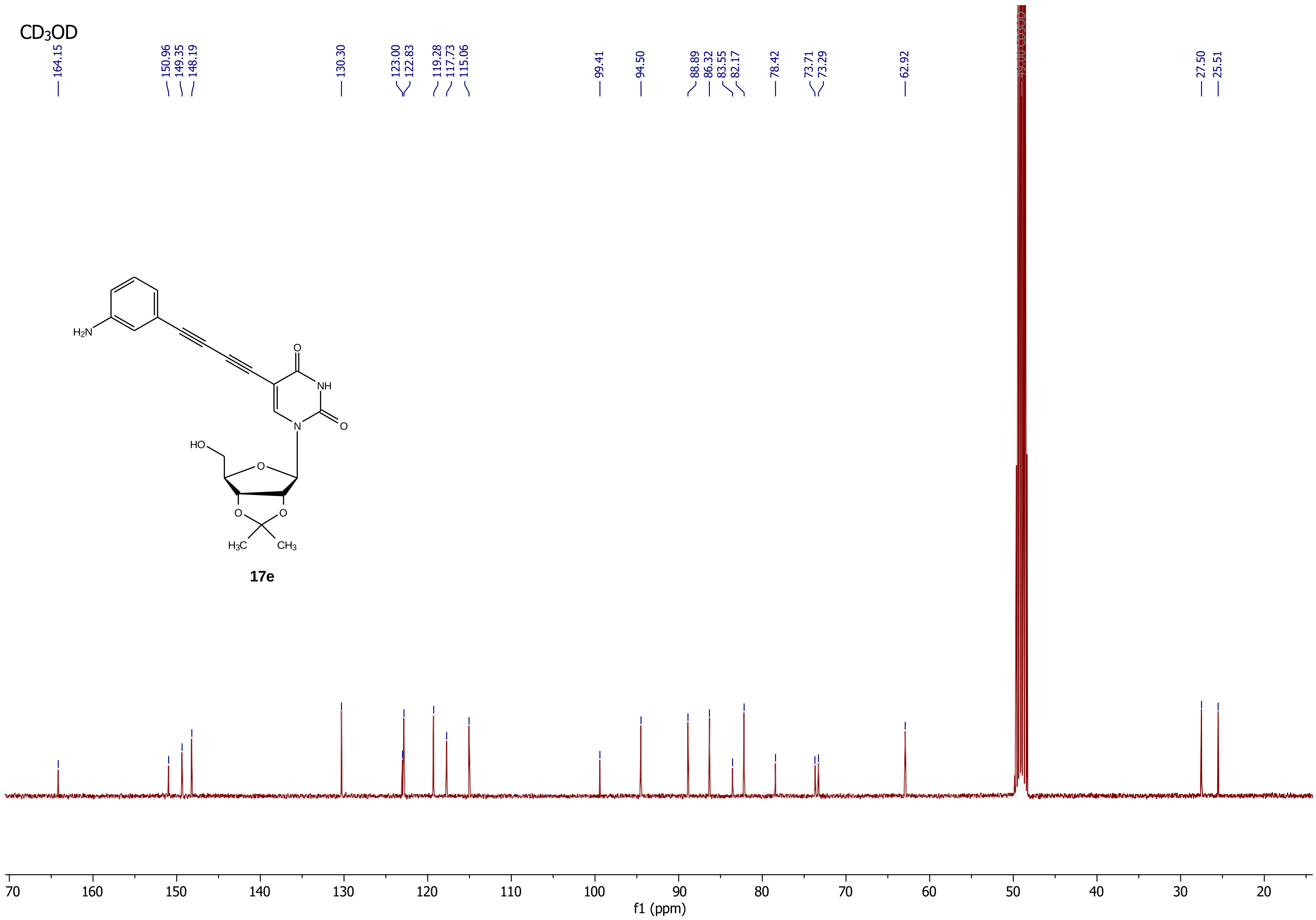

CD3OD
164.15
150.96
149.35
148.19
130.30
123.00
122.83
119.28
117.73
115.06
99.41
94.50
88.89
86.32
83.55
82.17
78.42
73.71
73.29
62.92
49.00 CD3OD
27.50
25.51
H2N
O
NH
O
N
HO
O
O
O
H3C
CH3
17e
70
160
150
140
130
120
110
100
90
80
70
60
50
40
30
20
f1 (ppm)

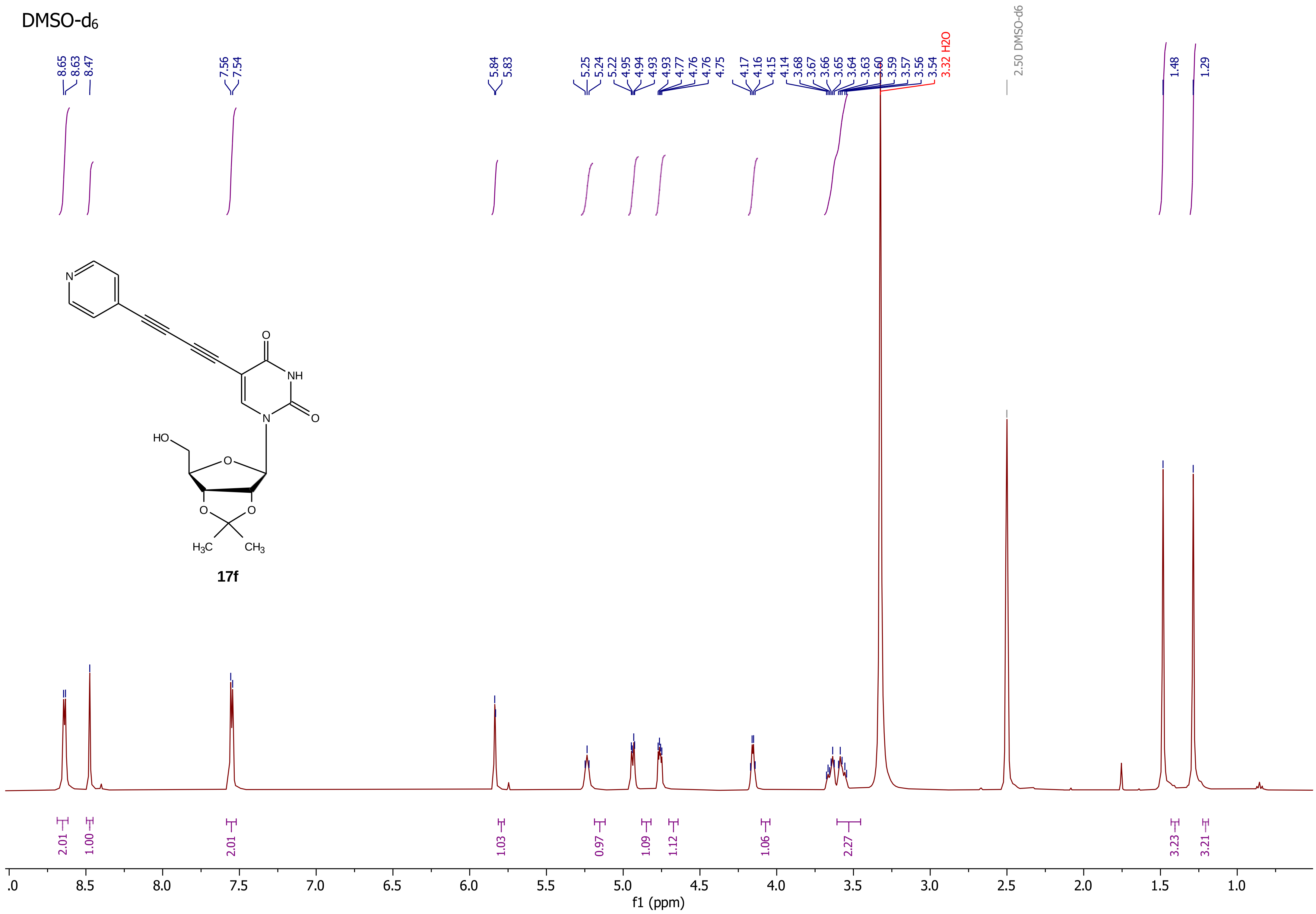
DMSO-d6
8.65
8.63
8.47
7.56
7.54
5.84
5.83
5.25
5.24
5.22
4.95
4.94
4.93
4.93
4.77
4.76
4.76
4.75
4.17
4.16
4.15
4.14
3.68
3.67
3.66
3.65
3.64
3.63
3.60
3.59
3.57
3.56
3.54
3.32 H2O
2.50 DMSO-d6
1.48
1.29
N
O
NH
O
N
HO
O
O
O
H3C
CH3
2.01
1.00
2.01
1.03
0.97
1.09
1.12
1.06
2.27
3.23
3.21
f1 (ppm)

17f

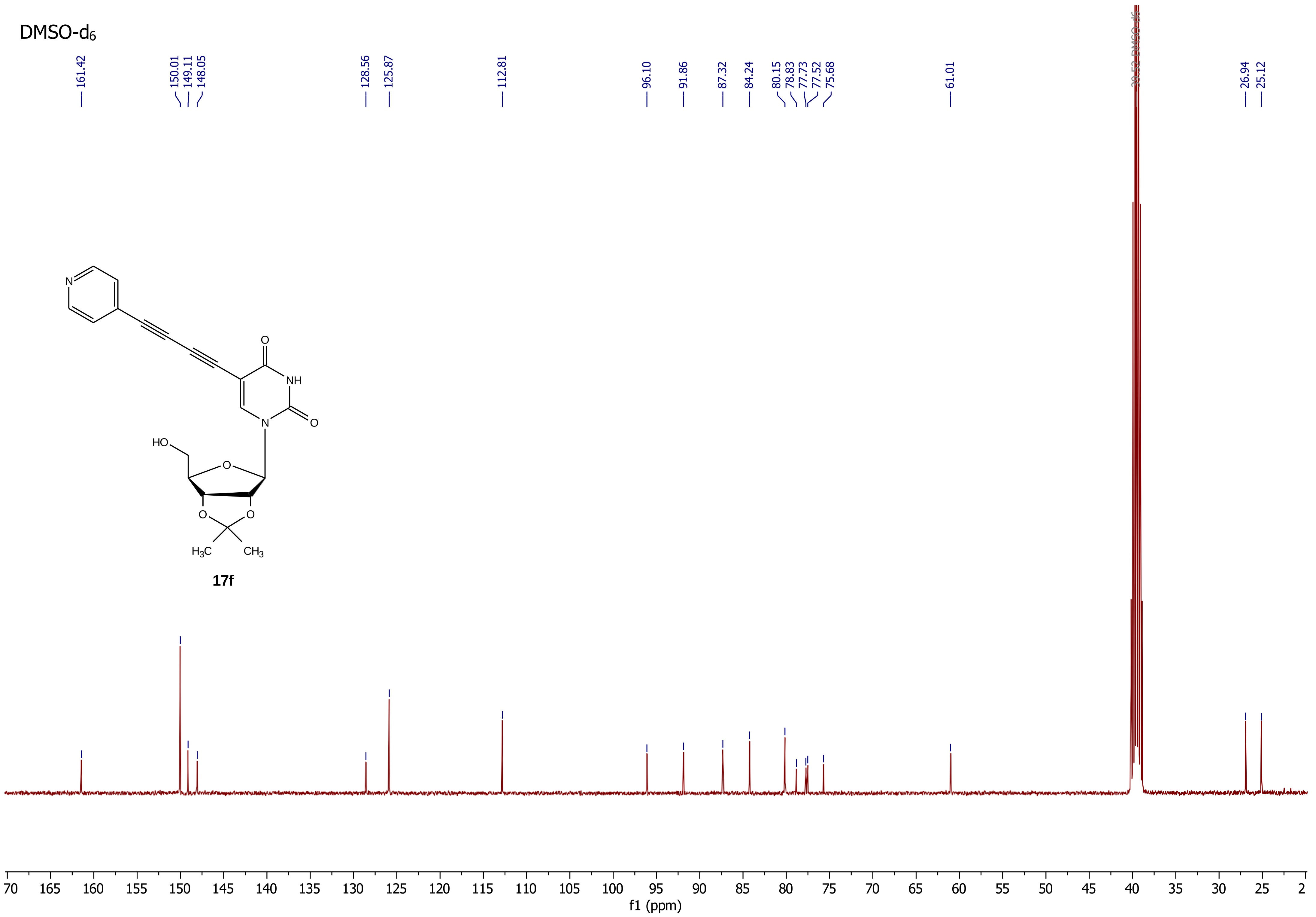
DMSO-d6
161.42
150.01
149.11
148.05
128.56
125.87
112.81
96.10
91.86
87.32
84.24
80.15
78.83
77.73
77.52
75.68
61.01
39.52 DMSO-d6
26.94
25.12
N
O
NH
N
O
HO
O
O
O
H3C
CH3
17f
f1 (ppm)

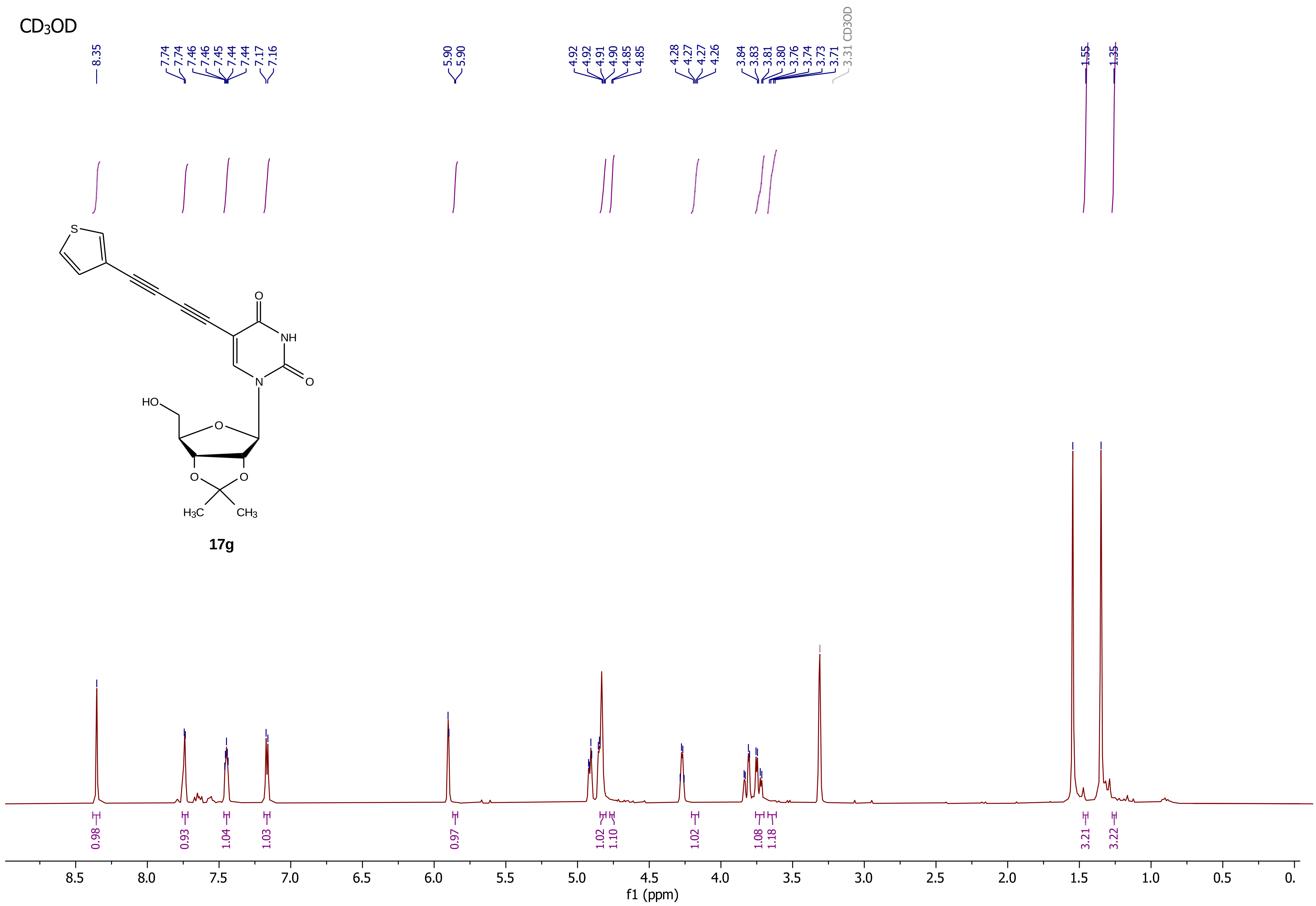
CD3OD
8.35
7.74
7.74
7.46
7.46
7.45
7.44
7.44
7.17
7.16
5.90
5.90
4.92
4.92
4.91
4.90
4.85
4.85
4.28
4.27
4.27
4.26
3.84
3.83
3.81
3.80
3.76
3.74
3.73
3.71
3.31 CD3OD
1.55
1.35
S
NH
O
N
HO
H3C
CH3
17g
0.98
0.93
1.04
1.03
0.97
1.02
1.10
1.02
1.08
1.18
3.21
3.22
8.5
8.0
7.5
7.0
6.5
6.0
5.5
5.0
4.5
4.0
3.5
3.0
2.5
2.0
1.5
1.0
0.5
0.
f1 (ppm)

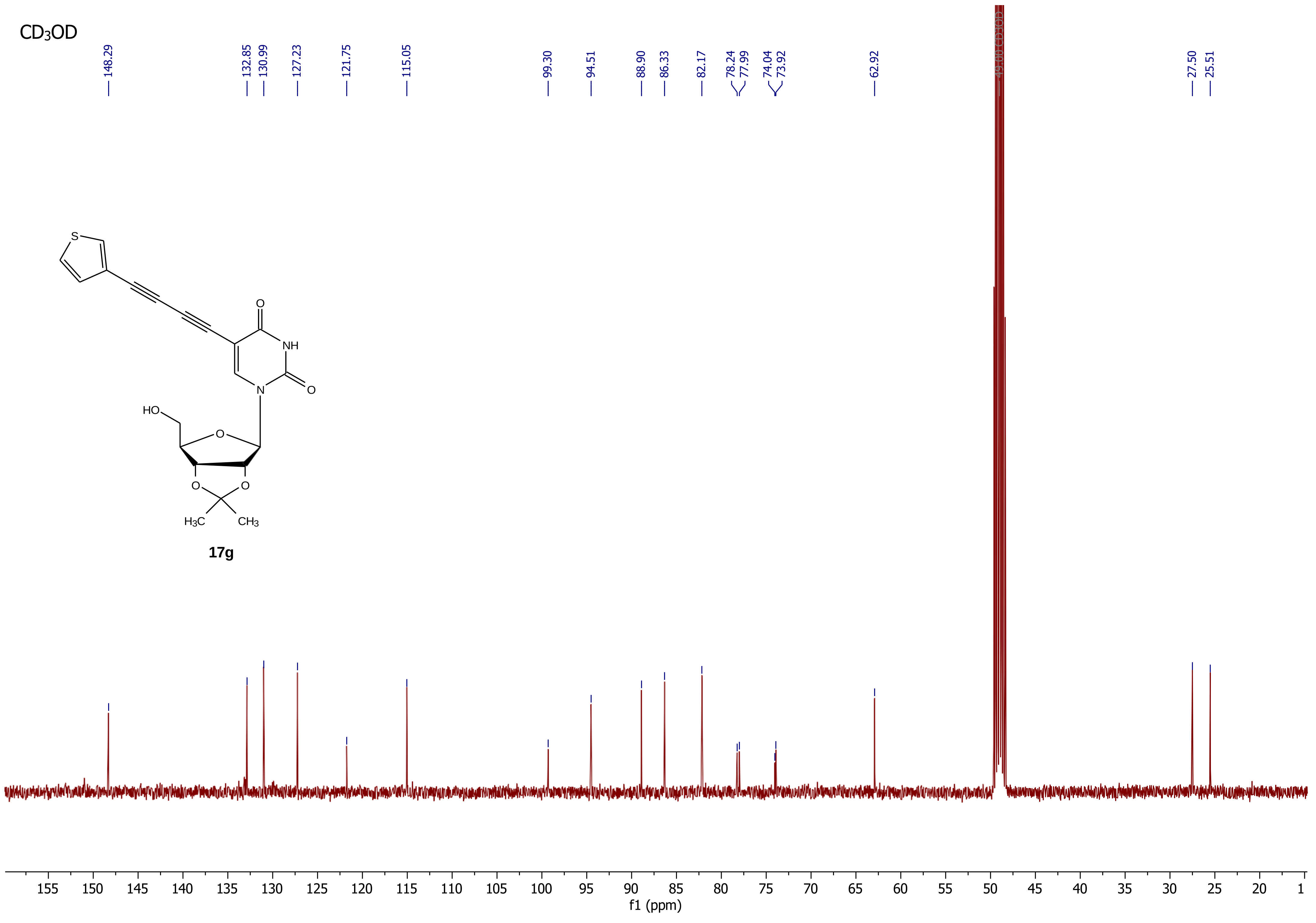
CD3OD
148.29
132.85
130.99
127.23
121.75
115.05
99.30
94.51
88.90
86.33
82.17
78.24
77.99
74.04
73.92
62.92
49.00 CD3OD
27.50
25.51
S
O
NH
N
HO
H3C
CH3
17g
155 150 145 140 135 130 125 120 115 110 105 100 95 90 85 80 75 70 65 60 55 50 45 40 35 30 25 20 1
f1 (ppm)

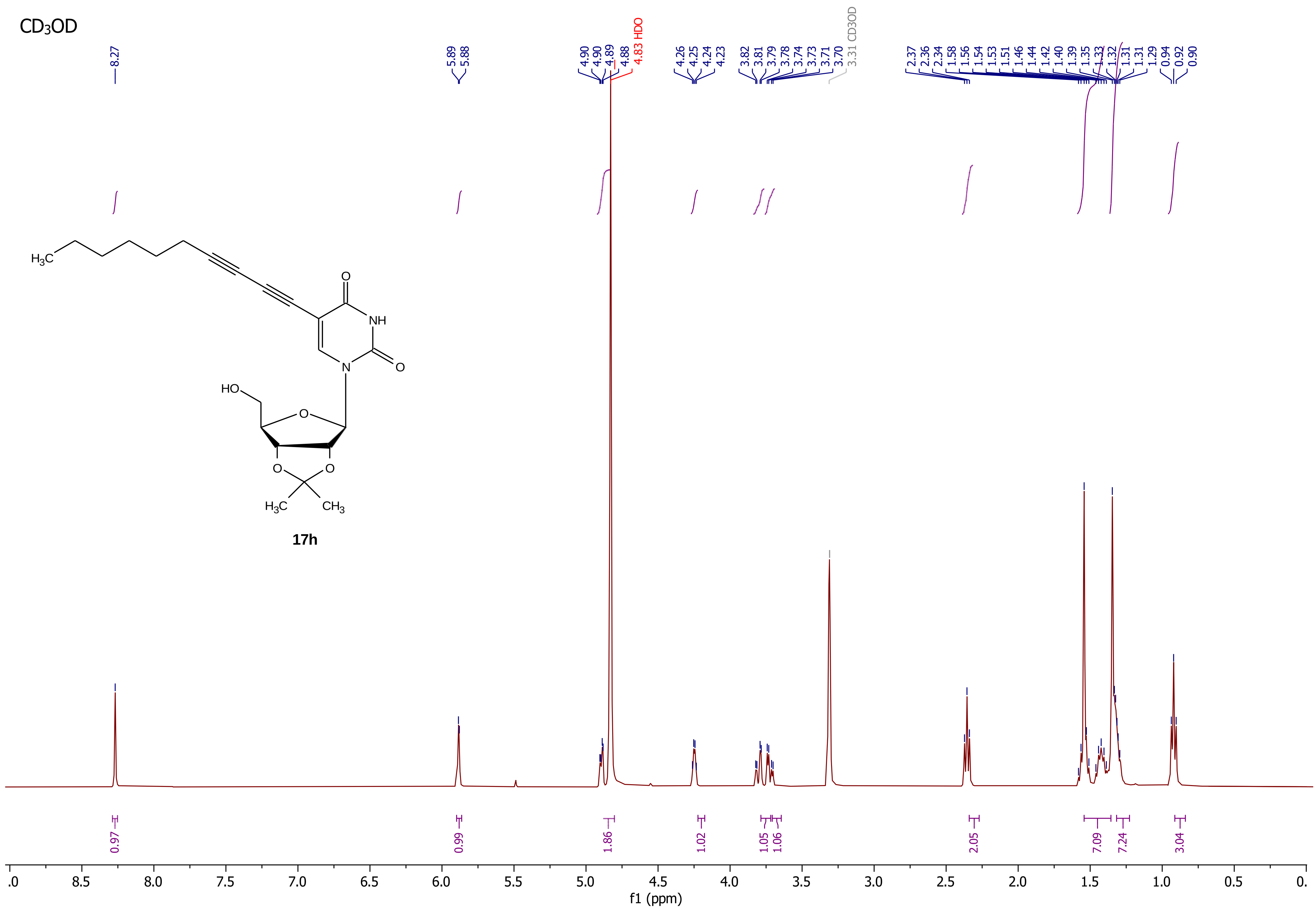

CD3OD
8.27
5.89
5.88
4.90
4.90
4.89
4.88
4.83 HDO
4.26
4.25
4.24
4.23
3.82
3.81
3.79
3.78
3.74
3.73
3.71
3.70
3.31 CD3OD
2.37
2.36
2.34
1.58
1.56
1.54
1.53
1.51
1.46
1.44
1.42
1.40
1.39
1.35
1.33
1.32
1.31
1.31
1.29
0.94
0.92
0.90
H_3C
O
NH
N
O
HO
O
O
O
H_3C
CH_3
0.97
0.99
1.86
1.02
1.05
1.06
2.05
7.09
7.24
3.04
.0
8.5
8.0
7.5
7.0
6.5
6.0
5.5
5.0
4.5
4.0
3.5
3.0
2.5
2.0
1.5
1.0
0.5
0.
f1 (ppm)

17h

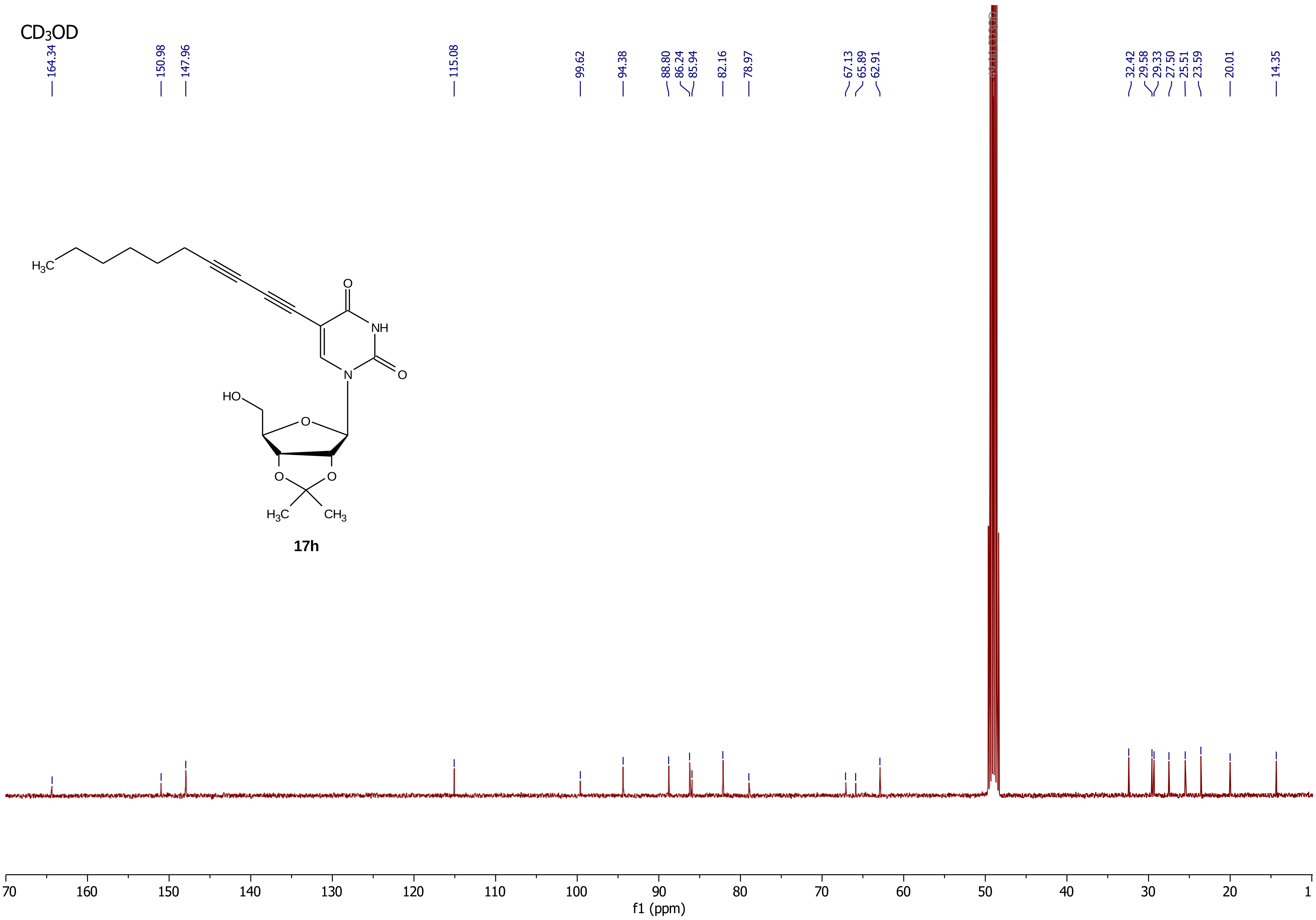
CD3OD
164.34
150.98
147.96
115.08
99.62
94.38
88.80
86.24
85.94
82.16
78.97
67.13
65.89
62.91
32.42
29.58
29.33
27.50
25.51
23.59
20.01
14.35
H3C
O
NH
O
N
HO
O
O
O
H3C
CH3
17h
f1 (ppm)

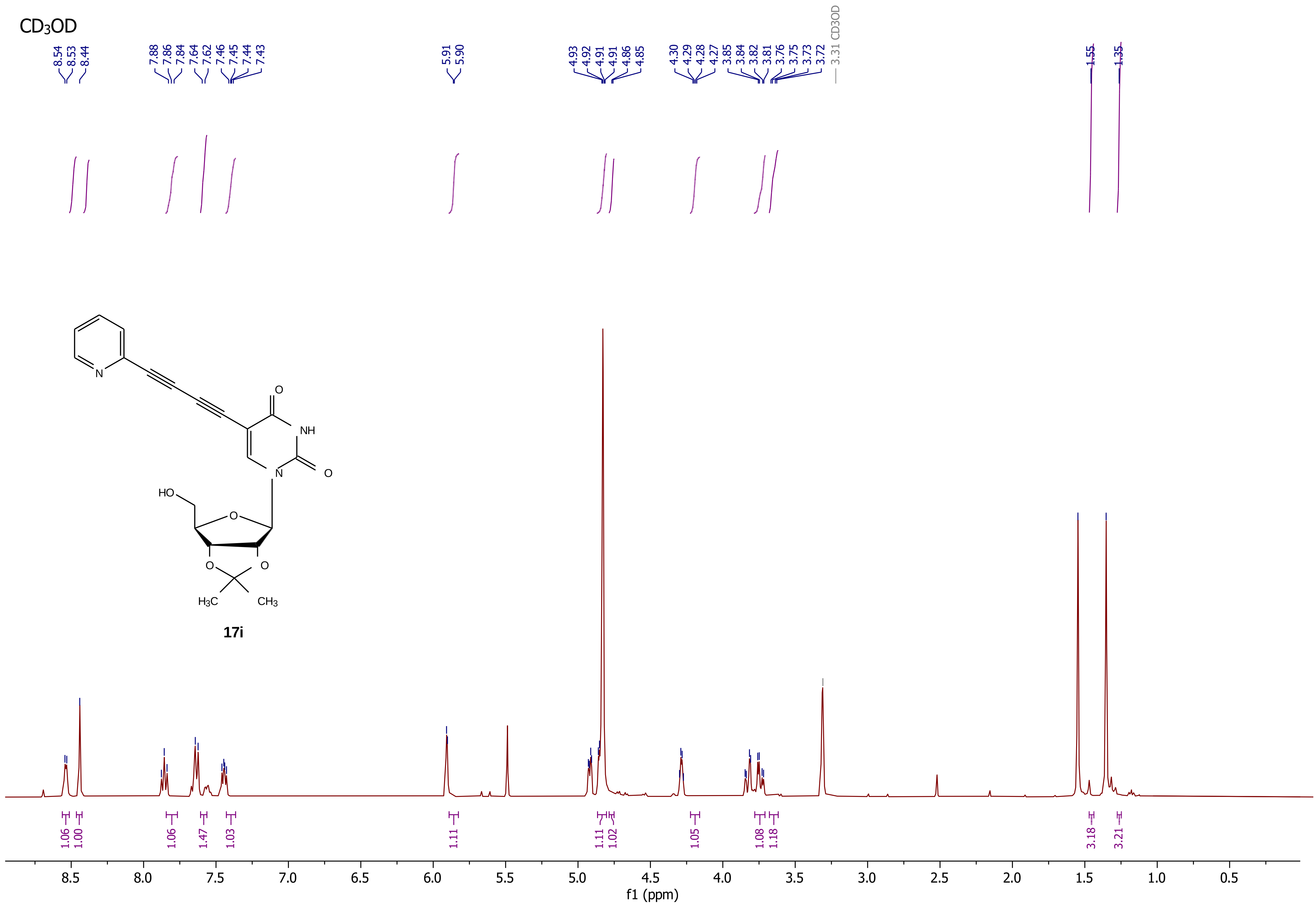
CD3OD
8.54
8.53
8.44
7.88
7.86
7.84
7.64
7.62
7.46
7.45
7.44
7.43
5.91
5.90
4.93
4.92
4.91
4.91
4.86
4.85
4.30
4.29
4.28
4.27
3.85
3.84
3.82
3.81
3.76
3.75
3.73
3.72
3.31 CD3OD
1.55
1.35
1.06
1.00
1.06
1.47
1.03
1.11
1.11
1.02
1.05
1.08
1.18
3.18
3.21
8.5
8.0
7.5
7.0
6.5
6.0
5.5
5.0
4.5
4.0
3.5
3.0
2.5
2.0
1.5
1.0
0.5
f1 (ppm)
N
O
NH
O
N
HO
O
O
O
H3C
CH3

17i

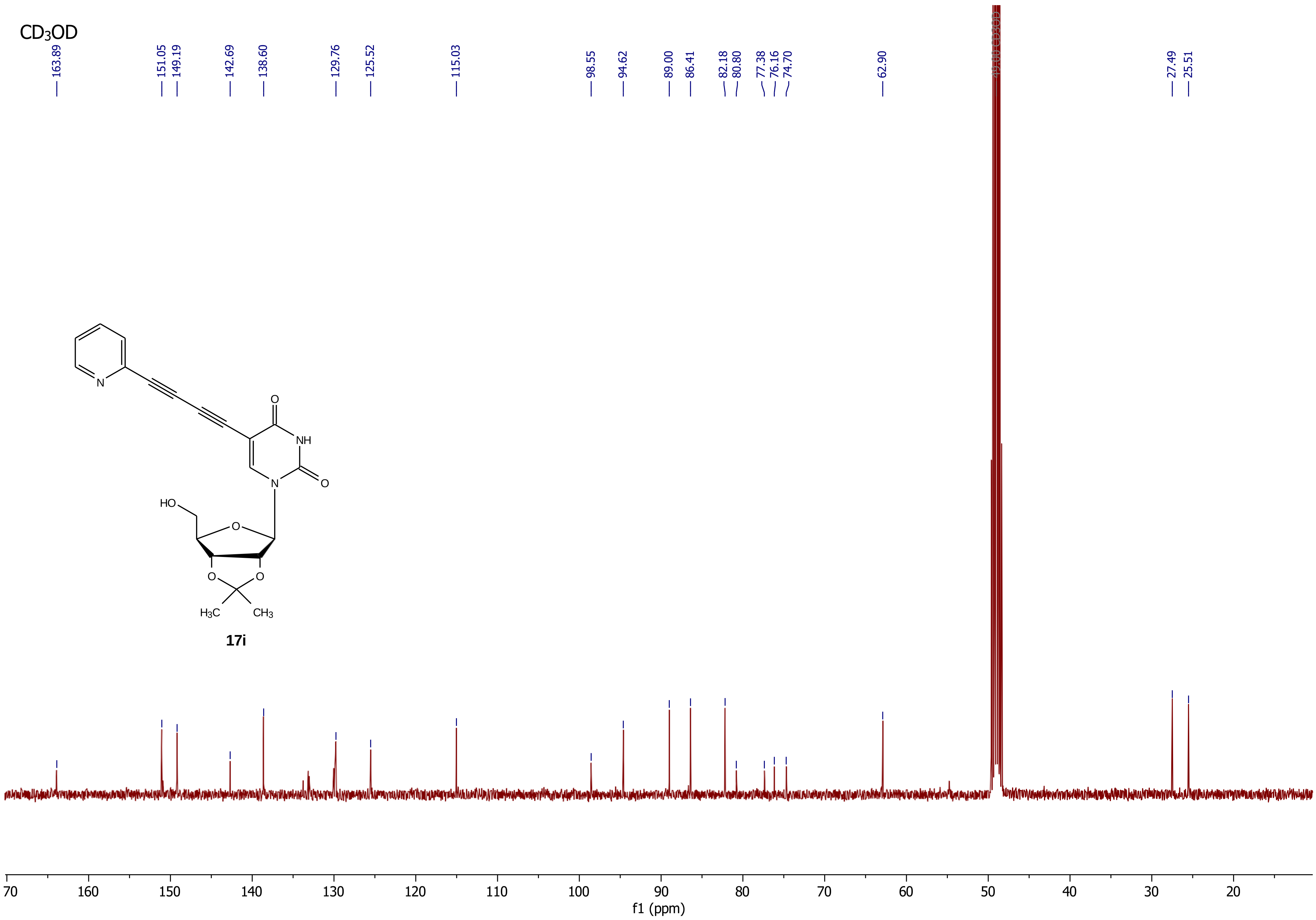
CD3OD
163.89
151.05
149.19
142.69
138.60
129.76
125.52
115.03
98.55
94.62
89.00
86.41
82.18
80.80
77.38
76.16
74.70
62.90
49.00 CD3OD
27.49
25.51
HO
O
N
NH
H3C
CH3
17i
70
160
150
140
130
120
110
100
90
80
70
60
50
40
30
20
f1 (ppm)

CD_3OD

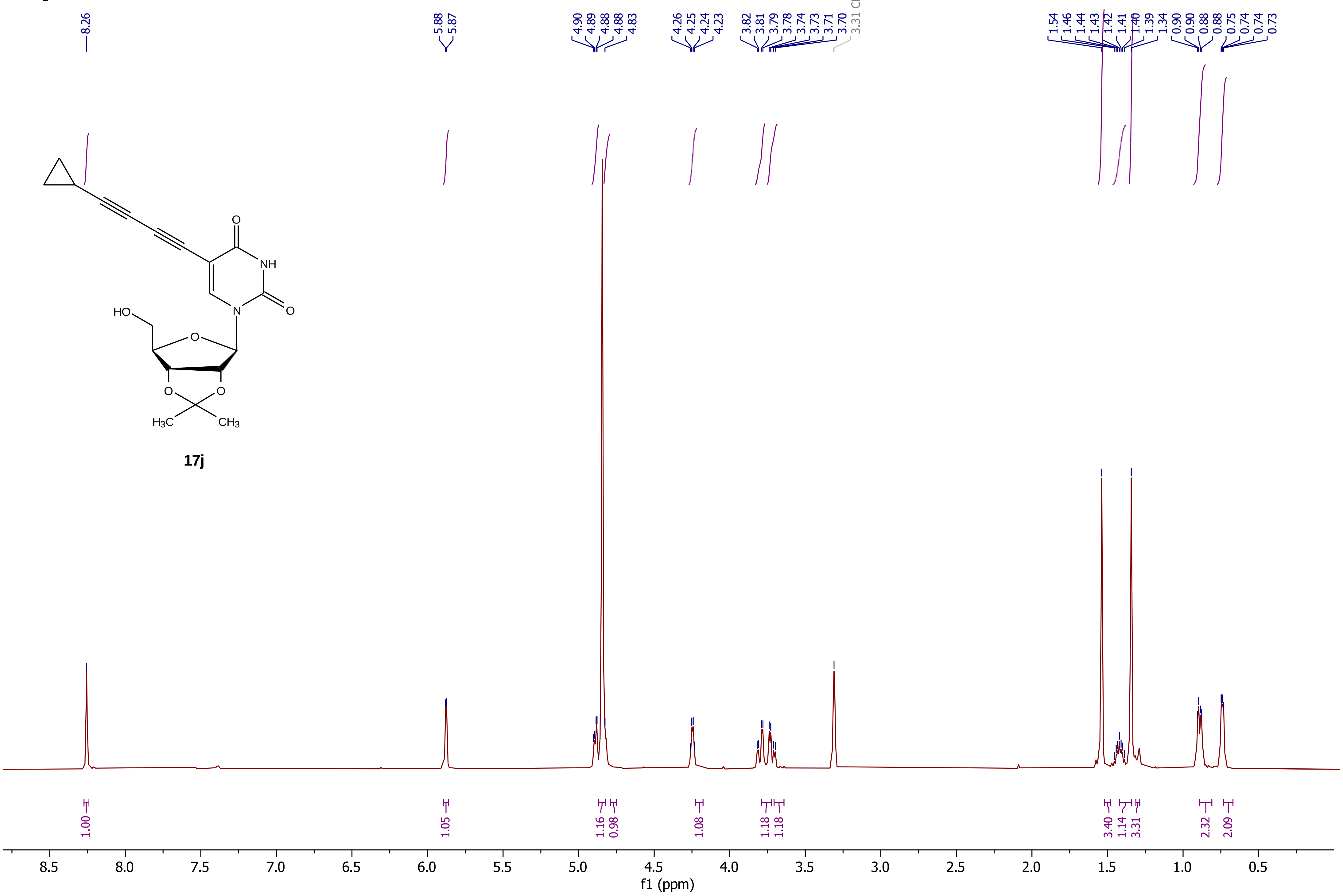

8.26
5.88
5.87
4.90
4.89
4.88
4.88
4.83
4.26
4.25
4.24
4.23
3.82
3.81
3.79
3.78
3.74
3.73
3.71
3.70
3.31 CD3OD
1.54
1.46
1.44
1.43
1.42
1.41
1.40
1.39
1.34
0.90
0.90
0.88
0.88
0.75
0.74
0.74
0.73
HO
O
N
NH
O
O
O
O
H3C
CH3
1.00
1.05
1.16
0.98
1.08
1.18
1.18
3.40
1.14
3.31
2.32
2.09
8.5
8.0
7.5
7.0
6.5
6.0
5.5
5.0
4.5
4.0
3.5
3.0
2.5
2.0
1.5
1.0
0.5
f1 (ppm)

17j

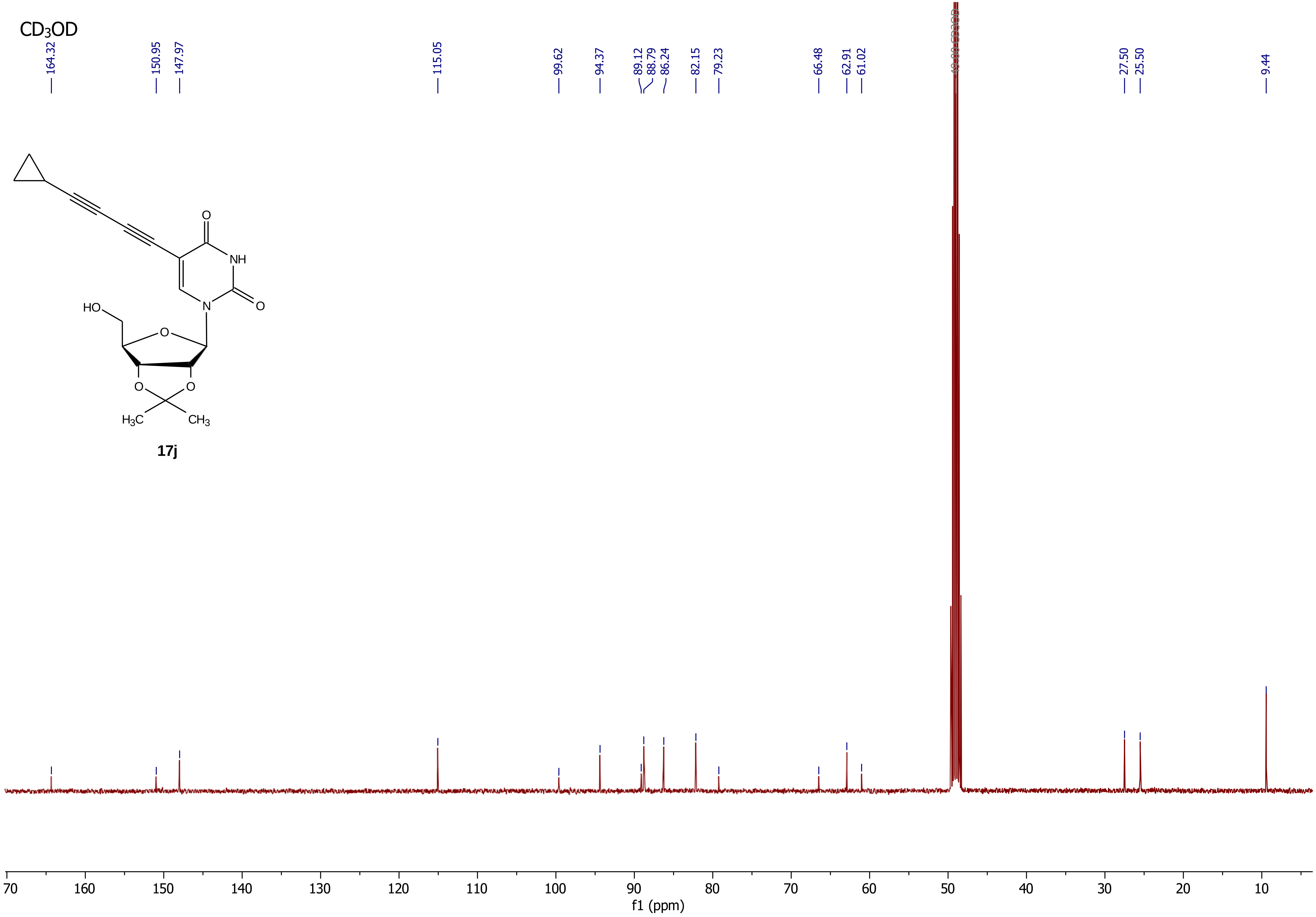

CD3OD
164.32
150.95
147.97
115.05
99.62
94.37
89.12
88.79
86.24
82.15
79.23
66.48
62.91
61.02
27.50
25.50
9.44
HO
O
NH
N
H3C
CH3
17j
70
160
150
140
130
120
110
100
90
80
70
60
50
40
30
20
10
f1 (ppm)

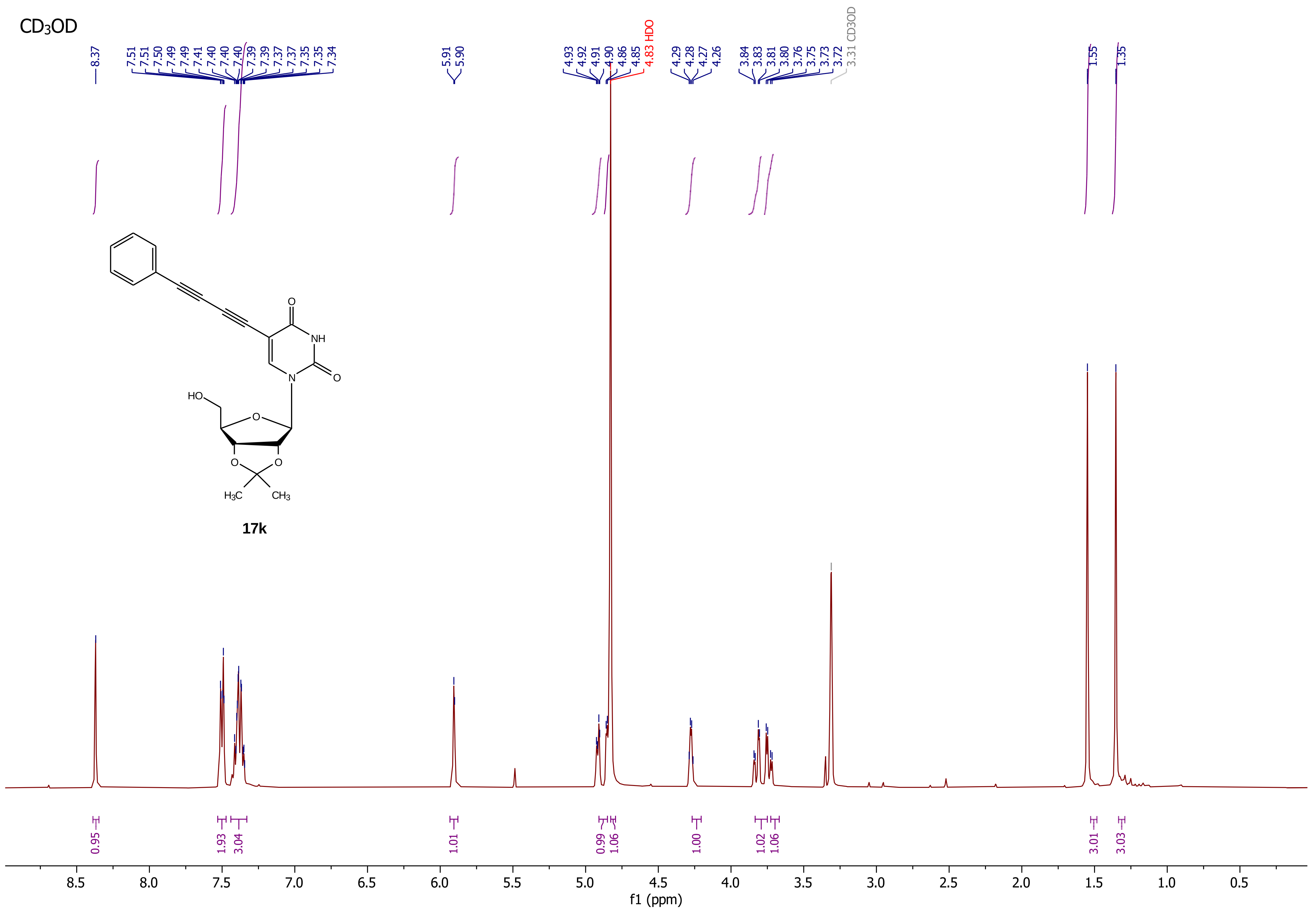
CD3OD
8.37
7.51
7.51
7.50
7.49
7.49
7.41
7.40
7.40
7.40
7.39
7.39
7.37
7.37
7.35
7.35
7.34
5.91
5.90
4.93
4.92
4.91
4.90
4.86
4.85
4.83 HDO
4.29
4.28
4.27
4.26
3.84
3.83
3.81
3.80
3.76
3.75
3.73
3.72
3.31 CD3OD
1.55
1.35
HO
O
N
NH
O
O
O
O
H3C
CH3
17k
0.95
1.93
3.04
1.01
0.99
1.06
1.00
1.02
1.06
3.01
3.03
8.5
8.0
7.5
7.0
6.5
6.0
5.5
5.0
4.5
4.0
3.5
3.0
2.5
2.0
1.5
1.0
0.5
f1 (ppm)

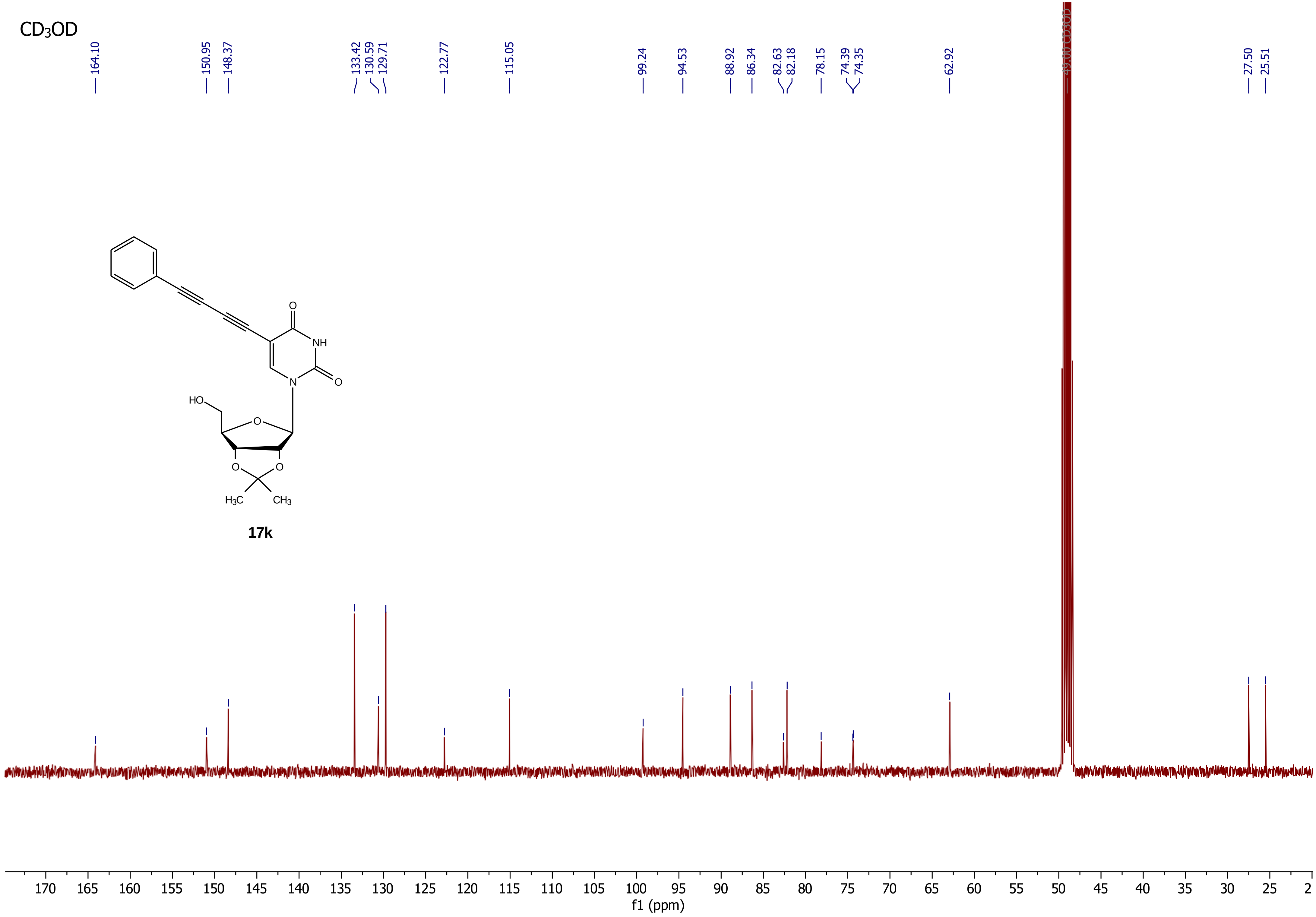
CD3OD
164.10
150.95
148.37
133.42
130.59
129.71
122.77
115.05
99.24
94.53
88.92
86.34
82.63
82.18
78.15
74.39
74.35
62.92
49.00 CD3OD
27.50
25.51
HO
O
NH
N
H3C
CH3
17k
170
165
160
155
150
145
140
135
130
125
120
115
110
105
100
95
90
85
80
75
70
65
60
55
50
45
40
35
30
25
2
f1 (ppm)

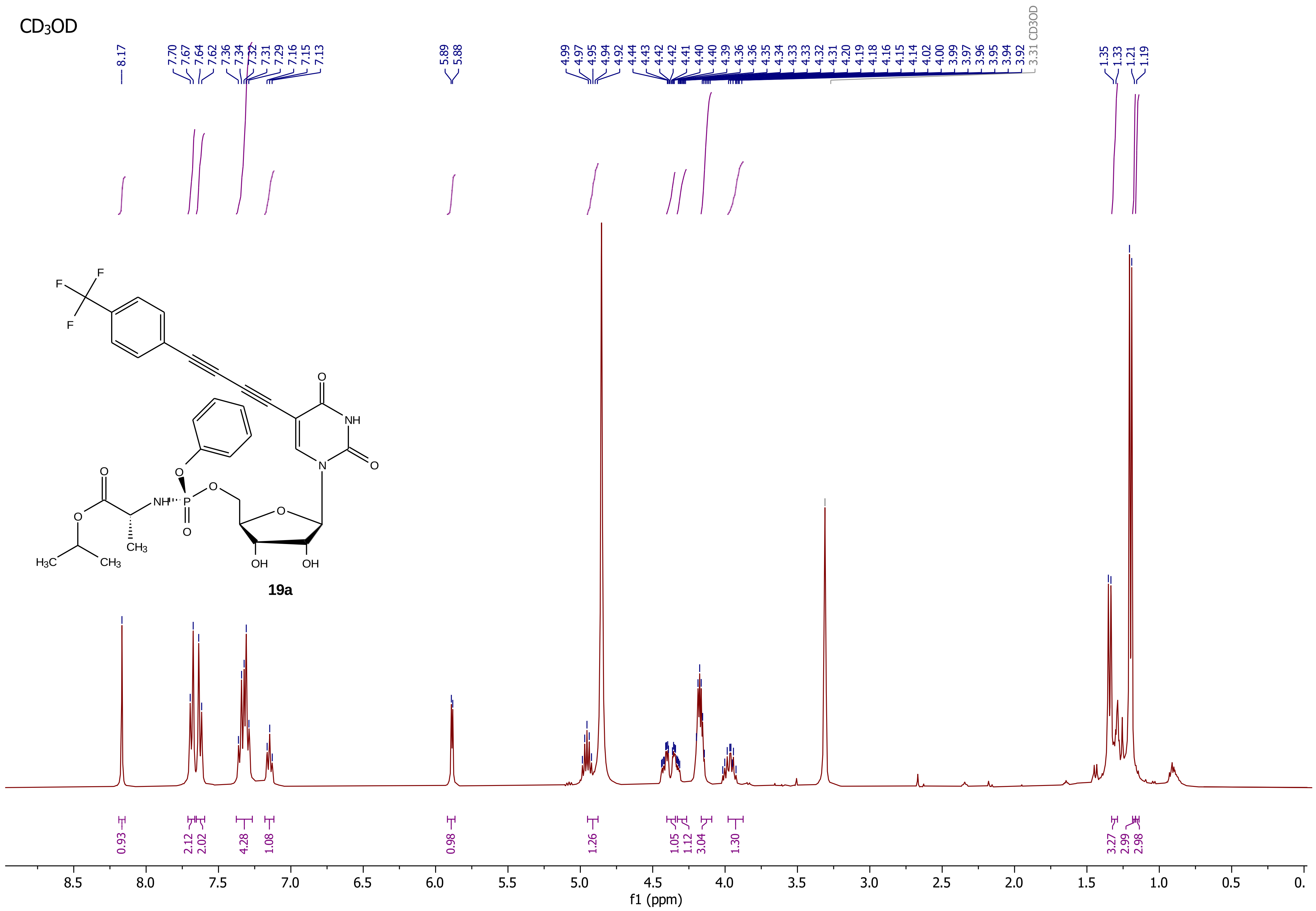
CD3OD
8.17
7.70
7.67
7.64
7.62
7.36
7.34
7.32
7.31
7.29
7.16
7.15
7.13
5.89
5.88
4.99
4.97
4.95
4.94
4.92
4.44
4.43
4.42
4.42
4.41
4.40
4.40
4.39
4.36
4.36
4.35
4.34
4.33
4.33
4.32
4.31
4.20
4.19
4.18
4.16
4.15
4.14
4.02
4.00
3.99
3.97
3.96
3.95
3.94
3.92
3.31 CD3OD
1.35
1.33
1.21
1.19
19a
0.93
2.12
2.02
4.28
1.08
0.98
1.26
1.05
1.12
3.04
1.30
3.27
2.99
2.98
8.5
8.0
7.5
7.0
6.5
6.0
5.5
5.0
4.5
4.0
3.5
3.0
2.5
2.0
1.5
1.0
0.5
0.
f1 (ppm)

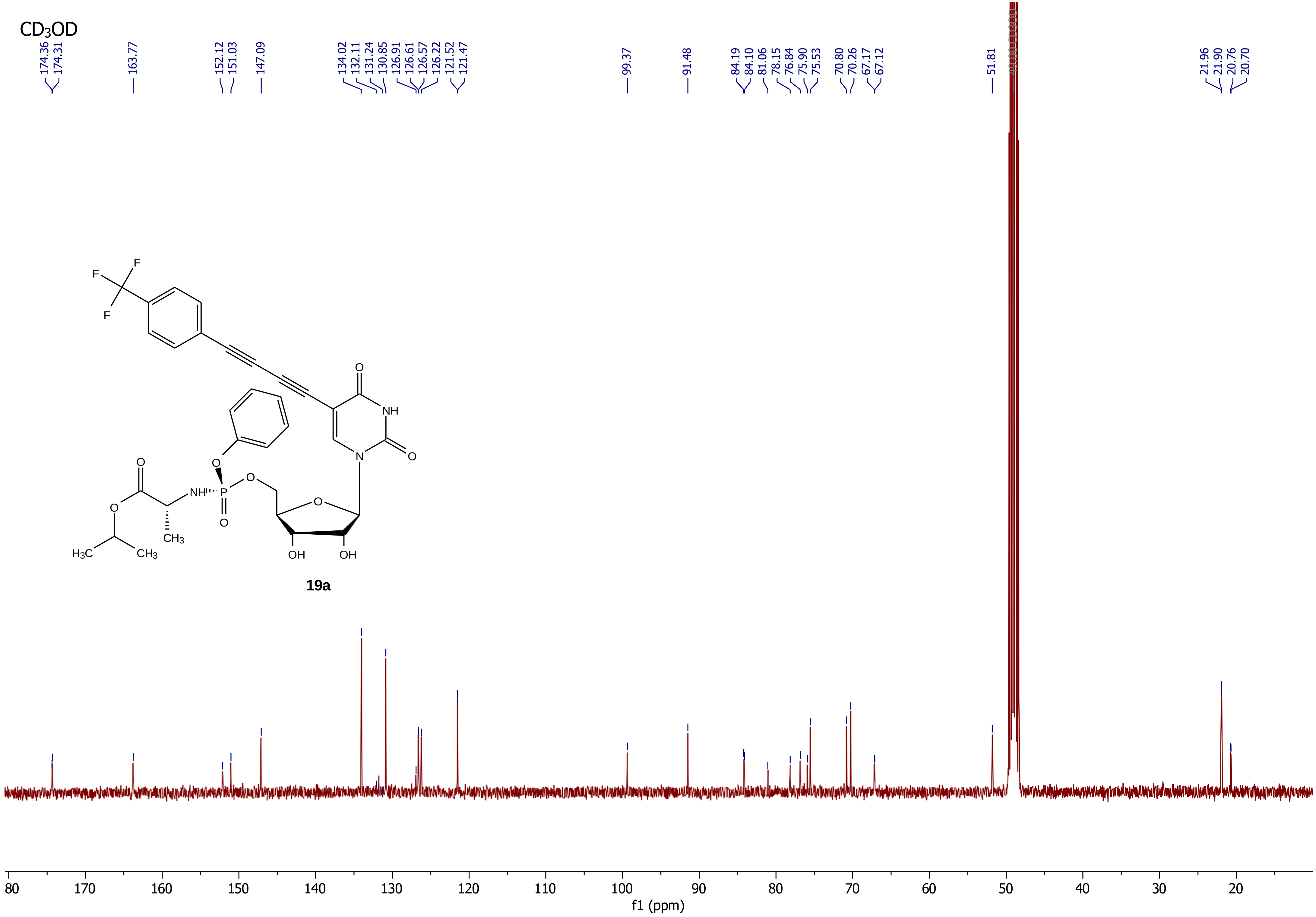
CD3OD
174.36
174.31
163.77
152.12
151.03
147.09
134.02
132.11
131.24
130.85
126.91
126.61
126.57
126.22
121.52
121.47
99.37
91.48
84.19
84.10
81.06
78.15
76.84
75.90
75.53
70.80
70.26
67.17
67.12
51.81
49.00 CD3OD
21.96
21.90
20.76
20.70
f1 (ppm)

19a

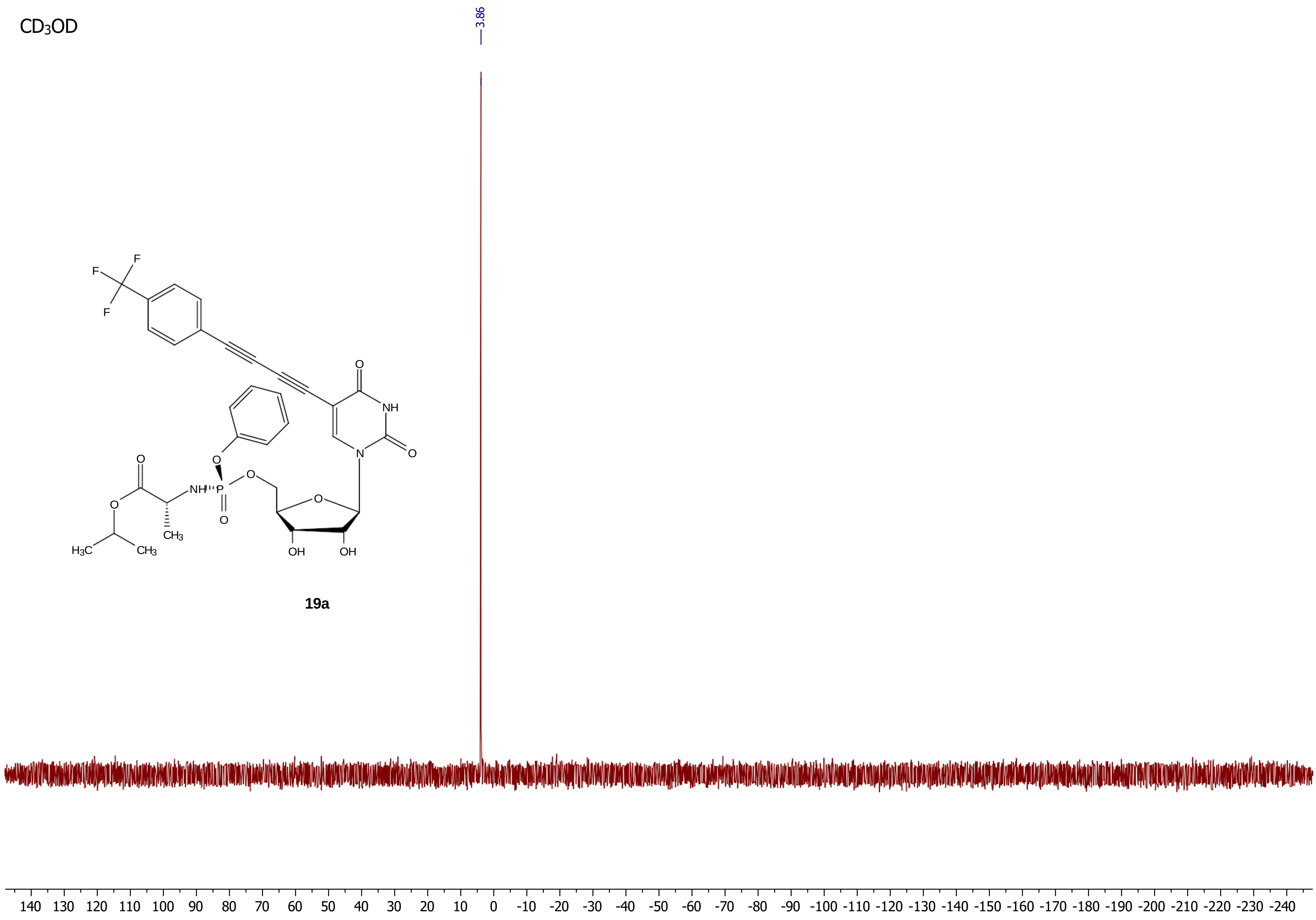
CD3OD
3.86
19a
140 130 120 110 100 90 80 70 60 50 40 30 20 10 0 -10 -20 -30 -40 -50 -60 -70 -80 -90 -100 -110 -120 -130 -140 -150 -160 -170 -180 -190 -200 -210 -220 -230 -240

f1 (ppm)

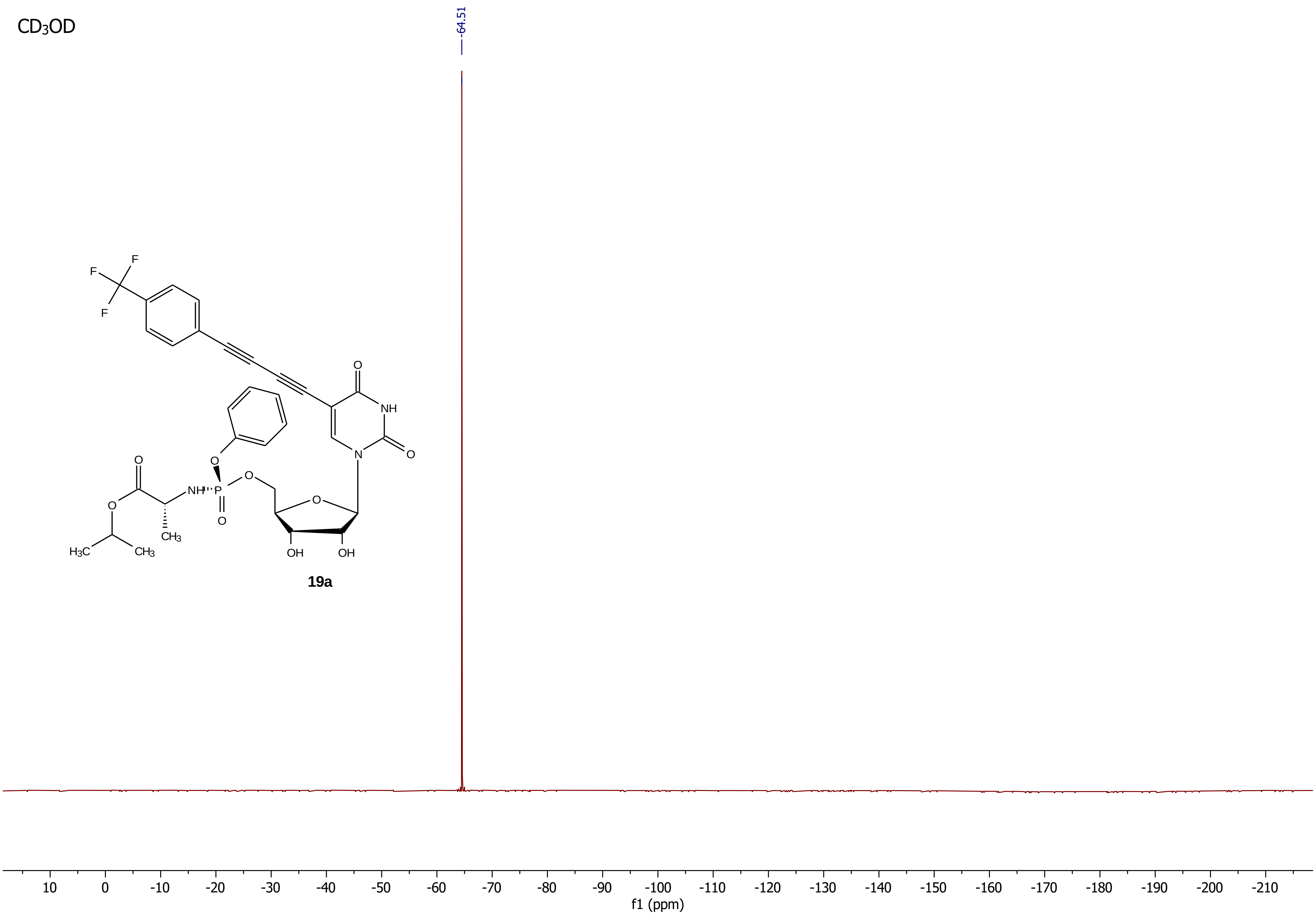
CD$_3$OD
-64.51
F
F
F
O
NH
O
N
O
O
NH
P
O
O
O
O
O
CH$_3$
H$_3$C
CH$_3$
OH
OH
19a
10 0 -10 -20 -30 -40 -50 -60 -70 -80 -90 -100 -110 -120 -130 -140 -150 -160 -170 -180 -190 -200 -210
f1 (ppm)

CD$_3$OD

19b

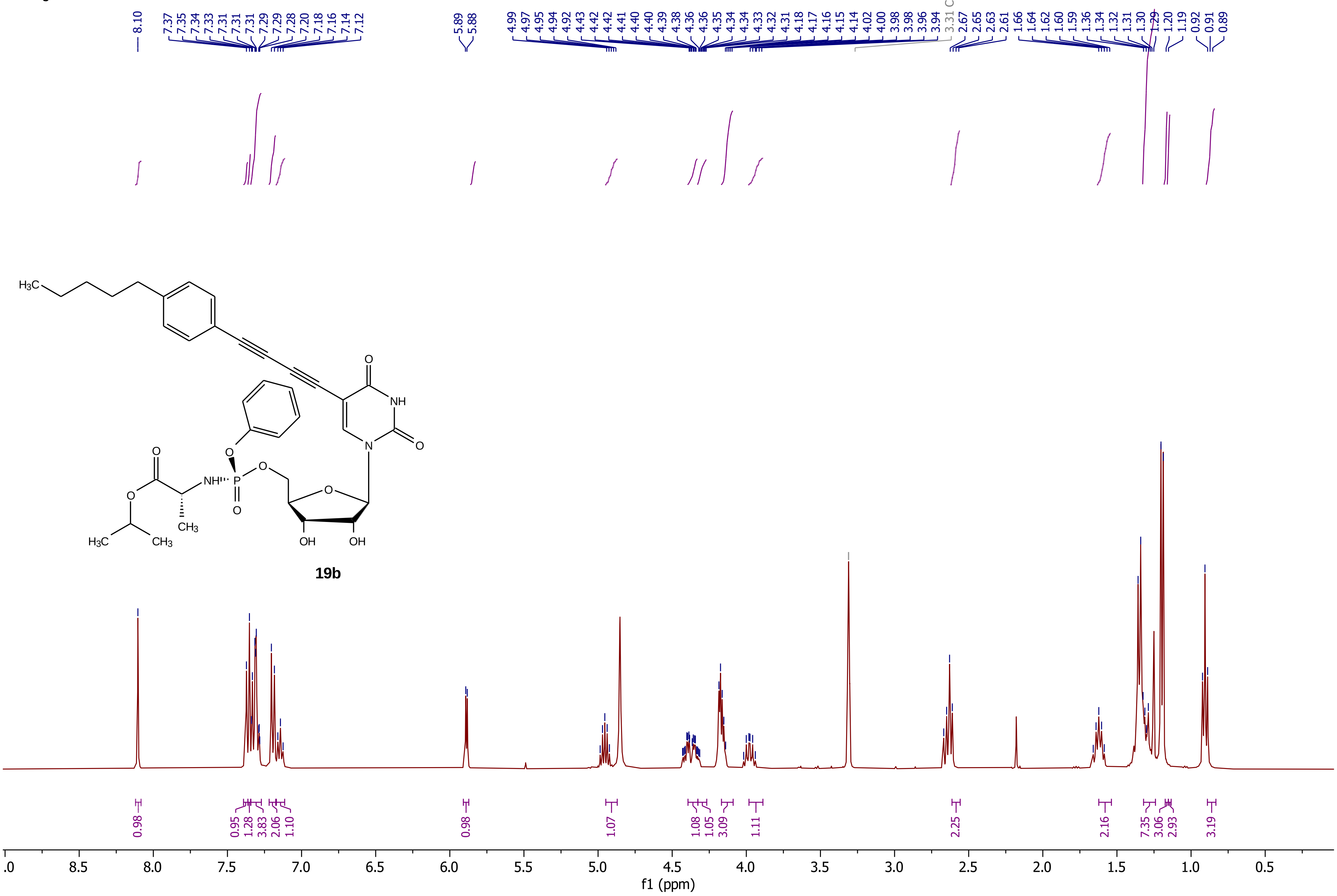
8.10
7.37 7.35 7.34 7.33 7.31 7.31 7.31 7.29 7.29 7.28 7.20 7.18 7.16 7.14 7.12
5.89 5.88
4.99 4.97 4.95 4.94 4.92 4.43 4.42 4.42 4.41 4.40 4.40 4.39 4.38 4.36 4.36 4.35 4.34 4.34 4.33 4.32 4.31 4.18 4.17 4.16 4.15 4.14 4.02 4.00 3.98 3.98 3.96 3.94
3.31 CD3OD
2.67 2.65 2.63 2.61
1.66 1.64 1.62 1.60 1.59 1.36 1.34 1.32 1.31 1.30 1.29 1.20 1.19 0.92 0.91 0.89
0.98
0.95 1.28 3.83 2.06 1.10
0.98
1.07
1.08 1.05 3.09
1.11
2.25
2.16
7.35 3.06 2.93
3.19
.0 8.5 8.0 7.5 7.0 6.5 6.0 5.5 5.0 4.5 4.0 3.5 3.0 2.5 2.0 1.5 1.0 0.5
f1 (ppm)

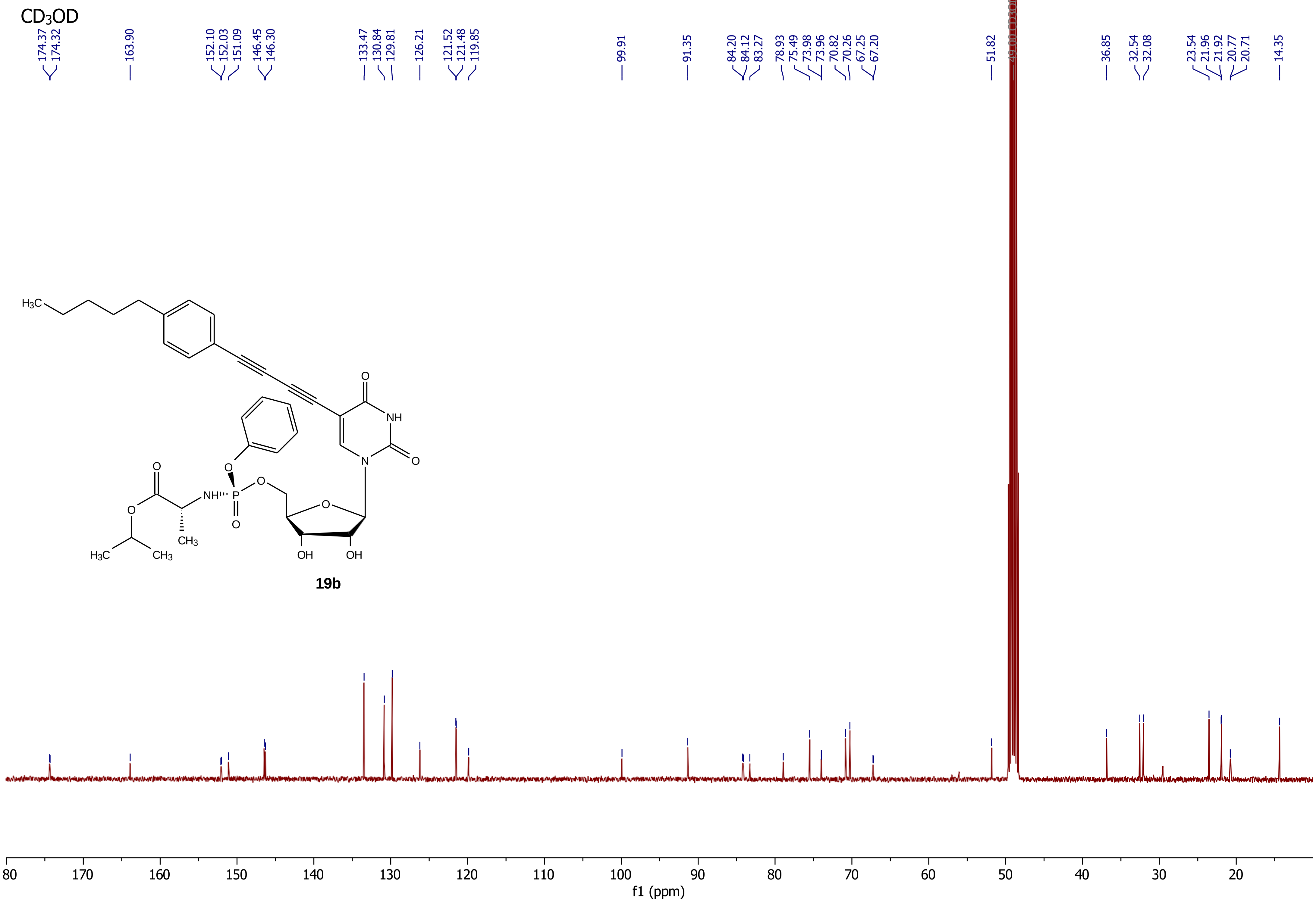
CD3OD
174.37
174.32
163.90
152.10
152.03
151.09
146.45
146.30
133.47
130.84
129.81
126.21
121.52
121.48
119.85
99.91
91.35
84.20
84.12
83.27
78.93
75.49
73.98
73.96
70.82
70.26
67.25
67.20
51.82
49.00 CD3OD
36.85
32.54
32.08
23.54
21.96
21.92
20.77
20.71
14.35
H3C
O
NH
N
O
P
OH
OH
CH3
80
170
160
150
140
130
120
110
100
90
80
70
60
50
40
30
20
f1 (ppm)

19b

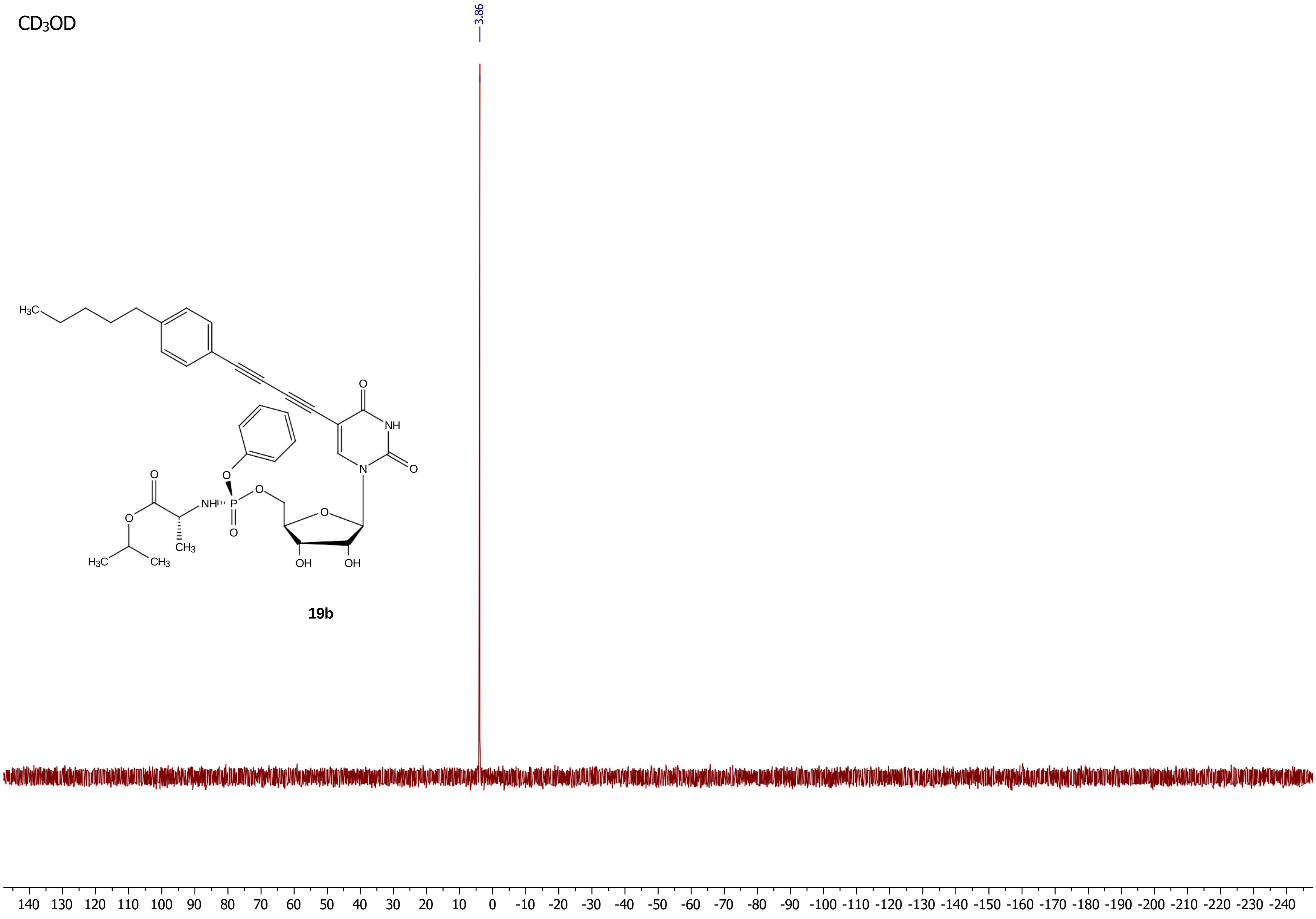
CD3OD
3.86
140 130 120 110 100 90 80 70 60 50 40 30 20 10 0 -10 -20 -30 -40 -50 -60 -70 -80 -90 -100 -110 -120 -130 -140 -150 -160 -170 -180 -190 -200 -210 -220 -230 -240

19b

f1 (ppm)

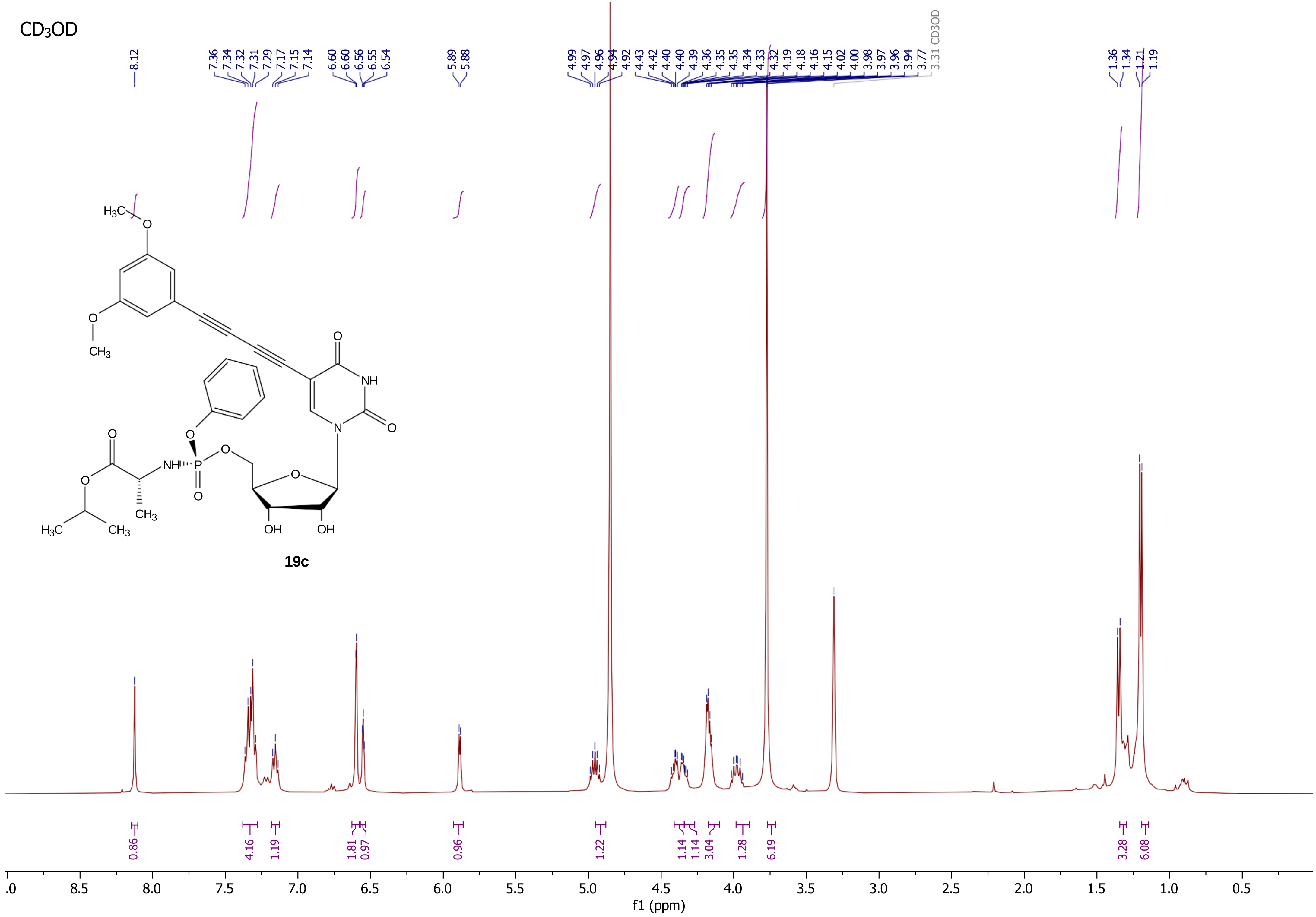
CD3OD
8.12
7.36 7.34 7.32 7.31 7.29 7.17 7.15 7.14
6.60 6.60 6.56 6.55 6.54
5.89 5.88
4.99 4.97 4.96 4.94 4.92 4.43 4.42 4.40 4.40 4.39 4.36 4.35 4.35 4.34 4.33 4.32 4.19 4.18 4.16 4.15 4.02 4.00 3.98 3.97 3.96 3.94 3.77
3.31 CD3OD
1.36 1.34 1.21 1.19
0.86 4.16 1.19 1.81 0.97 0.96 1.22 1.14 1.14 3.04 1.28 6.19 3.28 6.08
.0 8.5 8.0 7.5 7.0 6.5 6.0 5.5 5.0 4.5 4.0 3.5 3.0 2.5 2.0 1.5 1.0 0.5
f1 (ppm)
H3C O CH3 O NH O N O O OH OH NH P O O H3C CH3 CH3

19c

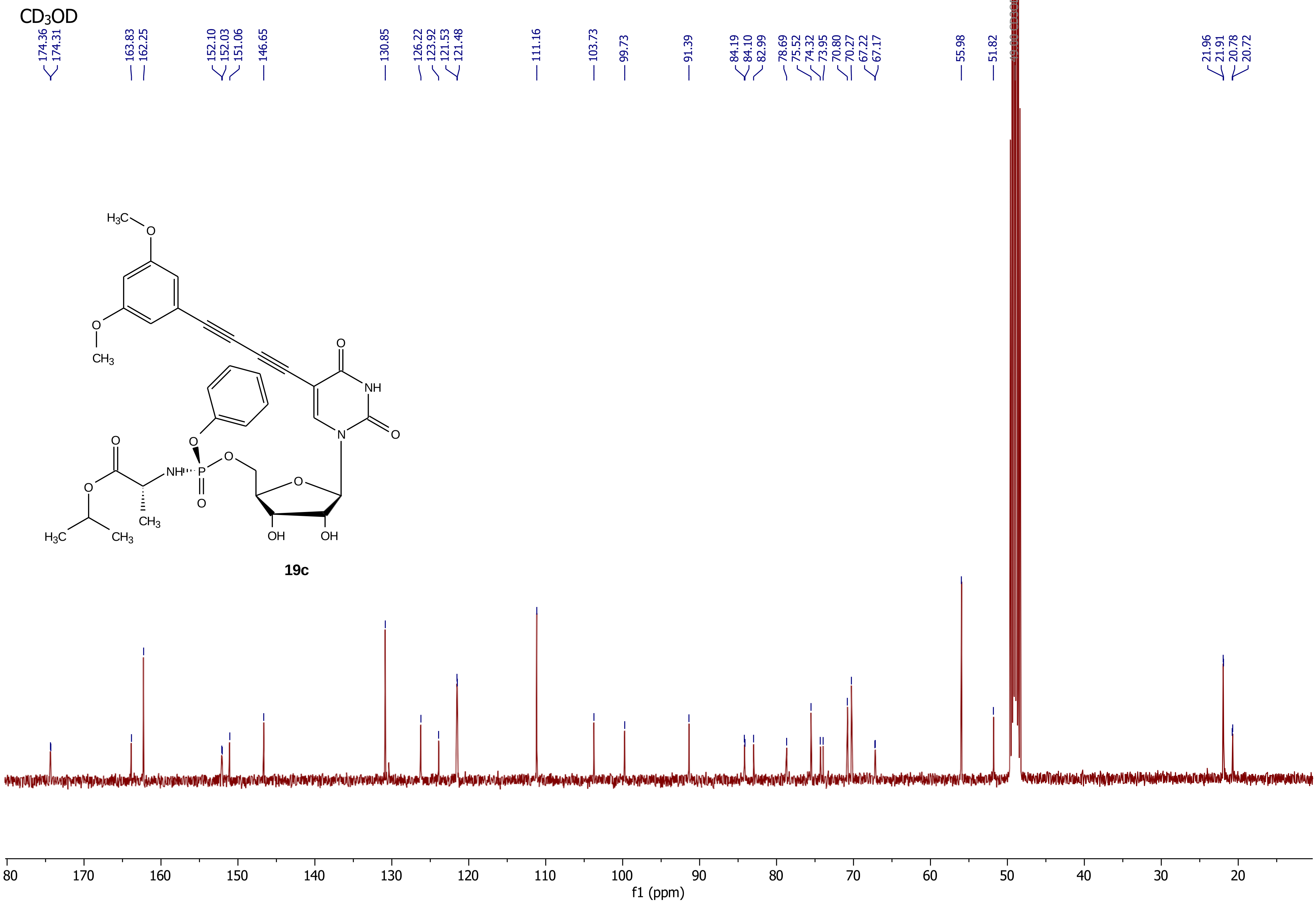
CD3OD
174.36
174.31
163.83
162.25
152.10
152.03
151.06
146.65
130.85
126.22
123.92
121.53
121.48
111.16
103.73
99.73
91.39
84.19
84.10
82.99
78.69
75.52
74.32
73.95
70.80
70.27
67.22
67.17
55.98
51.82
49.00 CD3OD
21.96
21.91
20.78
20.72
H3C
O
CH3
NH
N
OH
P
80 170 160 150 140 130 120 110 100 90 80 70 60 50 40 30 20
f1 (ppm)

19c

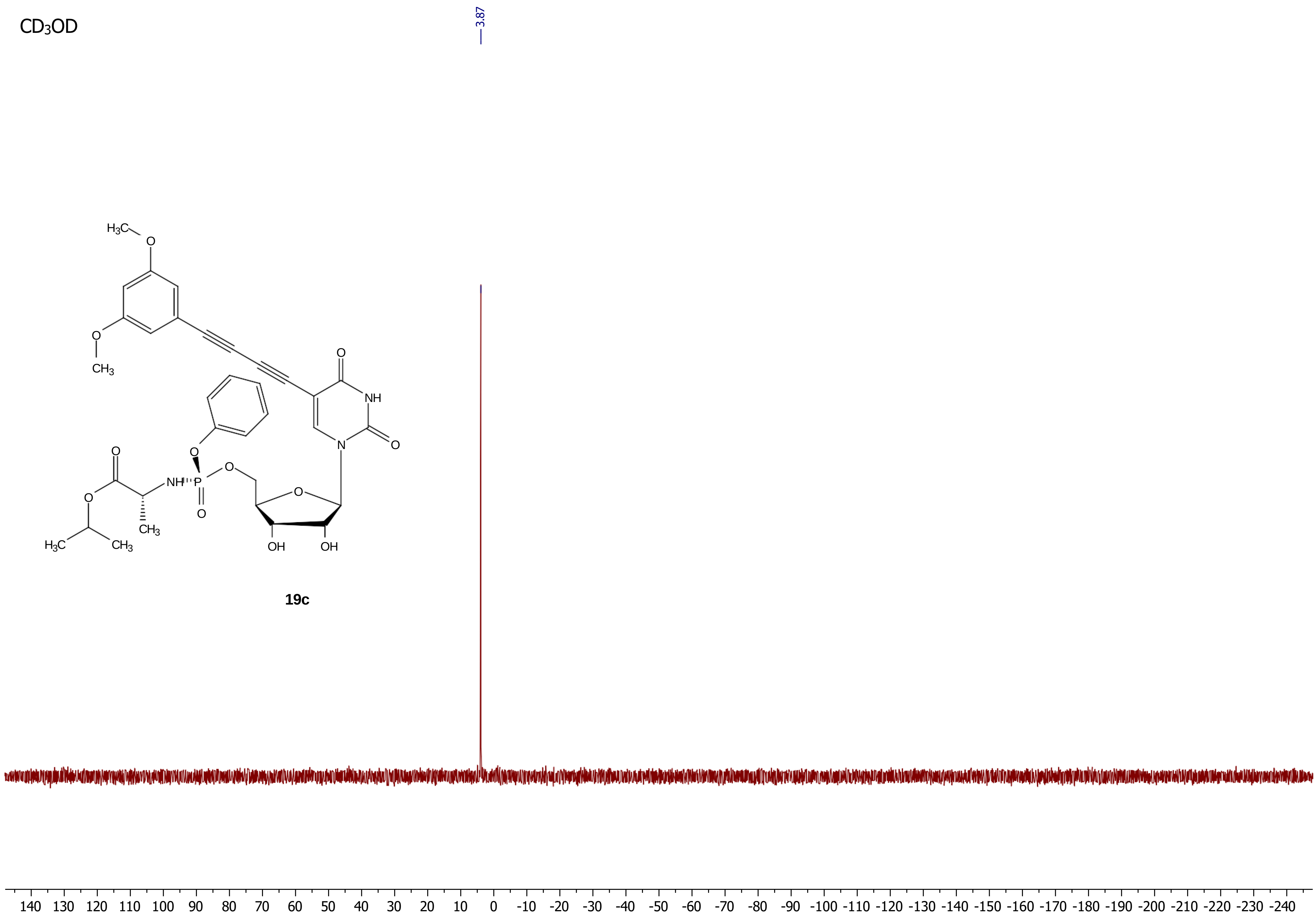
CD3OD
3.87
H3C
O
O
CH3
O
NH
O
N
O
O
O
NH
P
O
O
CH3
H3C
CH3
O
OH
OH
140 130 120 110 100 90 80 70 60 50 40 30 20 10 0 -10 -20 -30 -40 -50 -60 -70 -80 -90 -100 -110 -120 -130 -140 -150 -160 -170 -180 -190 -200 -210 -220 -230 -240

19c

f1 (ppm)

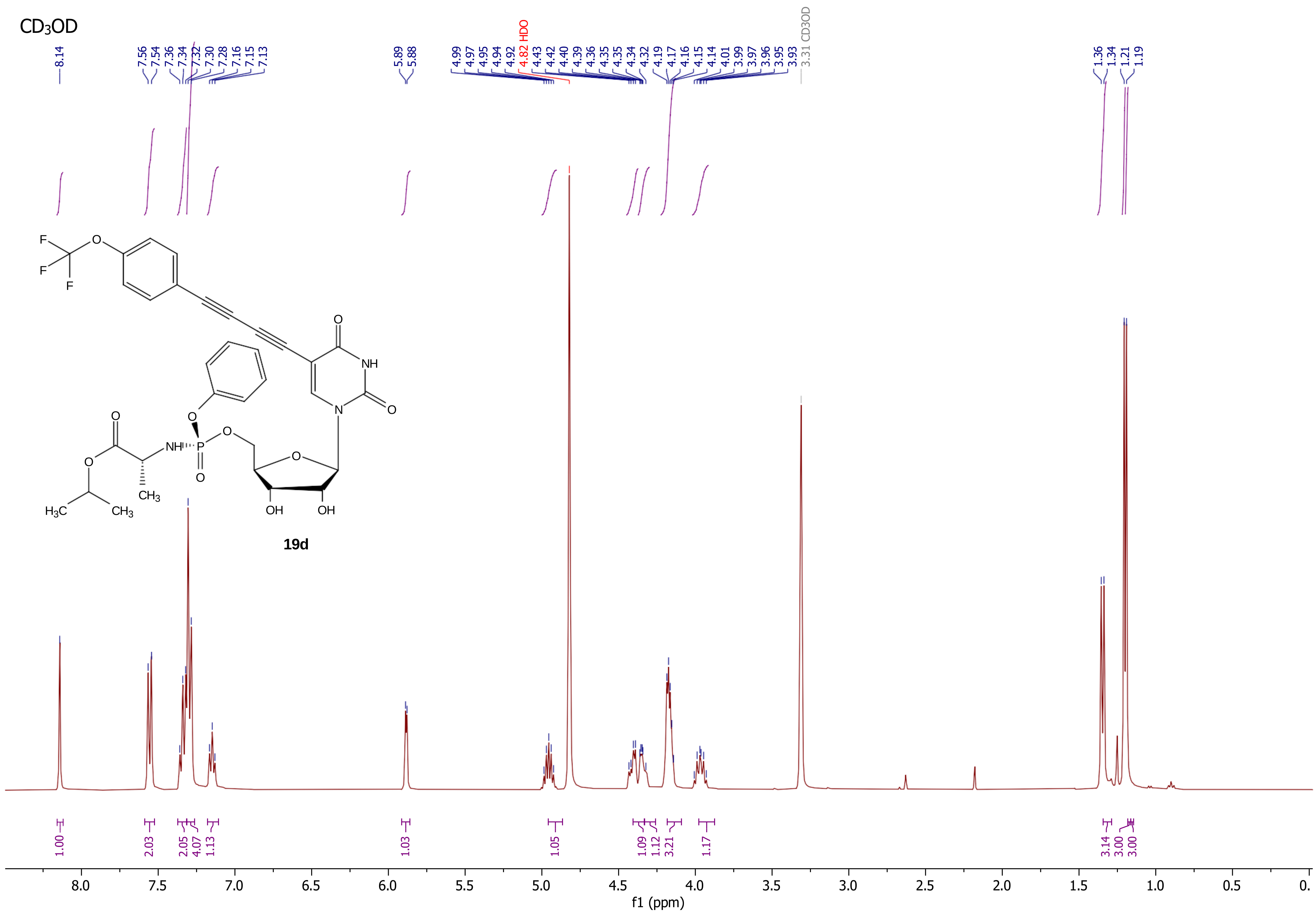
CD3OD
8.14
7.56
7.54
7.36
7.34
7.32
7.30
7.28
7.16
7.15
7.13
5.89
5.88
4.99
4.97
4.95
4.94
4.92
4.82 HDO
4.43
4.42
4.40
4.39
4.36
4.35
4.35
4.34
4.32
4.19
4.17
4.16
4.15
4.14
4.01
3.99
3.97
3.96
3.95
3.93
3.31 CD3OD
1.36
1.34
1.21
1.19
F
F
F
O
NH
O
O
N
O
O
O
NH
P
O
O
O
CH3
H3C
CH3
O
OH
OH
19d
1.00
2.03
2.05
4.07
1.13
1.03
1.05
1.09
1.12
3.21
1.17
3.14
3.00
3.00
8.0
7.5
7.0
6.5
6.0
5.5
5.0
4.5
4.0
3.5
3.0
2.5
2.0
1.5
1.0
0.5
0.
f1 (ppm)

CD$_3$OD

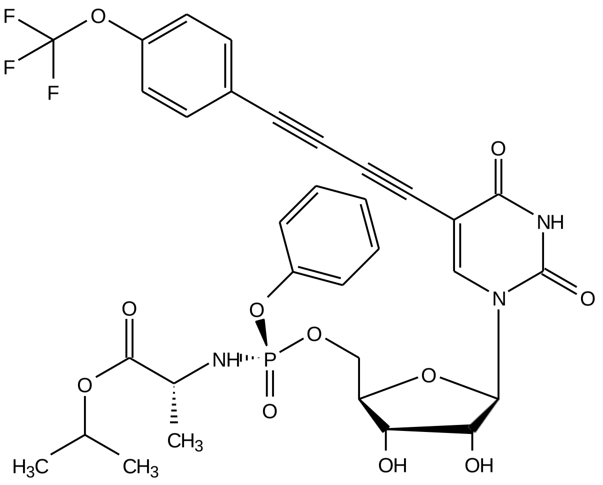
F
F
F
O
O
NH
O
N
O
NH
P
O
O
O
O
CH3
H3C
CH3
OH
OH

19d

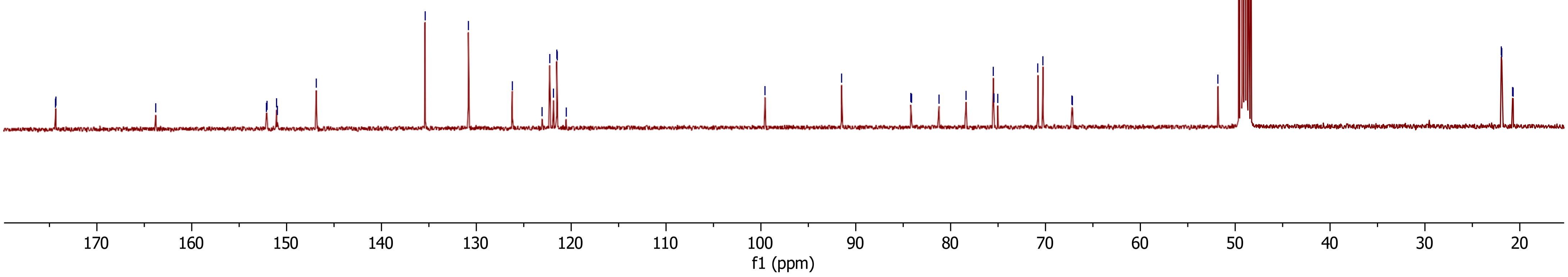
174.37
174.32
163.79
152.13
152.06
151.06
150.97
146.87
135.39
130.83
126.20
123.07
122.26
121.87
121.51
121.47
120.52
99.57
91.49
84.20
84.11
81.22
78.37
75.50
75.43
75.03
70.82
70.27
67.21
67.16
51.83
49.00 CD3OD
21.95
21.90
20.76
20.70
170
160
150
140
130
120
110
100
90
80
70
60
50
40
30
20
f1 (ppm)

CD$_3$OD

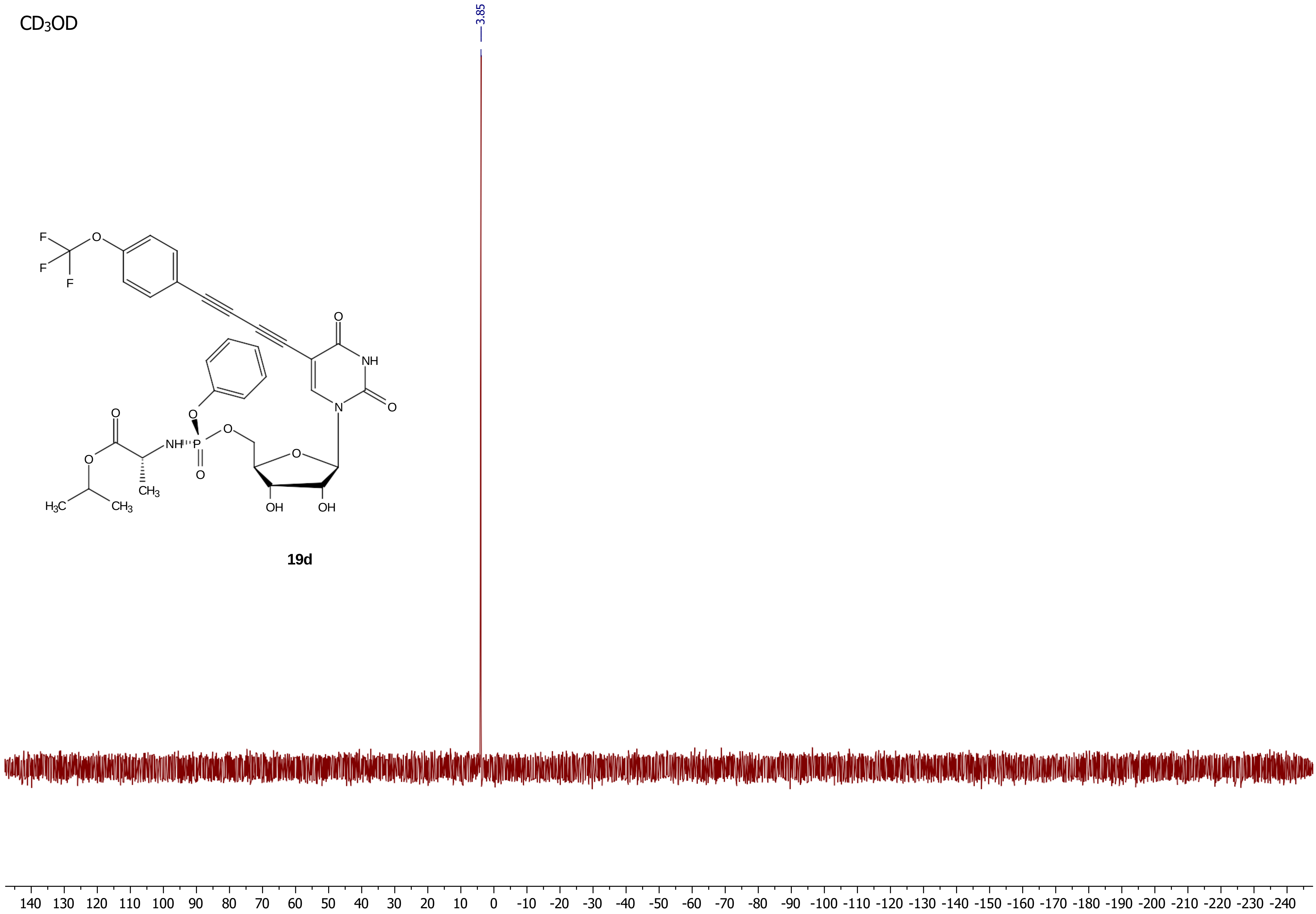
3.85
140 130 120 110 100 90 80 70 60 50 40 30 20 10 0 -10 -20 -30 -40 -50 -60 -70 -80 -90 -100 -110 -120 -130 -140 -150 -160 -170 -180 -190 -200 -210 -220 -230 -240

19d

f1 (ppm)

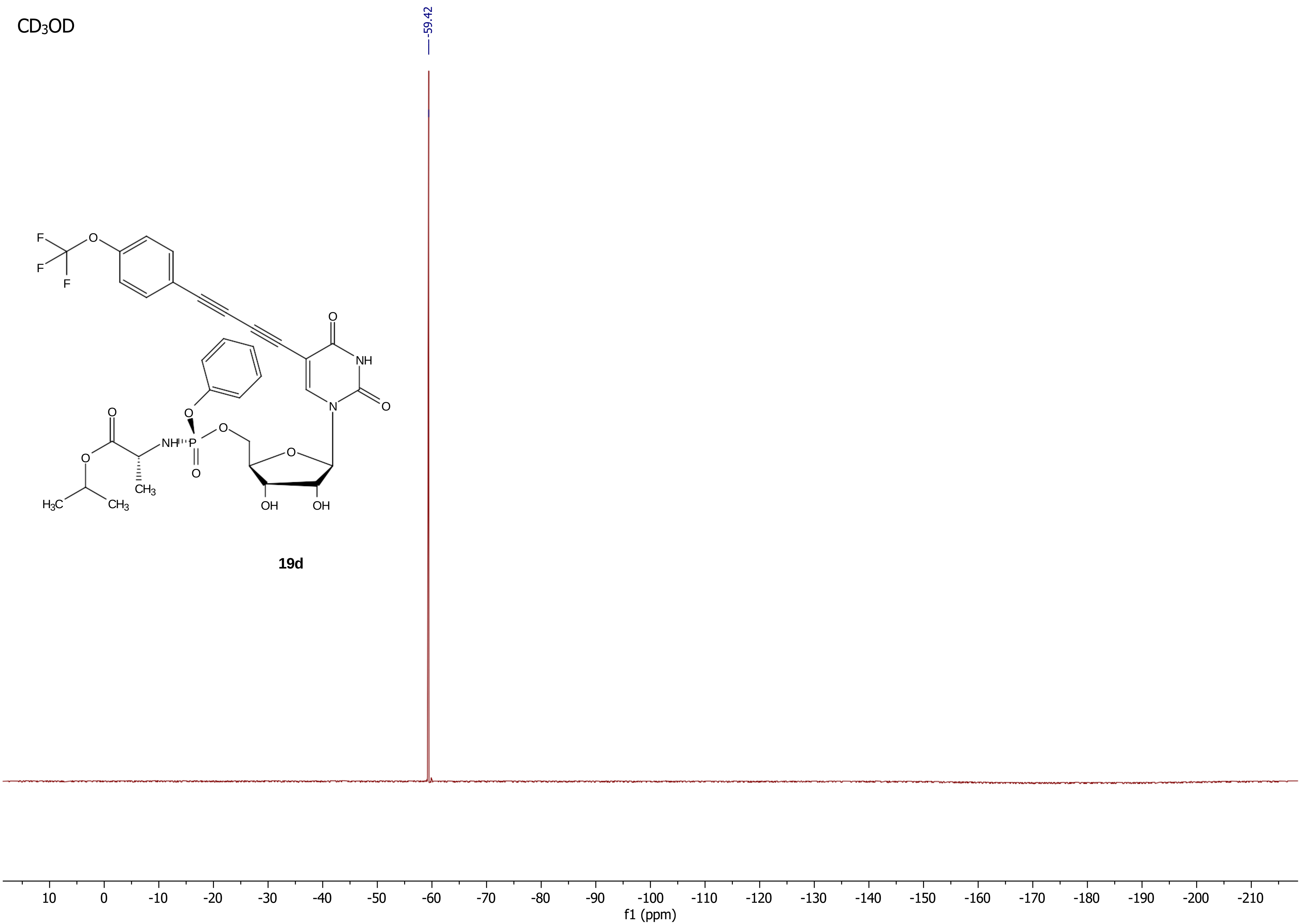
CD3OD
-59.42
F
F
F
O
O
NH
O
N
O
O
NH
P
O
O
O
O
O
CH3
H3C
CH3
OH
OH
19d
10 0 -10 -20 -30 -40 -50 -60 -70 -80 -90 -100 -110 -120 -130 -140 -150 -160 -170 -180 -190 -200 -210
f1 (ppm)

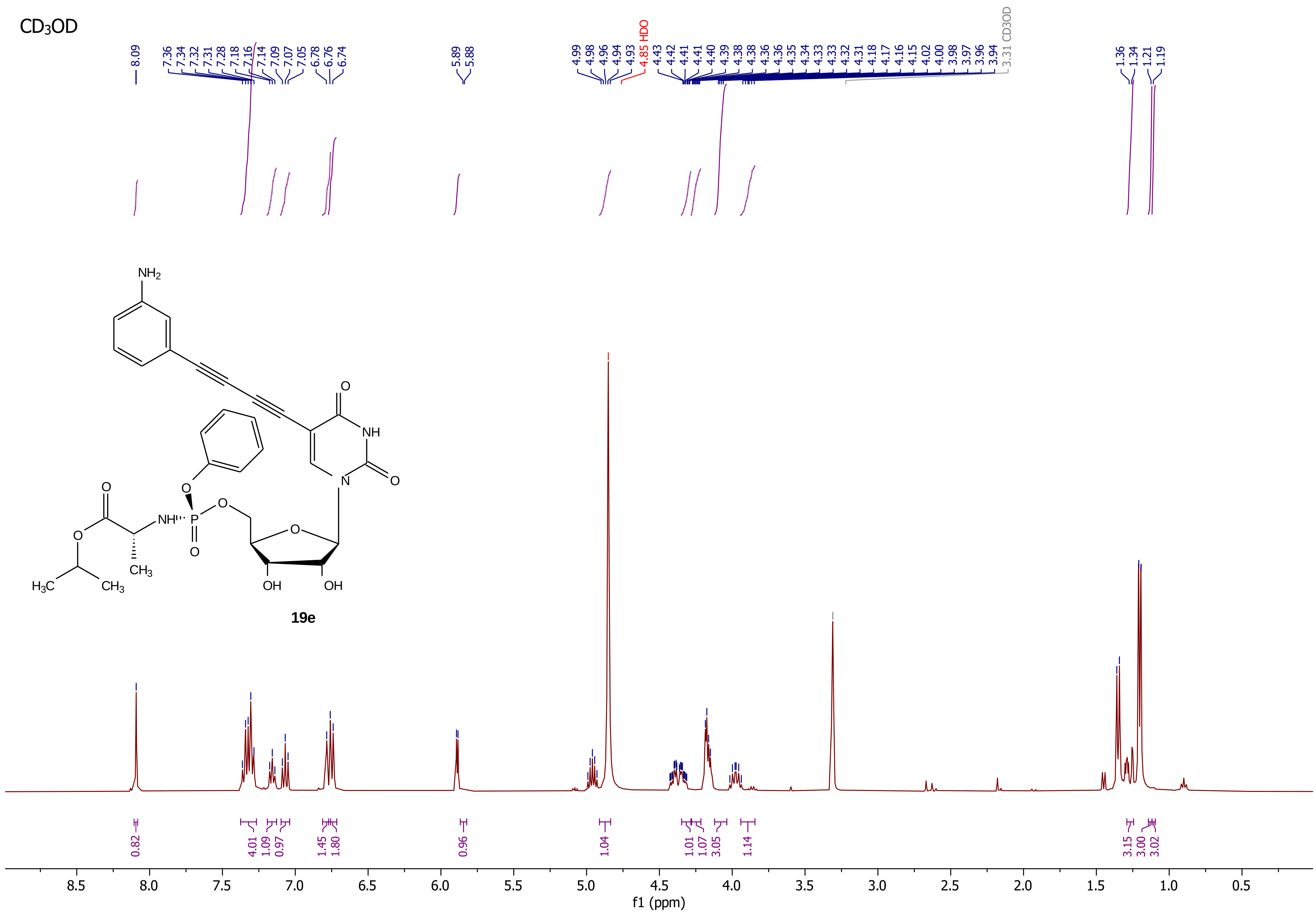
CD3OD
8.09
7.36 7.34 7.32 7.31 7.28 7.18 7.16 7.14 7.09 7.07 7.05 6.78 6.76 6.74
5.89 5.88
4.99 4.98 4.96 4.94 4.93
4.85 HDO
4.43 4.42 4.41 4.41 4.40 4.39 4.38 4.38 4.36 4.36 4.35 4.34 4.33 4.33 4.32 4.31 4.18 4.17 4.16 4.15 4.02 4.00 3.98 3.97 3.96 3.94
3.31 CD3OD
1.36 1.34 1.21 1.19
NH2
NH
N
O
OH
OH
P
H3C
CH3
CH3
19e
0.82 4.01 1.09 0.97 1.45 1.80 0.96 1.04 1.01 1.07 3.05 1.14 3.15 3.00 3.02
8.5 8.0 7.5 7.0 6.5 6.0 5.5 5.0 4.5 4.0 3.5 3.0 2.5 2.0 1.5 1.0 0.5
f1 (ppm)

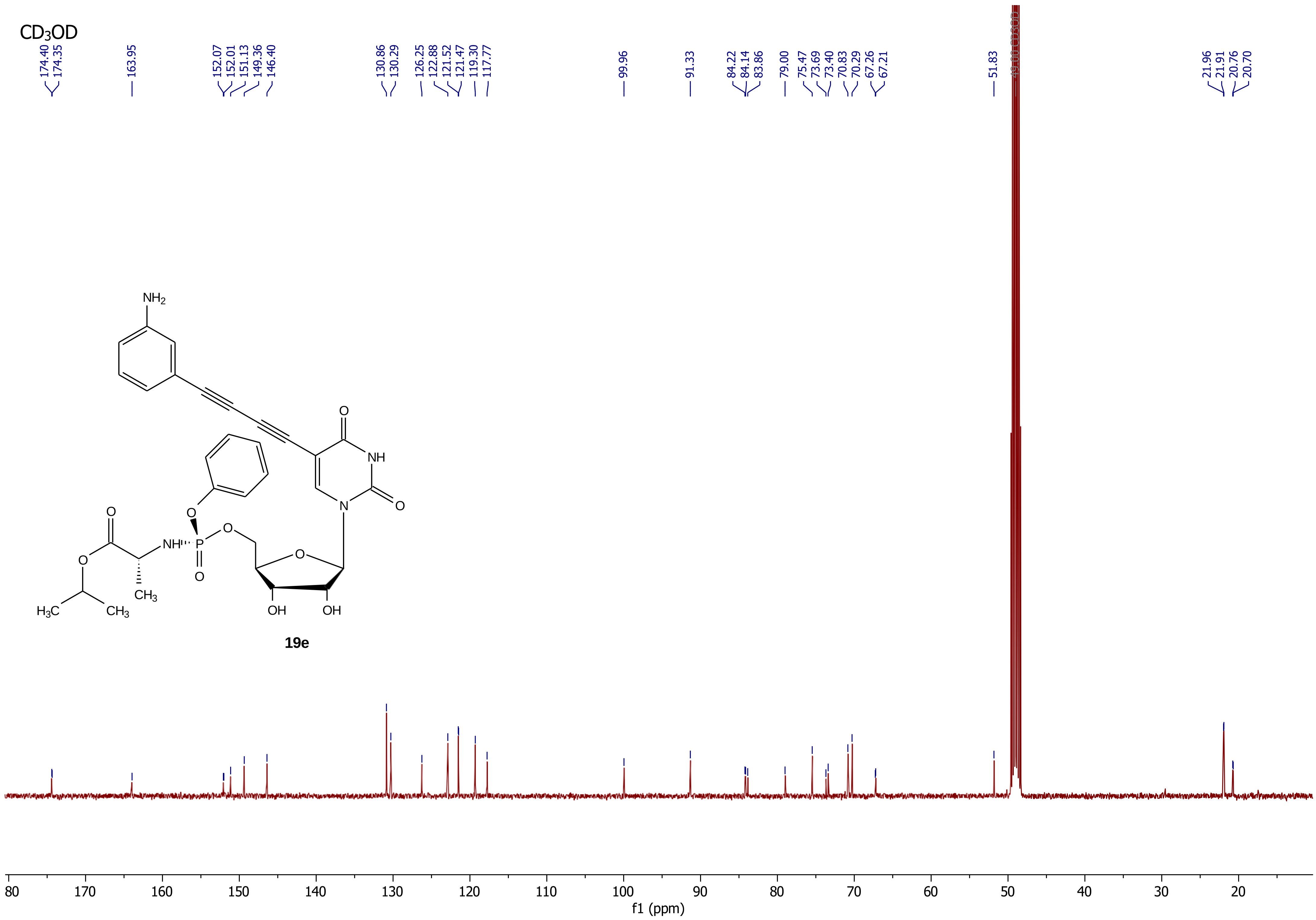
CD3OD
174.40
174.35
163.95
152.07
152.01
151.13
149.36
146.40
130.86
130.29
126.25
122.88
121.52
121.47
119.30
117.77
99.96
91.33
84.22
84.14
83.86
79.00
75.47
73.69
73.40
70.83
70.29
67.26
67.21
51.83
21.96
21.91
20.76
20.70
NH2
O
NH
N
O
O
O
NH
P
O
O
O
O
H3C
CH3
CH3
OH
OH
19e
80
170
160
150
140
130
120
110
100
90
80
70
60
50
40
30
20
f1 (ppm)

CD$_3$OD

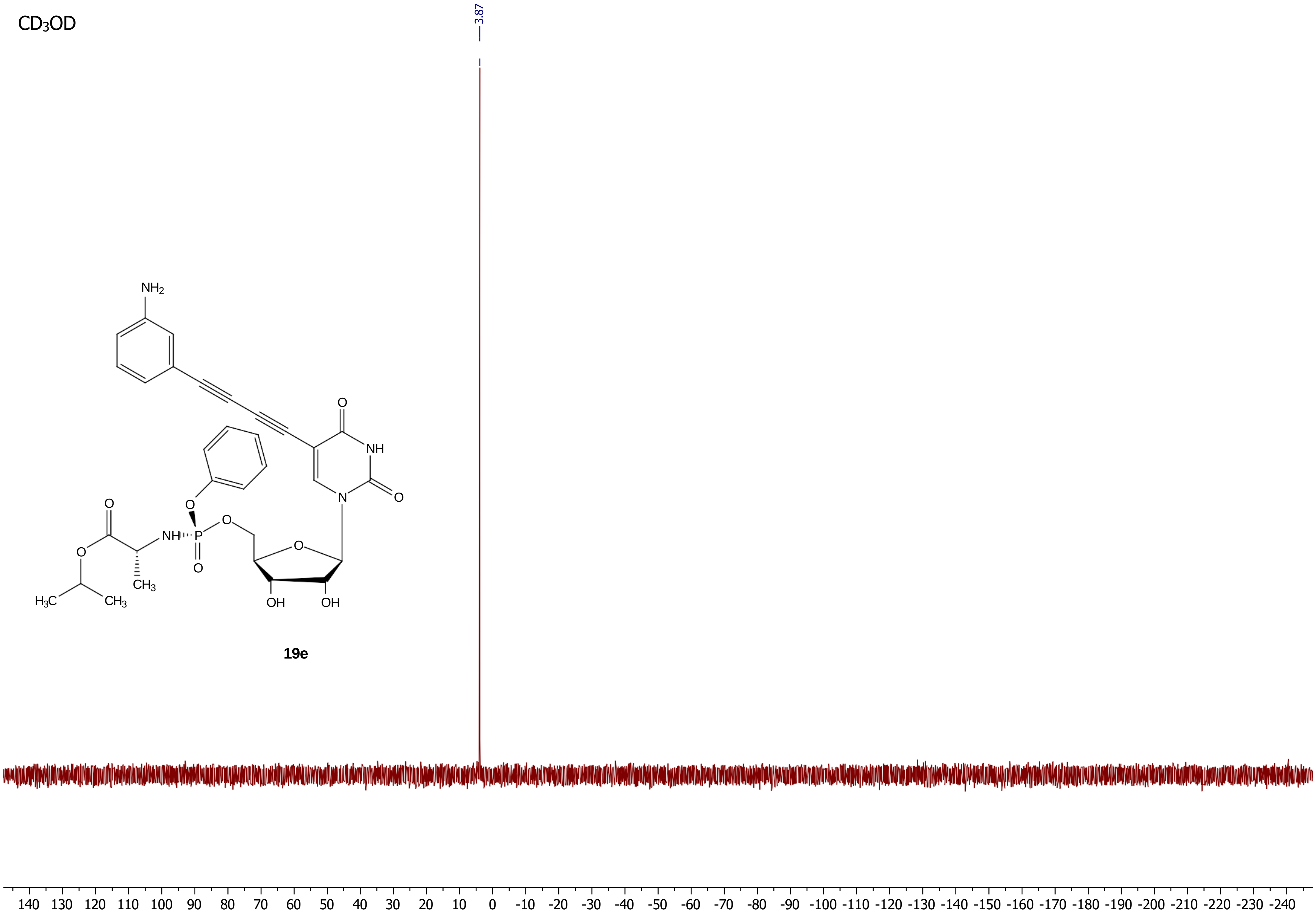
3.87
NH2
O
NH
O
N
O
O
NH
P
O
O
O
O
O
CH3
H3C
CH3
OH
OH
140 130 120 110 100 90 80 70 60 50 40 30 20 10 0 -10 -20 -30 -40 -50 -60 -70 -80 -90 -100 -110 -120 -130 -140 -150 -160 -170 -180 -190 -200 -210 -220 -230 -240

19e

f1 (ppm)

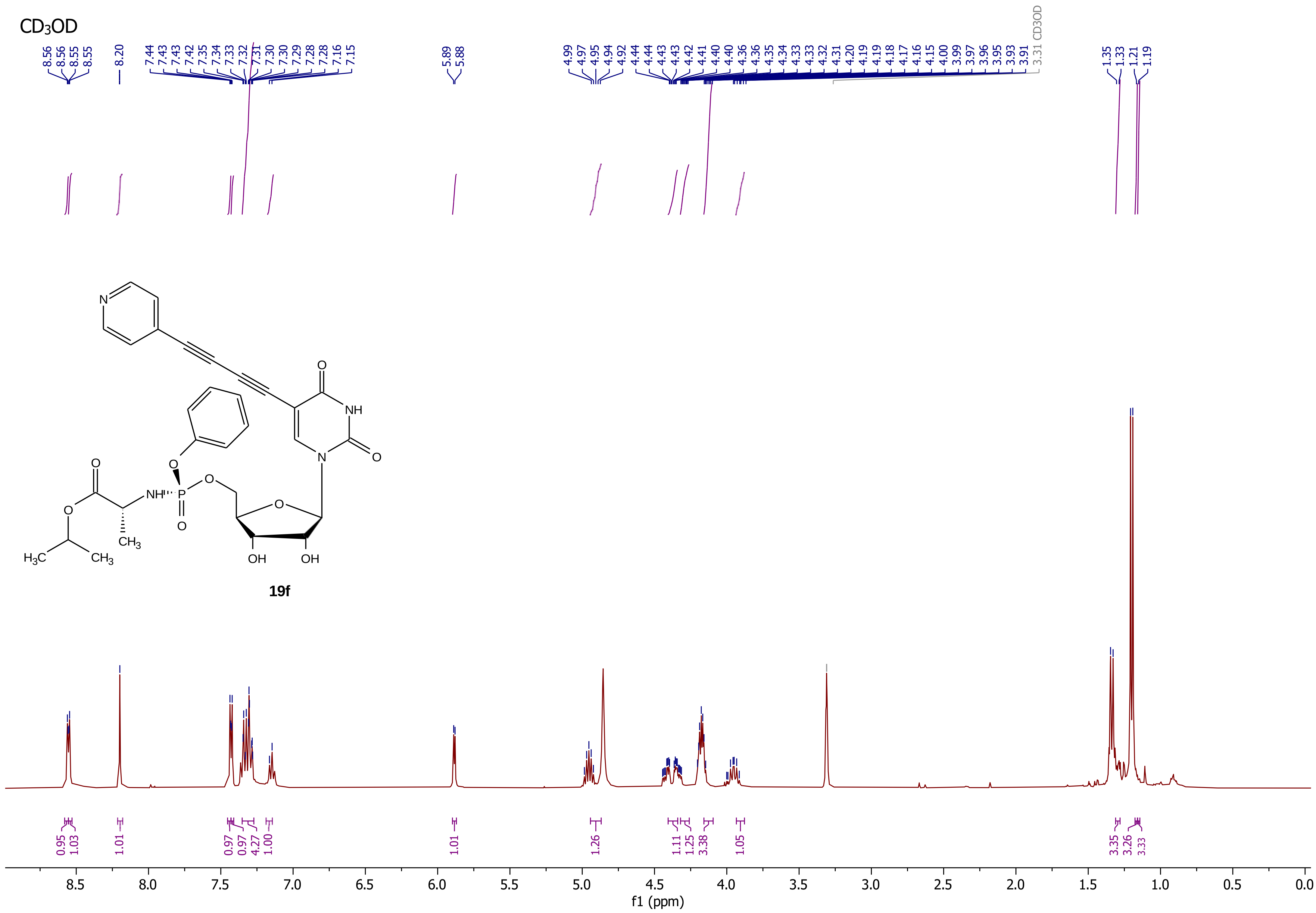
CD3OD
8.56 8.56 8.55 8.55 8.20 7.44 7.43 7.43 7.42 7.35 7.34 7.33 7.32 7.31 7.30 7.30 7.29 7.28 7.28 7.16 7.15 5.89 5.88 4.99 4.97 4.95 4.94 4.92 4.44 4.44 4.43 4.43 4.42 4.41 4.40 4.40 4.36 4.36 4.35 4.34 4.33 4.33 4.32 4.31 4.20 4.19 4.19 4.18 4.17 4.16 4.15 4.00 3.99 3.97 3.96 3.95 3.93 3.91 3.31 CD3OD 1.35 1.33 1.21 1.19
0.95 1.03 1.01 0.97 0.97 4.27 1.00 1.01 1.26 1.11 1.25 3.38 1.05 3.35 3.26 3.33
8.5 8.0 7.5 7.0 6.5 6.0 5.5 5.0 4.5 4.0 3.5 3.0 2.5 2.0 1.5 1.0 0.5 0.0
f1 (ppm)
19f

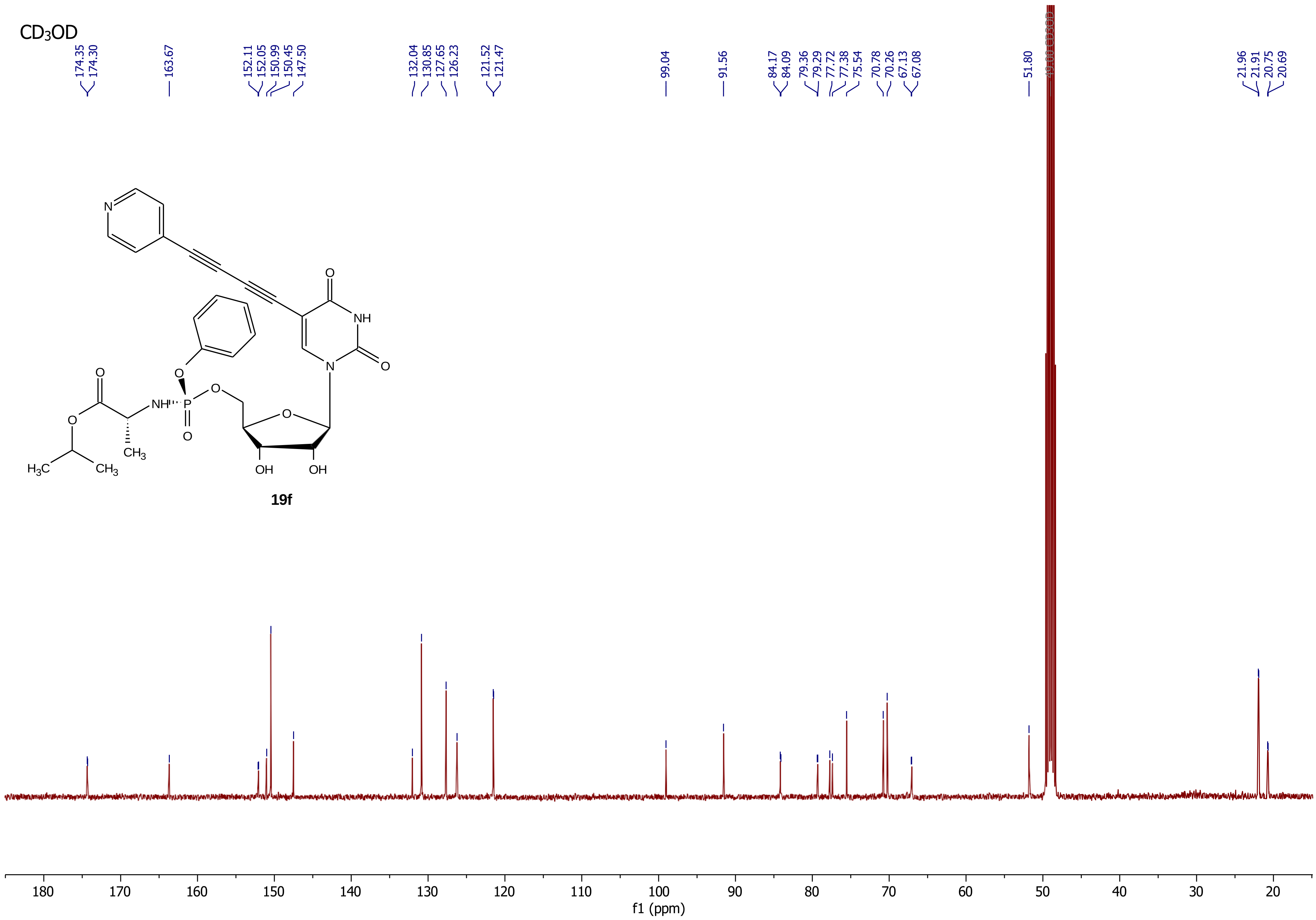
CD3OD
174.35
174.30
163.67
152.11
152.05
150.99
150.45
147.50
132.04
130.85
127.65
126.23
121.52
121.47
99.04
91.56
84.17
84.09
79.36
79.29
77.72
77.38
75.54
70.78
70.26
67.13
67.08
51.80
49.00 CD3OD
21.96
21.91
20.75
20.69
19f
180
170
160
150
140
130
120
110
100
90
80
70
60
50
40
30
20
f1 (ppm)

CD3OD

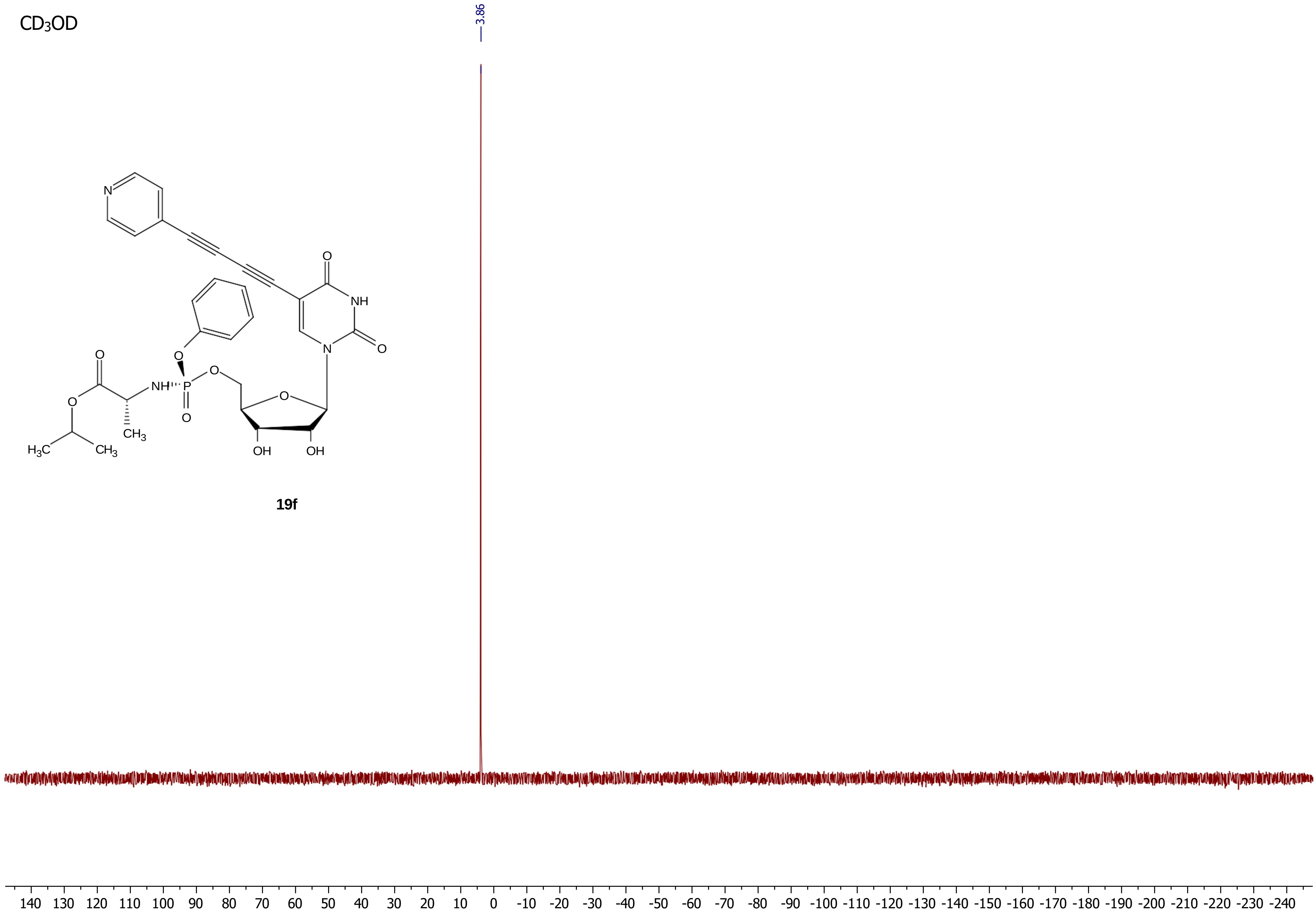
3.86
19f
140 130 120 110 100 90 80 70 60 50 40 30 20 10 0 -10 -20 -30 -40 -50 -60 -70 -80 -90 -100 -110 -120 -130 -140 -150 -160 -170 -180 -190 -200 -210 -220 -230 -240

f1 (ppm)

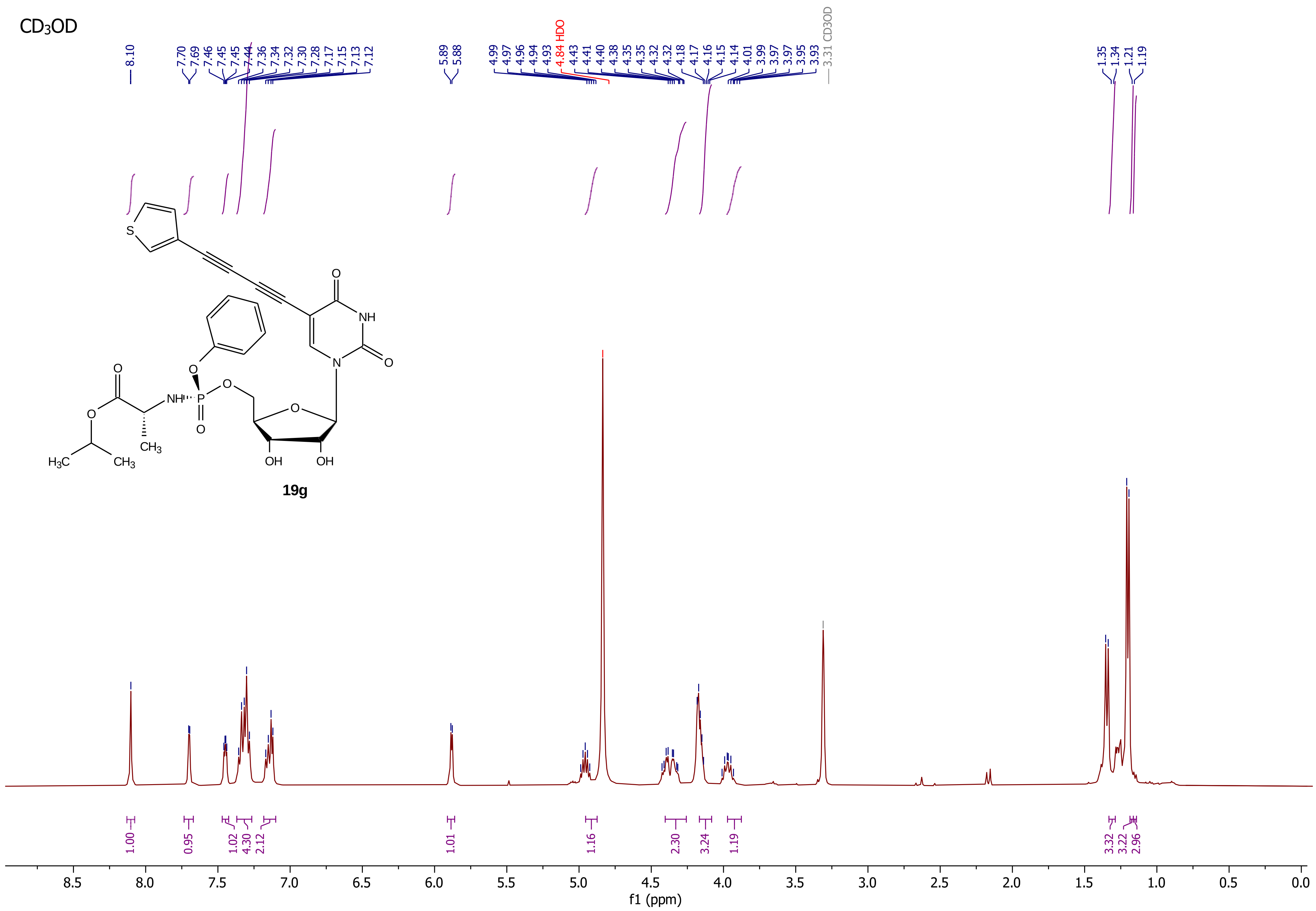
CD3OD
8.10
7.70
7.69
7.46
7.45
7.45
7.44
7.36
7.34
7.32
7.30
7.28
7.17
7.15
7.13
7.12
5.89
5.88
4.99
4.97
4.96
4.94
4.93
4.84 HDO
4.43
4.41
4.40
4.38
4.35
4.35
4.32
4.32
4.18
4.17
4.16
4.15
4.14
4.01
3.99
3.97
3.97
3.95
3.93
3.31 CD3OD
1.35
1.34
1.21
1.19
1.00
0.95
1.02
4.30
2.12
1.01
1.16
2.30
3.24
1.19
3.32
3.22
2.96
19g
f1 (ppm)

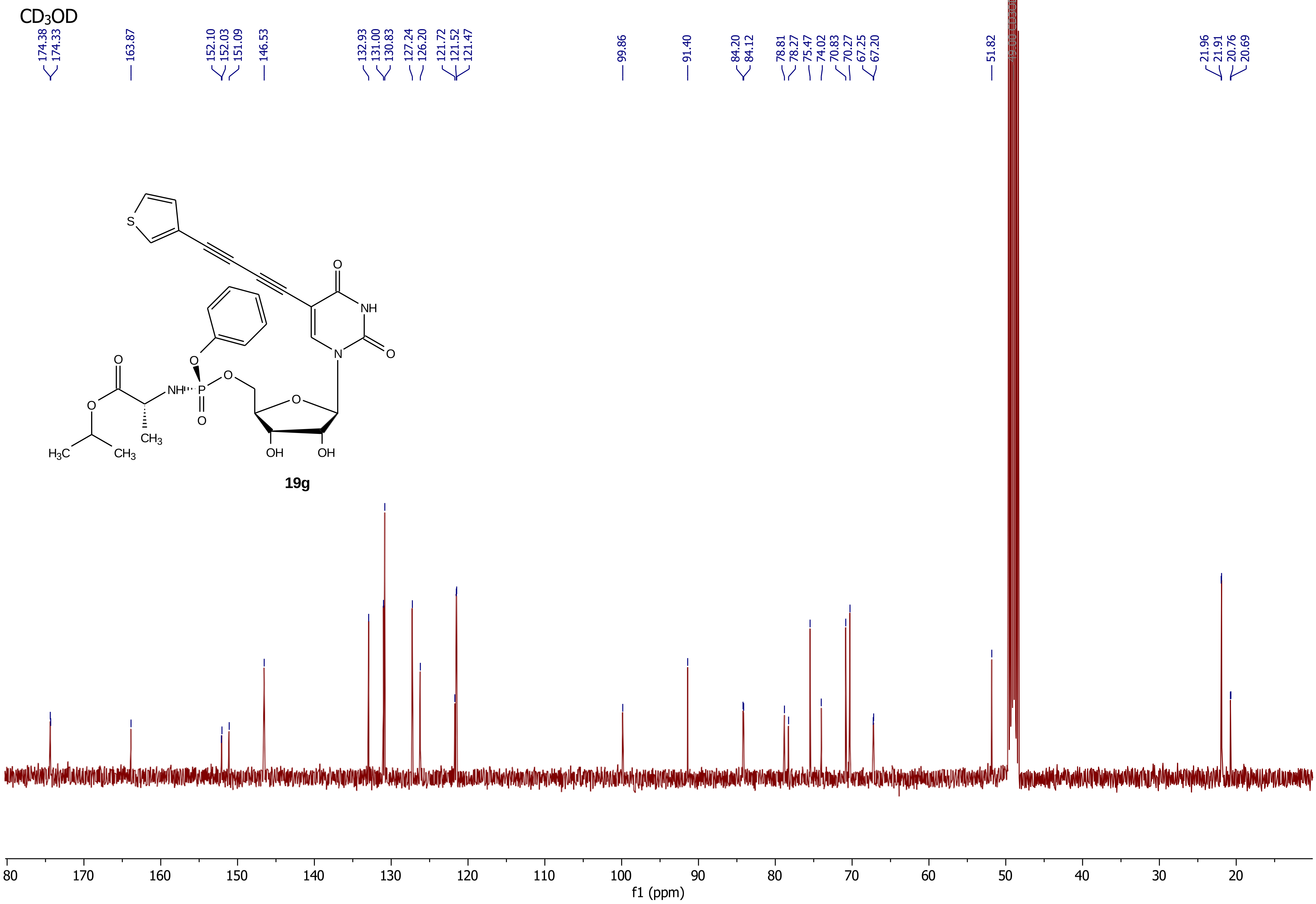
CD3OD
174.38
174.33
163.87
152.10
152.03
151.09
146.53
132.93
131.00
130.83
127.24
126.20
121.72
121.52
121.47
99.86
91.40
84.20
84.12
78.81
78.27
75.47
74.02
70.83
70.27
67.25
67.20
51.82
21.96
21.91
20.76
20.69
19g
80
170
160
150
140
130
120
110
100
90
80
70
60
50
40
30
20
f1 (ppm)

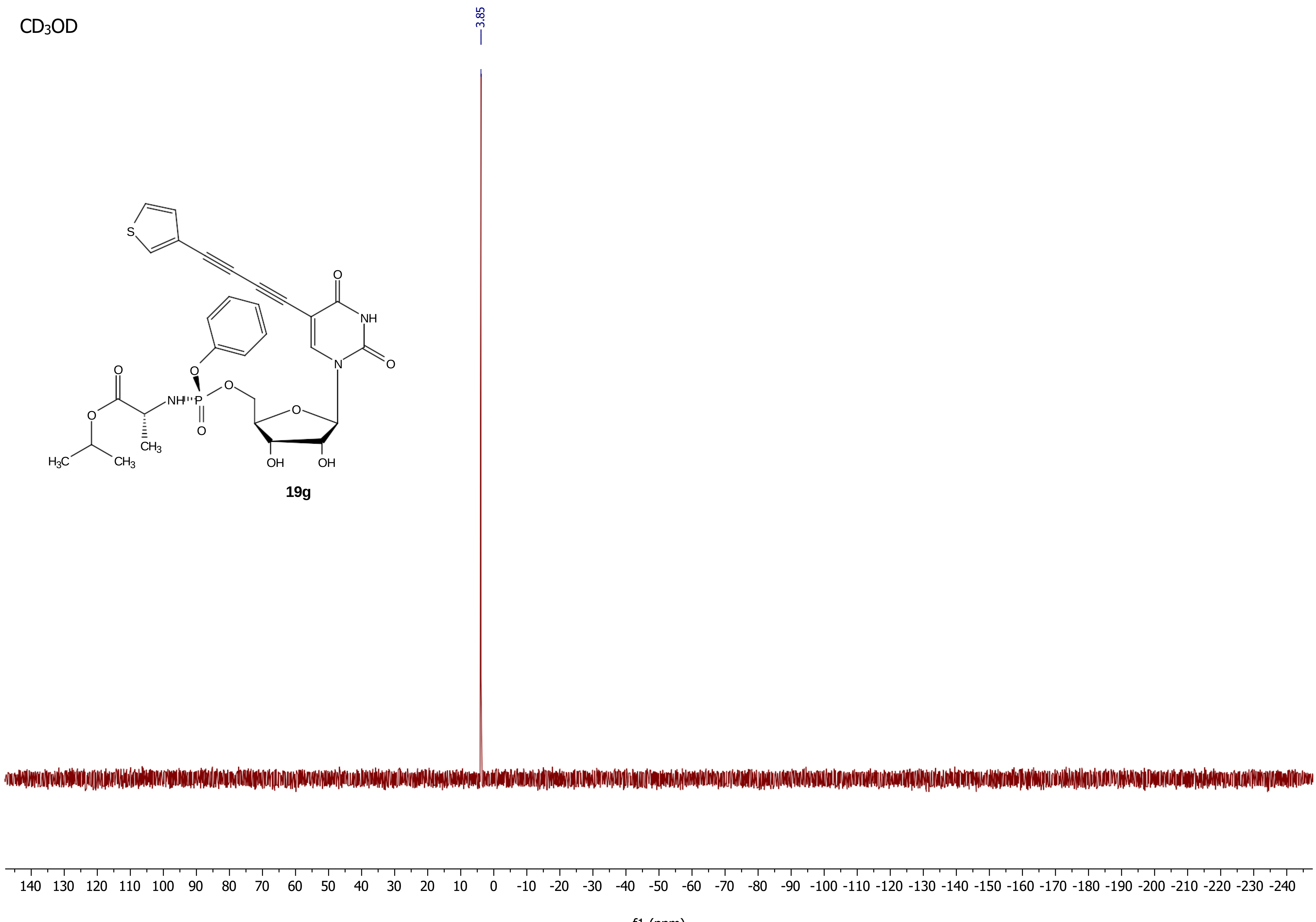
CD3OD
3.85
S
O
NH
N
O
O
NH
P
O
O
O
O
O
CH3
H3C
CH3
OH
OH
19g
140 130 120 110 100 90 80 70 60 50 40 30 20 10 0 -10 -20 -30 -40 -50 -60 -70 -80 -90 -100 -110 -120 -130 -140 -150 -160 -170 -180 -190 -200 -210 -220 -230 -240
f1 (ppm)

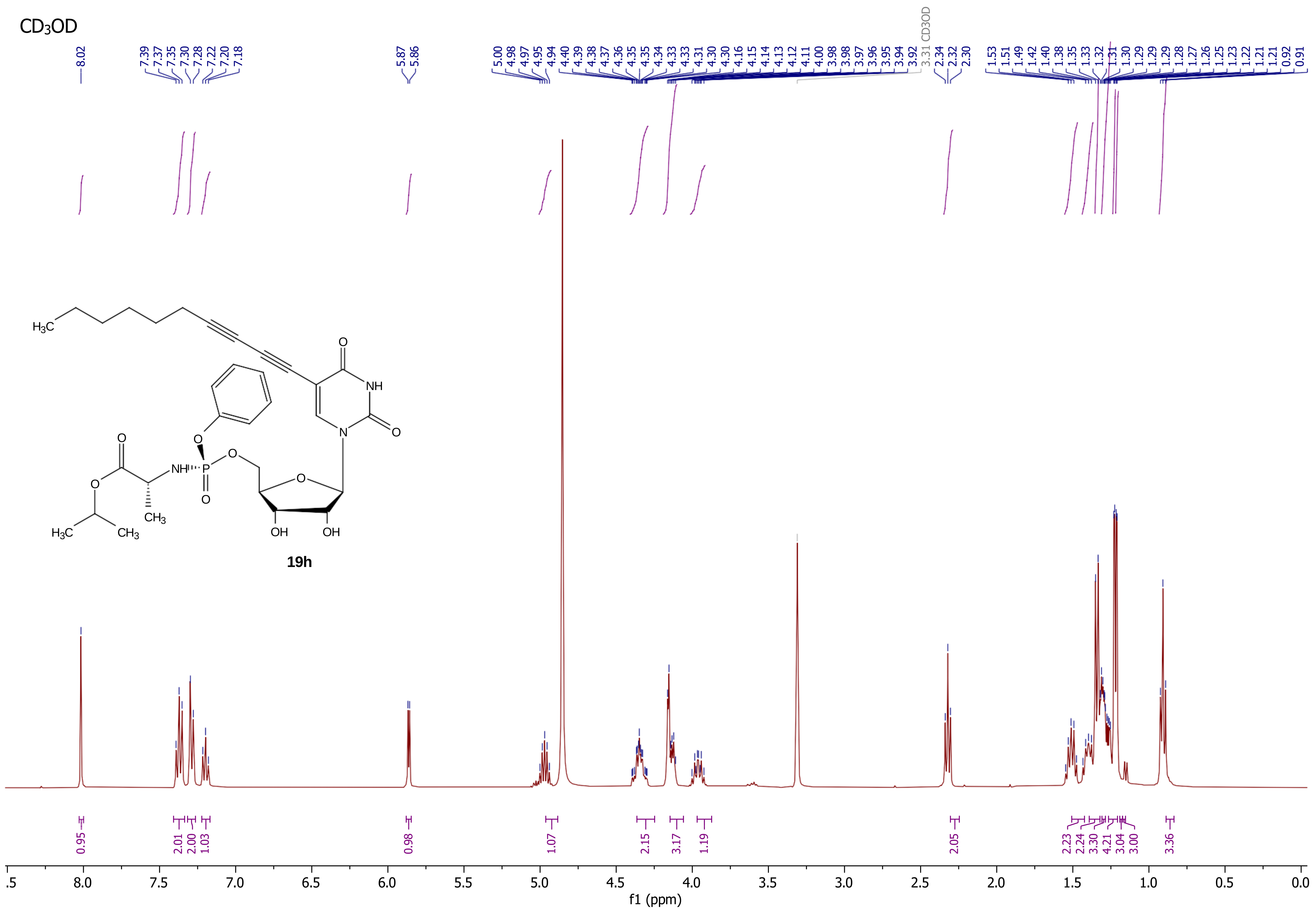
CD3OD
8.02
7.39
7.37
7.35
7.30
7.28
7.22
7.20
7.18
5.87
5.86
5.00
4.98
4.97
4.95
4.94
4.40
4.39
4.38
4.37
4.36
4.35
4.35
4.34
4.33
4.33
4.31
4.30
4.30
4.16
4.15
4.14
4.13
4.12
4.11
4.00
3.98
3.98
3.97
3.96
3.95
3.94
3.92
3.31 CD3OD
2.34
2.32
2.30
1.53
1.51
1.49
1.42
1.40
1.38
1.35
1.33
1.32
1.31
1.30
1.29
1.29
1.29
1.28
1.27
1.26
1.25
1.23
1.22
1.21
1.21
0.92
0.91
H3C
O
NH
N
O
O
NH
P
O
O
O
O
O
CH3
H3C
CH3
OH
OH
19h
0.95
2.01
2.00
1.03
0.98
1.07
2.15
3.17
1.19
2.05
2.23
2.24
3.30
4.21
3.04
3.00
3.36
.5
8.0
7.5
7.0
6.5
6.0
5.5
5.0
4.5
4.0
3.5
3.0
2.5
2.0
1.5
1.0
0.5
0.0
f1 (ppm)

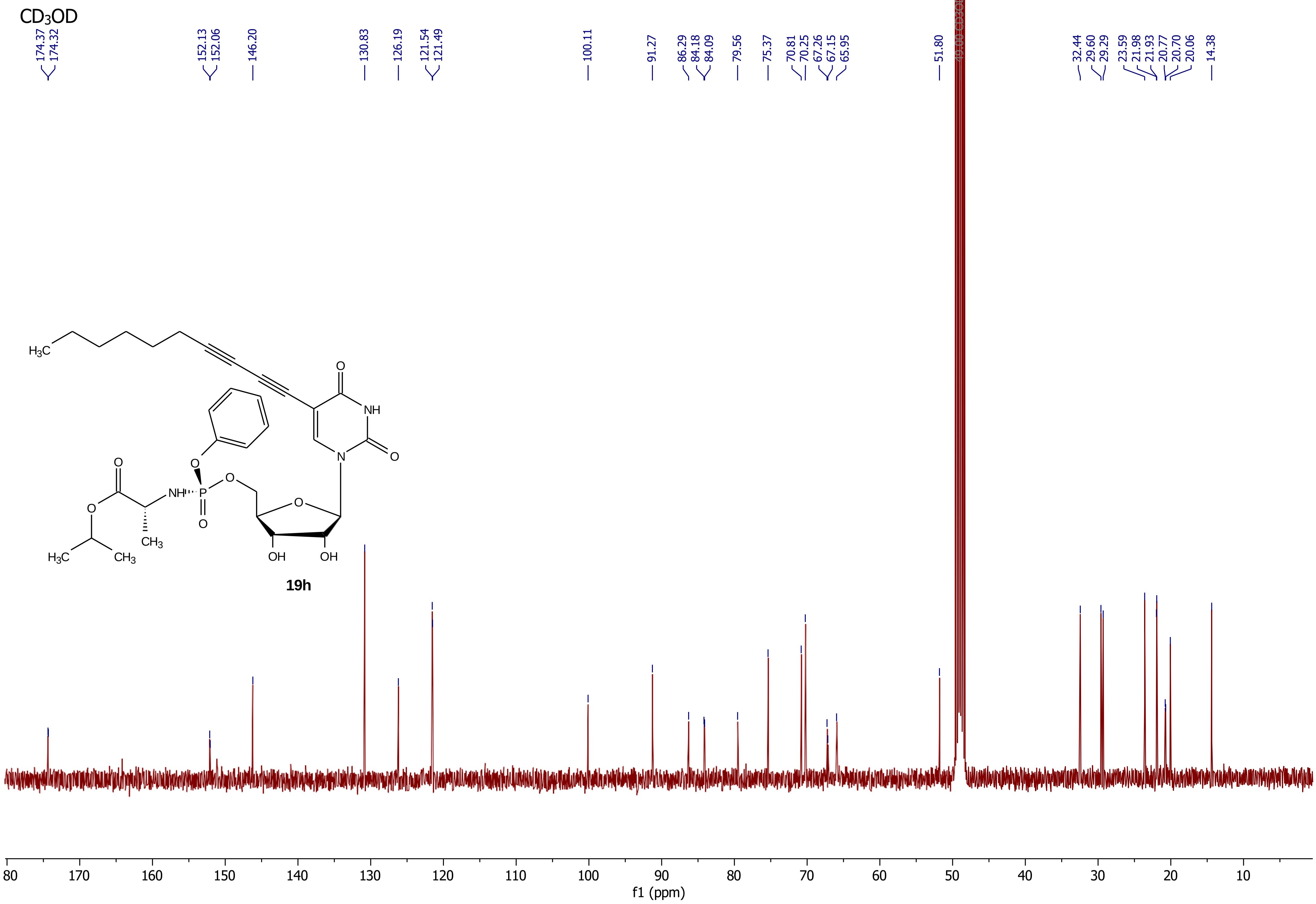
CD3OD
174.37
174.32
152.13
152.06
146.20
130.83
126.19
121.54
121.49
100.11
91.27
86.29
84.18
84.09
79.56
75.37
70.81
70.25
67.26
67.15
65.95
51.80
49.00 CD3OD
32.44
29.60
29.29
23.59
21.98
21.93
20.77
20.70
20.06
14.38
19h
f1 (ppm)
80
170
160
150
140
130
120
110
100
90
80
70
60
50
40
30
20
10

CD$_3$OD

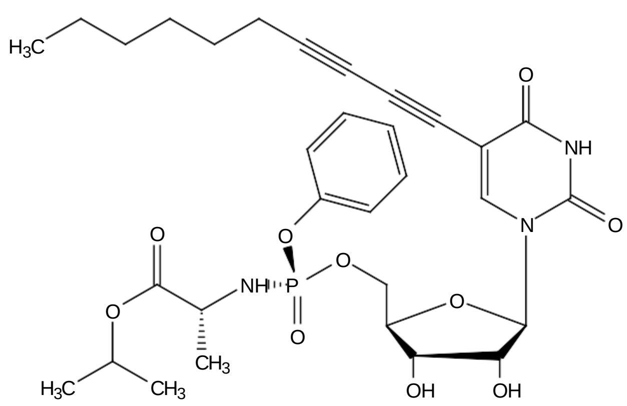

19h

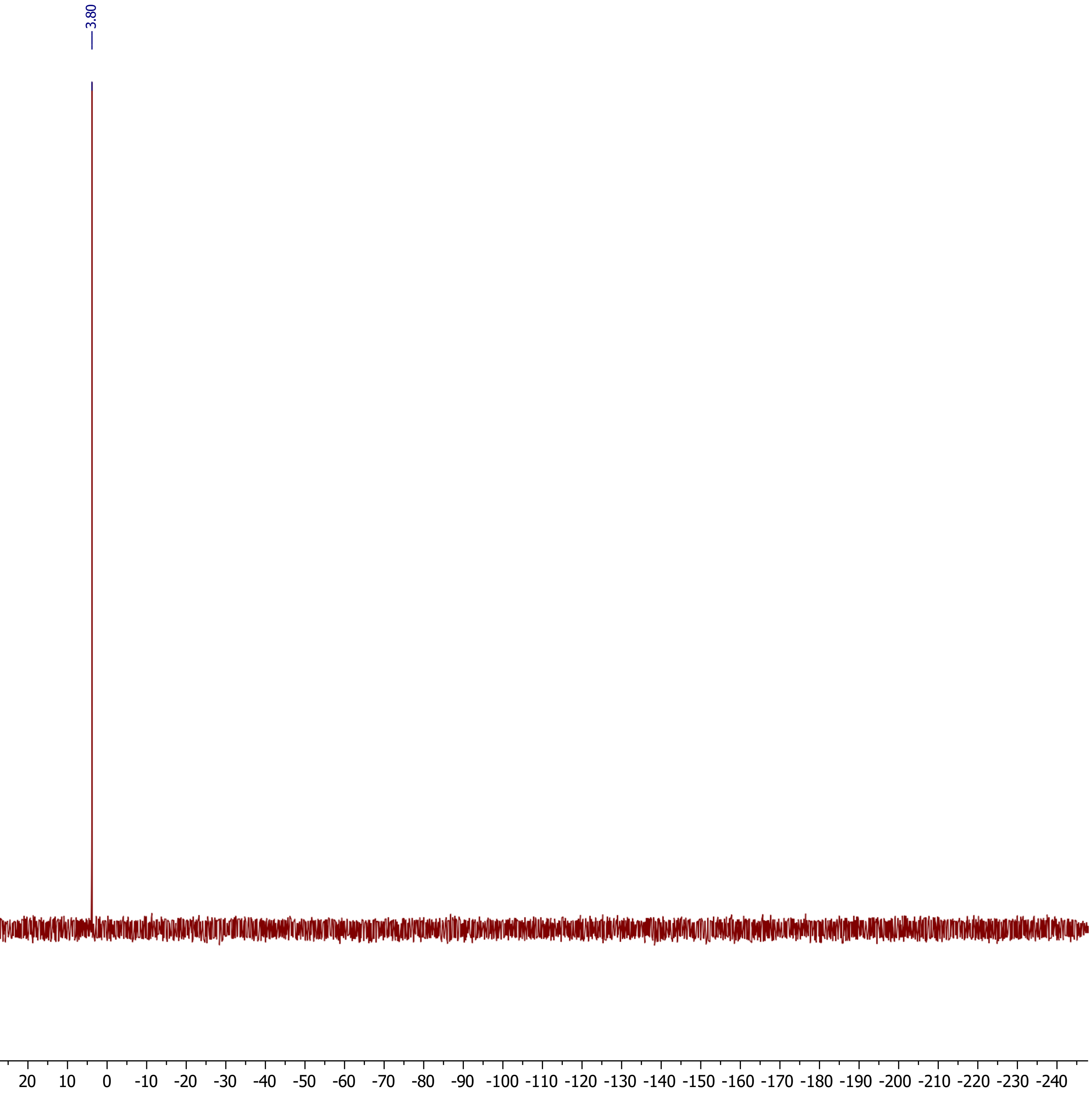
3.80
140 130 120 110 100 90 80 70 60 50 40 30 20 10 0 -10 -20 -30 -40 -50 -60 -70 -80 -90 -100 -110 -120 -130 -140 -150 -160 -170 -180 -190 -200 -210 -220 -230 -240

f1 (ppm)

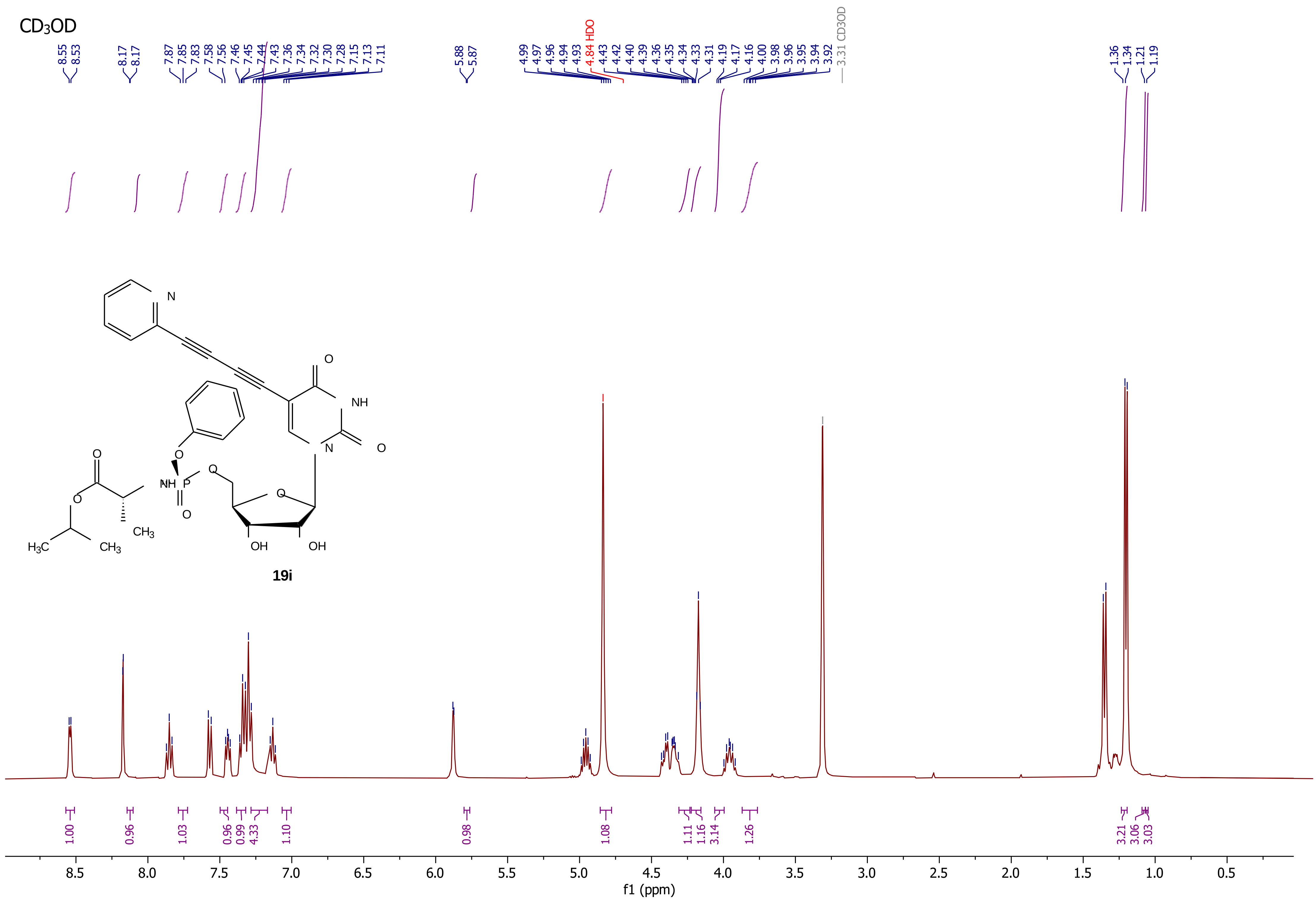
CD3OD
8.55 8.53 8.17 8.17 7.87 7.85 7.83 7.58 7.56 7.46 7.45 7.44 7.43 7.36 7.34 7.32 7.30 7.28 7.15 7.13 7.11
5.88 5.87
4.99 4.97 4.96 4.94 4.93 4.84 HDO 4.43 4.42 4.40 4.39 4.36 4.35 4.34 4.33 4.31 4.19 4.17 4.16 4.00 3.98 3.96 3.95 3.94 3.92
3.31 CD3OD
1.36 1.34 1.21 1.19
1.00 0.96 1.03 0.96 0.99 4.33 1.10 0.98 1.08 1.11 1.16 3.14 1.26 3.21 3.06 3.03
8.5 8.0 7.5 7.0 6.5 6.0 5.5 5.0 4.5 4.0 3.5 3.0 2.5 2.0 1.5 1.0 0.5
f1 (ppm)
N
O
NH
O
N
O
O
O
NH
P
O
O
O
OH
OH
H3C
CH3
CH3

19i

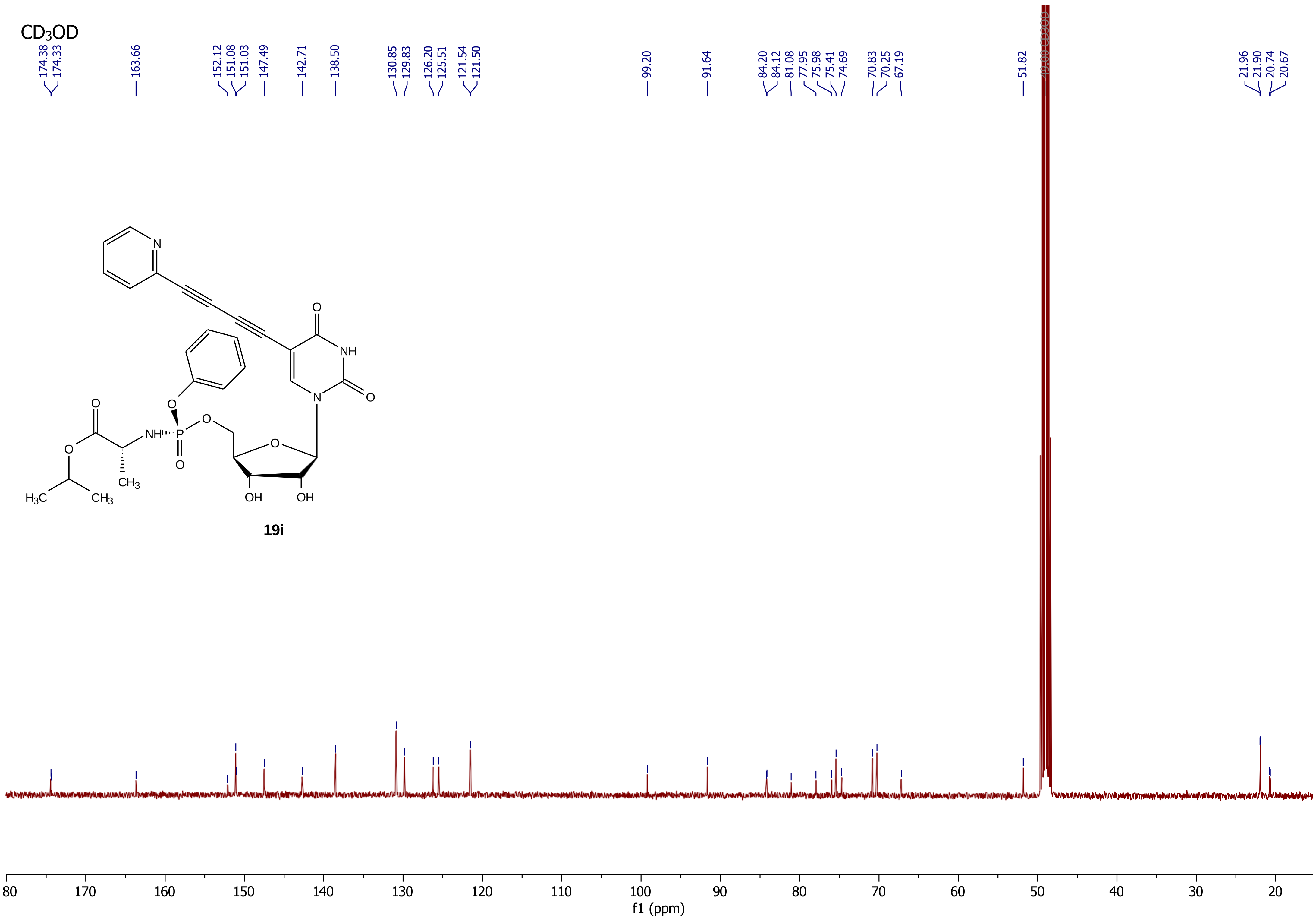
CD3OD
174.38
174.33
163.66
152.12
151.08
151.03
147.49
142.71
138.50
130.85
129.83
126.20
125.51
121.54
121.50
99.20
91.64
84.20
84.12
81.08
77.95
75.98
75.41
74.69
70.83
70.25
67.19
51.82
49.00 CD3OD
21.96
21.90
20.74
20.67
19i
80 170 160 150 140 130 120 110 100 90 80 70 60 50 40 30 20
f1 (ppm)

CD$_3$OD

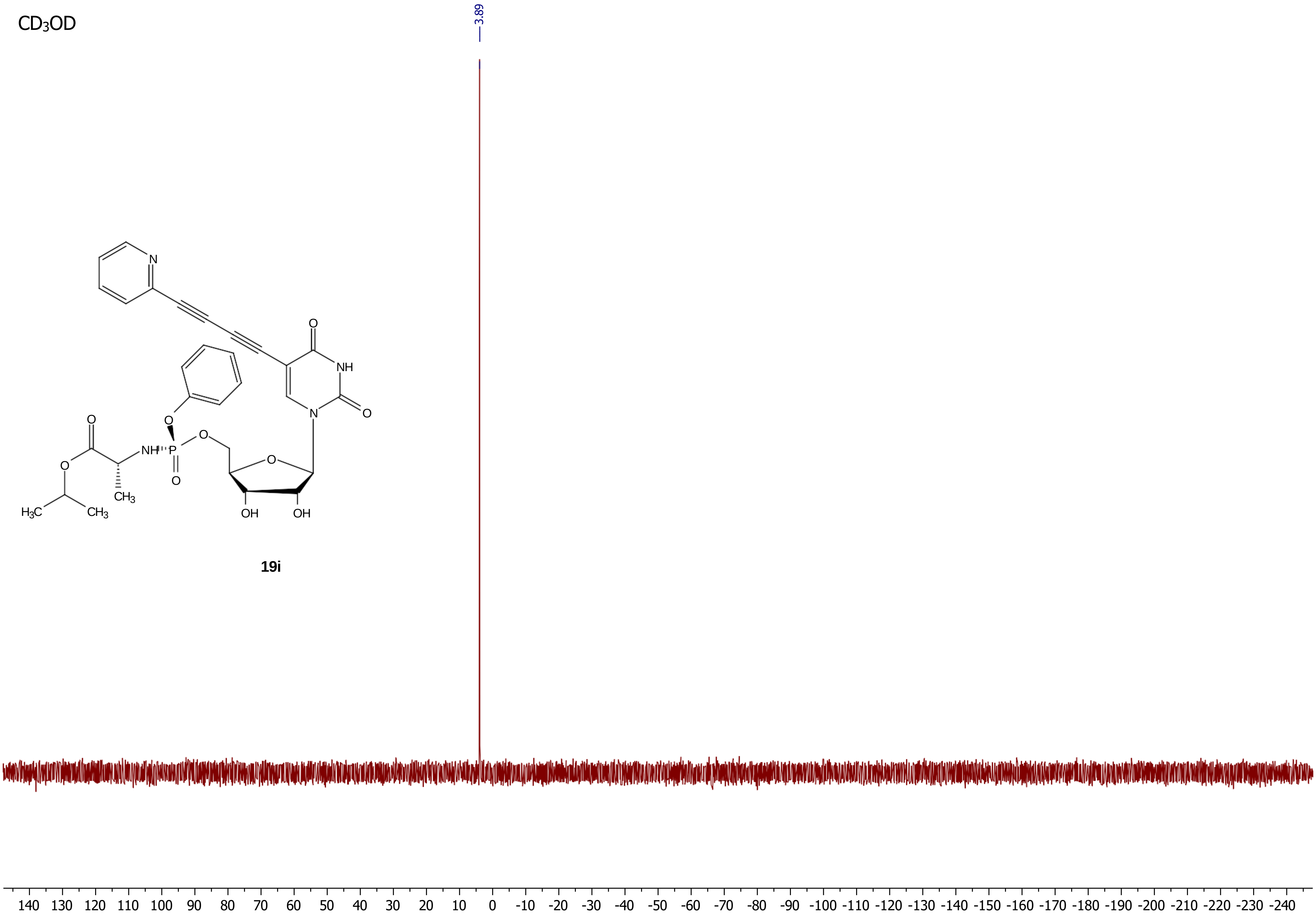
3.89
N
O
NH
O
N
O
O
O
NH
P
O
O
O
CH3
H3C
CH3
OH
OH
140 130 120 110 100 90 80 70 60 50 40 30 20 10 0 -10 -20 -30 -40 -50 -60 -70 -80 -90 -100 -110 -120 -130 -140 -150 -160 -170 -180 -190 -200 -210 -220 -230 -240

19i

f1 (ppm)

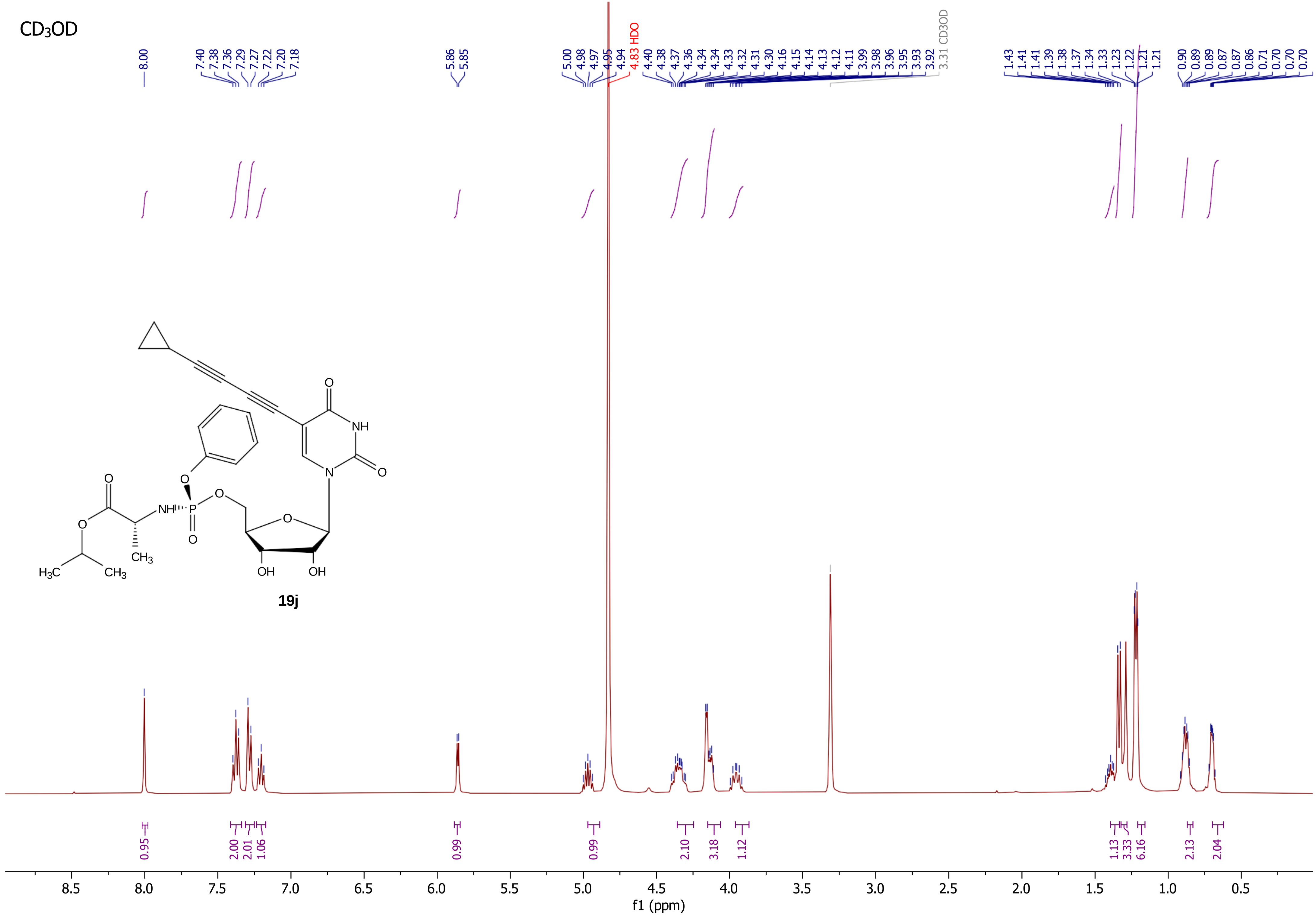
CD3OD
8.00
7.40 7.38 7.36 7.29 7.27 7.22 7.20 7.18
5.86 5.85
5.00 4.98 4.97 4.95 4.94
4.83 HDO
4.40 4.38 4.37 4.36 4.34 4.34 4.33 4.32 4.31 4.30 4.16 4.15 4.14 4.13 4.12 4.11 3.99 3.98 3.96 3.95 3.93 3.92
3.31 CD3OD
1.43 1.41 1.41 1.39 1.38 1.37 1.34 1.33 1.23 1.22 1.21 1.21
0.90 0.89 0.89 0.87 0.87 0.86 0.71 0.70 0.70 0.70
NH
O
N
OH
H3C
CH3
19j
0.95
2.00
2.01
1.06
0.99
0.99
2.10
3.18
1.12
1.13
3.33
6.16
2.13
2.04
8.5 8.0 7.5 7.0 6.5 6.0 5.5 5.0 4.5 4.0 3.5 3.0 2.5 2.0 1.5 1.0 0.5
f1 (ppm)

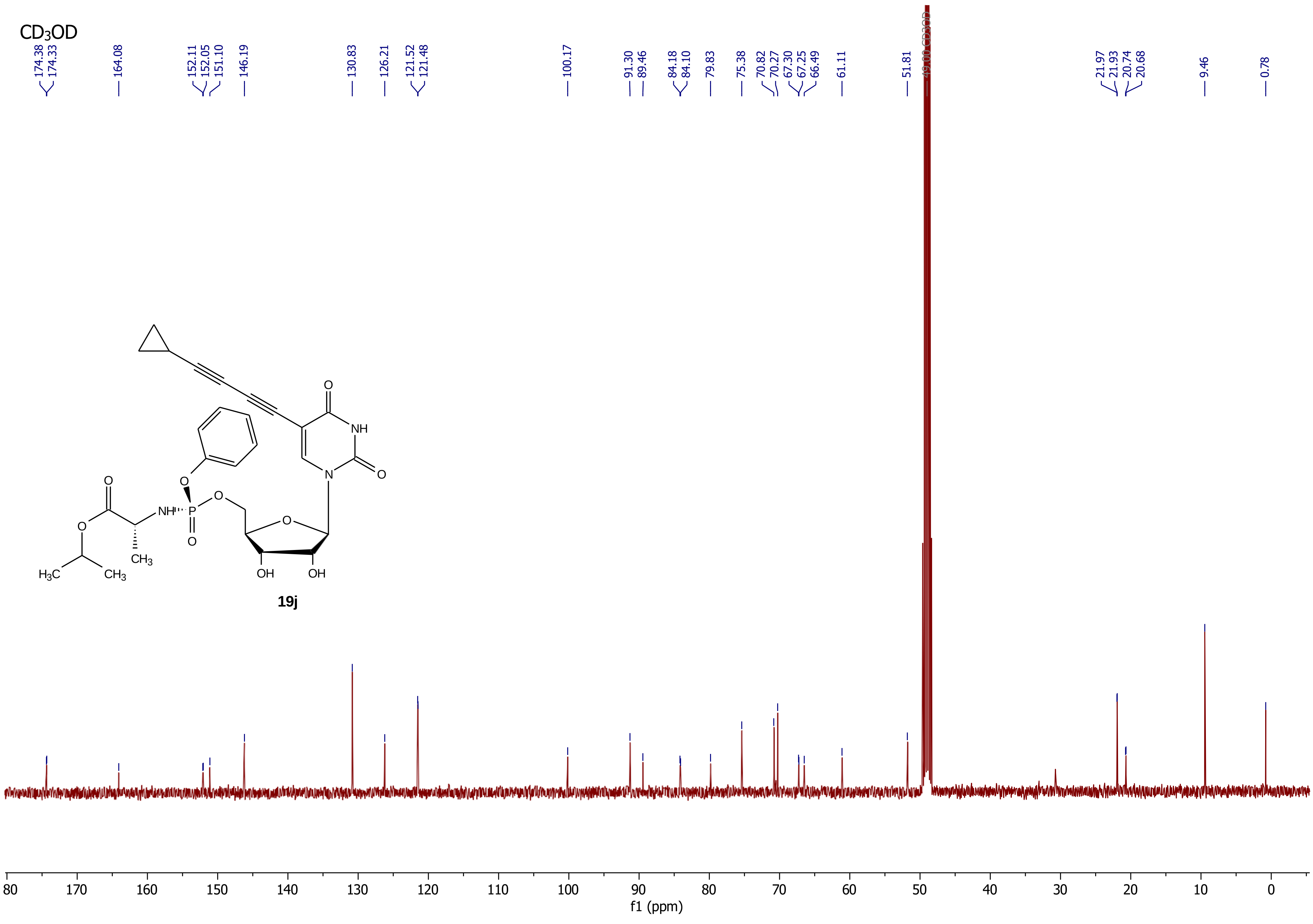
CD3OD
174.38
174.33
164.08
152.11
152.05
151.10
146.19
130.83
126.21
121.52
121.48
100.17
91.30
89.46
84.18
84.10
79.83
75.38
70.82
70.27
67.30
67.25
66.49
61.11
51.81
49.00 CD3OD
21.97
21.93
20.74
20.68
9.46
0.78
O
NH
O
N
O
O
NH
P
O
O
O
H3C
CH3
CH3
OH
OH
19j
80
170
160
150
140
130
120
110
100
90
80
70
60
50
40
30
20
10
0
f1 (ppm)

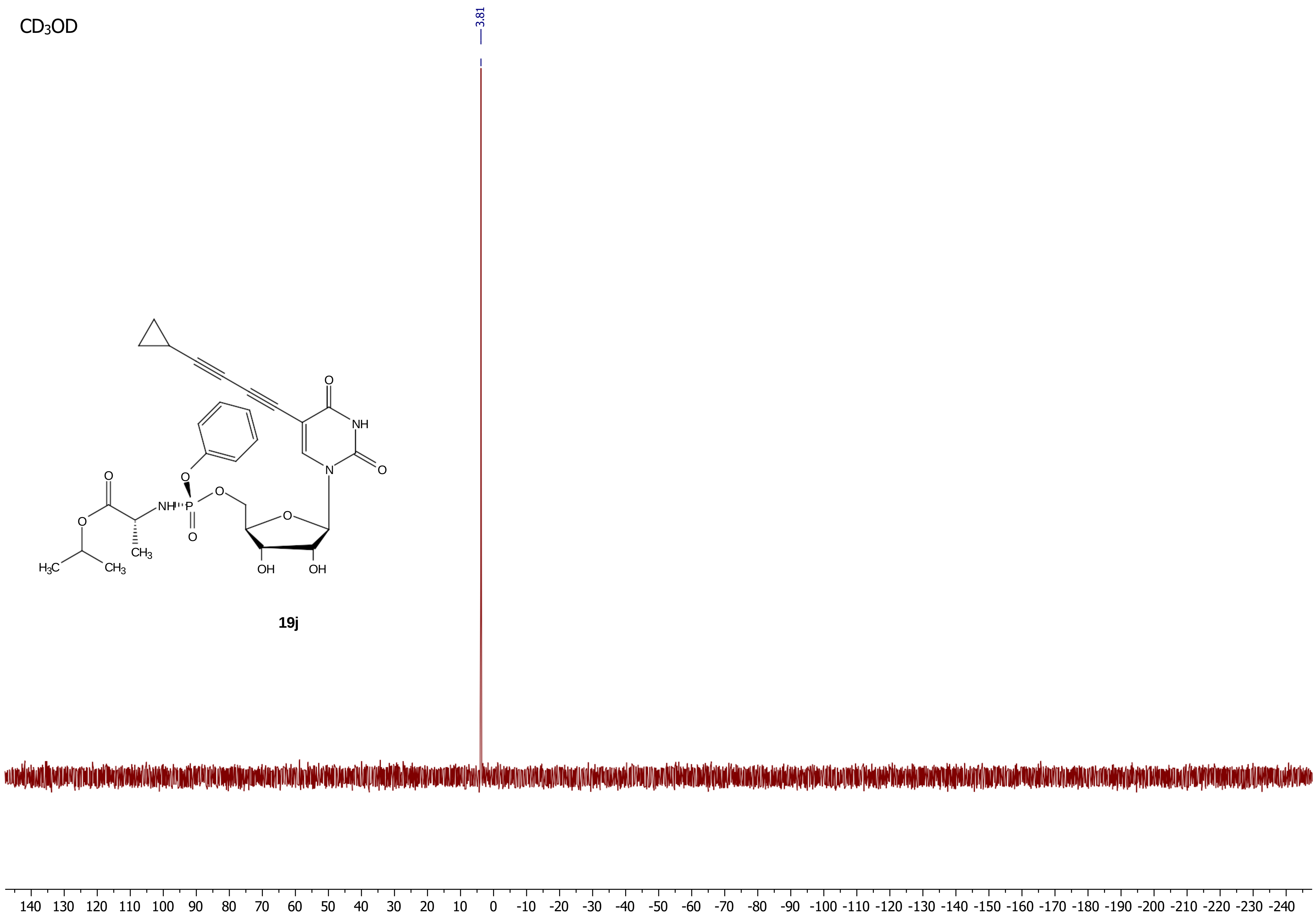
CD3OD
3.81
O
NH
O
N
O
O
NH
P
O
O
O
O
CH3
H3C
CH3
OH
OH
19j
140 130 120 110 100 90 80 70 60 50 40 30 20 10 0 -10 -20 -30 -40 -50 -60 -70 -80 -90 -100 -110 -120 -130 -140 -150 -160 -170 -180 -190 -200 -210 -220 -230 -240

f1 (ppm)

CD$_3$OD

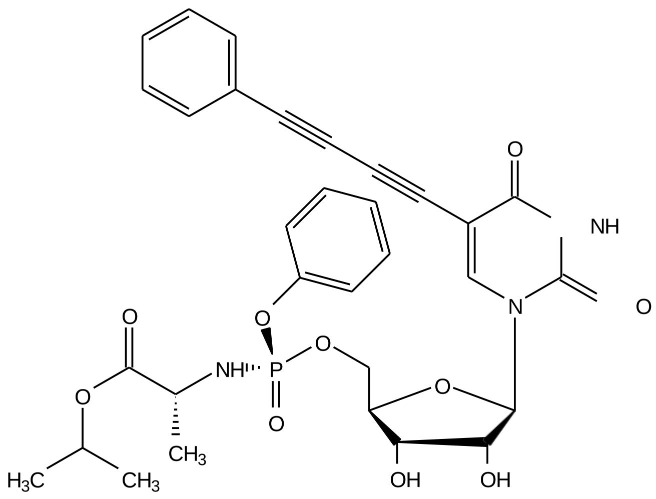
NH
O
O
O
NH
P
O
O
O
O
CH3
H3C
CH3
OH
OH
N

19k

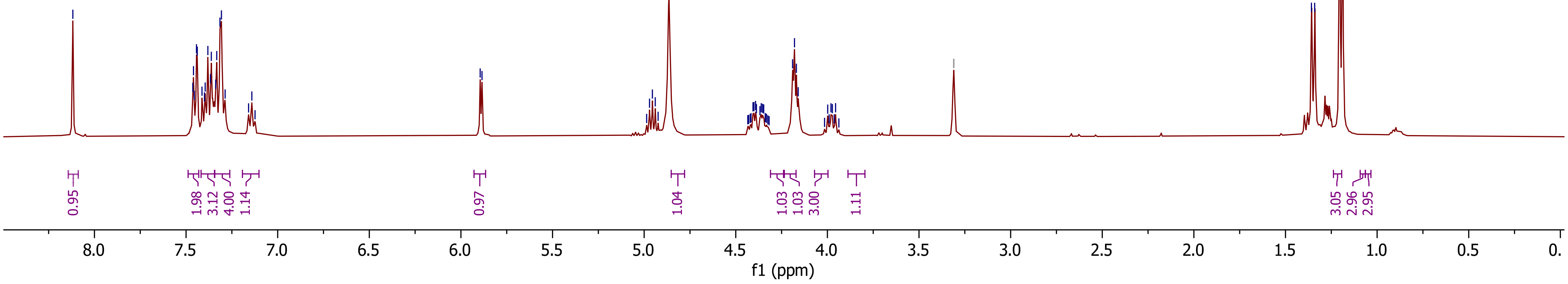
8.12
7.46
7.46
7.46
7.44
7.44
7.41
7.40
7.40
7.38
7.37
7.36
7.34
7.33
7.32
7.31
7.29
7.16
7.14
7.12
5.89
5.88
4.99
4.97
4.96
4.94
4.92
4.43
4.43
4.42
4.42
4.41
4.40
4.39
4.39
4.37
4.36
4.35
4.35
4.34
4.33
4.32
4.32
4.19
4.18
4.17
4.16
4.02
4.00
3.99
3.98
3.97
3.96
3.96
3.94
3.31 CD3OD
1.36
1.34
1.20
1.19
0.95
1.98
3.12
4.00
1.14
0.97
1.04
1.03
1.03
3.00
1.11
3.05
2.96
2.95
8.0
7.5
7.0
6.5
6.0
5.5
5.0
4.5
4.0
3.5
3.0
2.5
2.0
1.5
1.0
0.5
0.
f1 (ppm)

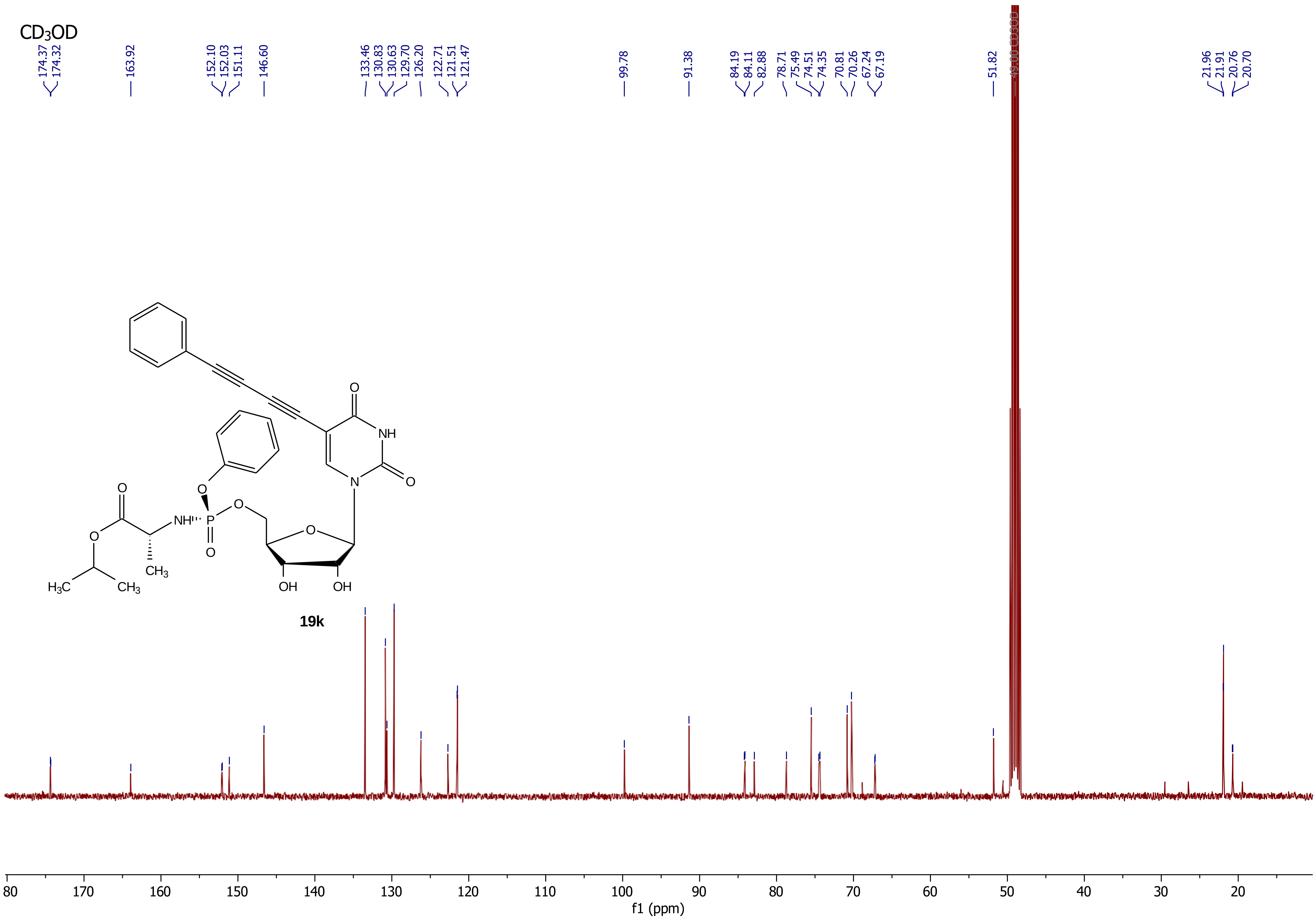
CD3OD
174.37
174.32
163.92
152.10
152.03
151.11
146.60
133.46
130.83
130.63
129.70
126.20
122.71
121.51
121.47
99.78
91.38
84.19
84.11
82.88
78.71
75.49
74.51
74.35
70.81
70.26
67.24
67.19
51.82
49.00 CD3OD
21.96
21.91
20.76
20.70
19k
80 170 160 150 140 130 120 110 100 90 80 70 60 50 40 30 20
f1 (ppm)

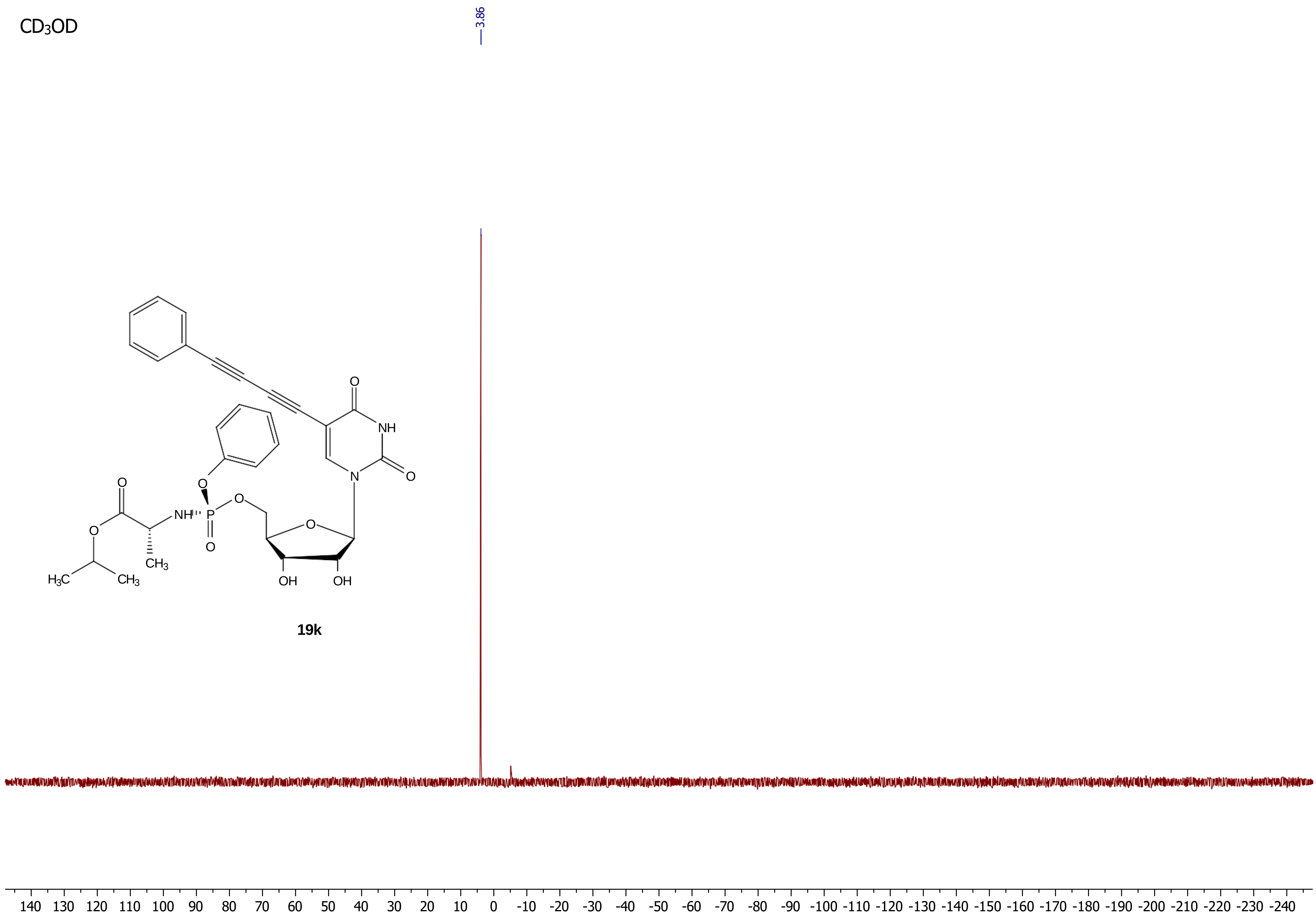
CD3OD
3.86
19k
140 130 120 110 100 90 80 70 60 50 40 30 20 10 0 -10 -20 -30 -40 -50 -60 -70 -80 -90 -100 -110 -120 -130 -140 -150 -160 -170 -180 -190 -200 -210 -220 -230 -240

f1 (ppm)
